# α-Halothioamide warheads with enhanced cysteine reactivity and specificity for covalent protein labelling

**DOI:** 10.1038/s41467-026-72993-6

**Published:** 2026-05-14

**Authors:** László Petri, Ronen Gabizon, Nikolett Péczka, Péter Ábrányi-Balogh, József Simon, Tímea Imre, György G. Ferenczy, Nir London, György M. Keserű

**Affiliations:** 1 Medicinal Chemistry Research Group, Research Centre for Natural Sciences, Budapest, Hungary; 2 National Laboratory of Pharmaceutical Research and Development, Research Centre for Natural Sciences, Budapest, Hungary; 3 Institute of Chemistry, Eötvös Loránd University, Budapest, Hungary; 4 Department of Chemical and Structural Biology, The Weizmann Institute of Science, Rehovot, Israel; 5 Department of Organic Chemistry and Technology, Budapest University of Technology and Economics, Budapest, Hungary; 6 MS Metabolomics Research Laboratory, Research Centre for Natural Sciences, Budapest, Hungary

**Keywords:** Chemical biology, Medicinal chemistry, Proteomic analysis

## Abstract

Covalent labelling is a promising modality relying on electrophilic warheads that form covalent bonds with their targets. Here we present the oxygen-to-sulfur exchange strategy, that transforms traditional carboxamides to thioamides, yielding electrophilic warheads, including chlorothioacetamides and fluorothioacetamides, with enhanced cysteine reactivity, while retaining aqueous stability and selectivity. We demonstrate their utility in targeted covalent inhibitor development targeting Janus kinase 3 and Bruton’s tyrosin kinase, when installed on relevant scaffolds and also in the development of antibody-drug conjugates. Next, alkyne-tagged α-halothioamide probes are evaluated by quantitative chemoproteomics. These studies reveal that the chlorothioacetamide probe preferentially labelled a distinct subset of the proteome, which results in Cys-targeted covalent phosphodiesterase 6δ labelling with functional impact. We discuss that the oxygen-to-sulfur exchange strategy offers alternative cysteine-specific thioamide-derived warheads and enables precise and even late-stage modulation of covalent reactivity, highlighting their promise for covalent probe design and applications in medicinal chemistry and chemical biology.

## Abstract (web summary)

Covalent labelling is a promising modality relying on electrophilic warheads that form covalent bonds with their targets. Here, authors present the oxygen-to-sulfur exchange strategy that transforms traditional carboxamides to thioamides, yielding several electrophilic warheads, including chlorothioacetamides and fluorothioacetamides, with enhanced cysteine reactivity, while retaining aqueous stability and selectivity.

## Introduction

Electrophilic compounds that form a covalent bond with their targets were historically avoided from drug discovery pipelines due to concerns about potential idiosyncratic toxicity and other pharmacological challenges related to insufficient selectivity^1,2^. Early covalent drugs, such as aspirin and penicillin, were typically discovered serendipitously. Recently, updated design principles have made targeted covalent ligands increasingly popular in chemical biology and drug discovery applications, addressing mostly antiinfective^3–6^ and oncology-related indications^7–13^. A key feature of covalent ligands is the electrophilic ‘warhead’^14^, which reacts with a nucleophilic residue to form a covalent bond at the binding site of the targeted protein. Since the turn of the millennium, numerous electrophilic warheads have been developed, with a significant number of functional groups available for covalent labelling^15^. Cysteine residues are frequently targeted due to their increased nucleophilicity and relatively low abundance in the human proteome. These characteristics have driven innovative covalent-targeting strategies in fragment-based drug discovery^16–18^, computer-based analysis of druggable targets^19,20^, and possible therapeutic applications^12,21–24^. One critical principle of covalent design is tailoring the warhead’s reactivity to the target^22,23,25–27^. For instance, minor changes in the warhead could significantly alter ligand binding mode, labelling efficiency, and binding affinity when targeting Cys12 in the oncogenic KRAS^G12C^ mutant protein^28,29,23,30,31^. Given the diverse local protein environments around targeted cysteines and the different reactivities, topology, sterics and polar features of warheads^16,32–36^, optimization of warheads is crucial in the development of targeted covalent inhibitors (TCIs)^37,38^. As a consequence, chemistries that can tune the reactivity of the electrophile without significantly changing its geometry can be especially useful for late-stage inhibitor optimization^39–41^.

In this work, we report the development of cysteine-specific α-halothioamide warheads through the transformation of carboxamides into thioamide derivatives based on the Hard-Soft Acid-Base (HSAB) theory^41^. We discuss the preparation, characterization and application of these warheads and show oxygen-to-sulfur exchange as a valuable strategy for TCI development, conjugation of small molecules to antibodies and also for chemoproteomic applications.

## Results and discussion

Considering the soft nucleophilic character of cysteine, labelling with a softer electrophilic warhead might be more effective and specific. Therefore, we picked chloroacetamides as cysteine-reactive warhead chemotype, and turned the amide function into a thioamide. Moreover, we hypothesized that oxygen-to-sulfur exchange might activate non-reactive fluoroacetamides and prepared these analogues as well, potentially introducing novel covalent warheads with intriguing potential for medicinal chemistry and chemical biology applications. In contrast to fluoromethyl ketones^42–44^, fluoroacetamides are rarely considered as electrophiles^26,27,45–50^ and they are typically not reactive enough for the covalent modification of biomolecules. Although the C=S bond is not directly engaged in the reaction, its electronic effect strongly influences the adjacent α-position. To support this, we performed DFT calculations (M062X/6-311 + + G(d,p)) and analyzed the NBO-charge distribution localized to the adjacent α-carbon atom. These results consistently showed a positive shift of charge, indicating its increased electrophilicity (Supplementary Table S1), which provides a straightforward explanation for the higher reactivity observed with α-halothioamide electrophiles.

### Synthesis and characterization of α-halothioamide warheads

**Fig. 1:**
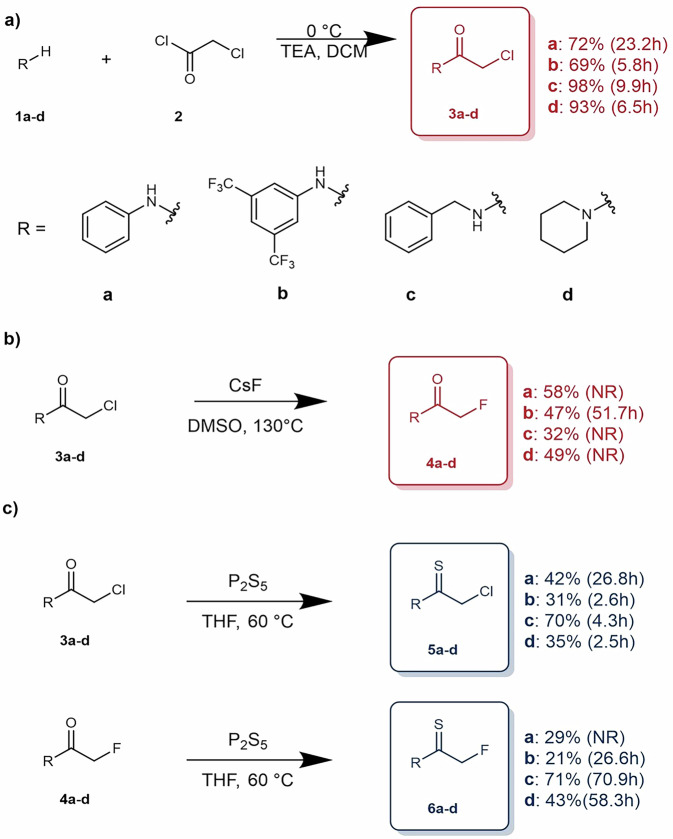
Synthetic procedures and thiol reactivity profiling of fragment electrophiles (**3-6**). Isolated yields and intrinsic thiol reactivity are indicated for each derivative. Glutathione (GSH) reactivity of electrophiles **3**–**6** were evaluated in PBS buffer (pH 7.4) at 25 °C using 0.25 mM fragment and 5 mM GSH. For each compound, the GSH half-life determined under these conditions is given in parentheses. Compounds labelled NR (not reactive) exhibited half-lives exceeding the maximal incubation period (72 h) under the assay conditions. (**a**) Preparation of α-chloroacetamide fragments (**3**) by acylation of corresponding amines with chloroacetyl chloride in the presence of TEA in DCM at 0 °C. (**b**) Conversion of α-chloroacetamides (**3**) to the corresponding α-fluoroacetamides (**4**) via halide exchange using CsF in DMSO at 130 °C. (**c**) Thionation of α-chloroacetamides (**3**) and α-fluoroacetamides (**4**) with P₂S₅ in THF at 60 °C to afford the corresponding thioamides (**5,6**), respectively.

To validate the concept of O-to-S exchange we developed a feasible procedure for the synthesis of the α-halothioamide warheads (Fig. 1)^51–53^. Chloroacetamides (**3a-d**) were obtained by *N*-acylation of anilines (**1a-d**) with the corresponding acyl chloride (**2**) (Fig. 1a). Next, the chloroacetamides (**3a-d**) were fluorinated by caesium-fluoride (Fig. 1b), leading to the corresponding fluoroacetamides (**4a-d**). Finally, we transformed the haloacetamides (**3a-d, 4a-d**) into halothioacetamides by applying phosphorus pentasulfide in THF (Fig. 1c) to obtain chlorothioacetamides (**5a-d**) and fluorothioacetamides (**6a-d**). Noteworthy, in addition to haloacetamides, the strategy was evaluated on acrylamide substrates (Supplementary Fig. S1), including phenylacrylamide (**S3a**), which can be considered as a benchmark electrophile to enable contextual comparison with other studies. While thioacrylamides could in principle be formed in a similar reaction, only phenylthioacrylamide proved sufficiently stable to be isolated in pure form. In contrast, five additional thioacrylamide derivatives could not be obtained due to rapid secondary reactivity and decomposition during workup and purification. Notably, conversion of phenylacrylamide (**S3a**) to the corresponding thioacrylamid (**S6a**) resulted in a pronounced increase of electrophilic reactivity, displaying hyperreactive behaviour in GSH-assay. However, owing to the limited stability and accessibility of acrylamide-derived thioamides, these compounds were not pursued further in this study.

We evaluated this comprehensive set of model electrophiles using our covalent fragment characterization workflow^37^. In order to test the reactivity of the warheads against a thiol-surrogate, we executed an HPLC-based aqueous stability and a kinetic l-glutathione (GSH) reactivity assay^18,37^. Half-lives were determined in PBS (pH 7.4) at room temperature using 250 µM electrophile and 5 mM GSH. Reported values were corrected for aqueous degradation of the electrophiles. We compared chloroacetamides (**3a-d**) and fluoroacetamides (**4a-d**) and their α-halothioamide analogues (**5a-d** and **6a-d**), respectively (Fig. 1, Supplementary Table S2). Our results confirmed that O-to-S exchange positively contributed to the thiol-reactivity of the covalent probes investigated herein, without compromising aqueous stability. The reactivity enhancement was more modest for chlorothioacetamides (**9a-b**), but still, the reactivity of **5b** is two times higher than its **3b** carboxamide analogue. An even higher contrast can be seen for the fluoroacetamide **4b** that has been converted into a more reactive fluorothioacetamide (**6b**); this warhead chemotype nearly doubled its reactivity under our assay conditions. Aqueous stability results demonstrated that thiocarbonyl analogues are stable and suitable for biological applications, as their half-life exceeds 72 h for all measured probes (**5a-d** and **6a-d**). For the assessment of amino acid selectivity of these electrophiles, we implemented an LC-MS/MS-based assay^37^ utilizing a nonapeptide (KGDYHFPIC) having multiple nucleophilic residues (Cys, Lys, Tyr). Although the terminal position of Lys and Cys residues might impact their reactivity, this well-established assay with the same peptide had been applied for multiple previous covalent drug discovery campaigns^23,30,37,54,55^. These measurements confirmed that cysteine selectivity was not changed after O-to-S exchange (Supplementary Table S2 and Fig. S2). Altogether, our data suggest that O-to-S exchange could be an attractive strategy for tailoring electrophiles, which in most cases increases thiol reactivity without significantly altering electrophile geometry. As observed for other warhead modifications, its impact depends on the electronic properties of the noncovalent scaffold and the nucleophilicity of the targeted residue^37^.

**Fig. 2:**
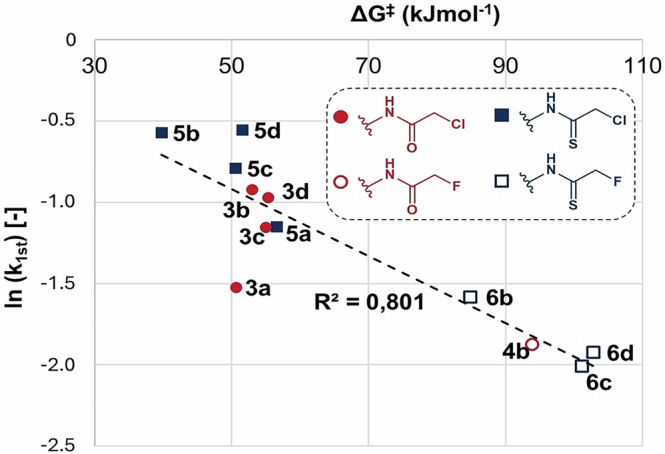
Correlation of calculated activation energy barriers (ΔG^‡^) and experimental reactivity (ln*k*_1st_) of amide (red) and thioamide (blue) probes. Activation Gibbs free energies (ΔG‡) for the reaction of haloacetamide (**3-4**) and halothioacetamide (**5-6**) probes with the cysteine surrogate methyl thiolate (MeS⁻) were calculated by DFT applying the SMD implicit solvation model (water) and the M062X functional with the 6-311 G+(d,p) basis. Calculated Gibbs free energies (ΔG‡) for the reaction of haloacetamide (**3-4**) and halothioacetamide (**5-6**) probes with the cysteine surrogate methyl thiolate (MeS⁻) were plotted against experimentally determined reactivity values expressed as ln(k_1st_) = ln2/t_1/2_. Red circles denote amide probes and blue squares denote the corresponding thioamide analogues; filled symbols represent chloro derivatives and unfilled symbols represent fluoro derivatives. The dashed line indicates the linear regression fit (R² = 0.801), showing that lower calculated activation barriers correlate with higher experimental reactivity.

Next, we rationalized the observed reactivity differences of haloacetamides by DFT calculations. The activation energy barriers^56,57^ (ΔG^‡^) against the cysteine surrogate methyl thiolate anion (MeS^−^) were estimated^58–61^ considering an S_N_2 mechanism with the nucleophilic attack not involving the carbonyl/thiocarbonyl moiety. Computed activation energies (Supplementary Table S3) were then plotted against experimental ln(k_1st_) = ln2 / t_1/2_ values (Fig. 2). The trend line (R^2^ = 0.801) shows that lower activation Gibbs free energy values (ΔG^‡^) belong to compounds reacting faster (having higher reactivity), as expected. Comparing the amide and thioamide pairs, the largest differences were obtained for **4d-6d** (25.4 kJ mol^−^^1^), and for **3b-5b** (13.2 kJ mol^−1^), which was in line with the reactivity enhancement observed half-lives: non-reactive vs. 58.3 h and 5.8 h vs. 2.6 h, respectively. In the case of **4a-6a** (2.6 kJ mol^−1^) there was no significant difference in the barriers, and the compounds were similarly non-reactive.

**Fig. 3:**
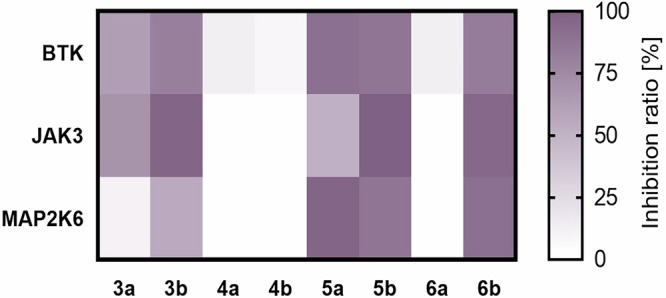
Inhibition efficiency of covalent fragments (3a-b, 4a-b, 5a-b, 6a-b) on BTK, JAK3 and MAP2K6 kinases. Reported as inhibition percentage measured at 100 μM concentration of the probes from biological duplicates after 60 min preincubation. For detailed results, see Supplementary Table S5.

Next, we aimed to evaluate the utility of thioamide-derived warheads in protein labelling. Thus, we performed protein-level reactivity evaluation on protein kinases frequently targeted in covalent drug discovery campaigns^23,62,63^. We have included the phenyl and 3,5-bis(trifluoromethyl)phenyl analogues of the carbonyl and thiocarbonyl haloacetamides in order to see the activating effect of the heteroatom and the electron-deficient aromatic ring on reactivity. To evaluate their kinase targeting potential, we have chosen three covalently druggable kinases: JAK3 (Janus kinase 3), BTK (Bruton’s tyrosine kinase) and MAP2K6 (mitogen-activated protein kinase kinase 6). Targeted cysteines and their reactivity and accessibility were characterized by the Cy-preds server (Supplementary Table S4)^64^. Importantly, the targeted cysteine residues in BTK and JAK3 are located at an equivalent position within the kinase front pocket, a well-established hotspot for covalent inhibition. In contrast, the targetable cysteine in MAP2K6 resides in the DFG region, representing a distinct structural and functional context. Interestingly, kinases characterized by similar accessibility showed significantly different reactivity as suggested by calculated pK_a_ values. However, it should be noted that such estimates are inherently approximate and may be affected by protein dynamics, local microenvironment, and ligand-induced conformational changes. Kinase activity was assessed at 100 μM probe concentration with selected kinases (after 60 min incubation in duplicates), and their inhibitory activity is summarized in Fig. 3 (for detailed results see Supplementary Table S5). Our results revealed a marked increase in target inhibition due to O-to-S exchange. The activity of 2-chloro-*N*-phenylthioacetamide **3a** was twice as high compared to **4a**, and an almost identical pattern could be observed by the 3,5-bis(trifluoromethyl)phenyl (BTF) analogues (**3b**/**4b**). Interestingly, the bis(trifluoromethyl) substitution of thioamide **5b** decreased the JAK3 inhibition. For **3b** a similar effect could be seen against MAP2K6. Fluoroacetamides **4a** and **4b** were non-reactive across the kinases we investigated, independently of the electron-withdrawing substitution. 2-Fluoro-*N*-phenylthioacetamide (**6a**) was also inactive, but its bis(trifluoromethyl) analogue **6b** showed a large increase in kinase inhibition against all targets, which is consistent with the expected electron-withdrawing effect of the substituents. To confirm the covalent mechanism of action, we performed MS measurements on all investigated kinase targets. Covalent labelling of BTK and MAP2K6 was confirmed for biochemically active fragment probes (**3a,** **3b,** **5a,** **5b,** **6b**) by intact MS measurements (Supplementary Fig. S3 and Table S6). Additionally, the labelling site of JAK3 was detected by MS/MS measurements of trypsin-digested protein for all biochemically active probes (**3a,** **3b,** **5a,** **5b,** **6b**) on residue Cys909, which is in proximity to the ATP-binding active site (Supplementary Fig. S4 and Table S7). The observed degrees of labelling across BTK, MAP2K6, and JAK3 must be interpreted in the context of markedly different probe-to-protein stoichiometries. BTK showed substantial labelling at relatively low probe excess (20 eq.), while MAP2K6 required higher probe excess (~70 eq.) to achieve comparably high labelling, suggesting a stronger dependence on on-target intrinsic reactivity. In contrast, JAK3 exhibited generally low labelling despite the high probe excess (~170 eq.), with only chlorothioacetamide probe (**5a**) showing significant modification. However, as MS measurement conditions were not standardized across the kinase targets, the heterogeneous labelling efficiencies should be considered rather qualitatively, and no robust quantitative conclusions can be drawn from these data. Across the majority of proteins and compounds, intact protein MS analysis revealed exclusively 1:1 protein-compound adducts. In fact, higher-order adducts (double and triple labelling) were only observed for MAP2K6 after treatment with the most reactive covalent fragments (5a, 5b, 6b). This behaviour is consistent with the fragment-sized nature of the compounds, which at this early stage lack extensive non-covalent secondary interactions and may therefore modify multiple accessible cysteine residues, and is in line with MAP2K6 in chemoproteomic data that identified labelling multiple cysteine sites^65–69^. Nevertheless, these results emphasized that the reactivity of the amide warheads can be modulated by both the O-to-S exchange and the conjugated noncovalent scaffold. In addition, the fluorothioacetamide (**6**) functionality described here represents a useful electrophile for chemical biology and medicinal chemistry applications.

### Development of α-halothioamide-based targeted covalent inhibitors

**Fig. 4:**
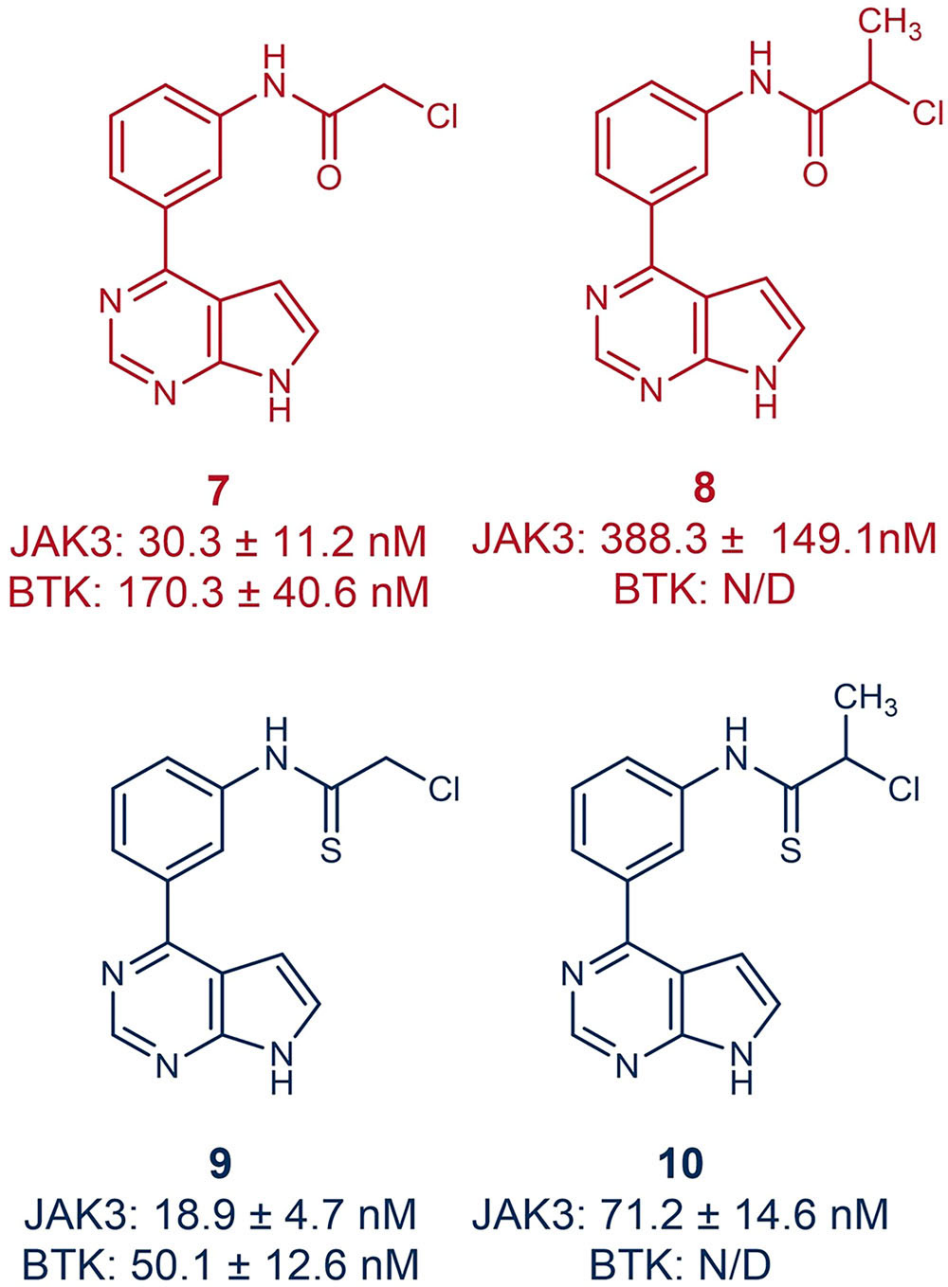
JAK3-targeting covalent probes with their biochemical activity on JAK3 and BTK inhibition. Reported as IC_50_ averages with standard errors, measured from biological duplicates after 5 min preincubation (N/D stands for “not determined in measured concentration range: 0.05 nM–1000 nM”).

Further exploring the utility of O-to-S exchange as a late-stage warhead modification, we applied the strategy to a previously published JAK3 inhibitor series developed by our fragment-based cysteine mapping methodology^23^. In this series (Fig. 4), we had identified an active chloroacetamide (**7**, IC_50_^JAK3^ = 30.3 ± 11.2 nM, IC_50_^BTK^ = 170.3 ± 40.6 nM) and a moderately active methyl-substituted chloroacetamide (**8**, IC_50_^JAK3^ = 388.3 ± 149.1 nM, and with no BTK activity at 1 μM). The methyl group on the adjacent α-carbon of the chloroacetamide sterically hinders the electrophilic center and exerts a +M electronic effect, reducing electrophilicity, which resulted in lower potency on all investigated targets. Both compounds display good selectivity for JAK3 over related kinases, including BTK, despite their close phylogenetic relationship and conserved ATP-binding site (Supplementary Table S4 and Fig. S5). To test the O-to-S exchange strategy, both chloroacetamide derivatives were converted into their chlorothioacetamide analogues (**9,10**) using phosphorus pentasulfide (according to Fig. 1c). Subsequently, we evaluated the JAK3 potency and selectivity of the sulfur-containing analogues (Fig. 4, Supplementary Fig. S6). For the highly active α-chloroacetamide (**7**), O-to-S exchange (resulting in α-chlorothioacetamide **9**) improved JAK3 potency (30.3 ± 11.2 nM versus 18.9 ± 4.7 nM, respectively) while selectivity over BTK was not significantly altered, as reflected by Δ*p*IC_50_^JAK3/BTK^ values of 0.75 for probe **7** and 0.46 for probe **9**. Remarkably, the methyl-chlorothioacetamide analogue (**10**) exhibited a substantial increase in potency on JAK3, resulted by the O-to-S exchange (388.3 ± 149.1 nM versus 71.2 ± 14.6 nM, respectively) while remaining practically inactive on BTK at 1 µM, demonstrating that even electronically attenuated and sterically hindered warheads could benefit from O-to-S exchange. Finally, covalent engagement of JAK3 by the chlorothioacetamide analogues (**9-10**) was confirmed using intact protein mass spectrometry, verifying covalent bond formation (Supplementary Fig. S3 and Table S6). These results provide clear proof-of-concept that O-to-S exchange can enhance potency without compromising selectivity and can be applied to design targeted covalent inhibitors.

**Fig. 5:**
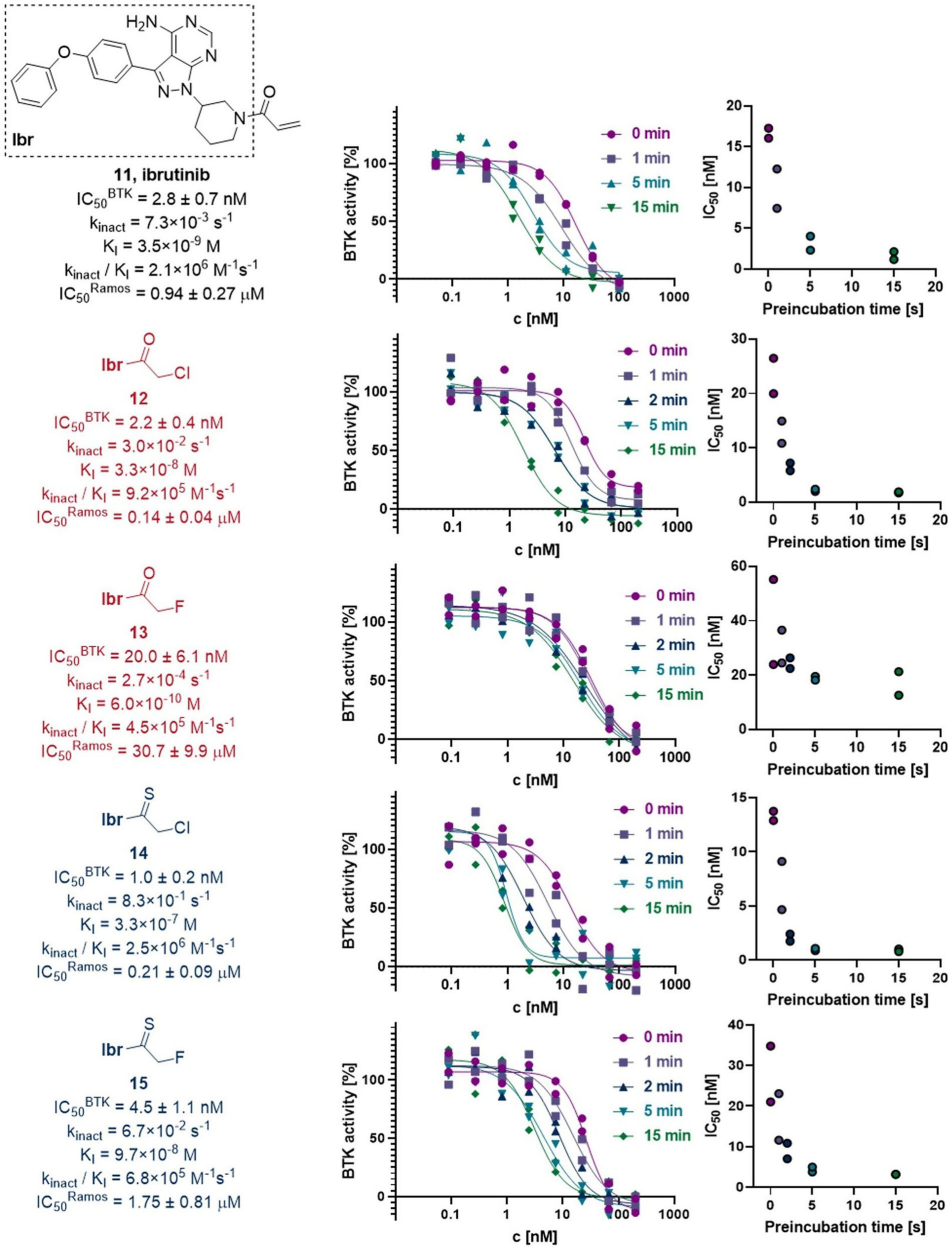
Biochemical activity and kinetic characterization of BTK targeting ibrutinib-derivative probes (11−15). Reported as IC_50_ averages with standard errors, measured from biological duplicates after 5 min preincubation. Time-dependent IC_50_-curves and subsequent depiction of the activity against the length of the preincubation are also shown, respectively. For kinase IC_50_ determination, the results were reported as mean ± SE calculated from two biological replicates, while for cell viability IC_50_ determination the results were reported as mean ± SD calculated from two biological replicates.

**Fig. 6:**
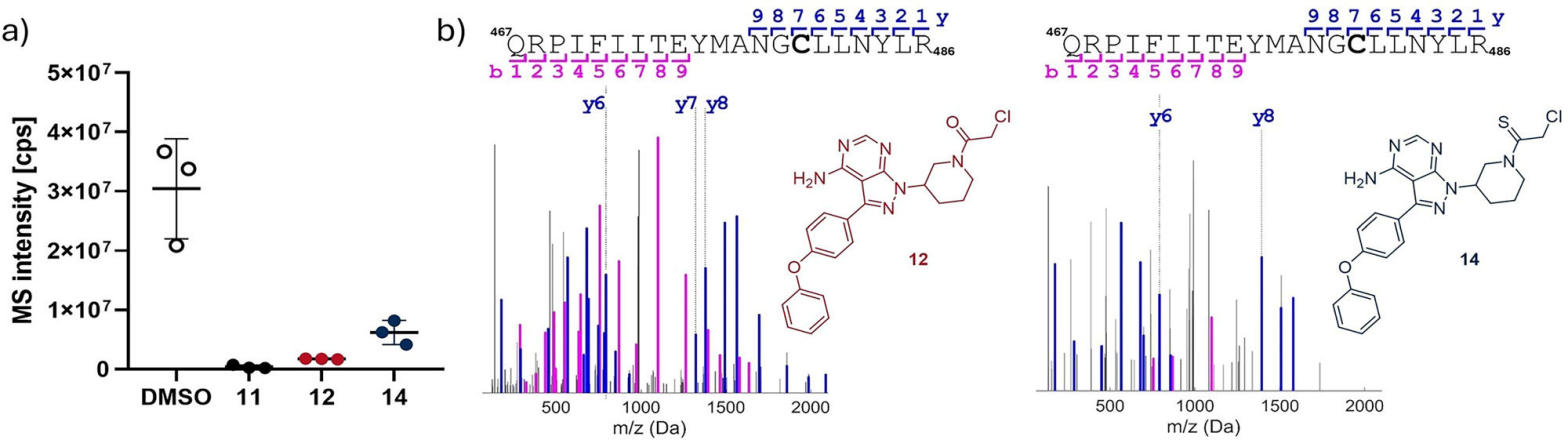
Tryptic digestion analysis of BTK protein labelled with 11, 12 and 14 covalent probes. **a** Sum of MS2 intensities related to CAM-labelled Cys481 in Glu467-Arg486 tryptic fragment of BTK protein. Results are shown as the individual values and mean with SD for triplicated experiments of DMSO, **11,** **12,** **14** treated BTK. **b** MS2 spectrum of **12,** **14** Glu467-Arg486 tryptic fragment of BTK protein treated with **12** α-chloroacetamide and **14** α-chlorothioacetamide showing identification of the site of labelling on Cys481.

Next, we selected the marketed covalent BTK inhibitor ibrutinib (**11**) as a benchmark scaffold and synthesized its carbonyl and thiocarbonyl α-haloacetamide derivatives (**12**-**15**) corresponding to the electrophiles of fragments **3**-**6** (Fig. 5). Briefly, the amine precursor of the modified TCI (**16**) was *N*-acylated with chloroacetyl chloride (**2**) as depicted in Fig. 1a, to afford the advanced chloroacetamide derivative (**12**). Compound **12** was subsequently converted into fluoroacetamide (**13**) by fluorination with caesium fluoride following the previously shown protocol (Fig. 1b). O-to-S exchange was achieved on both α-haloacetamides (**12,13**) according to the method described above (Fig. 1c) using phosphorus pentasulfide in tetrahydrofuran to get α-chlorothioacetamide (**14**) or α-fluorothioacetamide (**15**), respectively. Ibrutinib analogues were evaluated in a BTK-specific kinase activity assay that recapitulated the activity trends expected based on warhead reactivities and results from covalent fragment-level experiments (Fig. 5, Supplementary Fig. S6). In detail, a 5 min preincubation with **14** (α-chlorothioacetamide) resulted in the highest potency (IC_50_ = 1.0 ± 0.2 nM), surpassing the parent drug **11** (IC_50_= 2.8 ± 0.7 nM) and outperforming its chloroacetamide analogue **12** (IC_50_ = 2.2 ± 0.4 nM). Likewise, the fluorothioacetamide analogue **15** (IC_50_ = 4.5 ± 1.1 nM) exhibited improved potency compared to the weakly reactive α-fluoroacetamide **13** (IC_50_ = 20.0 ± 6.1 nM). Binding kinetics play a crucial role in the design of covalent inhibitors^70,71^, as the efficacy of TCIs is not governed solely by equilibrium affinity but by a two-step kinetic mechanism: noncovalent binding (characterized by K_I_) followed by covalent bond formation (characterized by k_inact_). Factors such as the polarity and geometry of the binding pocket, steric constraints, nucleophilicity and accessibility of the targeted residue, as well as its conformational flexibility, directly modulate k_inact_ and K_I_, thereby determining both selectivity and efficacy. Thus, rational optimization of covalent drugs requires a kinetic perspective, ensuring that the interplay of noncovalent recognition and chemical reactivity enables productive, selective, and physiologically relevant covalent modulation of the target. To better understand the binding mechanism and kinetics of the investigated ibrutinib-analogous (**11-15**), we determined k_inact_/K_I_ parameters for each probe (Fig. 5). Ibrutinib (**11**) displayed a k_inact_ of 7.33 × 10^−^^3 ^s⁻¹ and K_I_ of 3.5 × 10^−9 ^M, corresponding to a k_inact_/K_I_ of 2.1 × 10^6 ^M⁻¹s⁻¹, in line with its well-characterized covalent inhibition profile. The α-chloroacetamide analogue **12** showed increased covalent bond formation rate (k_inact_ = 3.0 × 10^−2 ^s^−^¹), leading to a similar (yet slightly lower) overall efficiency (k_inact_/K_I_ = 9.2 × 10^5 ^M⁻¹s⁻¹). The α-fluoroacetamide derivative **13**, which is nearly inactive in covalent mode under normal conditions, retained very low covalent turnover (k_inact_ = 2.7 × 10^−4 ^s⁻¹), as expected. On the contrary, O-to-S exchange dramatically boosted the inactivation rates of both analogues: the α-chlorothioacetamide **14** reached a k_inact_ of 8.3 × 10^-1 ^s⁻¹, yielding the highest efficiency (k_inact_/K_I_ = 2.5 × 10^6 ^M⁻¹s⁻¹) among the series despite its slightly reduced binding affinity (K_I_ = 3.3 × 10^−7 ^µM). Similarly, the α-fluorothioacetamide **15** showed a > 200-fold increase in k_inact_ (6.7 × 10^−2 ^s⁻¹) compared to its oxygen analogue **13**, and resulting in an improved efficiency (k_inact_/K_I_ = 6.8 × 10^5 ^M⁻¹s⁻¹). Taken together, these results indicate that the O-to-S exchange enhanced the potency of both the chloro- and fluoroacetamide analogues. Finally, the target occupancy of the ibrutinib analogues (**12**-**15**) was evaluated by intact MS, which confirmed covalent labelling of BTK with efficacy mostly proportional to the reactivity of the warheads (Supplementary Fig. S3 and Table S6). However, we note that the potency of covalent probes is not necessarily correlated with the MS labelling efficiency, as reported recently^72^. Moreover, specific labelling of Cys481 with acrylamide **11**,α-chloroacetamide **12** and α-chlorothioacetamide **14** probes was confirmed via tryptic digestion followed by MS proteomics analysis (Fig. 6, Supplementary Fig. S4 and Table S7). Moreover, quantification of the iodoacetamide (CAM) labelled Cys481 showed in all cases significant decrease of its averaged MS2 intensities related to the Cys481-containing Glu467-Arg486 compared to the DMSO-treated mock samples (Fig. 6a). These results suggest that changing the warhead chemotype did not bias the availability of the inherently targeted cysteine significantly, but the degree of labelling is strongly influenced by the intrinsic reactivity of the warhead attached to the non-covalent scaffold, as expected. To complement these experimental data, we analyzed the labelling reactions in the real protein environment by QM/MM simulations. In addition, molecular dynamics simulations were used to assess the dynamic behaviour of the compounds in the binding pocket. Analysis of three characteristic distances confirmed that probes **12** and **14** adopt highly similar binding modes, with identical key interactions involving Cys481, Glu475, and Phe570. The MD trajectories (Supplementary Fig. S7a), together with the tryptic digestion results, indicate that sulfurization does not alter the binding mode of the covalent inhibitors.

We next quantified binding and covalent reactivity of probes **12** and **14** using QM/MM free-energy methods. Specifically, we applied umbrella sampling combined with thermodynamic integration, a methodology we have previously validated for covalent inhibitor design^73^. This approach calculates both the relative binding free energies (ΔΔG_b_) of the noncovalent complexes and the free-energy barriers (G^‡^) of the covalent reaction. Importantly, ΔΔG_b_ can be directly related to differences in dissociation constants (K_I_), while G^‡^ can be converted into k_inact_ values, providing a direct link between calculated parameters and experimentally measured inhibition kinetics. This methodology has been widely used to provide valuable insights into covalent probe design across a variety of protein targets^74–76^. Comparison of the α-chloroacetamide (**12**) and α-chlorothioacetamide (**14**) analogues showed consistent trends between experiments and computations (Supplementary Fig. S7 and Table S8). Both experimental assays and QM/MM calculations indicated that the thioamide-derived warhead reduces noncovalent affinity relative to the oxygen analogue, reflected by higher K_I_ values. At the same time, the sulfur substitution dramatically lowered the reaction barrier, resulting in a substantially higher k_inact_. This kinetic acceleration overcompensates for the reduced binding affinity, yielding an overall increase in covalent efficiency (k_inact_/K_I_). Together, these results show that QM/MM simulations can support the conclusion that O-to-S exchange can be an effective strategy to increase the covalent inhibitory potential of BTK-targeting warheads. Furthermore, the simulation methodology might be useful to evaluate the impact of O-to-S exchange on the protein of interest.

**Fig. 7:**
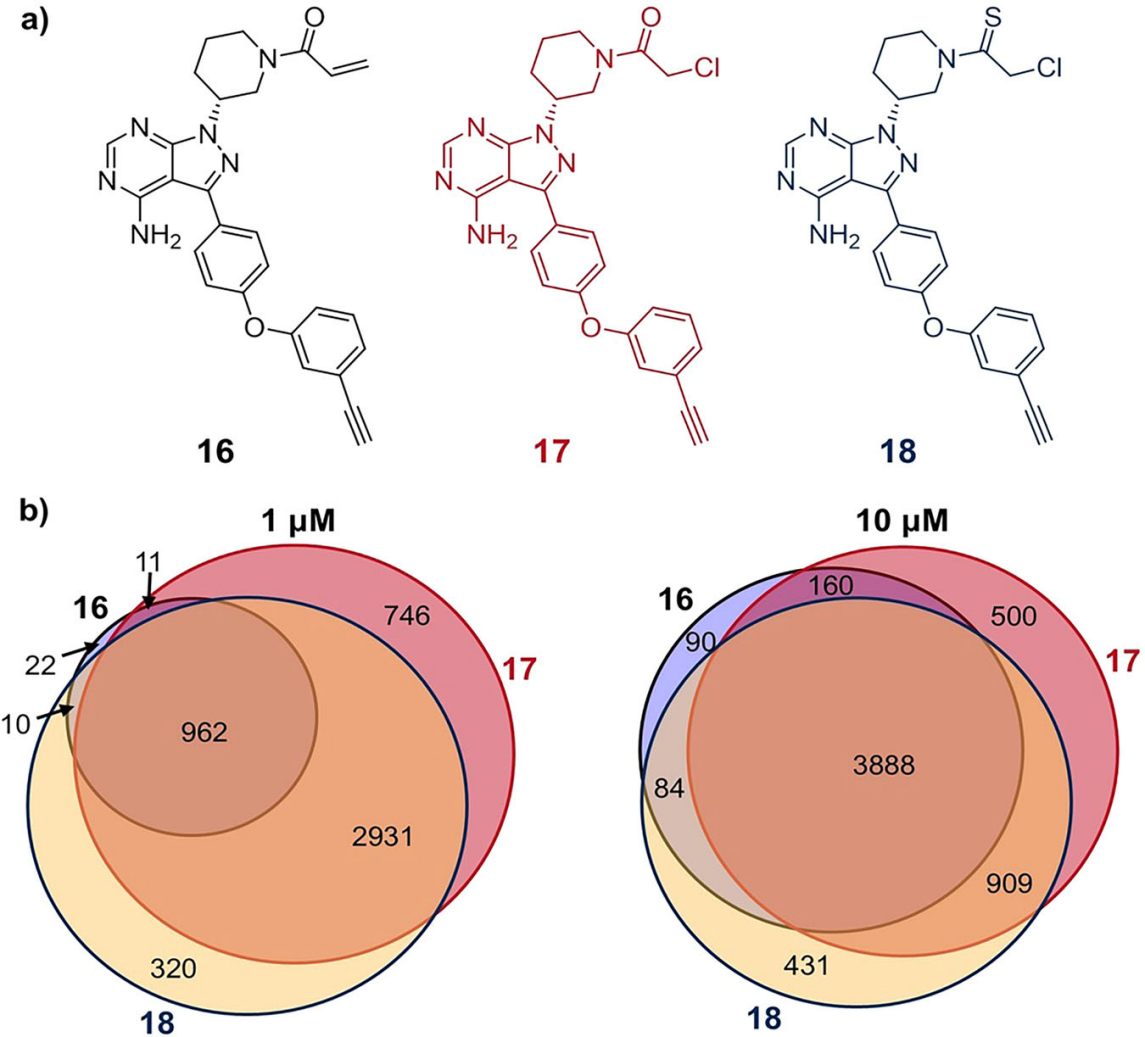
Proteome-wide target-scope analysis of ibrutinib-derivatives (16−18). **a** Chemical structure of the covalent probes (**16**−**18**) used for chemoproteomic pull-down experiments and **b** the proportional Venn diagrams measured after treatment of Mino cell lysates with 1 or 10 μM alkyne probes (**16-18**).

Finally, to assess whether the increased intrinsic reactivity of α-halothioamide warheads translates into more pronounced cellular engagement, we synthesized alkyne-tagged analogues of **11,** **12**, and **14** (resulting in probes **16-18**, Fig. 7) and performed chemoproteomic profiling in Mino cell lysates. In all cases, BTK was consistently among the top-enriched targets, confirming effective on-target engagement. At 10 µM probe concentration, the parent ibrutinib analogue **16** labelled 4222 proteins, while the chloroacetamide **17** showed the highest degree of promiscuity with 5457 proteins. The α-chlorothioacetamide **18**, in turn, engaged 5312 proteins, representing a modest reduction compared to **17**. Intersections revealed 3888 proteins captured by all three probes and 909 shared exclusively between the two α-chloroacetamides (**17** and **18**) (Fig. 7b). Notably, while lowering the probe concentration to 1 µM drastically reduced the off-target hits of **16**, the numbers for the **17** and **18** chloroacetamides were not significantly affected. Taken together, these results demonstrate that O-to-S exchange does not exacerbate proteome-wide promiscuity. Despite the experimentally and computationally confirmed increase in warhead reactivity, the off-target scope of **18** chlorothioacetamide was in fact slightly better than the α-chloroacetamide derivative (**17**).

To further evaluate the cellular relevance of the ibrutinib-derived covalent inhibitors with the thioacetamide-derived warheads, we performed MTT assay with Ramos cells to determine in vitro cellular IC_50_ values (Fig. 5 and Supplementary Fig. S8). The chlorothioacetamide derivative (**13**) exhibited comparable cellular potency to its parent chloroacetamide (**12**, IC_50_ = 0.21 ± 0.09 µM vs. 0.14 ± 0.04 µM, respectively) and significantly higher potency than ibrutinib (**11**, IC_50_ = 0.94 ± 0.27 µM)^68^. In contrast, the fluoroacetamide analogue (**14**) showed a marked loss of activity (30.72 ± 9.86 µM), consistent with its lower intrinsic reactivity. Importantly, conversion to the fluorothioacetamide (**15**) restored potency to IC_50_ = 1.75 ± 0.81 µM, bringing it into a similar range of cellular potency as the parent drug. Finally, considering the potential application of these thioamide-derived warheads in TCIs, we performed in vitro human liver microsome metabolism experiments and assessed the remaining compounds by HPLC measurements (Supplementary Fig. S9). In brief, both the **11** chloroacetamide and **12** chlorothioacetamide derivatives displayed a similar range of metabolic stability, with 1.1–3.7% of the compound remaining after 30 minutes. In contrast, the moderately reactive but proven covalent fluorothioacetamide derivative showed increased stability, with 10.2% remaining after 30 minutes. Taken together, these results suggest that O-to-S exchange may represent a promising late-stage warhead optimization strategy, with the potential to enhance potency while maintaining a favourable off-target profile.

### α-Halothioamide-based antibody conjugation

**Fig. 8:**
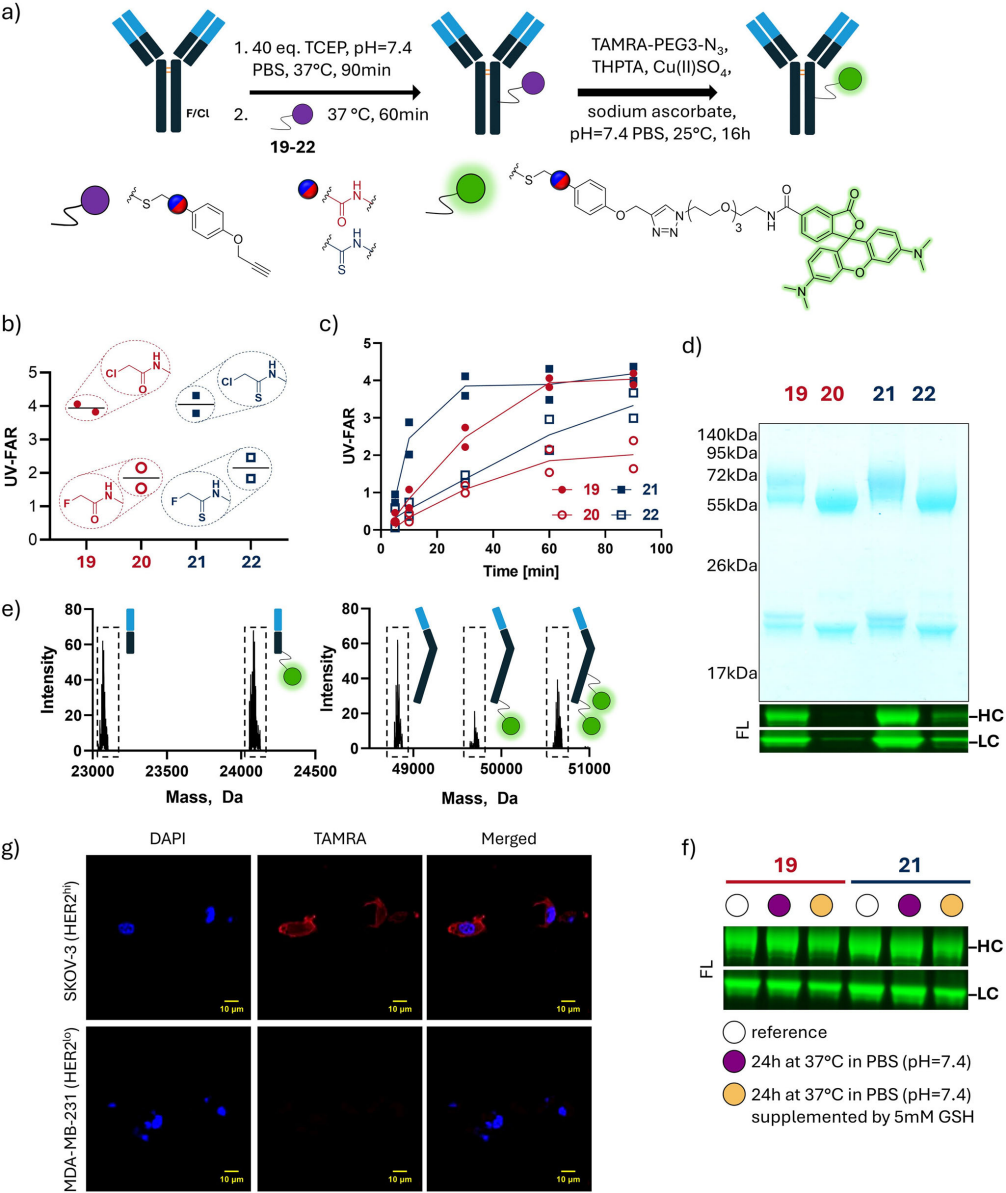
Antibody conjugation with acetamide (19-20) and thioacetamide (21-22) probes. **a** Conjugation of trastuzumab with **19-22** probes. After reduction with TCEP, covalent labelling was followed by the TAMRA-PEG_3_-N_3_ “click” to produce fluorescent dye-antibody conjugates; **b** UV-FAR analysis results showing individual results of biological duplicates, indicating the high labelling efficiency achieved by the α-chloroacetamide **19** and α-chlorothioacetamide **21** probes; **c** time-dependent bioconjugation efficiency was measured as UV-FAR after different time intervals applied in antibody conjugation step, individual datapoints of two independent biological replicates are shown; **d** in-gel visualization of the produced fluorescent dye-antibody conjugates, showing the fluorescence of heavy chain (HC) and light chain (LC) captured by both, fluorescence readout and coomassie brilliant blue staining corresponding to a single independent experiment; **e** covalent modification of the heavy and light chains treated with the chlorothioacetamide probe (**21**) are shown on deconvoluted MS spectrum (*n* = 1); **f** stability measurements on SDS-PAGE of the antibody-TAMRA conjugates in PBS (pH=7.4) and reductive PBS (pH=7.4, 5 mM GSH) with gels shown as representative experiments (*n* = 1); and **g** immunostaining on SKOV−3 HER2^hi^ and MDA-MB−231 HER2^lo^ cell lines applying trastuzumab-TAMRA conjugate produced by the chlorothioacetamide (**21**) probe. The nuclei of the cells are shown in blue, and trastuzumab-TAMRA staining is depicted in red. The immunostaining experiment was performed once; consistent staining was observed across the analyzed slide.

Next, we challenged thioamide-derived warheads in the conjugation of small molecules to antibodies (Fig. 8) and investigated whether they are useful for the synthesis of antibody-drug conjugates (ADCs)^77^. Specifically, we attempted to label trastuzumab, a monoclonal antibody essential in targeting HER2-positive cancer cells^78^ and commonly used as the basis for ADCs. Thus, we synthesized alkyne probes **19-22** (Supplementary Fig. S10) following the previously developed synthetic strategy (Fig. 1). These probes have analogous warhead structures as compared to the **3-6** covalent fragment probes. To achieve antibody functionalization, we applied a sequential labelling strategy, including reduction with TCEP, and then the alkynes (**19-22**) were covalently attached to trastuzumab, allowing subsequent click chemistry for conjugation with TAMRA-PEG_3_-azide (Fig. 8a). The fluorophore-antibody ratio (FAR) was confirmed by dual-wavelength UV-VIS spectroscopy, providing quantitative evidence of successful labelling (Fig. 8b, Supplementary Table S9).

The α-thiochloroacetamide (**21**) and the less reactive α-thiofluoroacetamide probes (**22**) exhibited FAR of 4.05, and 2.15, respectively. A similar trend was observed for the **19** α-chloroacetamide and **20** α-fluoroacetamide carboxamide probes (FAR = 3.94 vs 1.85, respectively). In addition, to gain a deeper understanding of the binding kinetics of antibody modification, we performed time-resolved UV-FAR analyses comparing the conjugation behaviour of haloacetamide and halothioacetamide probes (Fig. 8c, Supplementary Table S9). Antibody labelling was monitored over a 90-minute period at five time points (5, 10, 30, 60 and 90 min). These experiments revealed clear differences in kinetic profiles between the two classes of electrophiles. Although the final UV-FAR values obtained with **19** α-chloroacetamide and **21** α-chlorothioacetamide were similar, the thio-analogue reached maximal labelling within 30 minutes, consistent with its higher intrinsic thiol reactivity. In contrast, **22** α-fluorothioacetamide exhibited a more prolonged labelling process compared to **20** α-fluoroacetamide, which plateaued early at lower FAR values. The successful conjugation with the alkyne probes was further confirmed via fluorescent visualization on SDS-PAGE (Fig. 8d and Supplementary Fig. S11) and by MS (Fig. 8e, Supplementary Fig. S12) on both the heavy and light chains. The stability of the **19** chloroacetamide- and **21** thiochloroacetamide-based conjugates was then evaluated under physiologically relevant conditions at 37 °C in two media: PBS (pH 7.4) and a reductive PBS buffer (pH 7.4, supplemented with 5 mM GSH). In both cases, the conjugates were found to be stable, including those prepared with the thiochloroacetamide warhead (Fig. 8f and Supplementary Fig. S13). Finally, we investigated the **21** chlorothioacetamide-derived antibody-TAMRA fluorophore conjugate by confocal microscopy (Fig. 8g), which confirmed this antibody conjugate retained the ability to selectively label HER2^hi^ SKOV-3 cells over HER2^lo^ MDA-MB-231 cells. These findings underscore the potential of the thioacetamide-derived warheads as antibody conjugation tools in targeted therapy and imaging applications.

### α-Halothioamide-based target discovery

**Fig. 9:**
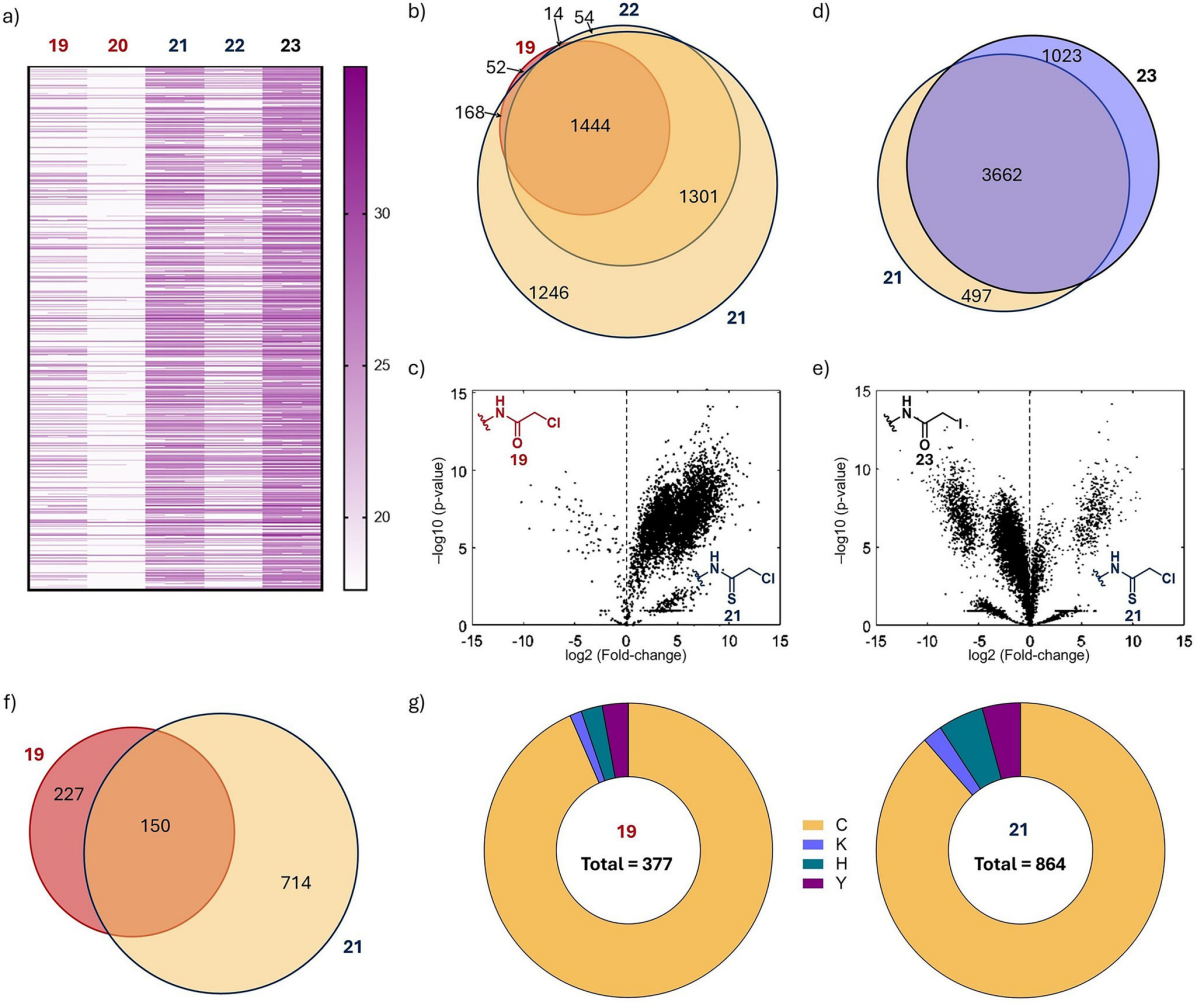
Proteome-Wide Profiling of Covalent Probe Reactivity and Selectivity. **a** Heat-map based on the intensity of MS-labelling for the whole proteome showing increasing labelling efficacy shifting from white (no labelling) into dark purple. Each column shows triplicate results obtained for the 5983 identified proteins. **b** Venn diagram showing the distribution of the number of identified proteins that were labelled by **19, 22 and 23** probes. **c** Volcano plot depiction of oxygen-to-sulfur matched molecular pair (**19** and **21**) representing the high increase of labelling effectiveness achieved by the chlorothioacetamide (**21**) probe. The x-axis represents the log2 fold-change in intensity, and the y-axis represents the statistical significance (-log10 *P* value). Statistical significance was determined using a two-sided Student’s *t*-test. No adjustments were made for multiple comparisons. **d** Venn diagram showing the distribution of identified proteins that were labelled by **21** and **23** probes. **e** Volcano plot depiction of protein labelling effectiveness achieved by the **23** iodoacetamide and the **21** chlorothioacetamide probes showing protein subset exclusively labelled by the **21** probe. The x-axis represents the log2 fold-change in intensity, and the y-axis represents the statistical significance (-log10 *P* value). Statistical significance was determined using a two-sided Student’s *t*-test. No adjustments were made for multiple comparisons. **f** Venn diagram showing the distribution of identified peptides that were labelled by **19** and **21** probes. **g** Proteome-wide residue specificity of chloroacetamide (**19**) and chlorothioacetamide (**21**) probes. Shown are the percentages of cysteine, lysine, histidine, and tyrosine residues identified as probe-modified sites. Probe **19** labelled 377 sites in total (93.3% Cys, 1.3% Lys, 2.4% His, 3.0% Tyr), whereas probe **21** yielded 864 sites (88.4% Cys, 2.2% Lys, 5.1% His, 4.3% Tyr).

Finally, we investigated the activity of α-halothioamide probes in chemoproteomics application for target discovery. Activity-based Protein Profiling (ABPP) is an advanced chemoproteomic analytical technique in chemical biology and drug discovery that is used to identify and validate molecular targets of bioactive small molecules^79–82^. This approach utilizes chemical probes that specifically interact with biological macromolecules such as proteins, nucleic acids, or lipids. Here, we aimed to evaluate the potential of the thioamide-derived warheads for use in chemoproteomics and for improved target discovery. Based on the surrogate-level results, we confirmed that most thioamide-derived warheads exhibit significant thiol reactivity. However, the benzylic moiety provides conformational flexibility, which we assume contributes to the broad coverage of proteome-wide cysteine targets by reducing sensitivity to steric factors in the vicinity of the targeted residues. Therefore, HEK293 lysates were incubated with the *N*-benzylamide-derived alkyne probes (Supplementary Fig. S10) at 10 μM concentration for 1 h, followed by clicking on TAMRA-azide and running on SDS-PAGE gel. In-gel proteomics experiments showed significant labelling potential for probes **19,** **21** and **22** (Supplementary Fig. S14). The labelling efficiency of the **21** chloro- and the **22** fluorothioacetamide probes was comparable to that of the iodoacetamide (**23**) probe, labelling the HEK293 lysates in specific chemoproteomic assay conditions, which is designed to probe intrinsic cysteine reactivity rather than evaluate target-selective behaviour in living cells. Next, pull-down, followed by MS proteomics experiments, demonstrated that α-halothioacetamide probes (**21,22**) are more effective labelling agents compared to their matched amide molecular pairs (Fig. 9a–c). In particular, α-chlorothioacetamide probe **21** labels a much higher number of proteins compared to its chloroacetamide analog (**19**, Fig. 9a–c), and also the α-fluorothioacetamide **21** labels more proteins compared to its α-fluoroacetamide pair (**20**, Fig. 9a, b). Overall, the iodoacetamide alkyne (**23**) has the highest proteomic reactivity, followed by **21** α-thiochloroacetamide, **22** α-thiofluoroacetamide, **19** α-chloroacetamide and **20** α-fluoroacetamide, respectively (Fig. 9a). Reduced electrophilicity, however, does not necessarily translate into proportionally reduced chemoproteomic labelling, as local cysteine microenvironments and experimental conditions can substantially modulate apparent proteome-wide engagement, thereby explaining the observed differences in labelling promiscuity across the chemoproteomic probes in the set. Although the iodoacetamide alkyne (**23**) labels somewhat more proteins in general (Fig. 9d, e), the probe **21** labelled a complementary subset of proteins, as shown in the Venn diagram and volcano plots depicted in Fig. 9d, e. This pattern likely reflects differences in the distinct reactivity of the **21** α-chlorothioacetamide warhead, which result in complementary selectivity profile relative to the other probes. This unique subset was further analyzed to show the functional clusters of proteins exclusively identified by α-thiochloroacetamide (**21**). We found that the most prominent subclasses of the proteome resulting in more than 10 exclusively labelled proteins were within the small GTPase family, the nucleotide-binding α-β plait domain superfamily, the peptidase C14 family and the tetratricopeptide-like helical domain superfamily (Supplementary Fig. S15). Next, we carefully evaluated the proteins uniquely identified with probe **21** and we focused on PDE6δ as a particularly alluring target. PDE6δ plays a key role in KRAS biology by stabilizing and shuttling Cys-farnesylated KRAS through the cytosol until its integration into the plasma membrane^83^. Although a number of nanomolar non-covalent PDE6δ inhibitors were reported^84^, these molecules were displaced by GTP-bound Arl2. Therefore, covalent targeting of PDE6δ would result in novel inhibitors with high cellular potency. This approach has been recently validated by Halo-tag inspired covalent inhibitors labelling Glu88^85,86^. However, a cysteine-targeting approach would represent a more advantageous line to follow due to its superior nucleophilicity and lower proteome-wide abundance. Notably, PDE6δ contains two cysteines (Cys56 and Cys86) residues close to the farnesyl-binding pocket (based on the PDB structure 5TB5^87^), yet no Cys-targeting covalent binders of PDE6δ have been reported in chemoproteomic target identification campaigns. This rendered PDE6δ an especially attractive candidate for validation. To this end, we investigated recombinant PDE6δ to confirm covalent labelling by probe **21** via intact protein MS analysis (Supplementary Fig. S3 and Table S6). Furthermore, subsequent enzymatic digestion with Glu-C and MS/MS analysis identified Cys56 as the covalent binding site of the α-chlorothioacetamide (**21**) probe (Supplementary Fig. S4). While full biological follow-up is beyond the scope of this study, we also sought to assess whether covalent labelling had a functional impact on PDE6δ. Thus, we applied a fluorescence polarization assay widely used to evaluate PDE6δ inhibitors, based on the displacement of TAMRA-atorvastatin (**24**) tracer (Supplementary Fig. S16)^88–90^. We confirmed tracer binding with a K_d_ of 154 nM and determined that probe **21** competitively displaced the tracer with an IC_50_ of 54.4 μM (Supplementary Fig. S16). Altogether, these experiments provide strong orthogonal validation that probe **21** covalently modifies PDE6δ and functionally interferes with ligand binding. Finally, to assess the proteome-wide residue specificity of the thiowarheads described herein, we conducted targeted ABPP experiments to identify labelling sites across the whole proteome. For technical reasons, at the peptide level, there were fewer identifications of modified peptides than at the protein level. Still, comparative analysis (Fig. 9f, g). revealed that the α-chloroacetamide alkyne probe (**19**) modified 377 amino acid residues, with a strong cysteine preference (93.3%), while the α-chlorothioacetamide alkyne probe (**21**) yielded a substantially larger number of modifications (864 sites), including 764 cysteines (88.4%) alongside minor labelling of other residues (lysine, histidine, tyrosine). These results demonstrate that, while both electrophiles retain a strong bias toward cysteine, the α-halothioamide probe exhibits broader reactivity across the whole proteome. These findings highlight the ability of thioacetamide-derived probes to uncover novel and biologically relevant covalent targets. Thus, ABPP-probes with thioamide-derived warheads could expand chemoproteomic coverage and facilitate advanced target discovery.

Overall, our results demonstrate that oxygen-to-sulfur (O-to-S) exchange provides a versatile electrophile tailoring strategy for late-stage optimizations, leading to the discovery of α-chlorothioacetamides and α-fluorothioacetamides as electrophilic warhead chemotypes. Initial fragment library screens for thiol reactivity and selectivity revealed that O-to-S exchange increases thiol-reactivity, while maintaining amino acid selectivity and aqueous stability. Our findings were further validated at the protein level by screening the covalent fragment probes against covalently relevant and tractable kinase targets. These experiments confirmed the utility of the O-to-S exchange strategy, demonstrating that it could transform even a nonreactive electrophilic moiety, such as fluoroacetamide, into a reactive covalent warhead like fluorothioacetamide. Next, the O-to-S exchange strategy was applied to design targeted covalent inhibitors. As a representative example, we synthesized potent and selective JAK3 inhibitors and characterized them in MS-based and biochemical experiments. In addition, ibrutinib derivatives were obtained and we confirmed their inhibitory activity and their ability to covalently label their BTK target. We thoroughly analyzed and characterized the kinetics of those covalent probes and confirmed their cellular efficacy on Ramos cells. Proteome-wide profiling revealed that α-halothioamide probes do not increase cellular promiscuity as compared to their α-haloacetamide analogues, revealing their application as covalent tools for TCI development. To extend this strategy to bioconjugation, we demonstrated efficient antibody conjugation by linking TAMRA to trastuzumab, highlighting the potential of thioacetamides for ADC synthesis. Finally, the corresponding alkyne-tagged α-halothioamide probes were evaluated by pull-down chemoproteomics, confirming robust proteome-wide target engagement, excellent cysteine selectivity and labelling of a distinct protein subset compared to the conventional iodoacetamide probe. Notably, this led to the finding of Cys-targeting covalent PDE6δ labelling with functional consequence. Altogether, our results establish O-to-S exchange as a feasible strategy to fine-tune the reactivity of amide warheads. Beyond the case studies shown here, this approach and the warheads described herein provide a valuable resource for advancing covalent probe development in medicinal chemistry and chemical biology applications.

## Methods

### General procedures and instruments

All starting materials and solvents used for chemical synthesis were obtained from commercial vendors (Sigma-Aldrich, Fluorochem, and Combi-Blocks) and were used without additional treatment or purification. Reaction progress was monitored by thin-layer chromatography (TLC) on silica gel 60 F_254_ TLC plates (Merck, Darmstadt, Germany). Column chromatography purifications were carried out using Teledyne ISCO CombiFlash Lumen+ Rf system. ^1^H NMR spectra were recorded at room temperature on a Varian Unity Inova 500 spectrometer (500 MHz for ^1^H NMR spectra). The deuterated solvent signal was used for field locking. ^1^H and ^13^C NMR spectra were processed using MestReNova 6.0.2 with automatic phase and baseline corrections applied. Chemical shifts (δ) are reported in ppm relative to residual solvent signals, and coupling constants (J) are given in Hz. HPLC-MS analyses were performed on a Shimadzu LCMS-2020 system equipped with a Reprospher 100 C18 (5 µm; 100 x 3mm) column and dual ion source (DUIS±) coupled to a quadrupole MS analyzer operating in a range of 50–1000 m/z. Samples were separated using gradient elution with mobile phase A (0.1 V/V% formic acid in water) and mobile phase B (0.1 V/V% formic acid in acetonitrile). Flow rate was set to 1 ml/min. The gradient programme started at 5% B, followed by a linear increase to 95% B within 2 min, from 2 to 4 min 95% B was retained; then returned to the initial conditions (5% B) and held until 5 min. The column temperature was maintained at room temperature and the injection volume was 10 µl. Compound purity was evaluated by HPLC with UV detection; all tested compounds showed purity above 95%. High-resolution mass spectrometric measurements were performed using a Q-TOF Premier mass spectrometer (Milford, MA) operated in positive or negative electrospray ionization mode. Data acquisition and processing were performed using Analyst software version 1.6.2 (AB Sciex Instruments, CA, USA). Chromatographic separation was achieved using a Purospher STAR RP-18 endcapped (50 mm × 2,1 mm, 3 µm) LiChocart ® 55-2 HPLC Cartridge. The sample was eluted with gradient elution using solvent A (0.1 V/V% formic acid in water) and solvent B (0.1 V/V% formic acid in acetonitrile) at a flow rate of 0.5 ml/min. The initial condition was 5% B for 2 min, followed by a linear gradient to 95% B by 6 min, from 6 to 8 min 95% B was retained; and from 8 to 8.5 min back to the initial condition with 5% eluent B and retained to 14.5 min. The column temperature was kept at room temperature, and the injection volume was 10 µl. Nitrogen was used as the nebulizer gas (GS1), heater gas (GS2), and curtain gas with the optimum values of 35, 45 and 45 (arbitrary units), respectively. The source temperature was set to 450 °C and the ion spray voltage was 5000 V. Declustering potential value was adjusted to 150 V.

### Thiol reactivity assay

For the l-glutathione (GSH) reactivity assay, 500 μM solution of the fragment (in PBS, pH 7.4, 10% acetonitrile, 250 μL) supplemented with 200 μM indoprofen as an internal standard was mixed with a 10 mM l-glutathione solution (dissolved in PBS, pH 7.4, 250 μL) in 1:1 ratio. The final assay mixture (500 μL) contained 250 μM fragment, 100 μM indoprofen, 5 mM l-glutathione and 5% acetonitrile. Samples were prepared in biological duplicates and analyzed by HPLC-MS at 0, 1, 2, 4, 8, 12, 24, 48, 72 h time points. Compounds aqueous stability (degradation kinetics) was also examined using the same experimental setup, but with PBS buffer replacing the l-glutathione solution. In this case, the final mixture (500 μL) contained 250 μM fragment, 100 μM indoprofen and 5% acetonitrile. Areas under the curve (AUC) were obtained by integration of HPLC chromatograms and subsequently normalized using the internal standard. The corrected AUC values were then used for ordinary least squares (OLS) linear regression. The kinetic parameters, including the kinetic rate constant (k) and half-life (t_1/2_) were calculated using a programmed Microsoft Excel sheet with Visual Basic for Applications (VBA). The reported data represent a mean of duplicate measurements, with standard errors always within 10% of the calculated values. The kinetic rate constants for degradation and thiol reactivity were determined as follows. For pseudo-first order reactions, the half-life is defined as t_1/2_ = ln2/k, where k is the reaction rate constant. In the case of competing reactions (reaction with glutathione and water), the effective rate constant for the disappearance of the starting compound is given as a sum of the individual rate constants: k_eff_ = k_deg_ + k_GSH_. Accodringly, the experimentally measured half-life correspond to t_1/2(eff)_ = ln2/(k_eff_) = ln2/(k_deg_ + k_GSH_). In this study, the corrected k_deg_ and k_eff_ values (derived from blank and GSH-containing samples, respectively) were obtained by linear regression of the kinetic data points. The corrected k_GSH_ value was calculated as k_eff_–k_deg_, and finally, the half-life associated with thiol rectivity was finally determined by applying t_1/2(GSH)_ = ln2/k_GSH_.

### Oligopeptide selectivity assay

For the selectivity assay, 2 mM solution of the fragment (prepared in PBS pH 7.4 containing 20 % acetonitrile) was mixed in 1:1 ratio with 200 μM solution of the nonapeptide (KGDYHFPIC in PBS buffer pH 7.4). The final reaction mixture contained 1 mM fragment, 100 μM nonapeptide and 10 % acetonitrile. Samples were incubated at room temperature overnight. The incubation time was determined based on the GSH reactivity of the fragments and was set to either 16 h or 24 h (fragments with a GSH half-life under 12 h were incubated for 16 h, the others for 24 h). Fragment binding selectivity was analyzed using an Information Dependent Acquisition (IDA) LC-MS/MS experiment to determine whether covalent modification occurred specifically at thiol residues. An enhanced MS scan was used as the survey scan, followed by enhanced product ion (EPI) scans as dependent experiments. In the EPI experiments, the collision energy in EPI experiments was set to 30 eV with a collision energy spread (CES) of 10 V. The site of covalent attachment was determined using GPMAW 4.2 software. Relative quantitation of the nonapeptide – fragment conjugates was calculated from the total ion chromatograms.

### DFT calculations

DFT methods were used to calculate activation energy barriers (ΔG^‡^) against the methylthiolate anion (MeS^−^) as a theoretical cysteine surrogate. We used the Gaussian 09 software package^91^ with the SMD implicit solvation model (water) and the M062X functional^92^, as it has been shown as one of the most accurate functionals to calculate these parameters^56,57^. We performed geometry optimizations with the 6-311 G + (d,p) basis set to obtain Gibbs free energies (G). Next, QST3 transition state optimization was applied (in the case of Michael acceptors, we always considered the s-cis transitional geometry^60^) to determine transitional state geometry, and subsequently Gibbs free energies were calculated as described for initial state calculations at standard conditions. Finally, the activation energy barriers (ΔG^‡^) were determined as Gibbs free energy differences of the optimized transition and the initial compounds.

### **T**hermodynamic Integration and QM/MM calculations

#### Structure preparations for thermodynamic integration

The structure for BTK-Ibrutinib complex (PDB: 5P9J^93^) was used as a starting structure to build the complexes of compound **12** (O) and **14** (S). The preparation was performed with Schrödinger’s Protein Preparation Workflow [Schrödinger Release 2024-3:, Schrödinger, LLC, New York, NY, 2024.] using default settings that include H-bond assignment optimization with PROPKA^94^. The covalent bond between Cys481 and the ligand was deleted, and the ligand warhead was modified. Cys481 was set either to thiol or to thiolate as both forms are present owing to its low pK_a_ of 7.7^95^. Restrained minimization was performed to alleviate steric clashes.

#### Thermodynamic integration

Thermodynamic integration was carried out by the Amber software package (Amber 2024, University of California, San Francisco, CA, USA). Both the complexes and the ligands were immersed in an octahedral water box and neutralized by sodium ions. The systems were subjected to the relaxation protocol with 1000 steps of steepest descent minimization, 20 ps NVT heating to 300 K and 20 ps of NPT equilibration with the coupling parameter value set to 0.5. The alchemical transformations involved 3 steps: The removal of the softcore atom’s partial charges, the transformation of the uncharged softcore atom into the perturbed structure (VdW step), and the reintroduction of the partial charge for the perturbed structure (Fig. S7). Every simulation involved MD runs with lambda coupling parameter values set from 0.0 to 1.0 with 0.1 increment. Every lambda ”window” contained a 20 ps long heating to 300 K in the NVT ensemble and a 200 ps long thermodynamic integration, also in the NVT ensemble with 1 fs timestep. SHAKE^96^ was enabled for the non-TI region atoms, and NOSHAKE was applied for the softcore hydrogens. TI was performed in triplicate both for the thiol and thiolate forms of Cys481. Results are shown in Table SX1.

#### Structure preparations for QM/MM calculations

The covalent enzyme-inhibitor complexes were generated by mutating the molecule covalently bound to the active site cysteine in the PDB: 5P9J structure. The covalent bond between the sulfur and carbon atom was broken and the ligand warhead was modified. The resulting enzyme-ligand complexes were immersed in an octahedral water box of TIP3P waters^97^ and neutralized by adding sodium ions. The QM region consisted of the active site cysteine residue cut between C_α_ and C_β_ and also the warheads of the ligands. All the other atoms were part of the MM region (Fig. S7). The dangling bonds of the QM region were filled with hydrogens (link atom approach). The systems were subjected to the relaxation protocol with 1000 steps of steepest descent minimization, 20 ps NVT heating to 300 K and 2 ns of NPT equilibration. The SHAKE algorithm was used to constrain hydrogens. The difference between the C-Cl and S-C bond distances was used as a reaction coordinate (Fig. S7).

#### PMF calculations with QM/MM MD simulations

Simulation of the covalent reaction step was performed by molecular dynamics combined with umbrella sampling^98^ (US) using the AMBERtools24 software package (Amber 2024, University of California, San Francisco, CA, USA). The QM part was treated with DFTB3 potential^99^, while FF14SB^100^ and GAFF^101^ force fields were applied for the MM region. Starting geometries for the simulation windows were generated by steered molecular dynamics simulations^102^ performed in the NVT ensemble at 300 K. The middle of the biasing potential was pulled along the reaction coordinate between −2.0 and 0.7 Å by the velocity of 1 Å/ps and a force constant of 250 kcal/(mol·Å^2^). Frames with 0.1 ps intervals were extracted (see Supplementary Data 1) and used as starting structures for the US simulation windows. QM/MM MD US simulations were performed in NVT ensemble at 300 K with a 250 kcal/(mol·Å^2^) force constant applied for the reaction coordinate. 30 ps simulation with 1 fs time step was performed for each of the 28 windows and the last 20 ps was used in the generation of the PMF by the weighted histogram analysis method (WHAM)^103^. The statistical uncertainties were calculated by WHAM’s bootstrapping algorithm using ten fake data sets coupled with a 140-step correlation time derived from the autocorrelation function of several data files of the Umbrella Sampling simulations. Approximate transition states were identified as maxima on the free energy curves. PMF profiles are shown in Fig. S7.

### Z'-LYTE kinase inhibition assay of JAK3, BTK and MAP2K6

Fragments **3a-b,** **4a-b,** **5a-b** and **6a-b** were evaluated at a concentration of 100 μM, using the Z′-LYTE kinase inhibition assay (Life Technologies). Measurements were performed in duplicates on a selected kinase subpanel consisting of BTK, JAK3 and MAP2K6. The Bruton’s tyrosine kinase (BTK) and the Janus kinase 3 (JAK3) were selected from the TK (tyrosine kinase) branch and the mitogen-activated protein kinase MAP2K6 from the STE (yeast sterile 7-,11- and 20-homologue kinases) branch. The Z’-LYTE assay is a fluorescence-based method, that relies on the different sensitivity of phosphorylated and non-phosphorylated peptides to proteolytic cleavage. In this system, the suitable peptide substrate is labelled with two fluorophores, forming a FRET pair. Tested compounds were first preincubated with the kinase for 1 h, after which the peptide substrate was added and further incubated the kinase + peptide + test compound mixture for an additional hour. Peptide molecules that were not phosphorylated by the kinase are subsequently cleaved, which disrupts the fluorescence resonance energy transfer between the FRET pairs. The reaction outcome is determined by measuring the ratio of fluorescence emission at 445 nm (coumarin) and 520 nm (fluorescein), corresponding to the relative amounts of cleaved and intact peptides. More detailed description of the assay is available in the manufacturer’s documentation (https://www.thermofisher.com/TFS-Assets/LSG/brochures/Z-LYTE_Brochure_0805.PD).

### Kinase activity assay of Janus kinase 3 (JAK3)

The JAK3 kinase activity assay was performed using the HitHunter® Kinase Enzyme Activity Assay Kit (DiscoverX) protocol with modifications to accommodate the biochemical properties of JAK3. Active recombinant human JAK3 kinase domain (MyBioSource, Cat. No. MBS2097317) was used as the enzyme source. In brief, 5 µL of a 4× inhibitor master mix prepared in ADP Hunter Plus Assay Buffer was dispensed into each well, followed by 5 µL of a 4× kinase master mix containing JAK3 (final concentration 0.05 µg/mL). Inhibitor-kinase mixtures were preincubated for 5 min at 20 °C. Reactions were initiated by the addition of 10 µL of a 2× substrate/ATP master mix consisting of peptide substrate (final concentration 1.25 mM) and ATP (final concentration 100 µM, corresponding to ~25 × K_m_)^104^. Plates were sealed and incubated for 30 min at 30 °C. For detection, 10 µL of Reagent A and 20 µL of Reagent B were sequentially added, and plates were incubated for 30 min at room temperature in the dark. The addition of a stop solution was used for endpoint stabilization. The fluorescence intensity was then measured at λ_ex_ 530 nm/λ_em_ 590 nm. Each sample was prepared in biological duplicates and finally, the data were processed and analyzed using GraphPad Prism 8.0.1 software. Results are given as mean ± SE calculated for the parallel.

### Kinase activity assay of Bruton’s Tyrosine Kinase (BTK)

The assay was performed according to the user manual instructions of the HitHunter® Kinase Enzyme Activity Assay Kit (DiscoverX). In brief, 5 µL of a 4× inhibitor master mix prepared in ADP Hunter Plus Assay Buffer was added to each well, followed by 5 µL of a 4× BTK kinase master mix. Prior to substrate addition, inhibitor–kinase mixtures were preincubated for 1, 2, 5 or 15 min at 20 °C, or applied directly without preincubation. Reactions were initiated by the addition of 10 µL of a 2× substrate/ATP master mix, and plates were sealed and incubated for 60 min at 30 °C. For detection, 10 µL of Reagent A and subsequently 20 µL of Reagent B were added to each well, and plates were incubated for 30 min at room temperature in the dark. The addition of a stop solution was used for endpoint stabilization. The fluorescence intensity was then measured at λ_ex_ 530 nm/λ_em_ 590 nm. Each sample was prepared in biological duplicates and finally, the data were processed and analyzed using GraphPad Prism 8.0.1 software. Based on the time-dependent IC_50_s, k_inact_ and K_I_ were fitted by a customized version of the programme by Mader and Keillor [https://github.com/dunadtx/kinact_KI_Calculator]^105^ Results are given as mean ± SE calculated for the parallel.

### Fluorescence polarization assay to determine PDE6δ binders’ affinity

Fluorescence-polarization displacement assays were performed to determine IC₅₀ and apparent Ki values for compounds binding to purified PDE6δ. Assays were carried out essentially as described in the literature^89,106^ with the following specifics: TAMRA-atorvastatin (**24**) was used as the fluorescent tracer, and recombinant human PDE6 δ (Novus Biologicals, NBP1-44480) was used as the protein. Tracer concentration (25 nM) was chosen from a prior K_D_ titration. Assays were assembled in black F-bottom 384-well plates (Greiner, #781900) at a final reaction volume of 30 µL per well. Compounds were prepared as 2-fold serial dilutions in DPBS buffer (no Ca²⁺/Mg²⁺) containing 0.05% CHAPS; without reducing agents in the assay buffer. Plates were preincubated with the covalent probe or DMSO for 15 min at 25 °C, followed by 60 min incubation at 25 °C with the fluorescent tracer (**24**) and then FP was measured on a SpectraMax iD5 plate reader with fluorescence polarization readout (λ_ex_ = 540 nm, λ_em_ = 590 nm) and reported as milli-polarization units (mP). Raw mP values were blank-corrected and processed in GraphPad Prism 8.0.1. Dose–response curves were fit using a four-parameter logistic (variable slope) model to obtain IC_50_ values^100^. All conditions were performed in biological triplicate.

### MTT assay

Ramos cells were cultured in suspension in high-glucose RPMI-1640 medium (4500 mg/l) supplemented with 10% foetal bovine serum (FBS) and 1% antibiotic (AntiAnti) at 37 °C in a 5% CO_2_ incubator. Cells were placed in 96-well cell culture plates at a density of 5000 cells/well and treated with ibrutinib in a 50 µM/2 dilution series for 5 days, with 6 replicate wells per concentration. Then, PrestoBlue reagent (Thermo Fisher Scientific) diluted in 1x PBS was added directly to the cells at a final concentration of 5%, and incubated for 1 hour at 37 °C in a 5% CO_2_ thermostat. After incubation, fluorescence was detected at 560 nm using a PerkinElmer EnSpire multimode plate reader. For Ibrutinib (**11**) reference a total of 5 biological replicates were applied; for other compounds (**12**-**15**), two biological replicates were applied and the results were averaged and reported as mean ± SD.

### In vitro metabolic stability assay in human liver microsome fractions

The metabolic stability of the test compounds was assessed in vitro using liver fractions derived from humans (Discovery). The assay was conducted in a 96-well plate format according to a standardized protocol, utilizing cofactor-supplemented incubation systems to mimic phase I and phase II metabolic pathways. Liver fractions were thawed on ice and diluted in 0.05 M potassium phosphate buffer (pH 7.4) to a final protein concentration of 1.25 mg/mL Test compounds (**11**-**15**) were dissolved in DMSO to create stock solutions of 5 mM. The drug concentration in the final assays was 10 μM. Reaction samples (*n* = 3) containing the diluted liver microsome fractions and test compounds were pre-incubated for 5 minutes at 37 °C while shaking at 450 rpm. The reaction was initiated by adding the 0.05 M NADPH cofactor mixture to the wells (100 µL per well). Aliquots (100 µL) were taken at zero time and at 30 minute time-point and quenched with an equal volume of chilled acetonitrile. The samples were centrifuged at 2600 RCF for 10 minutes to pellet proteins. Supernatants were transferred to LC vials and stored at −20 °C until LC-MS/MS analysis. The concentration of the parent compound remaining in each sample was determined using liquid chromatography coupled with tandem mass spectrometry (LC-MS/MS). The percentage of remaining substrate was calculated relative to the time-zero sample.

#### HPLC-MS analysis

Chromatography measurements were performed using a Shimadzu LCMS-2020 device equipped with a Reprospher 100 C18 (5 µm; 100 x 3 mm) column and positive-negative double ion source (DUIS±) with a quadrupole MS analyzer in a range of 50-1000 m/z. The sample was eluted with gradient elution using eluent A (0.1% formic acid in water) and eluent B (0.1% formic acid in acetonitrile). Flow rate was set to 1 ml/min. The initial condition was 5% B eluent, followed by a linear gradient to 100% B eluent by 4 min, from 4 to 6 min 100% B eluent was retained; and from 6 to 7 min back to the initial condition with 5% B eluent and retained to 8 min. The column temperature was kept at room temperature, and the injection volume was 10 µl. Compounds were assessed by HPLC with UV detection.

### Expression and purification of the human BTK kinase domain

The expression and purification of the human BTK kinase domain (residues 387–659) was performed following a protocol similar to that of described by Bradshaw J. M. et al.^107^ The kinase domain was cloned into pFastBac-1 vector containing an N-terminal 6×-His tag followed by a TEV protease cleavage site. The plasmid was kindly provided by Dr. Ville Paavilainen (University of Helsinki). Recombinant bacilovirus was produced in Sf9 cells and protein expression was subsequently carried out in Tni insect cells by infecting 2 L of cultured cells with virus at a ratio of 1:200 (V/V). Cell growth was allowed to proceed for 3 days after infection. Cells were harvested by centrifugation (800 *g* for 15 minutes), and the resulting pellet was resuspended in 50 mL lysis buffer (10 mM Hepes, pH 7.5, 400 mM NaCl, 1.5 mM DTT) supplemented with 1× protease inhibitor cocktail (Roche). Cell disruption was performed by passing the suspension five times through a cell homogenizer. Cellular debris was removed by centrifugation (30,000 *g* for 30 minutes). The supernatant containing soluble protein was incubated with nickel–nitrilotriacetic acid (Ni-NTA) agarose beads in binding buffer (lysis buffer supplemented with 20 mM imidazole) for 4 hours at 4 °C to allow affinity binding. Next, the beads were washed with binding buffer (four washes of 5 mL each), and the protein was eluted with four 0.5 mL fractions of elution buffer (lysis buffer supplemented with 300 mM imidazole). The eluate was further purified by size-exclusion chromatography using a HiLoad-16/60-Superdex75 (GE Healthcare) column equilibrated with 20 mM Tris, pH 8.0, 50 mM NaCl, 1 mM DTT. The purified protein was then flash-frozen in liquid nitrogen and stored at −80°C.

### LC/MS of labelled Bruton’s tyrosine kinase (BTK)

Covalent probes (**3-6, 12**-**15**) were dissolved to a concentration of 10 mM in DMSO and stored at –80 °C. For conducting the experiment, a solution of 50X concentrated molecule in DMSO was prepared and diluted 2-fold in reaction buffer (HEPES 20 mM pH = 7.5, 50 mm NaCl) immediately prior to the experiment. 1 μl of this solution was mixed with 24 μl of 1 μM His_6_-BTK (thus diluting the compound 50-fold from the original stock). To stop the reaction, 25 μl of 20% acetonitrile + 0.25% trifluoroacetic acid was added to each sample. The concentrations of the molecule, incubation time and incubation temperature varied depending on the molecule type – small fragments (**3a,** **3b,** **5a,** **5b** and **6b**) were incubated at 20 μM, at 4 °C for 20 hours. Molecules derived from ibrutinib (probes **11**-**15**) were incubated at 2 μM on ice for varying time points beginning at 10 seconds. Two injections from each sample (*n* = 2) were performed. The LC/MS runs for His_6_-BTK were performed on a Waters ACQUITY UPLC class H instrument, in positive ion mode using electrospray ionization. UPLC separation used a C4-BEH column (300 Å, 1.7 μm, 21 mm × 100 mm). The column was held at 40 °C and the autosampler at 10 °C. Mobile phase A was 0.1% formic acid in water, and mobile phase B was 0.1% formic acid in acetonitrile. The run flow was 0.4 mL min^−1^. The gradient used was 1% B for 0.2 min, increasing linearly to 95% B for 1.6 min, holding at 85% B for 0.5 min, changing to 1% B in 0.2 min, and holding at 1% for 1 min. The MS data were collected on a Waters SQD2 detector with an m/z range of 2–3071.98 at a range of 600–1900 m/z. The desolvation temperature was 500 °C with a flow rate of 800 L h^−1^. The voltages used were 1.00 kV for the capillary and 24 V for the cone. Raw data were processed using openLYNX and deconvoluted using MaxEnt with a range of 32500: 36500 Da and a resolution of 1 Da/channel.

### LC/MS of labelled Janus kinase 3 (JAK3)

Covalent probes (**9,10**) were prepared as 10 mM stock solutions in DMSO. For the reaction 0.4 μl of this solution was added to 19.6 μl of 15 μM solution of Janus Kinase 3 (MBS2097317, MyBioSource) in 20 mM Tris at pH 8.0 with 150 mM NaCl and 2 mM MnCl_2_. The samples were incubated at 25 °C for 60 min and the reactions were then terminated by adding 2 μl formic acid. The LC/MS measurements were carried out using a Triple TOF 5600+ hybrid Quadrupole-TOF LC/MS/MS system (Sciex, MA, USA) equipped with a DuoSpray IonSource and coupled to a Shimadzu Prominence LC20 UFLC (Shimadzu, Japan) system consisting of binary pump, an autosampler and a thermostated column compartment. Data acquisition and processing were performed using Analyst TF software version 1.7.1 (Sciex Instruments, CA, USA). Chromatographic separation was performed on a Merck BIOshell^TM^ 400 Å Protein C18 (75 mm × 2,1 mm, 3,4 µm, 400 Å) column. Samples were eluted using a gradient of solvent A (0.1 V/V% formic acid in water) and solvent B (0.1 V/V% formic acid in acetonitrile). The gradient programme started at 10% B for 2 min, followed by a linear increase to 90% B by 8 min. The composition was maintained at 90 % B from 10 to 12 min, after which it was returned to the initial conditions (10 % B) between 12 and 12.5 min and held from 12.5 to 15 min. The flow rate was 0.5 ml/min. The column temperature was kept at 50 °C and the injection volume was 5 µL. Nitrogen served as the nebulizer gas (GS1), heater gas (GS2), and curtain gas with optimized values of 40, 45 and 40 (arbitrary units), respectively. Data were collected in positive electrospray mode over a mass range of m/z = 250 to 3000, with an accumulation time 1 s. The source temperature was set to 400 °C, the spray voltage to 5000 V, and the declustering potential to 80 V. Raw ESI spectra were deconvoluted to determine neutral molecular masses using Peak View Software^®^ V.2.2 (Sciex, Redwood City, CA, USA).

### LC/MS of labelled Mitogen-activated protein kinase kinase 6 (MAP2K6)

Covalent probes (**3a, 3b, 5a, 5b, 6b**) were dissolved to a concentration of 50 mM in DMSO and 0.1 μl of this solution was mixed with 9.9 μl of 7 μM MAP2K6 (10422-HNCB, SinoBiological) in PBS pH=7.4, thus diluting the compound 100-fold from the original stock. To stop the reaction, 1 μl formic acid was added to each sample. The samples were incubated at 37 °C for 60 min. The LC/MS runs for MAP2K6 were performed and evaluated via the same instrumentation and methodology as for JAK3.

### LC/MS of labelled phosphodiesterase 6δ (PDE6δ)

Covalent probe **21** was dissolved to a concentration of 50 mM in DMSO and 0.4 μl of this solution was mixed with 19.6 μl of 25 μM PDE6δ (NBP1-44480, SinoBiological) in 50 mM TRIS pH = 8.0, supplemented with 150 mM NaCl and 0.01% CHAPS, thus diluting the compound 50-fold from the original stock. To stop the reaction, 1 μl formic acid was added to each sample. The samples were incubated at 25 °C for 30 min. The LC/MS runs for PDE6δ were performed and evaluated via the same instrumentation and methodology as for JAK3 and MAP2K6.

### Tryptic digestion and MS/MS analysis to determine labelled sites of Janus Kinase 3 (JAK3)

To 50 μL of a 12 μM recombinant human Janus Kinase 3 (JAK3, MyBioSource, San Diego, CA, USA) solution prepared in 20 mM Tris at pH 8.0 with 150 mM NaCl and 2 mM MnCl_2_, 1 μL of the covalent probe from a 100 mM DMSO stock solution was added. The reaction mixture was incubated at room temperature for 120 min. Following the labelling reaction, the sample was subjected directly to enzymatic digestion. For digestion, 40-50 μL of the reaction mixture was combined with 10 μL 0.2% (w/v) RapiGest SF (Waters, Milford, USA) prepared in 50 mM ammonium bicarbonate buffer (pH = 7.8) Subsequently, 3.3 μL of 45 mM DTT in 100 mM ammonium bicarbonate buffer was added to the mixture and incubated at 37 °C for 30 min. After cooling the sample to room temperature, 4.16 μL of 100 mM iodoacetamide in 100 mM ammonium bicarbonate buffer was added and the sample was left in the dark in room temperature for 30 min. The reduced and alkylated protein was then digested with 10 μL (1 mg/mL) trypsin (Sigma, St Louis, MO, USA), corresponding to an enzyme-to-protein ratio of 1:10. Digestion was carried out overnight at 37 °C. To decompose the surfactant, 7 μL of 500 mM formic acid aqueous solution was added to the digest and the mixture was incubated at 37 °C for 45 min. Prior to LC-MS analysis, the acidified sample was centrifuged at 13,000 x *g* for 5 min. LC-MS/MS measurements were performed using a QTRAP 6500 mode (AB Sciex, CA, USA) equipped with a Turbo V electrospray ion source and coupled to a Perkin Elmer Series 200 micro-LC system (Massachusetts, USA). Data acquisition and analysis were carried out using Analyst version 1.6.2 (AB Sciex Instruments, CA, USA). Peptide separation was achieved on a Vydac 218 TP52 Protein & Peptide C18 column (250 mm × 2.1 mm, 5 μm). A gradient of solvent A (0.1 V/V% formic acid in water) and solvent B (0.1 V/V% formic acid in acetonitrile) was used at a flow rate of 0.2 mL min^–1^. The gradient started at 5% B for 7 min, followed by a linear gradient to 90% B by 53 min, The composition was maintained at 90% B from 60 to 63 min, after which the system returned to the initial conditions (5% B) between 64 and 65 min and was held until 70 min. The injection volume was 10 μL. Modified tryptic peptides were identified using an Information Dependent Acquisition (IDA) LC-MS/MS workflow. Enhanced MS scan (EMS) was applied as a survey scan, while enhanced product ion (EPI) scans were acquired as dependent scans. Collision energy in the EPI experiments was applied in rolling collision energy mode, where the actual value was automatically adjusted according to the mass and charge state of the selected ion. Additional IDA criteria included selection of ions above 400 m/z with intensities exceeding 10^6^ counts, and dynamic exclusion of previously selected target ions for 30 s after 2 occurrences. The scan rate in both EMS and EPI modes was 1000 Da/s. Nitrogen was used as the nebulizer gas (GS1), heater gas (GS2), and curtain gas with optimized values of 50, 40 and 40 (arbitrary units), respectively. The ion source temperature was set to 350 °C and the ion spray voltage to 5000 V, while declustering potential was set 150 V. MS/MS spectra were evaluated using GPMAW 4.2. software together with ProteinProspector (http://prospector.ucsf.edu).

### Tryptic digestion and MS/MS analysis to determine labelled sites of Bruton’s Tyrosine Kinase (BTK)

Recombinant BTK was diluted to 5 µM in HEPES 20 mM pH = 7.5, 50 mM NaCl. 99 µl of protein solution was mixed with 1 µl of 2 mM molecule in DMSO, giving 20 µM molecule. With 2 hours at room temperature, all samples were confirmed to be fully covalently modified. The samples (*n* = 4) were then dialyzed against two changes of PBS, followed by two rapid dialysis steps against fresh 100 mM ammonium bicarbonate. Proteins remained modified after this treatment. 30 µl from each sample was mixed with 0.25 µg trypsin and incubated 37 °C overnight. The samples were then reduced by addition of 1 µl of 150 mM DTT (30 minutes at 37 °C), followed by alkylation with 1 µl of fresh 600 mM iodoacetamide (30 minutes at room temperature in the dark). The samples were then mixed with 200 µl of 0.1% trifluoroacetic acid and desalted by Oasis desalting columns (Waters) per the manufacturer's instructions. The samples were dried using SpeedVac and dissolved in 30 µl of 3% acetonitrile + 0.1% formic acid. 1 µl for each sample was injected for each run.

Samples were analyzed using EASY-nLC 1200 nano-flow UPLC system, using a PepMap RSLC C18 column (2 μm particle size, 100 Å pore size, 75 μm diameter × 50 cm length), mounted using an EASY-Spray source onto an Exploris 240 mass spectrometer. uLC/MS-grade solvents were used for all chromatographic steps at 300 nL/min. The mobile phase was: (A) H2O  +  0.1% formic acid and (B) 80% acetonitrile + 0.1% formic acid. Peptides were eluted from the column into the mass spectrometer using the following gradient: 1–100% B in 65 min, 100% B for 20 min, 100 to 1% in 10 min, and finally 1% for 5 min. Ionization was achieved using a 1800 V spray voltage with an ion transfer tube temperature of 275 °C.

Initially, Data were acquired in data-dependent acquisition (DDA) mode. MS1 resolution was set to 120,000 (at 200 m/z), a mass range of 375–1650 m/z, normalized AGC of 300%, and the maximum injection time was set to 150 ms. MS2 resolution was set to 15,000, quadrupole isolation 1.4 m/z, normalized AGC of 50%, automatic maximum injection time, and HCD collision energy at 30%. The run for each molecule + the DMSO-treated sample was analyzed using fragpipe version 23.1, using a database containing the sequence of the BTK kinase domain, the *E. coli* proteome and common contaminants. 1 missed cleavage was allowed, and the following variable modifications were searched: Methionine oxidation (up to 3), N-terminal acetylation (1), carbamidomethylation (up to 2) and modification by the molecule (1). The results from the DDA search were used to prepare a spectral library for the different peptides, and at this point, the samples were injected again (in triplicate), this time in PRM, with the search focusing on carbamidomethyl-modified cysteine-containing peptides; molecule-modified peptides; and several non-cysteine-containing peptides used as controls. In the PRM runs, MS1 resolution was set to 60,000, maximum injection time was set to 20 ms, while for product ion scans, resolution was set to 15,000 and maximum injection time was set to 150 ms. The product ion signals were used to quantify the peptides, and quantification was performed using Skyline.

### Glu-C digestion and MS/MS analysis to determine labelled sites of PDE6δ

Recombinant human PDE6δ (Novus Biologicals, Cat. No. NBP1-44480) was dissolved in 50 mM Tris buffer (pH 8.0) supplemented with 150 mM NaCl and 0.01% CHAPS to a final concentration of 25 μM. Subsequently, 0.5 μL of a 10 mM DMSO stock solution of covalent probe **21** was added, and the reaction mixture was incubated for 30 min at room temperature. Following labelling, 40 μL of the sample (*n* = 1) was mixed with 8 μL 0.2% (w/v) RapiGest SF (Waters, Milford, USA) solution buffered with 50 mM ammonium bicarbonate (pH=7.8). Then 4.8 μL of 45 mM DTT in 100 mM ammonium bicarbonate was added and the mixture was incubated at 37 °C for 30 min. After cooling to room temperature, 5.6 μL of 100 mM iodoacetamide in 100 mM ammonium bicarbonate buffer was added and the sample was kept in the dark at room temperature for 30 min. The reduced and alkylated protein was then digested with 3 μL sequencing grade Glu-C protease (1 mg/mL, Promega Corporation, Madison, USA) and incubated overnight at 37 °C. To degrade the surfactant, 5 μL of formic acid (500 mM) aqueous solution was added to reach a final concentration of approximately 40 mM (pH ≈ 2), followed by incubation at 37 °C for 30 min. The mixture was centrifuged for 5 min at 13 000 x *g* and the transferred into a microvial for LC-MS/MS analysis. Measurements were performed using a Triple TOF 5600+ (Sciex, MA, USA) equipped with a DuoSpray IonSource coupled with a Shimadzu Prominence LC20 UFLC (Shimadzu, Japan) system. Data were acquired and processed using Analyst TF version 1.7.1 (AB Sciex Instruments, CA, USA). Peptide separation was carried out on a Discovery® BIO Wide Pore C-18-5 (250 mm × 2.1 mm, 5 μm, 300 Å) column. Elution was performed with solvent A (0.1 V/V% formic acid in water) and solvent B (0.1 V/V% formic acid in acetonitrile) at a flow rate of 0.2 mL/min. The gradient started at 5% B for 7 min, followed by a linear increase to 90% B by 48 min, and was maintained at 90% B from 55 to 63 min. The system was then returned to 5 % eluent B by 65 min and held for an additional 10 min. The column temperature was 40 °C and the injection volume was 15 µL. Nitrogen was used as the nebulizer gas (GS1), heater gas (GS2), and curtain gas with values of 35, 35 and 35 (arbitrary units), respectively. The source temperature was 350 °C and the spray voltage was set to 5000 V. Advanced Information Dependent Acquisition (IDA) mode was applied to collect MS/MS spectra from the eight most abundant precursor ions detected in the TOF survey scan. Data were recorded in “high-sensitivity” mode with a resolution of approx. 35,000 (FWHM). In the TOF MS survey scan (positive TOF MS mode), spectra were acquired in the m/z range of 300–2500 with an accumulation time of 0.1 s and a declustering potential of 60 V. The precursor selection threshold was set to 1000 cps. MS/MS spectra were collected in the m/z range of 50–3000 with an accumulation time of 0.1 s. Initial data processing was performed using PeakView (v2.2, Sciex) and Sciex OS (v. 3.4.5.828). Data files were converted with MSConvert (v. 3.0.25071, ProteoWizard) for compatibility with open-source tools. Bottom-up proteomics analysis was carried out using SearchGUI (v. 4.3.15, CompOmics) employing four algorithms (Comet, Tide, MetaMorpheus and Sage). The results were interpreted using PeptideShaker (v. 3.0.11, CompOmics). Final data evaluation was performed with custom-made Python scripts (v. 3.11.0) using the pyOpenMS library (v. 3.3.0) within the Spyder IDE (v. 5.5.0).

### Antibody conjugation

In 500 μL LoBind^®^ Eppendorf microcentrifuge tubes 100 μL of 10 μM trastuzumab (1 nmol, MedChemExpress, Cat. No. HY-P9907) solution in pH=7.4 PBS buffer was treated with 40 equiv. (from 20 mM TCEP stock solution in water). After 5/10/30/60/90 minutes incubation at 37 °C and under constant agitation (300 rpm) the TCEP was removed and buffer exchanged to PBS buffer (pH=7.4) with VivaSpin 500 (10 kDa MWCO) centrifugal concentrator. To the solution 100 equiv. alkyne probes (**19-22**) were added from 100 mM DMSO stock solution, and the mixture was incubated at 37 °C under constant agitation (300 rpm) for 1 hour. After this time, the mixture was buffer exchanged with VivaSpin 500 (10 kDa MWCO) centrifugal concentrator to wash away unreacted probes. Next, 100 μM TAMRA-PEG_3_-N_3_, 200 μM Cu(II)SO_4_, 1 mM THPTA and 5 mM freshly dissolved sodium-ascorbate were added to the mixture and it was further incubated at ambient temperature, under constant agitation (300 rpm) for overnight. The product was buffer exchanged with VivaSpin 500 (10 kDa MWCO) centrifugal concentrator into PBS buffer (pH=7.4)

### UV-FAR analysis

The FAR (fluorophore antibody ratio) was determined from UV-VIS absorbance at 280 and 552 nm. The molar extinction coefficients were applied as ε_280_^T^ = 225000 cm^−^^1^M^−1^, ε_552_^T^ = 0 cm^−1^M^−1^, ε_280_^TAMRA^ = 9340 cm^−1^M^−1^, ε_552_^TAMRA^ = 40,740 cm^−1^M^−1^. The calculation using the Lambert-Beer equation gave the TAMRA to antibody ratio on average.

### SDS-Page

Glycine-SDS-PAGE at 10% acrylamide running were performed following standard lab procedures. A 4% stacking gel was used and a broad-range MW marker (4.6–300 kDa, ProSieve QuadColor Protein Marker, Lonza) was co-run to estimate protein weights. Samples (10 μL at 10 μM) were mixed with reducing loading buffer (5 μL, composition for 6×SDS: 1 g SDS, 3 mL glycerol, 6 mL 0.5 M Tris buffer pH = 6.8, 2 mg Coomassie-blue R250 in 10 mL and an additional 2% BME), heated at 90 °C for 5 minutes. Samples were subsequently loaded into the wells in a volume of 13 μL. All gels were run at a constant 200 mA for 45 minutes. After fluorescence imaging, the gels were stained using a Coomassie brilliant blue stain (0,12 g Coomassie-blue G-250, 0,10 g Coomassie-blue R-250, 500 mL MeOH, 400 mL distilled water, 100 mL acetic acid), after washing it was rested at room temperature for 16 h in a water-ethanol mixture. The gels were then imaged using GelDoc (Biorad).

### LC-MS measurements of antibody conjugates

The molecular masses of antibody conjugates were determined using a Triple TOF 5600+ (Sciex, Singapore, Woodlands) equipped with a DuoSpray IonSource and coupled with a Shimadzu Prominence LC20 UFLC (Shimadzu, Japan) system. Data acquisition and processing were performed using Analyst TF version 1.7.1 (AB Sciex Instruments, CA, USA). Samples were buffer-exchanged into ultrapure water and adjusted to a final concentration of 5 μM. Prior to MS analysis, 30 μL of Trastuzumab samples (*n* = 1) were deglycosylated with 1 μL PNGase F (glycerol free; New England Biolabs GmbH, P0705) at 37 °C overnight. Chromatographic separation was carried out on a PLRP-S, 1000 Å, 8 µM, 150 mM × 2.1 mM column (Agilent, UK). Elution was performed with mobile phase A (0.1 V/V% formic acid in water containing 5% acetonitrile) and B (0.1 V/V% formic acid in acetonitrile containing 5% water) using gradient conditions as follows. The elution started at 15% B and was held for 2 minutes, followed by a linear increase to 32% B over 3 minutes, which was maintained from 5 to 9 minutes. It then gradually increased to 50% B by 14 minutes and reached 95% B in the next 4 minutes, by 18 minutes. This condition was retained until 20 minutes, after which the gradient returned to 15% B by 22 minutes and was held until 25 minutes. The flow rate was 0.5 ml/min, the column temperature was kept at 60 °C and the injection volume was 10 µl. Nitrogen served as the nebulizer gas (GS1), heater gas (GS2), and curtain gas with values of 40, 45 and 40 (arbitrary units), respectively. Data were acquired in positive ESI mode over an m/z range of 500-5000 with a 1 s accumulation time applied. The source temperature was set to 400 °C and the spray voltage to 5000 V, and the declustering potential to 80 V. Raw spectra were deconvoluted to obtain neutral molecular mass using PeakViewTM V.2.2 (version 2.2, Sciex, Redwood City, CA, USA).

### Confocal microscopy imaging

MDA-MB-231 (HER2^lo^) and SKOV-3 (HER2^hi^) cell lines were cultured in sterile flasks at 37 °C in a humidified incubator with 5% CO_2_. Cells were maintained in RPMI-1640 (Biosera, Cholet, France) medium supplemented with 10% FBS (Foetal Bovine Serum South American; Biosera, Cholet, France), Penicillin/Streptomycin Solution (100x, Biosera, Cholet, France). All cell culture procedures were carried out in a laminar flow biosafety cabinet.

MDA-MB-231 (HER2^lo^) and SKOV-3 (HER2^hi^) cells were detached using acutase and collected by centrifugation at 300 g for 5 min. One million cells were prepared per sample with three replicates for each cell. Cells were washed with PBS, removing the supernatant after each wash. To block non-specific binding, 100 µL of 3% bovine serum albumin (BSA) was added to each tube and incubated for 30 minutes. Subsequently, 100 µL of primary antibody (T-TAMRA in 1:1000 dilution) was added and the samples were incubated for 45 minutes at room temperature. The cells were washed three times with PBS, after which 100 µL of secondary antibody (Fluorescein AffiniPure Goat Anti-Human IgG in 1:200 dilution) was added and incubated in the dark for 45 min. After the incubation, cells were washed three times with PBS, then resuspended in 300 µL PBS. Glass coverslips were sterilized with 90% ethanol, air-dried and placed in six-well cell culture plates (Starstedt, Nümbrecht, Germany). 100 µL fibronectin (Sigma, St. Louis, Missouri, USA) per well was added and incubated for 30 minutes at 37 °C. Cells were suspended using trypsin-EDTA (Biosera, Cholet, France), washed with PBS (Phosphate Buffered Saline, Biosera, Cholet, France), and approximately 200,000 cells were seeded into each well in 1 ml of culture medium. After 24 h incubation at 37 °C, the cells were washed three times with PBS and fixed with 4% paraformaldehyde (Thermo Scientific, Waltham, Massachusetts, USA) for 10 minutes. After additional PBS washes, non-specific binding sites were blocked by incubation with BSA for 30 min. The cells were then incubated with the dye-conjugated primary antibody (20 µg/mL) for 1 h. After washing with PBS, nuclei were stained with Hoechst (Sigma, St. Louis, Missouri, USA) dye and the samples were mounted using ProLong Antifade Mountant (Thermo Scientific, Waltham, Massachusetts, USA). Fluorescence imaging was performed using a Zeiss LSM 710 microscope (Zeiss, Jena, Germany).

### In-gel proteomics experiments

HEK293 cells were grown in DMEM containing FBS. Before incubation with molecules, the cells were washed twice with PBS and then incubated with 10 µM molecules for 1 hour in one of three conditions – medium with FBS, medium without FBS, and PBS. The cells were then washed twice with PBS and harvested. For testing of incubations of the molecule directly in lysates, the incubation step was omitted, and the cells were harvested immediately after the first set of PBS washes. The cells were then lysed in RIPA buffer supplemented with protease inhibitors (Sigma P8340) by suspending the cells in the buffer and incubating on ice with occasional vortexing for 15 minutes. The samples were then centrifuged (21000×g at 4 °C) for 10 minutes, and the protein concentration in the supernatant was measured using BCA. 50 µl samples were prepared in RIPA at 1 mg/ml and supplemented with 1.8 µl of CuSO_4_:THPTA (in water), 1 µl of 5 mM TAMRA azide (in DMSO), and 1.5 µl of freshly dissolved 200 mM sodium ascorbate (in water). For samples in which molecules were added directly to lysates, lysate samples were supplemented with 10 µM molecule for 1 hour at room temperature, and then the click reaction was set up as described before. The click reaction proceeded for 1.5 hours at room temperature in the dark. Proteins were precipitated using 150 µl water, 50 µl chloroform and 200 µl methanol, with intense vortex followed by centrifugation (21,000 × *g*, 10 minutes at 4 °C). After removal of the top layer, the pellet was washed twice with 200 µl methanol and air dried. The samples were dissolved in 20 µl of 1XLDS sample buffer supplemented with 5 mM DTT and heated to 70°C for 10 minutes. After cooling, 10 µl from each sample was loaded on Bis-Tris 4-20% gradient gels (GenScript) and run at 55 mAmp per gel until the colour ran out. The gels were fixed using 45% methanol, 45% water, 10% acetic acid (2 washes, 5 minutes each) and neutralized using 100 mM Tris pH = 8 in water (2 washes, 5 minutes each). The gels were then imaged using GelDoc (Biorad).

### Pull-down proteomics experiments on HEK293 lysate with probes 19-22

HEK293 cells were grown in DMEM + FBS and harvested. The cells were lysed by resuspension in ice - cold Mg/Ca-free phosphate-buffered saline, supplemented with protease inhibitors (Sigma P8340). The samples (*n* = 3) were sonicated by probe (22% amplitude, 16 pulses of 2 seconds), followed by centrifugation (21,000 × *g* at 4 °C). The protein concentration was estimated using BCA, and the lysate was diluted to 2 mg/ml in PBS. Samples of 350 µl were mixed with 3.5 µl of 10 mM compounds (giving 100 µM final concentration) and incubated at 25°C for 1 hour in the dark. Following this incubation, the samples were supplemented with 1 µl of 50 mM biotin azide (Click Chemistry Tools), 2.5 µl of CuSO4:THPTA 100 mM and 2.5 µl of freshly dissolved 200 mM sodium ascorbate. The reaction proceeded for 1.5 hours at 25 °C in the dark. The proteins were precipitated by the addition of 1050 µl water, 1400 µl methanol and 350 µl chloroform, followed by intense vortex and centrifugation (3200 × *g*, 10 min at 4°C). After removal of the top later, the pellet was washed twice with 1 ml methanol and air dried. The pellets were dissolved in 250 µl 2.5% SDS in PBS using sonication (22%, 10 pulses of 2 seconds), diluted to 5 ml with PBS, then added PBS-washed SA beads from Cytiva (50 µl beads each sample). Beads were tumbled in the solution for 3 hours at 25°C, then washed as follows: 3 washes with 0.4 ml of 1% SDS/PBS; resuspension in 0.3 ml 1% SDS/PBS, addition of 3 µl 1 M DTT, followed by 45 min incubation at 25 °C; addition of 25 µl of 0.8 M iodoacetamide (freshly dissolved in water), followed by 40 min incubation at 25 °C; 3 washes with 0.6 ml of 6 M Urea (freshly dissolved) in PBS; 5 washes with 0.6 ml of 20% methanol in PBS; 1 wash with 0.6 ml of PBS; two washes with 0.6 ml water.

The beads were then transferred to new tubes using two portions of 100 µl 50 mM triethylammonium bicarbonate (TEAB), and 2 µg of trypsin (Promega) were added to each sample. The samples were shaken overnight at 37 °C. Following the digestion, the beads were centrifuged, and the supernatant was transferred to a new tube. The beads were further washed with 50 mM TEAB + 2 M NaCl, and the wash was combined with the first supernatant. The samples were mixed with 40 µl of 1% TFA in water, and desalted using Oasis desalting columns (Waters) as per the manufacturer’s instructions. The eluted samples were evaporated and dissolved in 40 µl of 3% acetonitrile in water + 0.1% formic acid. 1.5 µl were injected per sample. 3 samples were injected per condition. Samples were analyzed using EASY-nLC 1200 nano-flow UPLC system, using PepMap RSLC C18 column (2 μm particle size, 100 Å pore size, 75 μm diameter × 50 cm length), mounted using an EASY-Spray source onto an Exploris 240 mass spectrometer. uLC/MS-grade solvents were used for all chromatographic steps at 300 nL/min. The mobile phase was: (A) H2O  +  0.1% formic acid and (B) 80% acetonitrile + 0.1% formic acid. Peptides were eluted from the column into the mass spectrometer using the following gradient: 1–40% B in 160 min, 40–100% B in 5 min, maintained at 100% for 20 min, 100 to 1% in 10 min, and finally 1% for 5 min. Ionization was achieved using a 2100 V spray voltage with an ion transfer tube temperature of 275 °C. Data were acquired in data-dependent acquisition (DDA) mode. MS1 resolution was set to 120,000 (at 200 m/z), a mass range of 375–1650 m/z, normalized AGC of 300%, and the maximum injection time was set to 20 ms. MS2 resolution was set to 15,000, quadrupole isolation 1.4 m/z, normalized AGC of 50%, automatic maximum injection time, and HCD collision energy at 30%.

For the triplicates in each condition, data analysis was performed separately using Fragpipe (version 19.1) using Msfragger search engine (version 3.8)119,120, IonQuant 1.8.10121, and Philosopher 4.8.1122. Analysis was performed using a human proteome database from December 2022 (Uniprot) with contaminants added and with Streptavidin added manually as a contaminant. Msfragger analysis was performed using Trypsin as the enzyme that cuts after Arg and Lys, with up to 2 missed cleavages, peptide length 7–50 and the N-terminal methionine removed. N-terminal acetylation and methionine oxidation were defined as variable modifications and carbamidomethyl was defined as a fixed modification. A false discovery rate of 0.01 was used both at the peptide and the protein level. Label-Free Quantification was performed using IonQuant, with Match Between Runs enabled with a tolerance of 1 min. After the analysis, the data for each condition were reviewed and only the data for proteins in which two or more nonzero intensity values were obtained were retained. The data for all conditions were then combined (with proteins not identified in a particular set receiving intensity values of 0 in all replicates). At this point, intensities were converted to Log2 values and zero intensity data points were replaced with a Log2 value of 18. Conditions were compared using students t test, and proteins were defined as significantly pulled down if the average intensity for the protein was two log units higher than the DMSO value with a *p*-value less than 0.01. The mass spectrometry proteomics data have been deposited to the ProteomeXchange Consortium via the PRIDE^108^ partner repository with the dataset identifier PXD054470.

### Peptide targeted ABPP experiments

HEK293 cells were grown as described before and lysed in RIPA buffer + protease inhibitors. After BCA, the lysate was diluted to 2 mg/ml in PBS, and 500 µl of lysate was treated with 1 µl of 50 mM molecule (giving 0.1 mM molecule). Incubation of samples (*n* = 3) was 1 hour at room temperature, after which the click reaction to DADPS biotin azide (Click Chemistry Tools) was performed by the addition of 1.88 µl DADPS biotin azide, 25 µl of THPTA:CuSO_4_ 100 mM: 20 mM, and 20 µl of freshly dissolved 200 mM sodium ascorbate. The click reaction proceeded in the dark for 1.5 hours and precipitated using methanol-chloroform. The pellets were dispersed in 0.5 ml of 100 mM ammonium bicarbonate (freshly dissolved) and dispersed by sonication (22%, 4 pulses of 2 seconds on ice). Each sample was supplemented with 10 µg of sequencing-grade trypsin and incubated overnight at 37 °C, leading to clarification of the solution. The peptides were reduced by 5 µl of 1 M DTT (30 minutes incubation at 37 °C), followed by 25 µl of 0.8 M iodoacetamide + 40 minutes incubation in the dark, followed by 20 µl of 1 M DTT to neutralize the rest of the iodoacetamide. At this stage 50 µl of streptavidin agarose beads + 2 ml of 100 mM ammonium bicarbonate solution were added and the samples were tumbled for 2 hours at room temperature. The beads were washed 3 times PBS, twice with PBS + 2 M NaCl, and 3 times with water (600 µl per wash), and then dispersed in 10% formic acid in water (400 µl) and incubated at room temperature for 2 hours to elute the peptides. The supernatant was collected and lyophilized. Desalting of the samples using Oasis columns, dissolving and running in data-dependent acquisition was performed as described before. For each sample, analysis was performed separately with FragPipe version 23.1. MSfragger 4.3 and IonQuant 1.11.11, with carbamidomethyl as a variable modification on cysteine (up to 3 instances), and both the Oxo and the Thio adducts as variable modifications on cysteine, lysine, histidine and tyrosine (up to one instance). The mass spectrometry proteomics data have been deposited to the ProteomeXchange Consortium via the PRIDE^108^ partner repository with the dataset identifier PXD069527.

### Pull-down proteomics experiments on Mino lysate with probes 16-18

Mino cells were grown in RPMI medium + 15% FBS and harvested. The cells were lysed by resuspension in ice - cold Mg / Ca-free phosphate-buffered saline, supplemented with protease inhibitors (Sigma P8340). The samples were sonicated by probe (22% amplitude, 16 pulses of 2 seconds), followed by centrifugation (21000 × *g* at 4 °C). The protein concentration was estimated using BCA, and the lysate was diluted to 2 mg/ml in PBS. Molecules (or DMSO) were added to a concentration of 1 µM or 10 µM and incubated with the lysates for 1 hour at room temperature. At this point to each sample (250 µl, *n* = 3) was added 0.75 µl of 50 mM biotin azide, 12.5 µl CuSO_4_:THPTA 20 mM:100 mM and 10 µl of 200 mM freshly dissolved sodium ascorbate. The samples were incubated at room temperature in the dark for 1.5 hours and precipitated with methanol and chloroform. The samples were then processed using the same protocol used for HEK293 lysates treated with molecules 19-22, but with 10 µl beads instead of 50 µl beads. Data were acquired in data-independent acquisition (DIA) using the same gradient described. Ionization was achieved using 1800 V spray voltage with an ion transfer tube temperature of 275 °C. MS1 resolution was set to 60,000 (at 200 m/z), a mass range of 375–1650 m/z, normalized AGC of 300%, and the maximum injection time was set to 20 ms. DIA was measured with a precursor mass range of 390-920, 17 isolation windows, orbitrap resolution of 30,000 at 200 m/z, maximum injection time of 50 ms and HCD collision energy of 27%. Fragpipe was used to analyze the data with quantification by DIA-NN (version 1.8.2 beta), with carbamidomethyl as a fixed modification, and methionine oxidation and N-terminal acetylation as variable modifications. The mass spectrometry proteomics data have been deposited to the ProteomeXchange Consortium via the PRIDE^108^ partner repository with the dataset identifier PXD069336.

### Synthetic procedures

#### General procedure for the synthesis of chloroacetamides

The amine (10 mmol) and triethylamine (1.67 mL, 12 mmol, 1.2 equiv.) were dissolved in 10 mL dichloromethane, and at 0 °C 2-chloroacetyl chloride (0.88 mL, 11 mmol, 1.1 equiv.) was added dropwise, and the mixture was stirred at room temperature for 90 min. The reaction mixture was then washed with 10 mL saturated NaHCO_3_, 10 mL aqueous NH_4_Cl, and 10 mL brine. Organic phase was separated, dried over Na_2_SO_4_ and concentrated to obtain the corresponding chloroacetamides.

#### 2-chloroacetanilide (**3a**)

Yield 72%, white powder. ^1^H NMR (500 MHz, DMSO-*d*_6_) δ 10.26 (s, 1H), 7.59 (d, *J* = 7.9 Hz, 2H), 7.34 (t, *J* = 7.9 Hz, 2H), 7.09 (t, *J* = 7.4 Hz, 1H), 4.25 (s, 2H) ppm.

#### N-(3,5-bis(trifluoromethyl))-2-chloroacetamide (**3b**)

Yield 69%, white powder. ^1^H NMR (500 MHz, DMSO-*d*_6_) δ 10.92 (s, 1H), 8.26 (s, 2H), 7.82 (s, 1H), 4.34 (s, 2H) ppm.

#### N-benzyl-2-chloroacetamide (**3c**)

Yield 98%, brownish solid, ^1^H NMR (500 MHz, DMSO-*d*_6_) δ 8.73 (s, 1H), 7.37 – 7.23 (m, 5H), 4.33 (d, *J* = 6.0 Hz, 2H), 4.13 (s, 2H) ppm.

#### 2-chloro-1-(piperidin-1-yl)ethanone (**3 d**)

Yield: 93%, yellow solid, ^1^H NMR (500 MHz, CDCl_3_) δ 4.08 (s, 2H), 3.60 – 3.54 (m, 2H), 3.48–3.43 (m, 2H), 1.69–1.63 (m, 3H), 1.62–1.56 (m, 2H) ppm.

#### 1-(3-(4-Amino-3-(4-phenoxyphenyl)-1H-pyrazolo[3,4-d]pyrimidin-1-yl)piperidin-1-yl)-2-chloroethanone (**12**)

Yield 67%, white powder. ^1^H NMR (500 MHz, CDCl_3_) δ 8.39 (d, *J* = 9.4 Hz, 1H), 7.66 (d, *J* = 8.4 Hz, 2H), 7.41 (t, *J* = 7.6 Hz, 2H), 7.19 (m, 3H), 7.11 (d, *J* = 7.9 Hz, 2H), 5.64 (s, 2H), 5.09–4.84 (m, 1H), 4.63 (dd, *J* = 147.9, 12.6 Hz, 1H), 4.18–4.10 (m, 2H) 4.00 (dd, *J* = 74.8, 13.3 Hz, 1H), 3.65 (dt, J = 201.4 Hz, 12.3 Hz, 1H), 3.12 (dt, *J* = 22.9, 11.8 Hz, 1H), 2.51–2.31 (m, 1H), 2.32–2.23 (m, 1H), 2.10–1.96 (m, 1H), 1.91–1.79 (m, 2H).ppm.

#### 1-(3-(4-amino-3-(4-(3-ethynylphenoxy)phenyl)-1H-pyrazolo[3,4-d]pyrimidin-1-yl)piperidin-1-yl)-2-chloroethanone (**17**)

Yield: 66% yellow solid, ^1^H NMR (500 MHz, CDCl_3_) δ 8.36 (d, *J* = 11.5 Hz, 1H), 7.68 (d, *J* = 8.3 Hz, 2H), 7.39–7.30 (m, 2H), 7.22–7.16 (m, 3H), 7.11 (d, *J* = 8.0 Hz, 1H), 5.91 (bs, 2H), 5.03–4.86 (m, 1H), 4.83–4.42 (m, 1H), 4.25–4.15 (m, 1H), 4.12 – 4.04 (m, 1H), 4.00–3.81 (m, 1H), 3.52–3.22 (m, 1H), 3.12 (s, 1H), 2.95 (t, *J* = 11.4 Hz, 1H), 2.55–2.20 (m, 2H), 2.09–1.62 (m, 3H) ppm.

#### 2-Chloro-N-(4-(prop-2-yn-1-yloxy)phenyl)acetamide (**19**)

Yield 79%, white solid ^1^H NMR (500 MHz, CDCl_3_) δ 8.38 (bs, 1H), 7.35–7.27 (m, 2H), 7.05–6.96 (m, 2H), 4.84 (d, *J* = 5.2 Hz, 2H), 4.73 (d, *J* = 2.4 Hz, 2H), 4.65 (s, 2H), 2.56 (t, *J* = 2.4 Hz, 1H) ppm. ^13^C NMR (75 MHz, CDCl_3_) δ 178.9, 145.4, 138.8, 130.3, 129.2, 115.3, 75.6, 55.9, 43.4, 42.6 ppm. HRMS: (M + H)^+^ calcd. for C_12_H_13_NO_2_Cl^+^, 238.0634; found, 238.0632.

#### General procedure for the synthesis of fluoroacetamides

The chloroacetamide (3 mmol) was dissolved in 10 mL acetonitrile:DMSO 3:2 at 80 °C, and caesium fluoride (1.37 *g*, 9 mmol, 3 equiv.) was added. When needed, further 3 equiv. CsF was added. The reaction mixture was heated overnight. Then 30 mL ethyl acetate was added and washed with 2 × 20 mL water and 20 mL saturated NH_4_Cl. The organic phase was dried over Na_2_SO_4_ and evaporated to silica. The product was obtained after flash chromatography (hexane-ethyl acetate).

#### N-phenyl-2-fluoroacetamide (**4a**)

Yield 58%, white powder. ^1^H NMR (500 MHz, DMSO-*d*_6_) δ 10.05 (s, 1H), 7.65 (d, *J* = 7.9 Hz, 2H), 7.33 (t, *J* = 7.9 Hz, 2H), 7.10 (t, *J* = 7.4 Hz, 1H), 4.98 (d, *J* = 46.9 Hz, 2H) ppm.

#### N-(3,5-bis(trifluoromethyl)phenyl)-2-fluoroacetamide (**4b**)

Yield 47%, white powder. ^1^H NMR (500 MHz, DMSO-*d*_6_) δ 10.72 (s, 1H), 8.38 (s, 2H), 7.82 (s, 1H), 5.06 (d, J = 46.5 Hz, 2H) ppm.

#### N-benzyl-2-fluoroacetamide (**4c**)

Yield: 32%, white solid, ^1^H NMR (500 MHz, CDCl_3_) δ 7.43–7.34 (m, 2H), 7.34–7.28 (m, 3H), 6.62 (bs, 1H), 4.88 (s, 1H), 4.78 (s, 1H), 4.52 (d, *J* = 6.0 Hz, 2H) ppm.

#### 2-fluoro-1-(piperidin-1-yl)ethanone (**4 d**)

Yield: 49%, brown oil, ^1^H NMR (500 MHz, CDCl_3_) δ 4.95 (s, 1H), 4.86 (s, 1H), 3.54–3.44 (m, 2H), 3.30–3.21 (m, 2H), 1.70–1.42 (m, 6H) ppm.

#### 1-(3-(4-amino-3-(4-phenoxyphenyl)-1H-pyrazolo[3,4-d]pyrimidin-1-yl)piperidin-1-yl)-2-fluoroethanone (**13**)

Yield 48%, white crystals. ^1^H NMR (500 MHz, CDCl_3_) δ 8.38 (s, 1H), 7.66 (d, *J* = 8.1 Hz, 2H), 7.41 (t, *J* = 7.4 Hz, 2H), 7.19 (t, *J* = 10.9 Hz, 3H), 7.11 (d, *J* = 7.7 Hz, 2H), 5.67 (s, 2H), 5.16–4.86 (m, 3H), 4.61 (dd, *J* = 151.4, 11.3 Hz, 1H), 3.95 (d, J = 11.9 Hz, 1H), 3.79-3.75 (m, 1H), 3.33 (dt, *J* = 123.4, 11.2 Hz, 1H), 3.07–2.96 (m, 1H), 2.98 (t, *J* = 11.0 Hz, 1H), 2.54–2.33 (m, 1H), 2.33–2.23 (m, 1H), 1.32 (t, *J* = 7.1 Hz, 2H) ppm. ^13^C NMR (125 MHz, CDCl_3_) δ 189.6, 165.6, 157.9, 155.9, 155.8, 130.0, 127.8, 127.5, 124.1, 122.0, 119.6, 119.1, 110.0, 80.8, 79.3, 53.2, 48.8, 45.5, 42.3, 30.2, 29.8, 23.7 ppm. HRMS: (M + H)^+^ calcd. for C_24_H_24_N_6_O_2_F^+^, 447.1943; found, 447.1946.

#### 2-Fluoro-N-(4-(prop-2-yn-1-yloxy)phenyl)acetamide (**20**)

Yield 59%, pale crystals (130 mg, 59%). ^1^H NMR (500 MHz, CD_3_CN) δ 7.38 (bs, 1H), 7.34–7.27 (m, 2H), 7.03–6.96 (m, 2H), 4.88 (s, 1H), 4.79 (s, 1H), 4.77 (d, *J* = 2.4 Hz, 3H), 4.41 (d, *J* = 6.2 Hz, 2H), 2.86 (t, *J* = 2.4 Hz, 1H) ppm. ^13^C NMR (125 MHz, CD_3_CN) 168.6, 157.7, 133.0, 129.8, 115.8, 82.1, 80.6, 79.9, 76.8, 56.6, 42.4 ppm. HRMS: (M + H)^+^ calcd. for C_12_H_13_NO_2_F^+^, 222.0930; found, 222.0925.

#### General procedure for the synthesis of chlorothioacetamides and fluorothioacetamides

The chloro/fluoroacetamide (0.4 mmol) was dissolved in 2 mL dry methyltetrahydrofurane, and phosphorus pentasulfide (191 mg, 0.86 mmol, 2 equiv.) was added. The reaction mixture was stirred at 70 °C for 1 h, then evaporated to silica, and purified by flash chromatography (hexane-ethyl acetate).

#### N-phenyl-2-chlorothioacetamide (**5a**)

Yield 42%, white crystals. ^1^H NMR (500 MHz, CDCl_3_) δ 9.71 (s, 1H), 7.78 (d, *J* = 7.9 Hz, 2H), 7.47 (t, *J* = 7.6 Hz, 2H), 7.35 (t, *J* = 7.3 Hz, 1H), 4.73 (s, 2H) ppm. ^13^C NMR (125 MHz, CDCl_3_) δ 191.3, 137.6, 129.1, 127.6, 123.4, 50.6 ppm. HRMS: (M-H)^–^ calcd. for C_8_H_7_SNCl^–^, 183.9988; found, 183.9992.

#### N-(3,5-bis(trifluoromethyl)phenyl)-2-chlorothioacetamide (**5b**)

Yield 31%, white powder. ^1^H NMR (500 MHz, DMSO-*d*_6_) δ 9.60 (s, 1H), 8.13 (s, 2H), 7.61 (s, 1H), 4.52 (s, 2H) ppm. ^13^C NMR (75 MHz, CDCl_3_) δ 187.9, 133.9, 127.8, 127.5, 118.9, 118.4, 118.4, 115.8, 45.8 ppm. HRMS: (M-H)^–^ calcd. for C_10_H_5_SNF_6_Cl^–^, 319.9735; found, 319.9747.

#### N-benzyl-2-chloroethanethioamide (**5c**)

Yield: 70%, yellow solid, 1H NMR (500 MHz, CDCl_3_) δ 8.45 (s, 1H), 7.45–7.32 (m, 5H), 4.91 (d, J = 5.3 Hz, 2H), 4.65 (s, 2H) ppm. ^13^C NMR (125 MHz, CDCl_3_) δ 193.2, 135.3, 129.1, 128.4, 128.2, 50.1, 49.8 ppm. HRMS: (M + H)^+^ calcd. for C_9_H_11_NSCl^+^, 200.0295; found, 200.0292.

#### 2-chloro-1-(piperidin-1-yl)ethanethione (**5 d**)

Yield: 35%, yellow oil, ^1^H NMR (500 MHz, CDCl_3_) δ 4.47 (s, 2H), 4.09 (s, 2H), 3.69 (s, 2H), 1.66 (s, 6H) ppm. ^13^C NMR (125 MHz, CDCl_3_) δ 192.4, 52.2, 51.8, 48.8, 26.7, 25.3, 23.8 ppm. HRMS: (M + H)^+^ calcd. for C_7_H_13_NSCl^+^, 178.0451; found, 178.0448.

#### N-phenyl-2-fluorothioacetamide (**6a**)

Yield 29%, pale powder (25 mg, 29%). ^1^H NMR (500 MHz, DMSO-*d*_6_) δ 11.61 (s, 1H), 7.74 (d, *J* = 7.8 Hz, 2H), 7.43 (t, *J* = 7.9 Hz, 2H), 7.30 (t, *J* = 7.4 Hz, 1H), 5.25 (d, *J* = 47.8 Hz, 2H) ppm. ^13^C NMR (75 MHz, CDCl_3_) δ 195.0 (d, *J* = 8.2 Hz), 138.7 Hz, 129.0 Hz, 127.2 Hz, 124.9 Hz, 86.1 (d, *J* = 114.0 Hz) ppm. HRMS: (M-H) ^–^ calcd. for C_8_H_7_SNF^–^, 168.0288; found, 168.0287.

#### N-(3,5-bis(trifluoromethyl)phenyl)-2-fluorothioacetamide (**6b**)

Yield 21%, as white powder. ^1^H NMR (500 MHz, CDCl_3_) δ 9.55 (s, 1H), 8.43 (s, 2H), 7.83 (s, 1H), 5.25 (d, *J* = 48.2 Hz, 2H) ppm. ^13^C NMR (125 MHz, CDCl_3_) δ 194.9, 138.4, 132.7, 132.4, 123.9, 123.1, 123.0, 121.7, 120.6, 85.8 (d, *J* = 197.4 Hz) ppm. HRMS: (M + H)^+^ calcd. for C_10_H_5_SNF_7_^–^, 304.0031; found, 303.9973.

#### N-benzyl-2-fluoroethanethioamide (**6c**)

Yield: 71%, yellow solid, ^1^H NMR (500 MHz, CDCl_3_) δ 8.20 (s, 1H), 7.44–7.33 (m, 5H), 5.16 (d, *J* = 48.1 Hz, 2H), 4.93 (d, *J* = 5.5 Hz, 2H) ppm. ^13^C NMR (125 MHz, CDCl_3_) δ 195.4, 135.5, 129.0, 128.4, 128.4, 86.0, 84.2, 48.6 ppm. HRMS: (M + H)^+^ calcd. for C_9_H_11_NSF^+^, 184.0590; found, 184.0587.

#### 2-fluoro-1-(piperidin-1-yl)ethanethione (**6 d**)

Yield: 43%, yellow oil, ^1^H NMR (500 MHz, CDCl_3_) δ 5.32 (s, 1H), 5.23 (s, 1H), 4.21–4.15 (m, 2H), 3.78–3.72 (m, 2H), 1.79 – 1.68 (m, 6H) ppm. ^13^C NMR (125 MHz, CDCl_3_) δ 192.0, 86.2 (d, *J* = 186.9 Hz), 52.0 (d, *J* = 7.8 Hz), 51.9, 26.9, 25.4, 24.1 ppm. HRMS: (M + H)^+^ calcd. for C_7_H_13_NSF^+^, 162.0747; found, 162.0745.

#### *N-(3-(7H-pyrrolo[2,3-d]pyrimidin-4-yl)phenyl)-2-chloroethanethioamide* (**9**)

Stable in DMSO solution, yellow. ^1^H NMR (500 MHz, DMSO-*d*_6_) δ 12.93 (s, 1H), 12.37–12.24 (m, 1H), 9.02 (s, 1H), 8.86 (s, 1H), 8.10 (t, *J* = 8.8 Hz, 2H), 7.90 (s, 1H), 7.72 (t, *J* = 7.9 Hz, 1H), 7.17 (s, 1H), 4.66 (s, 2H) ppm. In the ^1^H NMR 2-methyltetrahydrofuran from the reaction is visible. HRMS: (M + H)^+^ calcd. for C_14_H_12_N_4_SCl^+^, 303.0471; found, 303.0468.

#### N-(3-(7H-pyrrolo[2,3-d]pyrimidin-4-yl)phenyl)-2-chloropropanethioamide (**10**)

Stable in DMSO solution, yellow. ^1^H NMR (500 MHz, DMSO-*d*_6_) 13.06 (s, 1H), 12.15 (s, 1H), 9.07 (s, 1H), 8.71 (s, 1H), 8.11–7.92 (m, 3H), 7.75 (t, *J* = 7.9 Hz, 1H), 7.16 (s, 1H), 5.19 (q, *J* = 6.6 Hz, 1H). In the ^1^H NMR, MeTHF from the reaction is visible. Methyl group ~ 1.80 ppm is below the signal of the 2-methyltetrahydrofuran solvent. HRMS: (M + H)^+^ calcd. for C_15_H_14_N_4_SCl^+^, 317.0628; found, 317.0631.

#### 1-(3-(4-Amino-3-(4-phenoxyphenyl)-1H-pyrazolo[3,4-d]pyrimidin-1-yl)piperidin-1-yl)-2-chloroethanethione (**14**)

Yield 35%, yellow solid. ^1^H NMR (500 MHz, CDCl_3_) δ 7.63 (bs, 2H), 7.43 (t, *J* = 7.8 Hz, 2H), 7.21 (q, *J* = 7.3 Hz, 5H), 7.13 (d, *J* = 8.2 Hz, 2H), 7.00 (s, 1H), 5.55–5.30 (m, 1H), 5.23–4.98 (m, 2H), 4.66 – 4.59 (m, 2H), 4.57–4.46 (m, 1H), 4.05–3.96 (m, 2H), 3.96–3.89 (m, 2H), 3.79–3.70 (m, 2H) ppm. ^13^C NMR (125 MHz, CDCl_3_) δ 155.8, 151.6, 147.5, 138.4, 138.0, 135.9, 130.2, 129.9, 129.1, 128.3, 125.6, 125.4, 124.6, 120.1, 120.0, 119.5, 67.8, 33.1, 30.4, 29.8, 25.9, 21.0 ppm. HRMS: (M + H)^+^ calcd. for C_24_H_24_N_6_OSCl^+^, 479.1420; found, 479.1410.

#### 1-(3-(4-Amino-3-(4-phenoxyphenyl)-1H-pyrazolo[3,4-d]pyrimidin-1-yl)piperidin-1-yl)-2-fluoroethanethione (**15**)

Yield 30%, pale-yellow solid. ^1^H NMR (500 MHz, CDCl_3_) δ 8.35 (d, *J* = 14.8 Hz, 1H), 7.62 (d, *J* = 8.1 Hz, 2H), 7.44 (t, *J* = 7.7 Hz, 2H), 7.26–7.04 (m, 7H), 5.16–4.88 (m, 3H), 4.79 – 4.42 (m, 1H), 4.06–3.71 (m, 2H), 3.54 – 3.17 (m, 1H), 2.53–2.21 (m, 3H), 2.17–2.00 (m, 1H), 1.88–1.70 (m, 1H) ppm. ^13^C NMR (125 MHz, CDCl_3_) δ 160.0, 153.8, 153.6, 147.0, 146.4, 145.6, 130.2, 129.7, 125.0, 124.7, 124.7, 120.0, 119.2, 103.4, 100.3, 87.0, 85.5, 54.2, 53.6, 52.8, 50.9, 50.6, 29.8, 24.8 ppm. HRMS: (M + H)^+^ calcd. for C_24_H_24_N_6_OSF^+^, 463.1716; found, 463.1715.

#### 1-(3-(4-amino-3-(4-(3-ethynylphenoxy)phenyl)-1H-pyrazolo[3,4-d]pyrimidin-1-yl)piperidin-1-yl)-2-chloroethanethione (**18**)

Yield 8%, pale yellow solid. ^1^H NMR (500 MHz, CDCl_3_) δ 8.41 (s, 1H), 7.70 (d, *J* = 7.5 Hz, 2H), 7.43–7.31 (m, 3H), 7.23–7.16 (m, 3H), 7.12 (d, *J* = 7.7 Hz, 1H), 5.67–5.26 (m, 3H), 5.09 (p, *J* = 5.2 Hz, 1H), 4.67–4.29 (m, 3H), 4.12–3.79 (m, 1H), 3.41 (dt, *J* = 48.6, 11.2 Hz, 1H), 3.12 (s, 1H), 2.55 – 1.84 (m, 3H) ppm. ^13^C NMR (125 MHz, CDCl_3_) δ 209.3, 159.1, 156.0, 130.1, 130.1, 130.0, 130.0, 127.8, 127.8, 122.7, 122.7, 120.2, 120.1, 119.5, 54.4, 53.8, 53.3, 52.0, 50.9, 50.7, 48.8, 48.7, 29.7, 29.7, 29.5, 24.7, 23.0 ppm. HRMS: (M + H)^+^ calcd. for C_26_H_24_N_6_OSCl^+^, 503.1421; found, 503.1437.

#### 2-Chloro-N-(4-(prop-2-yn-1-yloxy)phenyl)ethanethioamide (**21**)

Yield 35%, pale yellow solid. ^1^H NMR (300 MHz, CDCl_3_) δ 8.38 (bs, 1H), 7.35–7.27 (m, 2H), 7.05–6.96 (m, 2H), 4.84 (d, *J* = 5.2 Hz, 2H), 4.73 (d, *J* = 2.4 Hz, 2H), 4.65 (s, 2H), 2.56 (t, *J* = 2.4 Hz, 1H) ppm. ^13^C NMR (125 MHz, CDCl_3_) δ 191.2, 156.5, 131.4, 125.0, 115.3, 78.2, 75.9, 56.1, 50.5 ppm. HRMS: (M + H)^+^ calcd. for C_12_H_13_NOSCl^+^, 254.0406; found, 254.0401.

#### 2-Fluoro-N-(4-(prop-2-yn-1-yloxy)phenyl)ethanethioamide (**22**)

Yield 42%, white crystals. ^1^H NMR (500 MHz, CD_3_CN) δ 9.07 (bs, 1H), 7.41–7.34 (m, 2H), 7.04–6.97 (m, 2H), 5.21 (s, 1H), 5.11 (s, 1H), 4.90 (d, *J* = 6.1 Hz, 2H), 4.78 (d, *J* = 2.4 Hz, 2H), 2.84 (t, *J* = 2.4 Hz, 1H) ppm. ^13^C NMR (125 MHz, CD_3_CN) 158.0, 130.8, 130.5, 115.9, 87.2, 85.6, 79.8, 76.8, 56.6, 47.7 ppm. HRMS: (M + H)^+^ calcd. for C_12_H_13_NOSCl^+^, 238.0701; found, 238.0696.

#### 2-(6-(dimethylamino)-3-(dimethyliminio)-3H-xanthen-9-yl)-5-(((5-((3 R,5 R)-7-(2-(4-fluorophenyl)-5-isopropyl-3-phenyl-4-(phenylcarbamoyl)-1H-pyrrol-1-yl)-3,5-dihydroxyheptanamido)pentyl)oxy)carbonyl)benzoate (**24**)

Atorvastatin (27 mg, 0.048 mmol) and TAMRA-cadaverine TFA salt (30 mg, 0.048 mmol) were dissolved in 0.5 mL DMF. PyBOP (37 mg, 0.72 mmol, 1.5 equiv.) and DIPEA (33 μL, 0.192 mmol, 4 equiv.) were added, and the reaction mixture was stirred at room temperature for 2 h. The product was isolated by preparative HPLC (acetonitrile-water) as a purple solid after lyophylization. Yield: 20%, purple solid, ^1^H NMR (500 MHz, CDCl_3_) δ 8.51 (s, 1H), 8.17 (d, *J* = 7.8 Hz, 1H), 7.54 (s, 1H), 7.24–7.06 (m, 11H), 7.03–6.91 (m, 4H), 6.86 (d, *J* = 8.9 Hz, 2H), 6.61–6.51 (m, 3H), 4.13–3.99 (m, 2H), 3.93–3.82 (m, 1H), 3.75–3.67 (m, 1H), 3.56–3.47 (m, 2H), 3.45–3.40 (m, 2H), 3.35–3.23 (m, 1H), 3.21–3.14 (m, 2H), 3.14–2.98 (m, 10H), 2.36–2.19 (m, 2H), 1.75–1.57 (m, 4H), 1.55–1.38 (m, 10H), 1.38–1.20 (m, 4H) ppm.^13^C NMR (125 MHz, CDCl_3_) δ 172.2, 169.3, 166.5, 165.0, 163.2, 161.2, 154.5 (d, *J* = 78.5 Hz), 149.7, 141.3, 138.5, 136.5, 134.8, 133.2 (d, *J* = 7.9 Hz), 132.4, 130.5, 130.0, 128.8, 128.7, 128.4, 128.3, 128.3, 127.4, 126.5, 125.3, 123.5, 121.7, 120.2, 119.6, 115.4, 115.4, 115.3, 114.7, 110.8, 109.2, 105.9, 97.6, 69.5, 67.8, 59.1, 51.4, 42.8, 42.2, 41.5, 40.9, 40.4, 39.8, 39.4, 38.8, 36.8, 33.9, 31.9, 29.7, 29.1, 28.8, 28.5, 28.5, 26.1, 23.8, 21.8, 21.7 ppm. HRMS: (M + H)^+^ calcd. for C_63_H_68_N_6_O_8_F^+^, 1055.5077; found, 1055.5111.

### Reporting summary

Further information on research design is available in the Nature Portfolio Reporting Summary linked to this article.

## Supplementary information

Supplementary_Information

Description of Additional Supplementary Files

Supplementary Data 1

Supplementary Data 2

Reporting Summary

Transparent Peer Review file

## Source data

Source Data

## Data Availability

Data supporting the findings of this study are available from the corresponding author(s) upon request. The data generated in this study are provided in the Supplementary Information, Supplementary Data files and Source Data file. Other proteomics data generated in this study have been deposited in the ProteomeXchange Consortium via the PRIDE partner repository under accession codes PXD054470, PXD069527, PXD069336. Source data are provided with this paper.
