# Supplementary material for: α-Halothioamide warheads with enhanced cysteine reactivity and specificity for covalent protein labelling
Source: Nat Commun. 2026 May 14;17:6824. doi: 10.1038/s41467-026-72993-6 (PMC13388716; doi:10.1038/s41467-026-72993-6)
Supplement: Supplementary file 1 — Supplementary_Information [file 41467_2026_72993_MOESM1_ESM.pdf]

Supplementary Information

α-Halothioamide warheads with enhanced cysteine reactivity and specificity for covalent protein labelling

László Petri†[1,2,3], Ronen Gabizon†[4], Nikolett Péczka[1,2], Péter Ábrányi-Balogh[1,2,5], József Simon[1,6], Tímea Imre[1,6], György G. Ferenczy[1,2], Nir London*[4] and György M. Keserű*[1,2,5]

1 Medicinal Chemistry Research Group, Research Centre for Natural Sciences, Magyar tudósok krt. 2, 1117 Budapest (Hungary)
2 National Laboratory of Pharmaceutical Research and Development, Research Centre for Natural Sciences, Magyar tudósok krt. 2, 1117 Budapest (Hungary)
3 Institute of Chemistry, Eötvös Loránd University, Egyetem tér 1-3., 1053 Budapest (Hungary)
4 Department of Chemical and Structural Biology, The Weizmann Institute of Science, 7610001 Rehovot (Israel)
5 Department of Organic Chemistry and Technology, Budapest University of Technology and Economics, Szt. Gellért tér 4, 1111 Budapest (Hungary)
6 MS Metabolomics Research Laboratory, Research Centre for Natural Sciences, Magyar tudósok krt. 2, 1117 Budapest (Hungary)

† These authors contributed equally.
* Correspondence: nir.london@weizmann.ac.il and keseru.gyorgy@ttk.hu

TABLE OF CONTENTS

Supplementary Tables 3

Table S1. O-S exchange effect on NBO-charge distribution localized to the adjacent carbon atom in β-position (assessed by M062X/6-311++G(d,p) DFT calculations) 3

Table S2. GSH assay and NP assay results of the covalent probes (3,6,8-11)........ 3

Table S3. Computed activation Gibbs free energies of the covalent probes (3,6,8-11)........ 4

Table S4. Positioning of the targeted cysteines and characterization of their reactivity and accessibility based on *Cy-preds* server predictions 4

Table S5. Inhibiton effeciency of covalent fragments (3a-b, 4a-b, 5a-b, 6a-b) on BTK, JAK3 and MAP2K6 kinases, reported as individual inhibition percentage of the biological duplicates, measured at 100 µM concentration of the after 60 min preincubation. 5

Table S6. Intact protein MS results summary reporting % degree of labelling on kinase targets JAK3, BTK and MAP2K6. Molar stochiometries varied across the experiments, the following equivalents of the covalent probes related to the protein of interest was applied: probes 3-6 on BTK with 20 eq., on JAK3 with 170 eq. and on MAP2K6 with 70 eq; probes 9-10 on JAK3 with 13 eq., probes 11-15 on BTK with 2 eq. 5

Table S7. Annotations of MS/MS spectrum of the modified proteins after enzymatic digestion and subsequent peptide mapping........ 6

Table S8. QM/MM-based calculation results of BTK interactions with probe 12, 14........ 7

Table S9. UV-FAR analysis results of 19-22 conjugated antibodies by biological replicates.. 8

Supplementary Figures........ 9

Figure S1. Results summary of acrylamide and thioacrylamide covalent fragments 9

Figure S2. The MSMS spectrum of the modified nonapeptide (NP) in selectivity assay........10

Figure S3. Intact protein MS spectrum of the proteins modified by covalent probes. 11

Figure S4. The MSMS spectrum of the modified proteins after enzymatic digestion and subsequent peptide mapping........12

Figure S5. Investigated kinases localized on the visualized phylogenetic kinase tree...........12

Figure S6. Dose-response curves of kinase inhibitor probes on BTK and JAK3. 13

Figure S7. QM/MM-based calculation results of BTK interactions with probe 12, 14 14

Figure S8. Dose-response curves of kinase inhibitor probes cell viablitiy effect on Ramos cells. 15

Figure S9. In vitro metabolic stability of compounds 11-15 in human liver microsomes 16

Figure S10. Structure of the applied alkyne probes (19-23)..16

Figure S11. SDS-PAGE gel image of in-gel visualization of fluorescent dye-antibody conjugates (produced by probes 19-22), showing heavy chain (HC) and light chain (LC) captured by both, coomassie brilliant blue staining (left) and fluorescence readout (right). ...16

Figure S12. Covalent modification of the heavy and light chains treated with the alkyne probes (19-22) are shown on deconvoluted MS spectrum...17

Figure S13. SDS-PAGE gel image of stability measurements on SDS-PAGE of the antibody-TAMRA conjugates. 18

Figure S14. In-gel proteomics results with 19-23 alkyne probes. 10 µM compounds were incubated in the following conditions: HEK293 cells in the presence of media + FBS. HEK293 cells in media without FBS. HEK293 cells in PBS. or lysates of HEK293 cells in RIPA buffer. Labeled lysates were clicked to TAMRA azides and analyzed using SDS-PAGE. All gels were imaged together to ensure identical exposure times. Images were taken at short (10 sec) exposure...18

Figure S15. Functional clustering of proteins exclusively labelled by 21 19

Figure S16. Fluorescent polarization assay to evaluate PDE6δ binder (21).........................19

^{1}H-NMR spectra of the covalent probes...20

^{13}C-NMR spectra of the covalent probes 35

HRMS spectra of the covalent probes 44

Uncropped SDS-PAGE gels...53

Supplementary Tables

Table S1. O-S exchange effect on NBO-charge distribution localized to the adjacent carbon atom in β-position (assessed by M062X/6-311++G(d,p) DFT calculations)

NBO charge distribution	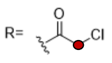	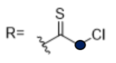	$\Delta NBO_{O \rightarrow S}$	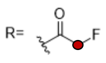	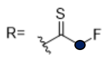	$\Delta NBO_{O \rightarrow S}$
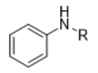	-0.454	-0.400	**+0.054**	0.003	0.029	**+0.026**
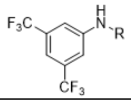	-0.459	-0.402	**+0.057**	0.002	0.031	**+0.029**
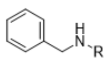	-0.437	-0.398	**+0.039**	0.007	0.034	**+0.027**
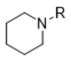	-0.461	-0.443	**+0.018**	-0.004	0.017	**+0.021**

Table S2. GSH assay and NP assay results of the covalent probes (3,6,8-11)

Entry	GSH assay			NP assay	
	k_{GSH} [1000×h^{-1}]	$t_{1/2(GSH)}$ [h]	$t_{1/2(deg)}$ [h]	Labelling conversion [%]	Labelled amino acid[a]
**3a**	29,9	23.2	>72.0	34%	C
**3b**	119,5	5.8	>72.0	33%	C
**3c**	70.0	9.9	>72.0	69%	C
**3d**	106.6	6.5	>72.0	87%	C
**4a**	N/D	>72.0	>72.0	20%	C
**4b**	13,4	51.7	>72.0	0%	-
**4c**	N/D	>72.0	>72.0	0%	-
**4d**	N/D	>72.0	>72.0	0%	-
**5a**	25,9	26.8	>72.0	21%	C
**5b**	266,6	2.6	>72.0	5%	C
**5c**	161.2	4.3	>72.0	52%	C
**5d**	277.3	2.5	>72.0	76%	C
**6a**	N/D	>72.0	>72.0	38%	C
**6b**	26,1	26.6	>72.0	20%	C
**6c**	9.8	70.9	>72.0	70%	C
**6d**	11.9	58.3	>72.0	17%	C

[a] Preferred residue in nonapeptide assay (C: cysteine). [b] The reactions faster than the minimal time window necessary to obtain LC-MS spectra are reported here with a kinetic rate constant >13863, due to the minimal running time was 3 min, which would be equal to 13.863 h^{-1} kinetic rate constant.

Table S3. Computed activation Gibbs free energies of the covalent probes (3,6,8-11)

Entry	$G_{\text{Initial State}}$ (E_{ht})	$G_{\text{Transition State}}$ (E_{ht})	$\Delta G^{\ddagger}$ ($^{kJ}/_{mol}$)	Reactivity $\ln(k_{1st})$ [-]
**3a**	-899.6961	-1337.8625	50.7133	-3.51
**3b**	-1573.7975	-2011.9630	52.9874	-2.12
**3c**	-938.974	-1377.14	55.0515	-2.66
**3d**	-863.956	-1302.12	55.3902	-2.24
**4a**	-539.3328	-977.4839	90.6968	N/A
**4b**	-1213.4343	-1651.5842	93.8532	-4.31
**4c**	-578.61	-1016.76	99.1210	N/A
**4d**	-503.593	-941.74	128.2276	N/A
**5a**	-1222.6442	-1660.8083	56.6113	-2.65
**5b**	-1896.7436	-2334.9142	39.7734	-1.32
**5c**	-1261.92	-1700.09	50.6293	-1.83
**5d**	-1186.9	-1625.07	51.6219	-1.28
**6a**	-862.2784	-1300.4306	88.0892	N/A
**6b**	-1536.3795	-1974.5329	84.8329	-3.65
**6c**	-901.563	-1339.71	101.1798	-4.63
**6d**	-826.543	-1264.68	102.8158	-4.43
**MeS^-**	-438.1857			

Table S4. Positioning of the targeted cysteines and characterization of their reactivity and accessibility based on *Cy-preds* server predictions

Protein	Kinome tree major branch	Targeted residue	Region of the targeted residue	pKa	SASA* [Å^2]
**BTK**	TK	C481	front pocket	6.1	25.4
**MAP2K6**	STE	C196	DFG	11.1	25.6
**JAK3**	TK	C909	front pocket	4.7	25.5

* cysteine sulfur atom solvent-accessible surface area

Table S5. Inhibiton effeciency of covalent fragments (3a-b, 4a-b, 5a-b, 6a-b) on BTK, JAK3 and MAP2K6 kinases, reported as individual inhibition percentage of the biological duplicates, measured at 100 µM concentration of the after 60 min preincubation.

Covalent probe	Inhibition efficiency at 100 uM [%]					
	BTK		JAK3		MAP2K6	
**3a**	62	63	73	65	11	11
**3b**	80	82	97	97	57	53
**4a**	13	13	3	-4	2	-2
**4b**	11	5	5	2	-2	-1
**5a**	89	91	49	53	96	99
**5b**	91	83	100	101	86	89
**6a**	9	16	-3	-6	-5	3
**6b**	84	82	95	94	88	92

Table S6. Intact protein MS results summary reporting % degree of labelling on kinase targets JAK3, BTK and MAP2K6. Molar stochiometries varied across the experiments, the following equivalents of the covalent probes related to the protein of interest was applied: probes 3-6 on BTK with 20 eq., on JAK3 with 170 eq. and on MAP2K6 with 70 eq; probes 9-10 on JAK3 with 13 eq., probes 11-15 on BTK with 2 eq.

Entry	BTK	JAK3*	MAP2K6
**3a**	31%	38%	43%
**3b**	94%	9%	51%
**5a**	32%	59%	79%
**5b**	52%	8%	57%
**6b**	11%	1%	85%
**9**	-	41%	-
**10**	-	33%	-
**11**	86%	-	-
**12**	93%	-	-
**13**	1%	-	-
**14**	43%	-	-
**15**	13%	-	-

* Labelling ratio on JAK3 was calculated from the measured MS1 intensities of labelled versus nonlabelled peptides (L900-R911) resulted from tryptic digesteion of JAK3.

Table S7. Annotations of MS/MS spectrum of the modified proteins after enzymatic digestion and subsequent peptide mapping

a) JAK3 [L900-R911] modification with probe **3a**.

Ion	Theoretical Peaks	Observed Peaks	Ion	Theoretical Peaks	Observed Peaks	Ion	Theoretical Peaks	Observed Peaks
$a1^+$	86.10	86.10	$b2^+$	213.16	213.16	$y12^{2+}$	757.38	757.38
$a2^+$	185.16	185.16	$b3^+$	344.20	344.20	$y11^+$	1400.67	1400.68
$a4^+$	445.25	445.25	$b4^+$	473.24	473.24	$y10^+$	1301.60	1301.62
$a5^+$	608.31	608.31	$b5^+$	636.31	636.31	$y9^+$	1170.56	1170.57
$a6^+$	721.40	721.40	$b6^+$	749.39	749.39	$y8^+$	1041.52	1041.52
			$b7^+$	846.44	846.44	$y7^+$	878.46	878.46
			$b8^+$	933.48	933.47	$y6^+$	765.37	765.37
			$b11^+$	1339.64	1339.65	$y5^+$	668.32	668.32
						$y4^+$	581.29	581.29
						$y3^+$	524.26	524.26
						$y2^+$	288.20	288.2
						$y1^+$	175.12	175.12

b) JAK3 [L900-R911] modification with probe **3b**.

Ion	Theoretical Peaks	Observed Peaks	Ion	Theoretical Peaks	Observed Peaks	Ion	Theoretical Peaks	Observed Peaks
$a1^+$	86.10	86.10	$b2^+$	213.16	213.16	$y12^{2+}$	825.37	825.37
$a2^+$	185.16	185.16	$b3^+$	344.20	344.20	$y10^+$	1437.58	1437.58
$a4^+$	445.25	445.25	$b4^+$	473.24	473.24	$y9^+$	1306.54	1306.54
$a5^+$	608.31	608.32	$b5^+$	636.31	636.31	$y8^+$	1177.49	1177.50
$a6^+$	721.40	721.40	$b6^+$	749.39	749.39	$y7^+$	1014.43	1014.44
						$y6^+$	901.35	901.35
						$y5^+$	804.29	804.28
						$y4^+$	717.26	717.26
						$y1^+$	175.12	175.12

c) JAK3 [L900-R911] modification with probe **5a**.

Ion	Theoretical Peaks	Observed Peaks	Ion	Theoretical Peaks	Observed Peaks	Ion	Theoretical Peaks	Observed Peaks
$a1^+$	86.10	86.10	$b2^+$	213.16	213.16	$y12^{2+}$	765.37	765.37
$a2^+$	185.16	185.16	$b3^+$	344.20	344.20	*y10*	1317.58	1317.59
$a4^+$	445.25	445.25	$b4^+$	473.24	473.25	*y9*	1186.54	1186.55
$a5^+$	608.31	608.31	$b5^+$	636.31	636.31	*y8*	1057.50	1057.50
$a6^+$	721.40	721.40	$b6^+$	749.39	749.39	*y7*	894.43	894.44
						y6	781.35	781.35
						y5	684.30	684.30
						y4	597.26	597.26
						y1	175.12	175.12

d) JAK3 [L900-R911] modification with probe **5b**.

Ion	Theoretical Peaks	Observed Peaks	Ion	Theoretical Peaks	Observed Peaks	Ion	Theoretical Peaks	Observed Peaks
a1^{+}	86.10	86.10	*b2*$^{+}$	213.16	213.16	*y12*$^{2+}$	833.36	833.38
a2^{+}	185.16	185.16	*b3*$^{+}$	344.20	344.20	*y10*$^{+}$	1453.55	1453.60
a4^{+}	445.25	445.25	*b4*$^{+}$	473.24	473.25	*y9*$^{+}$	1322.51	1322.57
a5^{+}	608.31	608.31	*b5*$^{+}$	636.31	636.31	*y8*$^{+}$	1193.47	1193.52
a6^{+}	721.40	721.39	*b6*$^{+}$	749.39	749.39	*y7*$^{+}$	1030.41	1030.45
						y6^{+}	917.32	917.37
						y5^{+}	820.27	820.32
						y4^{+}	733.24	733.25
						y1^{+}	175.12	175.12

e) JAK3 [L900-R911] modification with probe **6b**.

Ion	Theoretical Peaks	Observed Peaks	Ion	Theoretical Peaks	Observed Peaks	Ion	Theoretical Peaks	Observed Peaks
a1^{+}	86.10	86.10	*b2*$^{+}$	213.16	213.16	*y12*$^{2+}$	833.36	833.38
a2^{+}	185.16	185.16	*b3*$^{+}$	344.20	344.20	*y10*$^{+}$	1453.55	1453.60
a4^{+}	445.25	445.25	*b4*$^{+}$	473.24	473.25	*y9*$^{+}$	1322.51	1322.57
a5^{+}	608.31	608.31	*b5*$^{+}$	636.31	636.31	*y8*$^{+}$	1193.47	1193.52
a6^{+}	721.40	721.39	*b6*$^{+}$	749.39	749.39	*y7*$^{+}$	1030.41	1030.46
						y6^{+}	917.32	917.37
						y5^{+}	820.27	820.32
						y1^{+}	175.12	175.12

Table S8. QM/MM-based calculation results of BTK interactions with probe 12, 14

a) Results of thermodynamic integration mutating chloroacetamide to chlorothioacetamide ligand (**12** to **14**) in water and bound to BTK in water. All data are in kcal/mol**.**

	Cys481 thiol				Cys481 thiolate			
	Calc 1	*Calc 2*	*Calc 3*	*Mean*	*Calc 1*	*Calc 2*	*Calc 3*	*Mean*
**ligand(O->S)**	3.18	3.44	3.85		2.93	3.45	2.82	
**complex(O->S)**	3.97	4.63	3.65		3.83	3.56	4.53	
**$\Delta\Delta$G(O->S)**	0.79	1.19	-0.19	0.60	0.90	0.11	1.71	0.75

b) QM/MM calculated k_{inact}/K_I values compared to the experimental results.

	experimental	calculated
**K_I[14] / K_I[12]**	10.1	3.5
**k_{inact}[14] /k_{inact}[12]**	28	111
**k_{inact} /K_I[14] / k_{inact} /K_I[12]**	2.7	31

K_I[**14**]/K_I[**12**] was calculated as exp($\Delta\Delta$G/RT) with $\Delta\Delta$G=0.80, the mean of the six evaluations in Table S8a and RT=0.6 kcal/mol.
k_{inact}[**14**]/k_{inact}[**12**] was calculated as exp(($G^{\#}$[12]- $G^{\#}$[14])/RT) with $G^{\#}$[**12**]=17.8 kcal/mol and $G^{\#}$[**14**]= 15.0 kcal/mol (see Figure S6d).

Table S9. UV-FAR analysis results of 19-22 conjugated antibodies by biological replicates

Conjugation agent	Measured UV-FAR				
	5min	10min	30min	60min	90min
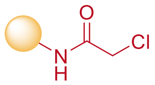 **19**	0.22	1.08	2.22	3.82	4.19
	0.46	0.59	2.74	4.06	3.88
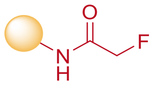 **20**	-0.09	0.22	1.19	1.54	2.39
	0.22	0.45	0.99	2.16	1.64
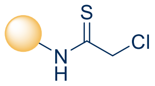 **21**	0.95	2.02	3.59	3.48	4.37
	0.73	2.88	4.11	4.31	3.99
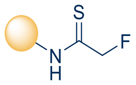 **22**	0.06	0.74	1.47	2.13	3.67
	0.59	0.37	1.26	2.96	2.99

Supplementary Figures

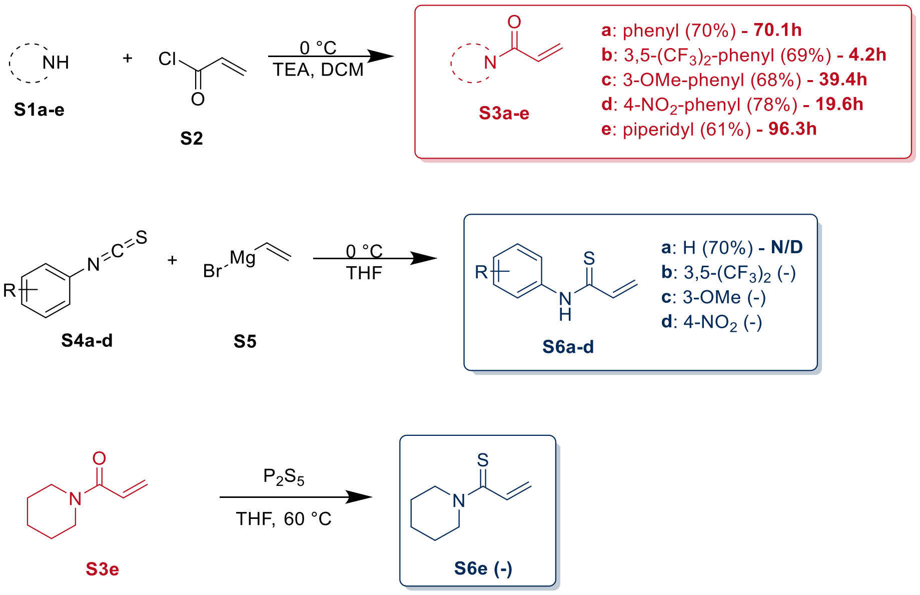

Figure S1. Results summary of acrylamide and thioacrylamide covalent fragments

Acrylamides (**S3a-e**) were obtained by *N*-acylation of anilines/piperidin (1a-g) with the corresponding acyl chloride (**S2**). Isothiocyanates (**S4a-d**) were reacted with vinylmagnesium bromide (**S5**) Grignard-reagent, however, not all of the thioacrylamides could be isolated due to their rapid degradation during purification. Nevertheless, pure **S6a** could be obtained in good yields. The results of GSH-assay shown in the figure as half-life time in hours, however the thioacrylamide molecular pairs could be measured only in case of **S6a** probe, which was found to be hyperreactive (100% reaction with 5mM GSH was achived within 3 minutes). As any further attempts to prepare thioacrylamides (**S6b-e**) were unsuccessful due to the extremely high reactivity and the limited stability of these compounds, we finally decided to remove all thioacrylic and acrylic probes from the study and instead focus exclusively on the haloacetamide and halothioacetamide series.

MS spectra of Nonapeptide (NP)

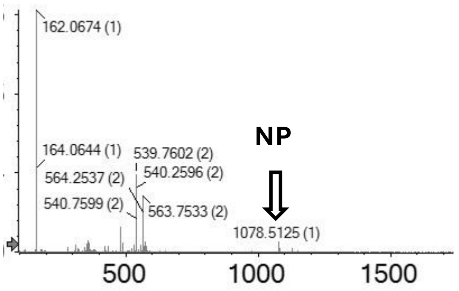

General scheme of peptide fragmentation nomenclature

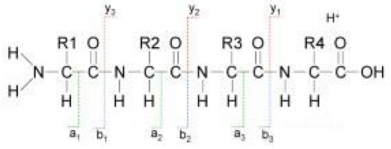

MS spectra of Nonapeptide labelled with probe 3c (NP+125Da)

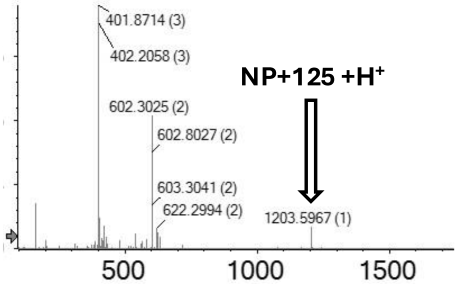

MS2 spectra of 602.3 m/z precursor ion (NP+125 +2H^{+}) with a/b/y annotations highlighted in red

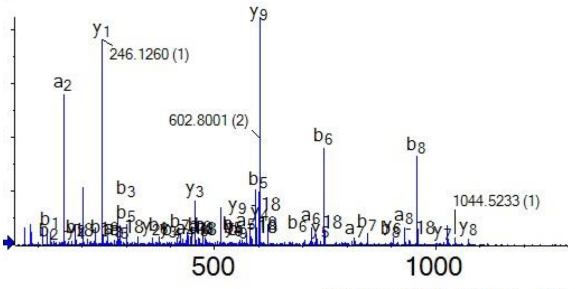

# (N)	a	b	b - 18	y	y - 18	# (C)
1	101.10732	129.10224	111.09167	1203.59793	1185.58736	9
2	158.12879	186.12370	168.11314	1075.50296	1057.49240	8
3	273.15573	301.15065	283.14008	1018.48150	1000.47094	7
4	436.21906	464.21398	446.20341	903.45456	885.44399	6
5	573.27797	601.27289	583.26232	740.39123	722.38066	5
6	720.34639	748.34130	730.33074	603.33232	585.32175	4
7	817.39915	845.39407	827.38350	456.26390	438.25334	3
8	930.48321	958.47813	940.46756	359.21114	341.20057	2
9	1157.59245	1185.58736	1167.57680	246.12708	228.11651	1

Chemical structure of the representative covalent probe (3c)

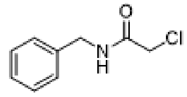

Figure S2. The MSMS spectrum of the modified nonapeptide (NP) in selectivity assay

The identification of labeled amino acids in nonapeptide assay was performed using standard LC–MS/MS analysis. As the oligopeptide is already compatible with downstream MS analysis, the labeled peptide was directly detected, and the position of the modification was confirmed by the MS/MS fragmentation pattern with defined cleavage sites, including a/b/c and x/y/z ion series. Labeled peptides were detected as precursor (mother) ions, and their fragmentation patterns were analyzed to determine the exact position of the modification. Differences in the m/z ratios of fragment ions between labeled and unlabeled peptides allowed unambiguous identification of the labeling site. Thus, the position of the covalent modification was confirmed by the MS/MS fragmentation pattern. To illustrate this, we prepared a representative figure (a), showing the labeled peptide with the fragmentation pattern annotated to highlight the a, b and y ions that differ from the unlabeled series at the modification site. Additionally, we have added the MS/MS spectra of all covalent probes reacted with the nonapeptide surrogate in the supplementary material. These spectra demonstrate the unambiguous assignment of the labeling sites. This approach is consistent with standard practices in peptide mapping and chemical proteomics studies. All recorded MS/MS spectra are provided in Supplementary Data 2.

a)

b)

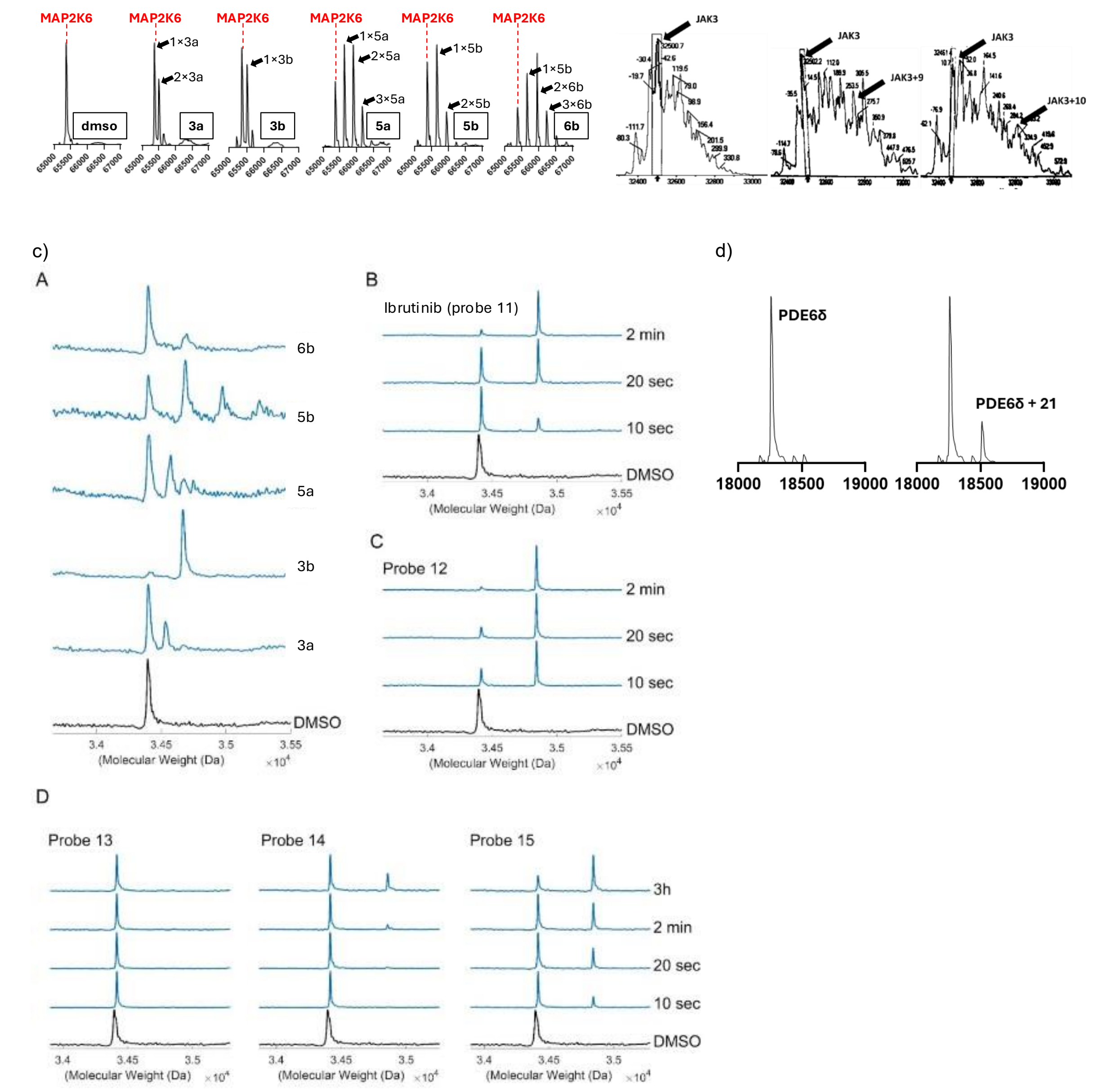

Figure S3. Intact protein MS spectrum of the proteins modified by covalent probes.
a) MAP2K6 kinase domain with covalent probes (3-6) b) JAK3 kinase domain with covalent probes (9-10), c) BTK kinase domain with covalent probes (3-6, 11-15): A) Incubation with small fragments. His_6-BTK (1 μM) was incubated with 20 μM molecule for 20 hours at 4°C. B-D) Ibrutinib derived molecules were incubated at 2 μM with His_6-BTK (1 μM) on ice for different times before stopping the reaction. Ibrutinib and Probe 12 reacted very rapidly (B-C). completing the reaction within minutes. Fragments 13-15 reacted much more slowly. or not at all (D). d) PDE6δ with covalent probe **21**

JAK3 [L900-R911] modification with probe **3a**

JAK3 [L900-R911] modification with probe **3b**

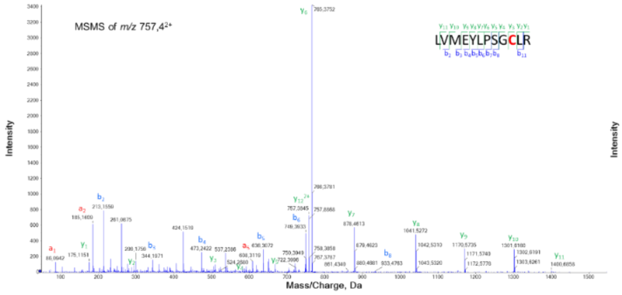

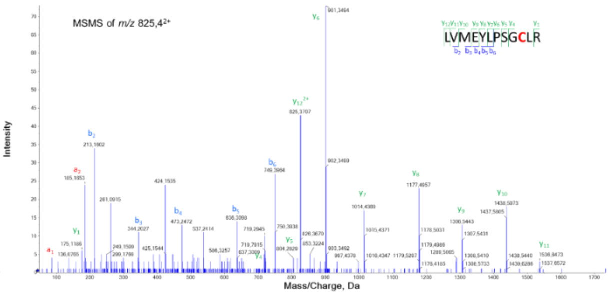

JAK3 [L900-R911] modification with probe **5a**

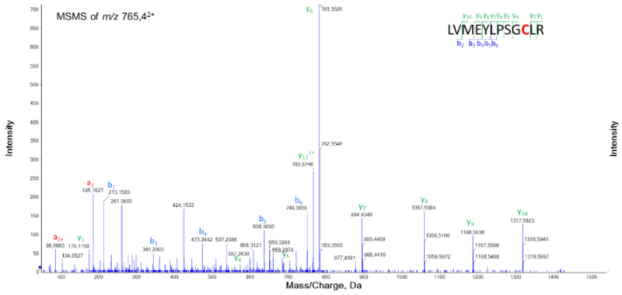

JAK3 [L900-R911] modification with probe **5b**

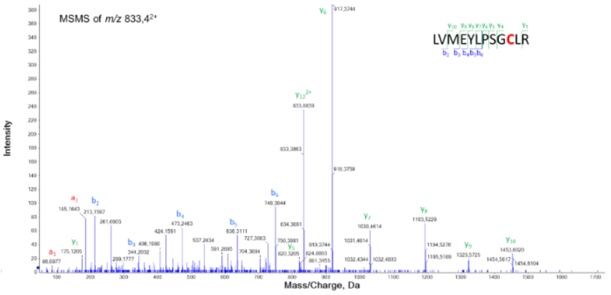

JAK3 [L900-R911] modification with probe **6b**

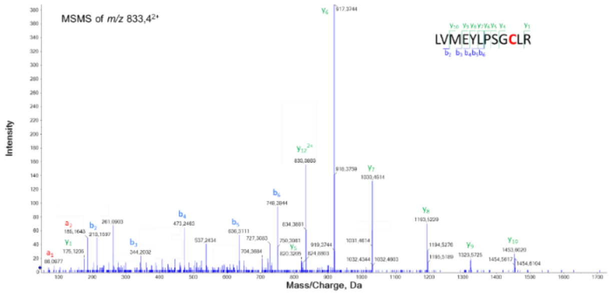

PDE6δ [H43-E62] modification with probe **21**

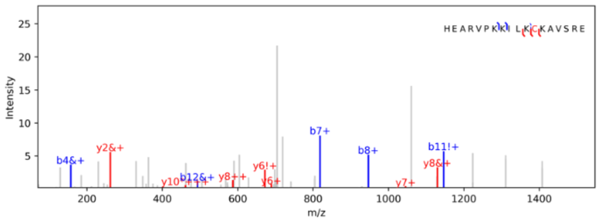

Figure S4. The MSMS spectrum of the modified proteins after enzymatic digestion and subsequent peptide mapping.

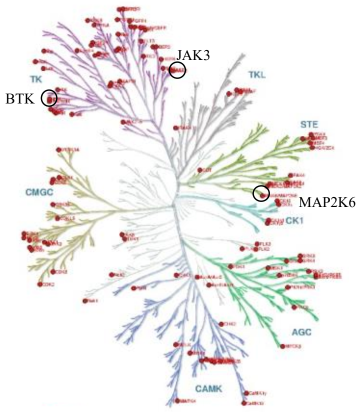

Figure S5. Investigated kinases localized on the visualized phylogenetic kinase tree

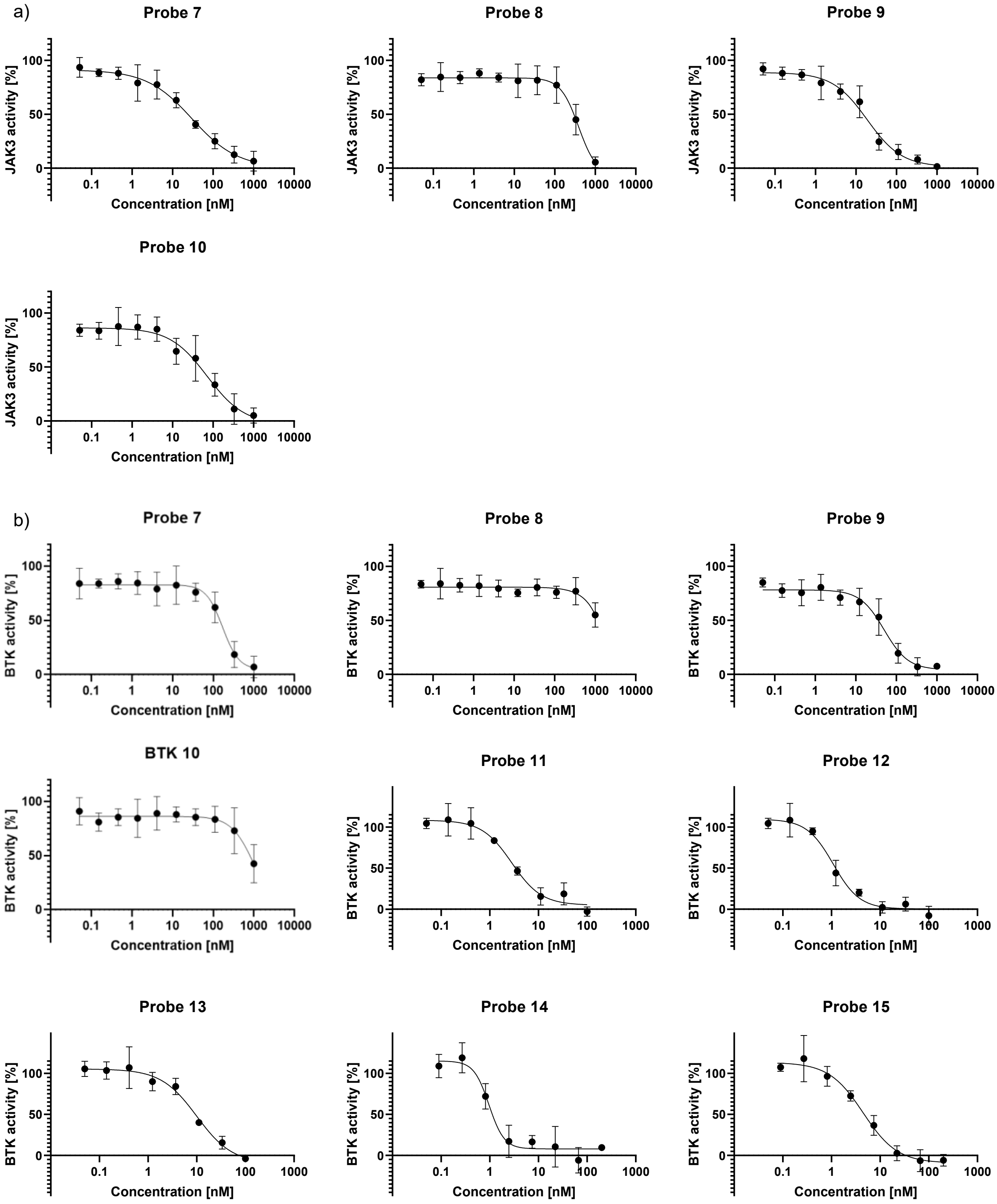

Figure S6. Dose-response curves of kinase inhibitor probes on BTK and JAK3. a) Dose-response curves of kinase inhibitor probes on JAK3 b) Dose-response curves of kinase inhibitor probes on BTK

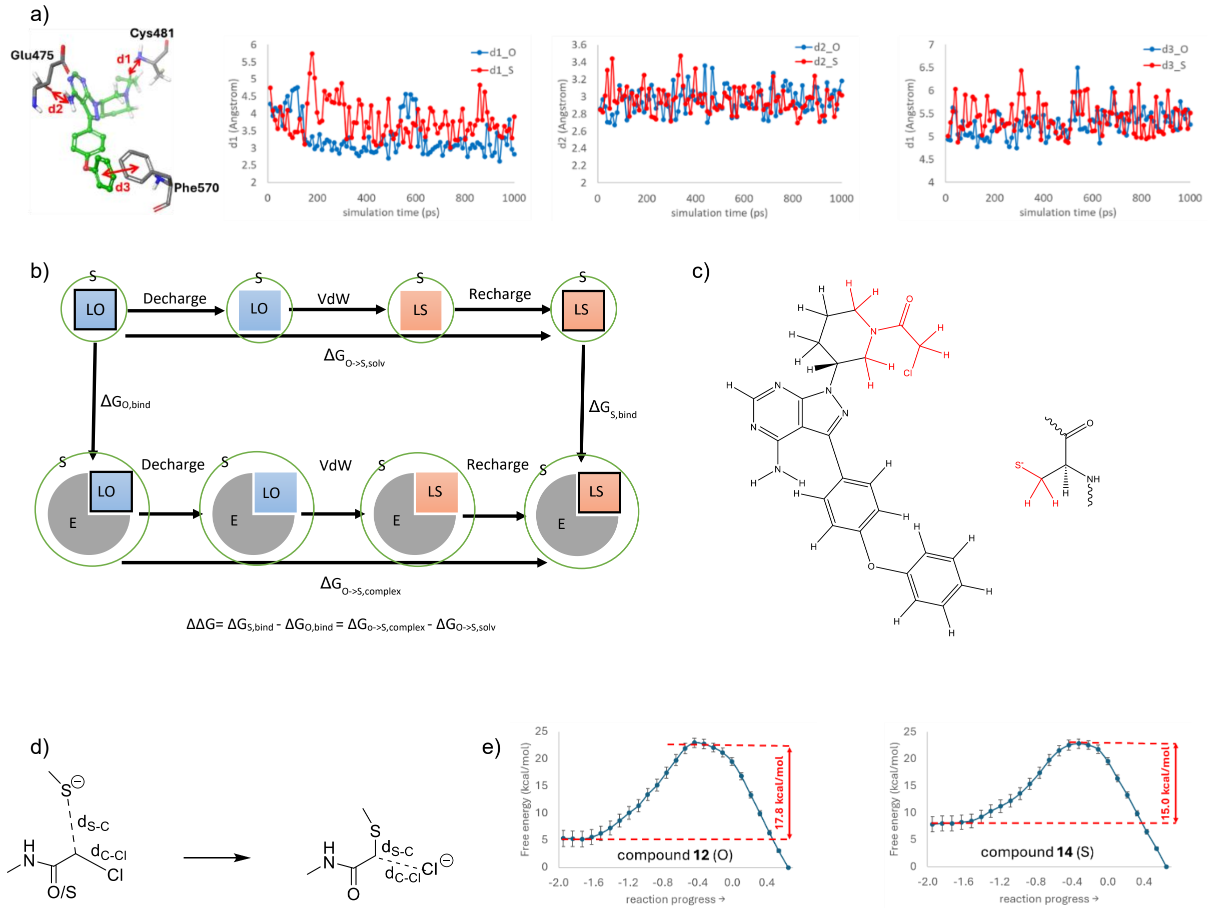

Figure S7. QM/MM-based calculation results of BTK interactions with probe 12, 14
a) α-Chloroacetamide (**12**) and α-chlorothioacetamide (**14**) binding modes to BTK. The highly similar binding modes and subsequent Cys481 covalent labeling are supported by the same key interactions between BTK and both the **12** and **14** probes. These interactions are between Cys481 N and ligand 12(O)/14(S) (d1), between Glu475 backbone carbonyl and ligand NH_2, and between the centroids of the aromatic rings of Phe570 and the ligand (d3). The three distances (d1, d2, d3) as functions of molecular dynamics simulation times are shown. b) Thermodynamic cycle of ligand mutations (LO, LS: ligands; E: enzyme; S: solvent; ligands with charged softcore atoms are framed) c) Separation of the QM and MM systems. QM atoms are shown in red and MM atoms in black. All other atoms =(protein and water) not shown are part of the MM system. d) Reaction coordinate is defined as the difference between the broken (C-Cl) and forming (S-C) bond lengths. e) Free energy profiles for the reactions of compounds **12** and **14** with BTK. The starting structures of each window (28-28 structures for complexes with **12** and **14**) are presented as Source Data.

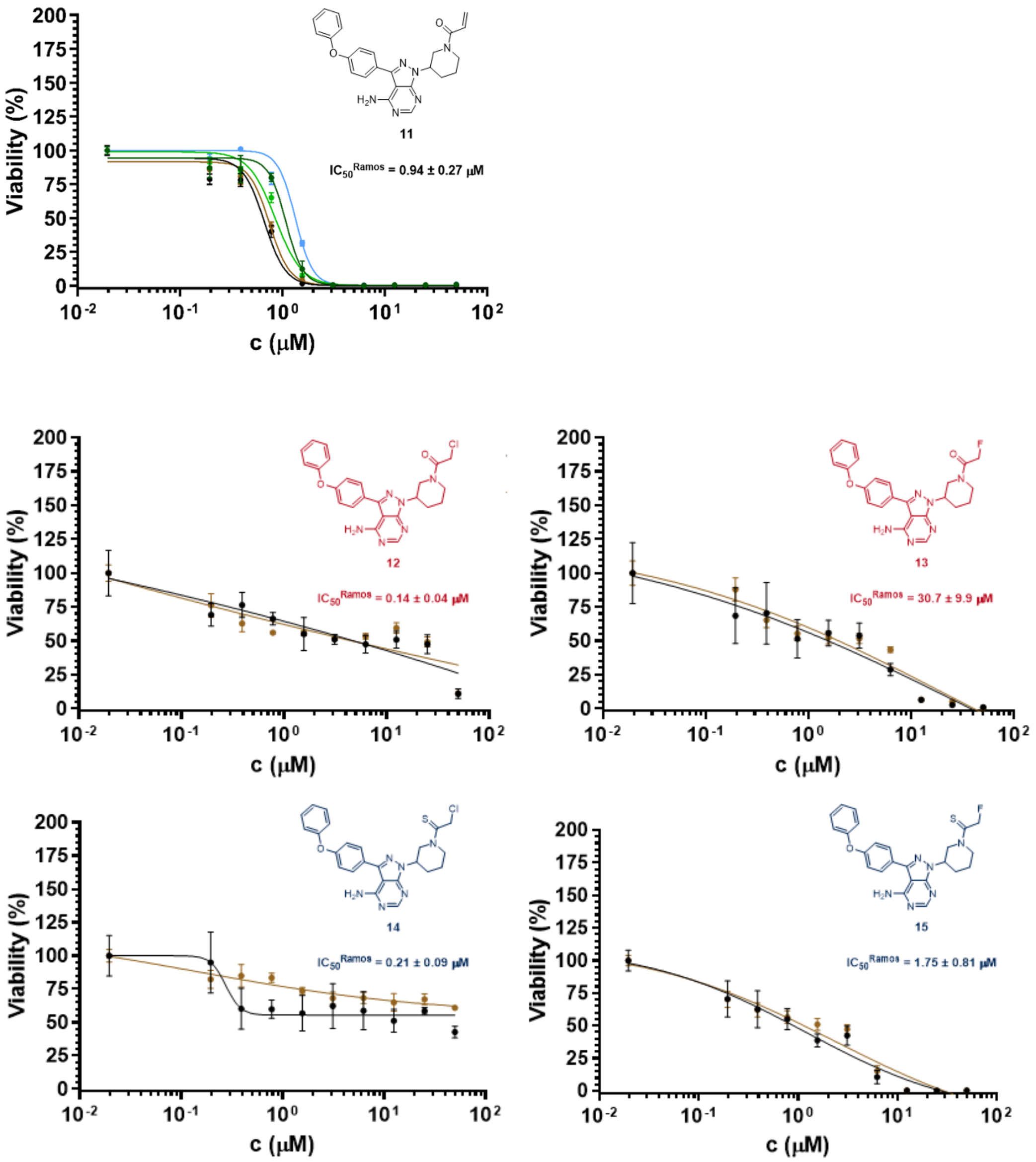

Figure S8. Dose-response curves of kinase inhibitor probes cell viablitiy effect on Ramos cells.

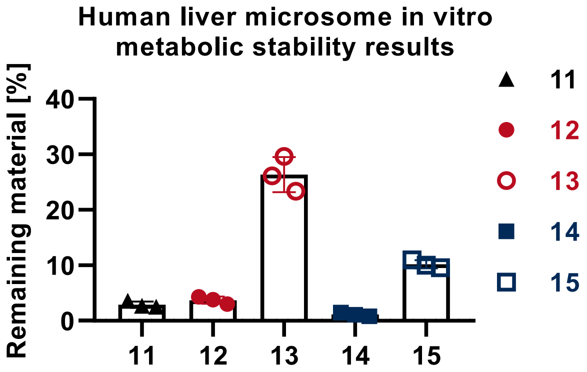

Figure S9. In vitro metabolic stability of compounds 11-15 in human liver microsomes. Compounds (10 µM) were incubated with human liver microsome fractions (1.25 mg/mL) in the presence of NADPH, and the remaining parent compound was quantified by LC–MS/MS at 0 and 30 min. Data are shown as percentage of compound remaining relative to time zero (mean of triplicate measurements with SD).

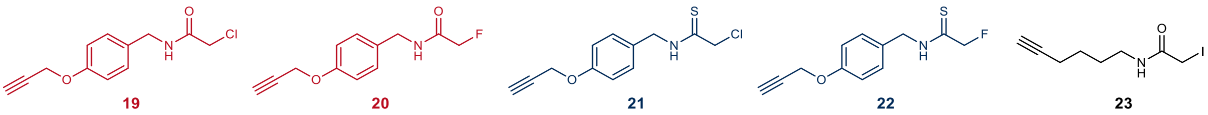

Figure S10. Structure of the applied alkyne probes (19-23)

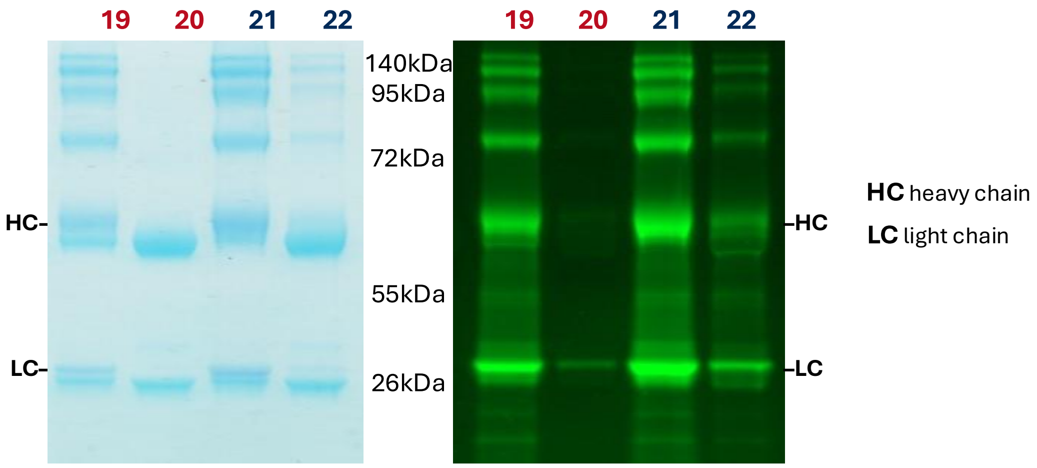

Figure S11. SDS-PAGE gel image of in-gel visualization of fluorescent dye-antibody conjugates (produced by probes 19-22), showing heavy chain (HC) and light chain (LC) captured by both, coomassie brilliant blue staining (left) and fluorescence readout (right).

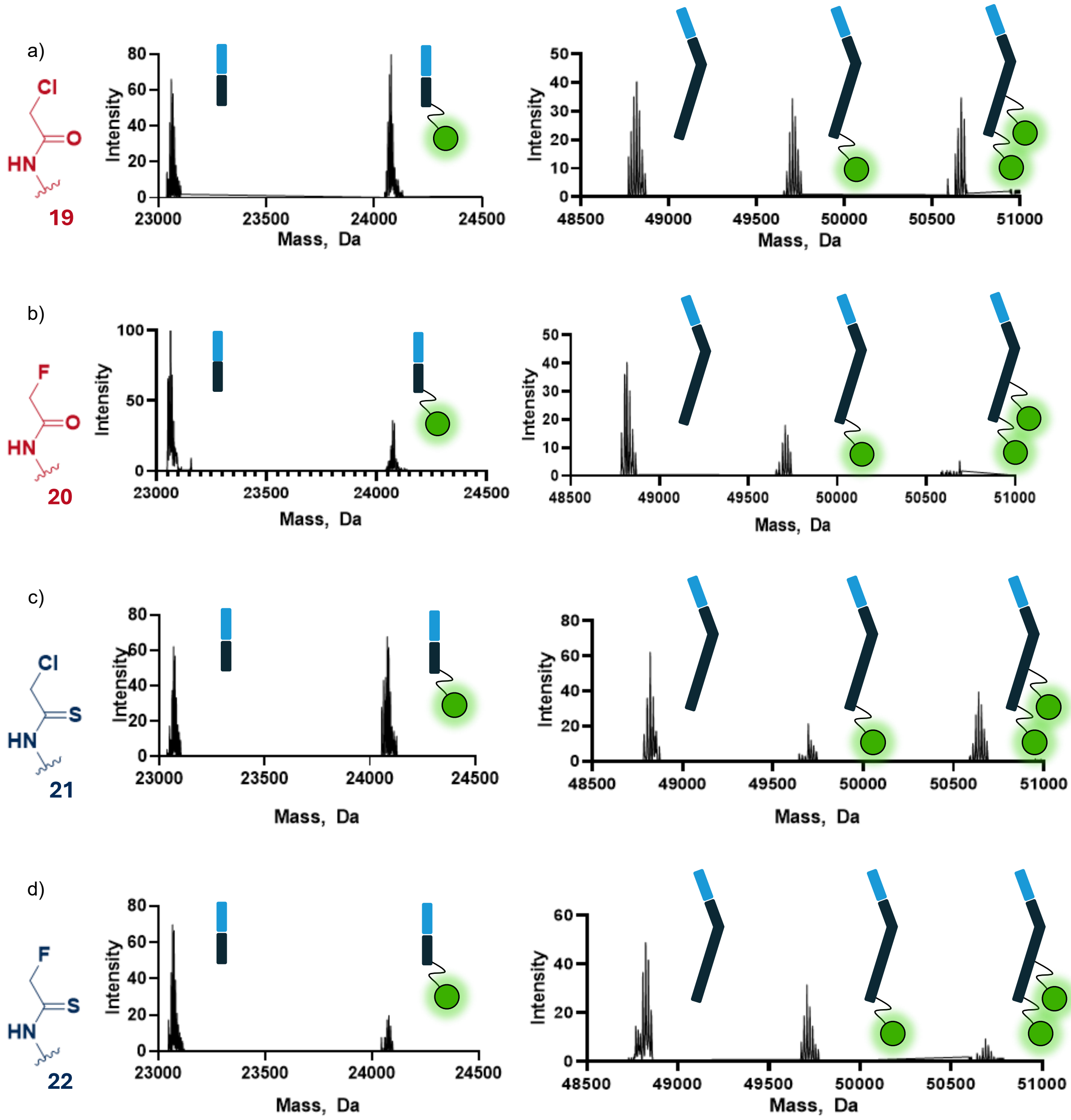

Figure S12. Covalent modification of the heavy and light chains treated with the alkyne probes (19-22) are shown on deconvoluted MS spectrum a) Trastuzumab-TAMRA conjugate obtained *via* attachment of probe **19**. b) Trastuzumab-TAMRA conjugate obtained *via* attachment of probe **20**. c) Trastuzumab-TAMRA conjugate obtained *via* attachment of probe **21.** d) Trastuzumab-TAMRA conjugate obtained *via* attachment of probe **22**:

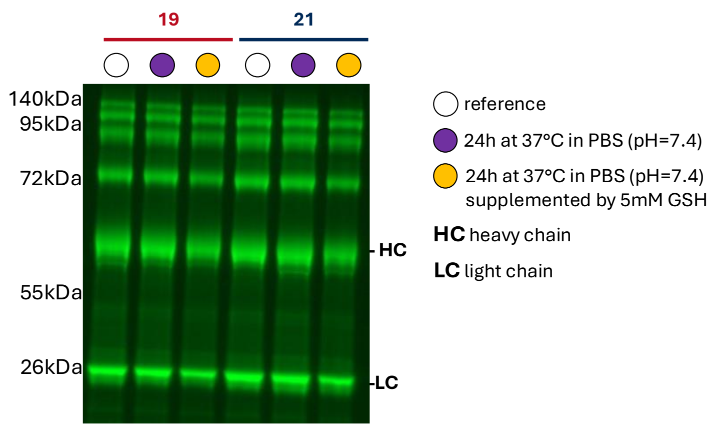

Figure S13. SDS-PAGE gel image of stability measurements on SDS-PAGE of the antibody-TAMRA conjugates.

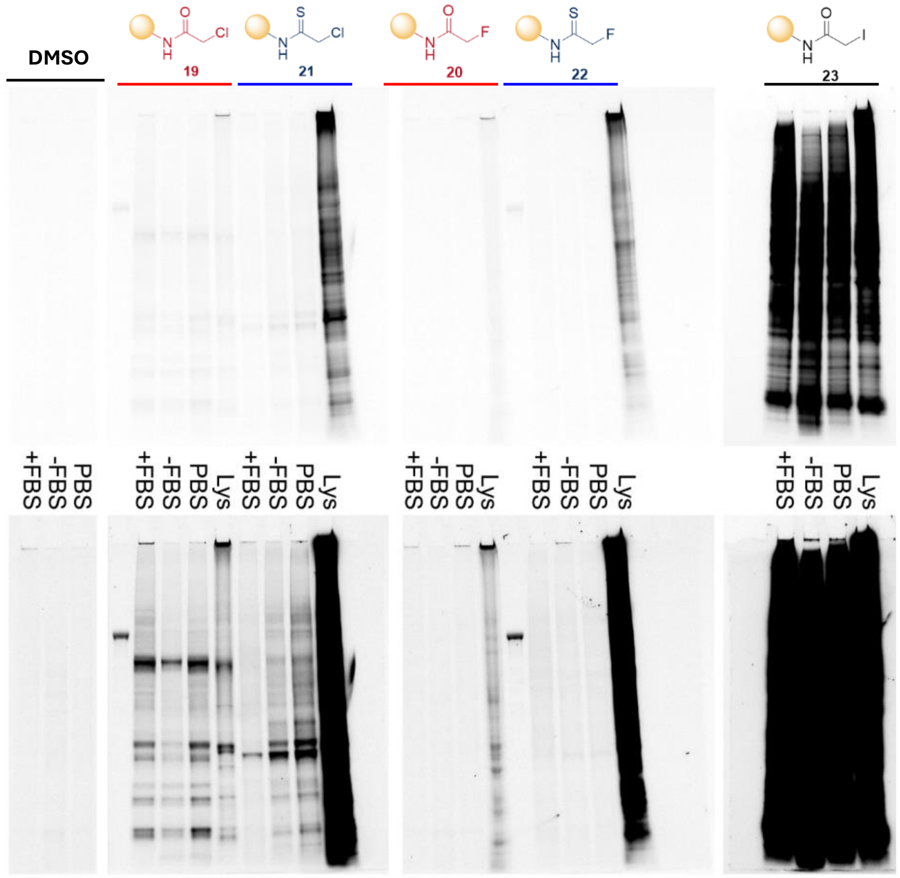

Figure S14. In-gel proteomics results with 19-23 alkyne probes. 10 µM compounds were incubated in the following conditions: HEK293 cells in the presence of media + FBS. HEK293 cells in media without FBS. HEK293 cells in PBS. or lysates of HEK293 cells in RIPA buffer. Labeled lysates were clicked to TAMRA azides and analyzed using SDS-PAGE. All gels were imaged together to ensure identical exposure times. Images were taken at short (10 sec) exposure.

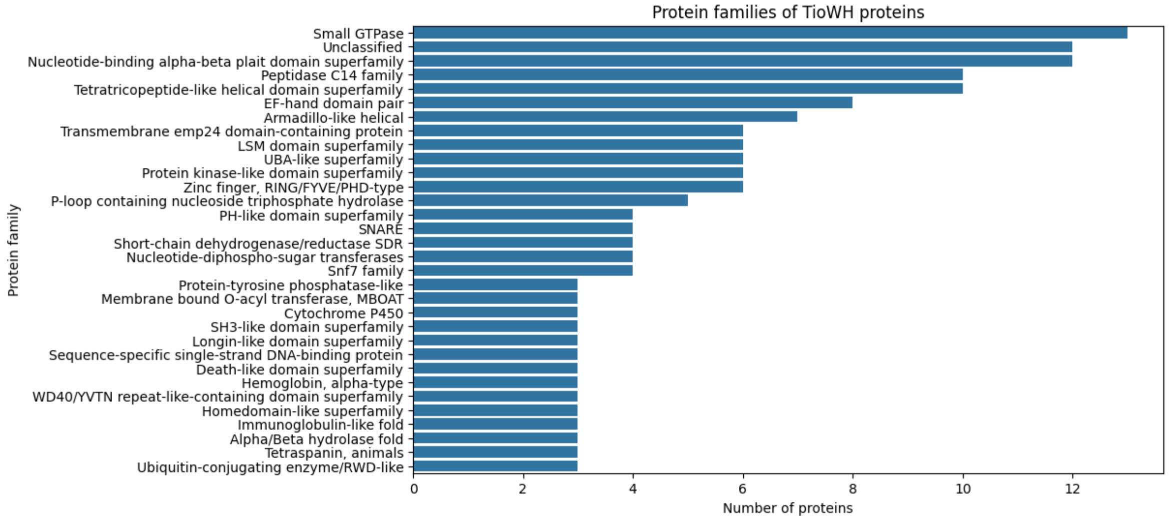

Figure S15. Functional clustering of proteins exclusively labelled by 21

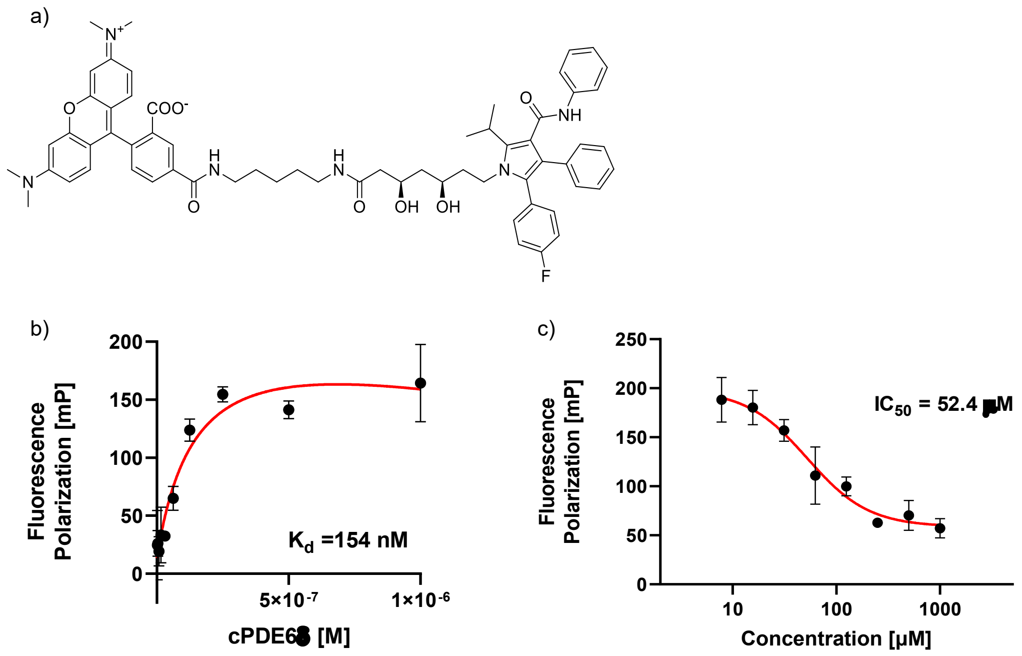

Figure S16. Fluorescent polarization assay to evaluate PDE6δ binder (21) a) Structure of the TAMRA-atorvastatin(24) fluorescent tracer; b) K_d-determination of TAMRA-atorvastatin on PDE6δ and c) Fluorescence plarization measurment of probe **21**

^{1}H-NMR spectra of the covalent probes

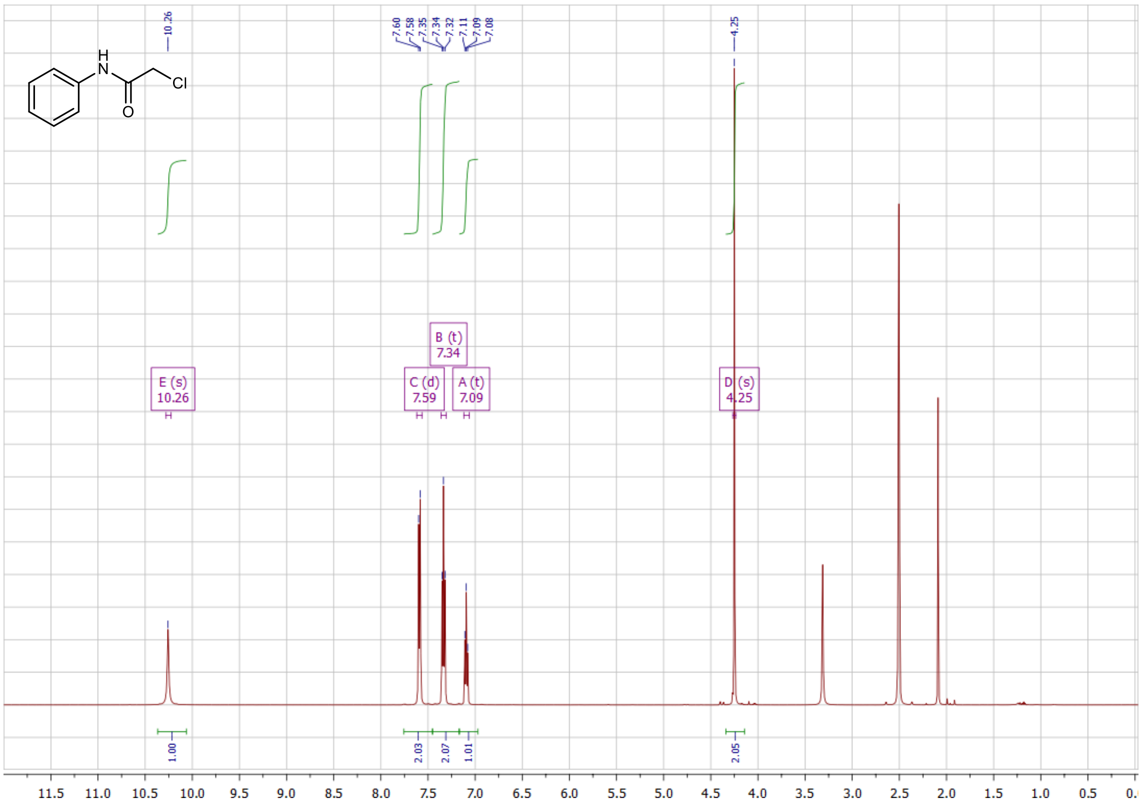

Figure S17. 2-chloroacetanilide (**3a**, DMSO-d_6, 500MHz)

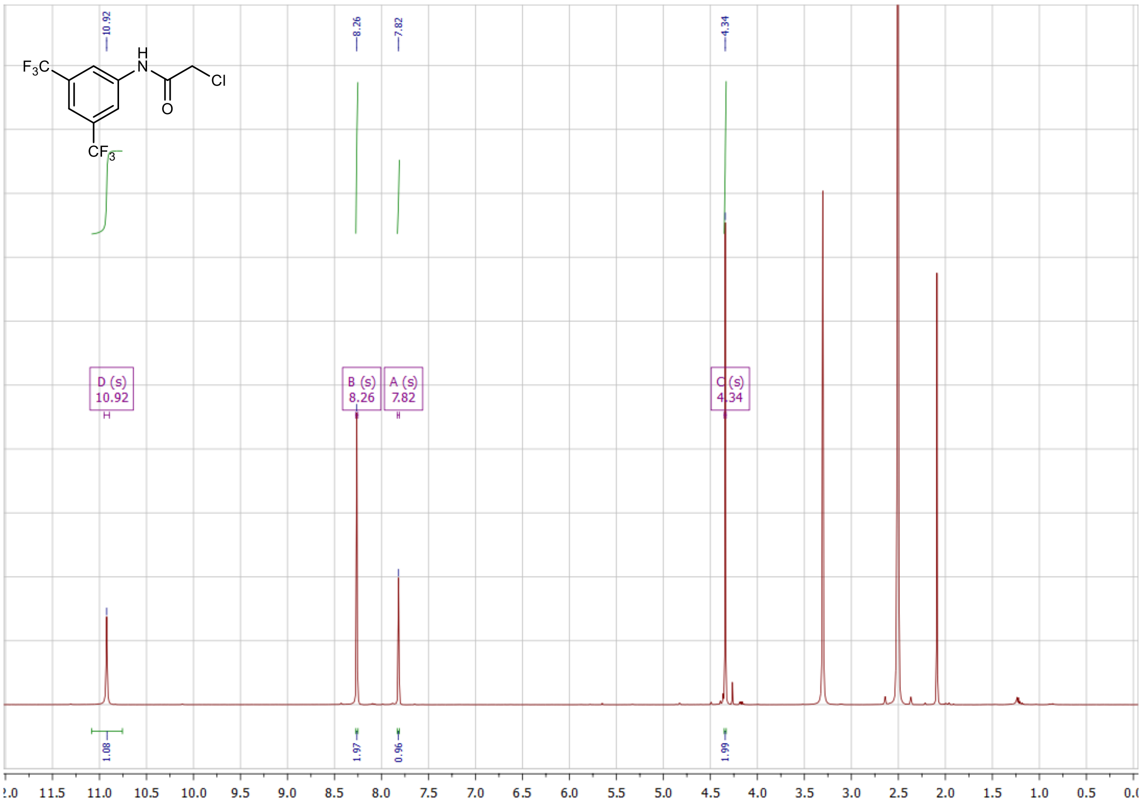

Figure S18. N-(3,5-bis(trifluoromethyl))-2-chloroacetamide (**3b**, DMSO-d_6, 500MHz)

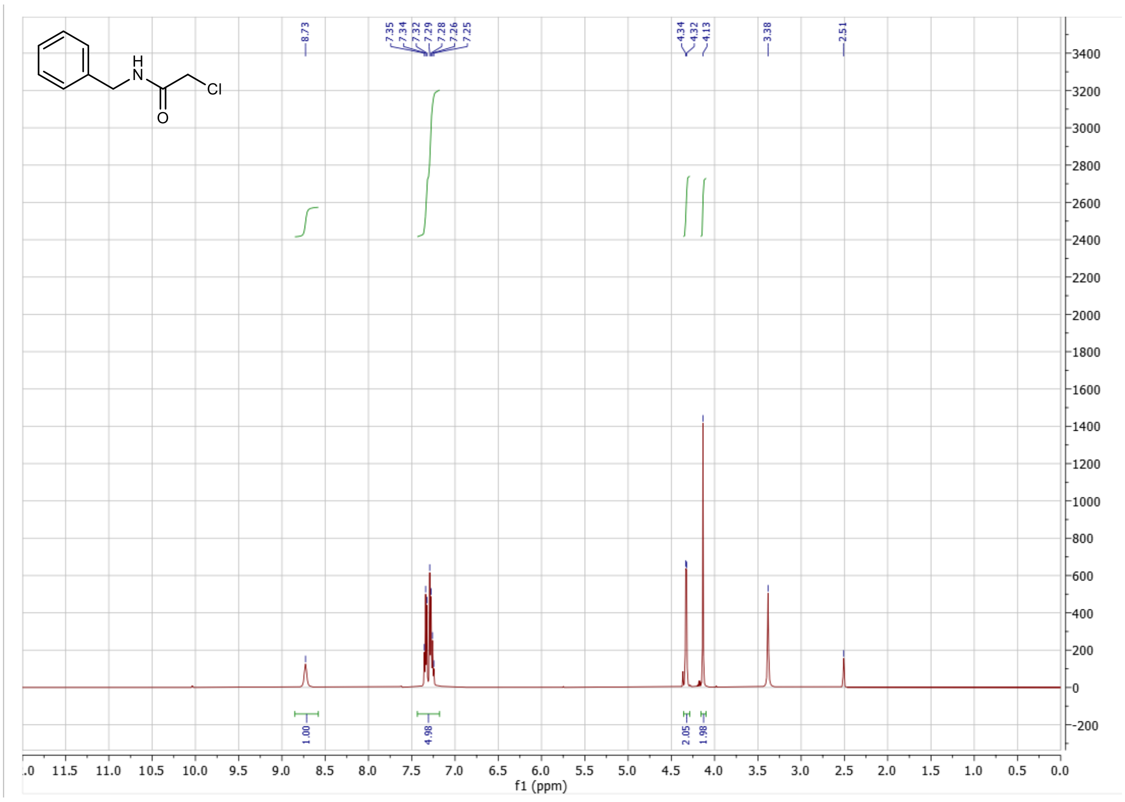

Figure S19. N-benzyl-2-chloroacetamide (**3c**, DMSO-d_6, 500MHz)

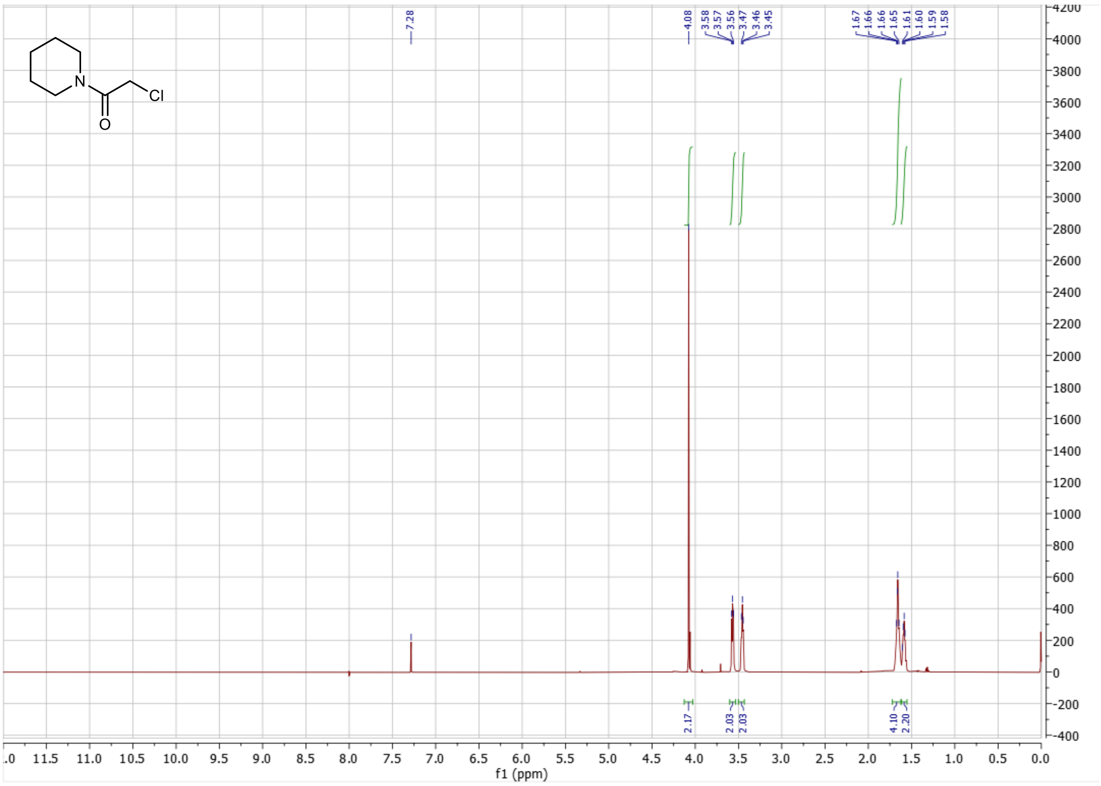

Figure S20. 2-chloro-1-(piperidin-1-yl)ethanone (**3d**, $CDCl_3$, 500MHz)

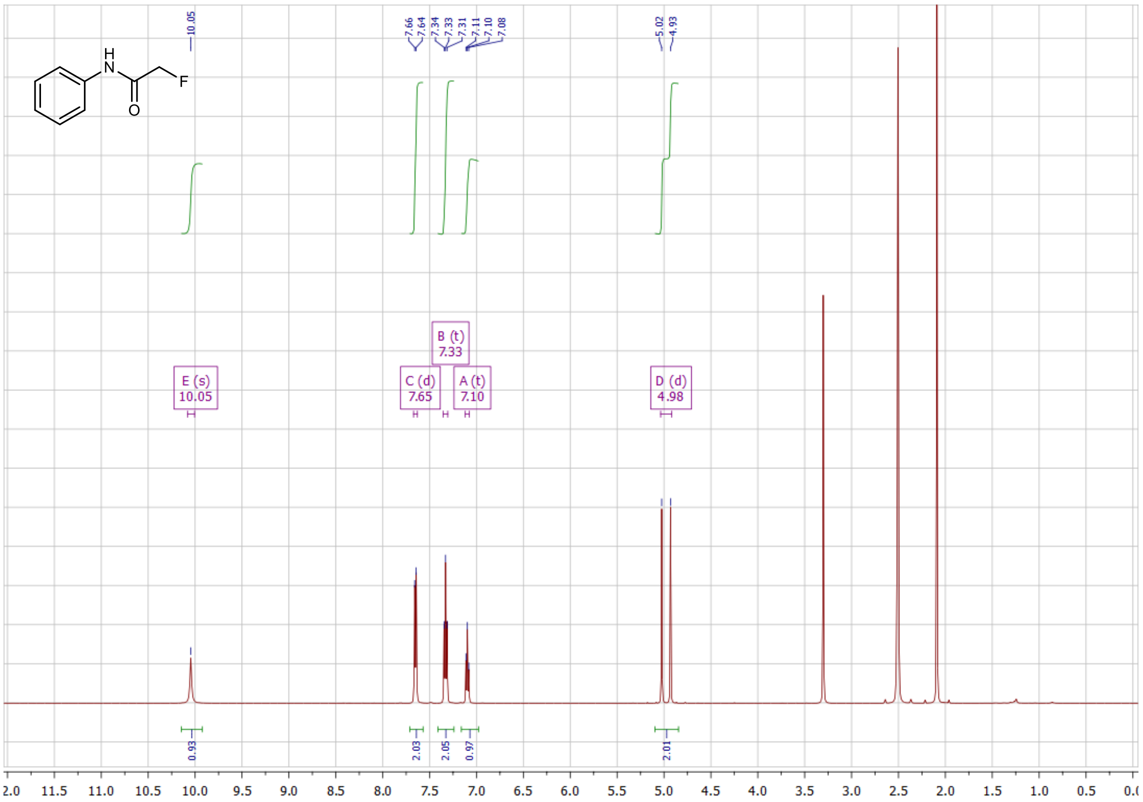

Figure S21. N-phenyl-2-fluoroacetamide *(***4a**, DMSO-*d*$_6$, 500MHz)

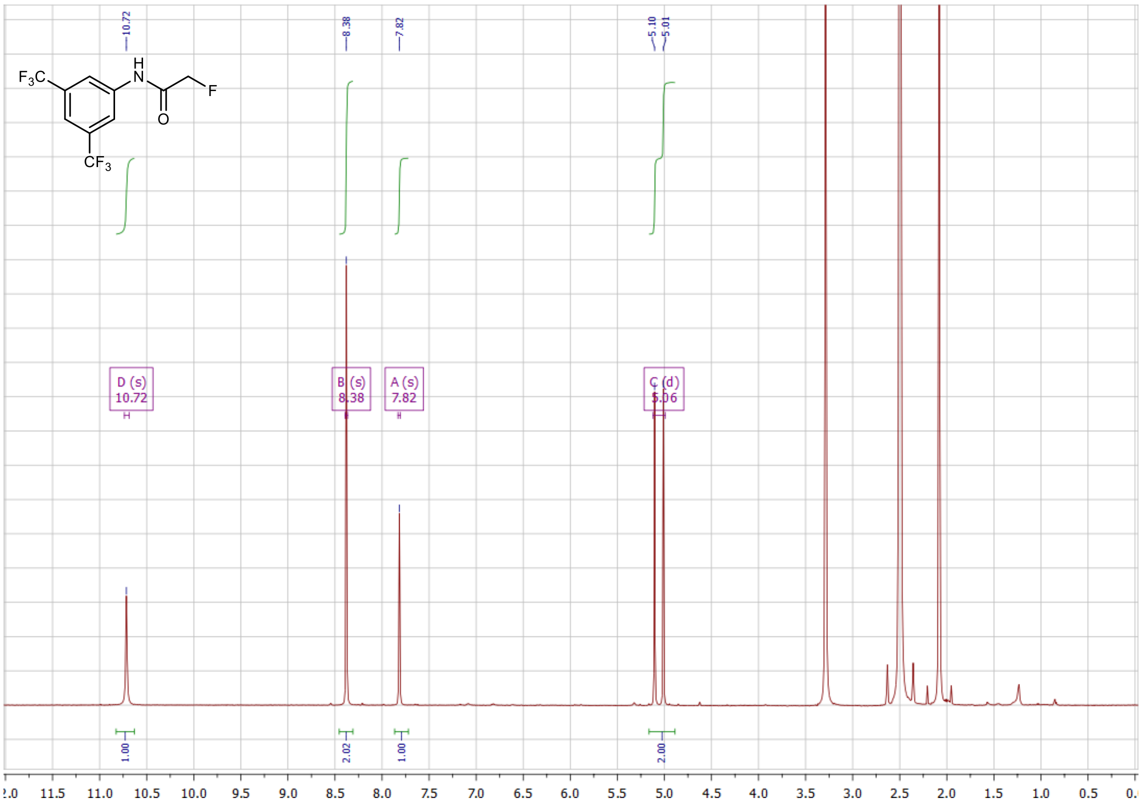

Figure S22. N-(3,5-bis(trifluoromethyl)phenyl)-2-fluoroacetamide *(***4b**, DMSO-*d*$_6$, 500MHz)

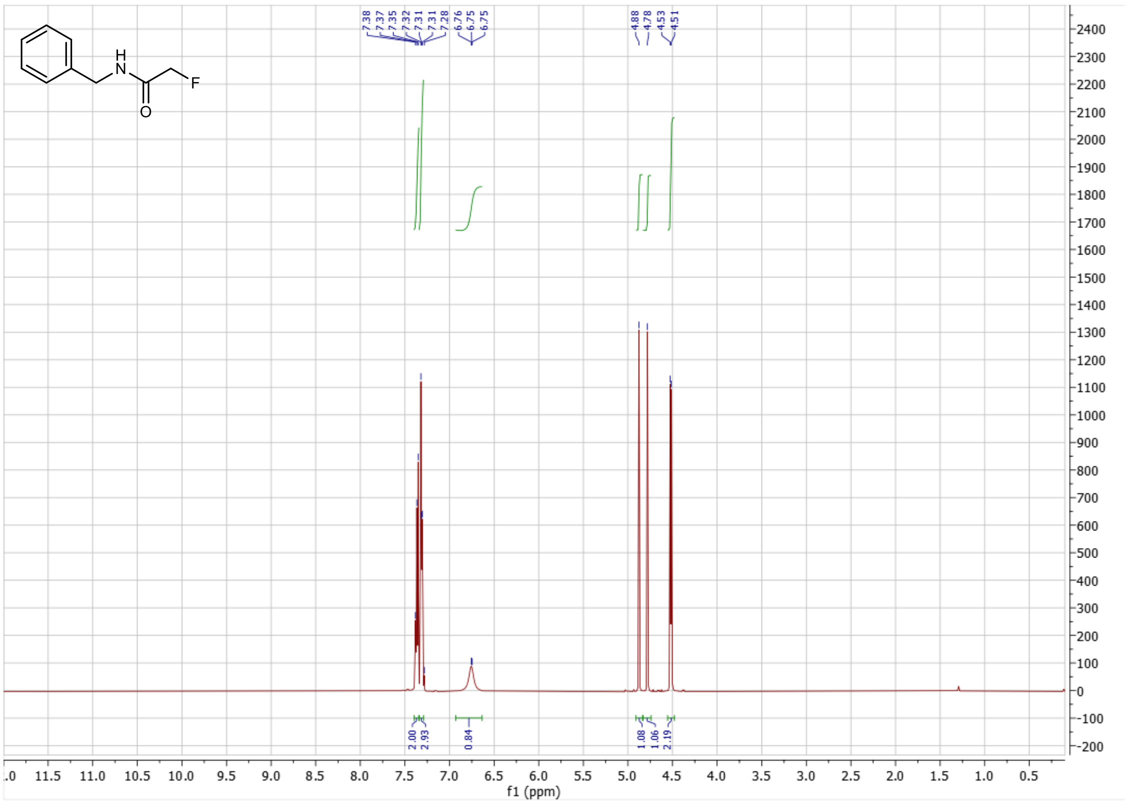

Figure S23. N-benzyl-2-fluoroacetamide (**4c**, $CDCl_3$, 500MHz)

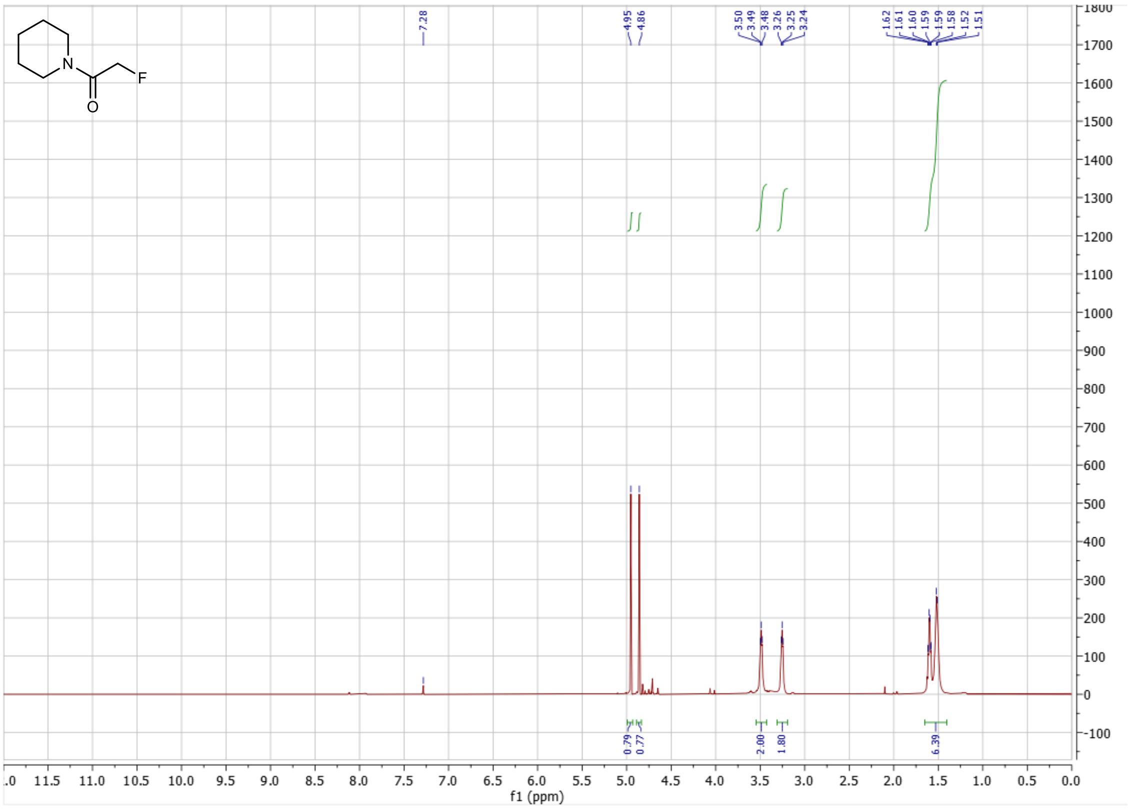

Figure S24. 2-fluoro-1-(piperidin-1-yl)ethanone (**4d**, $CDCl_3$, 500MHz)

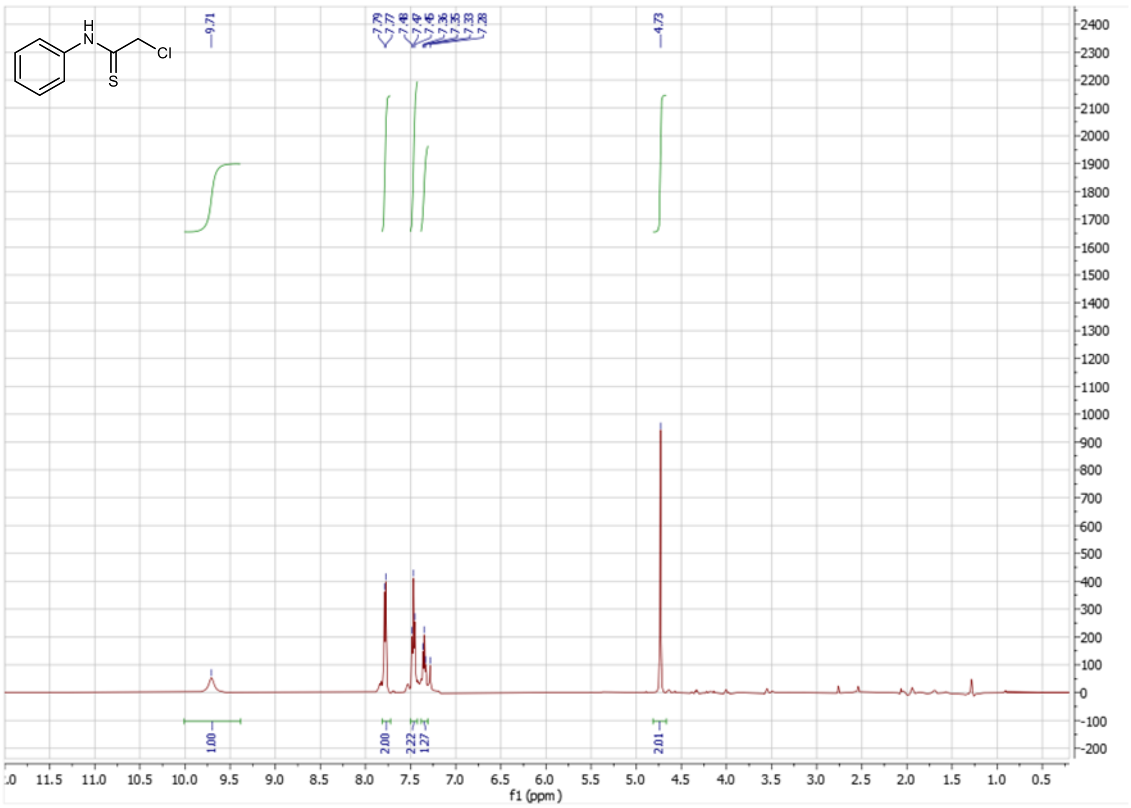

Figure S25. N-phenyl-2-chlorothioacetamide *(***5a**, $CDCl_3$, 500MHz)

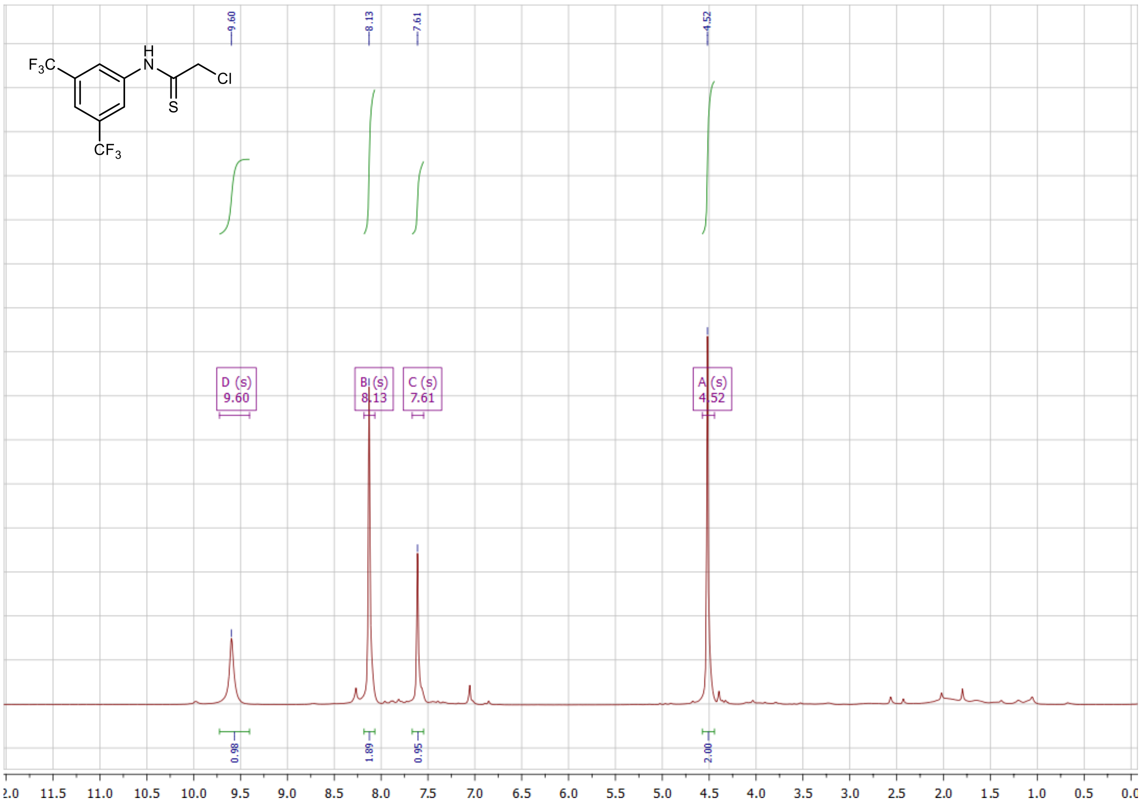

Figure S26. N-(3,5-bis(trifluoromethyl)phenyl)-2-chlorothioacetamide *(***5b**, DMSO-d_6, 500MHz)

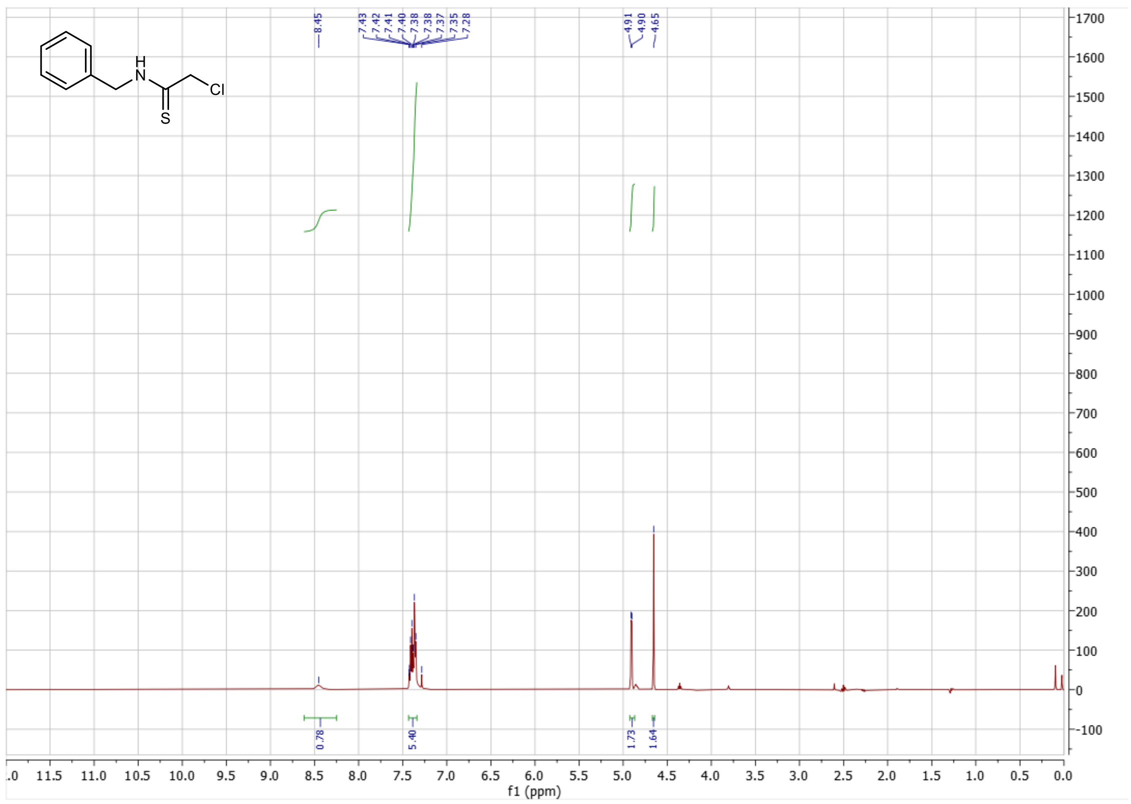

Figure S27. N-benzyl-2-chlorothioacetamide *(***5c**, $CDCl_3$, 500MHz)

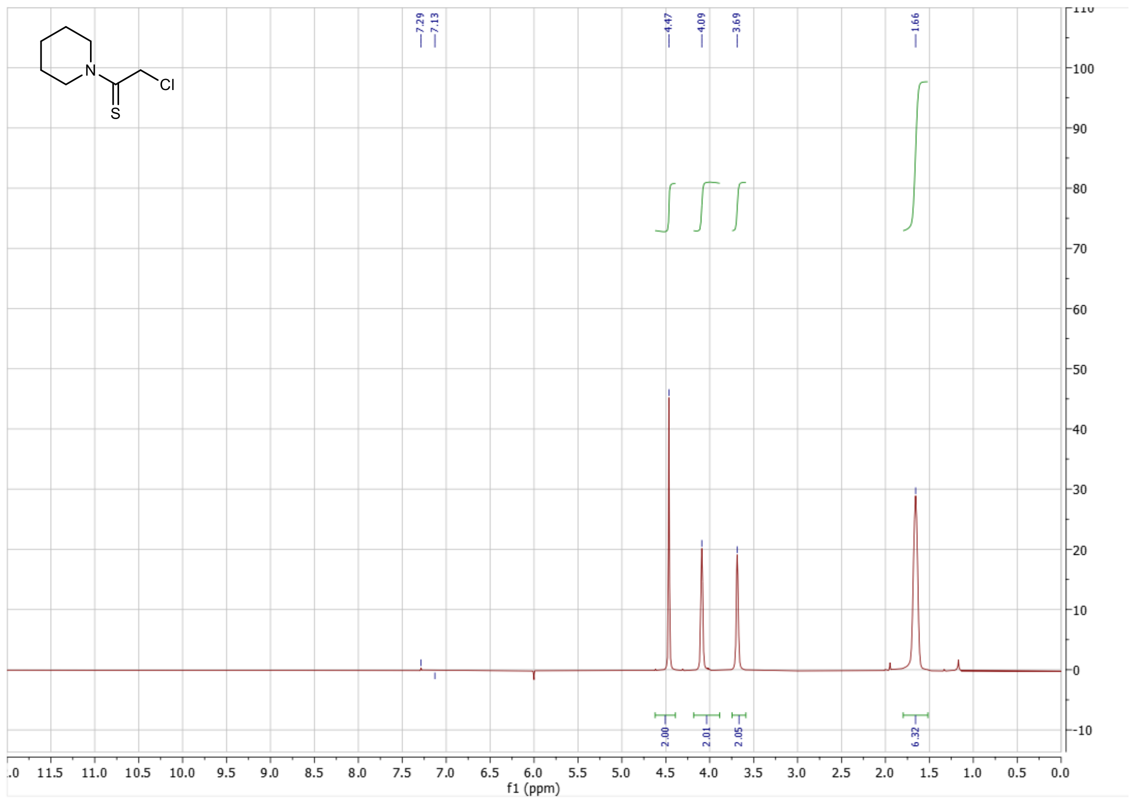

Figure S28. 2-chloro-1-(piperidin-1-yl)ethanethione *(***5d**, DMSO-d_6, 500MHz)

N-phenyl-2-fluorothioacetamide (**6a**)

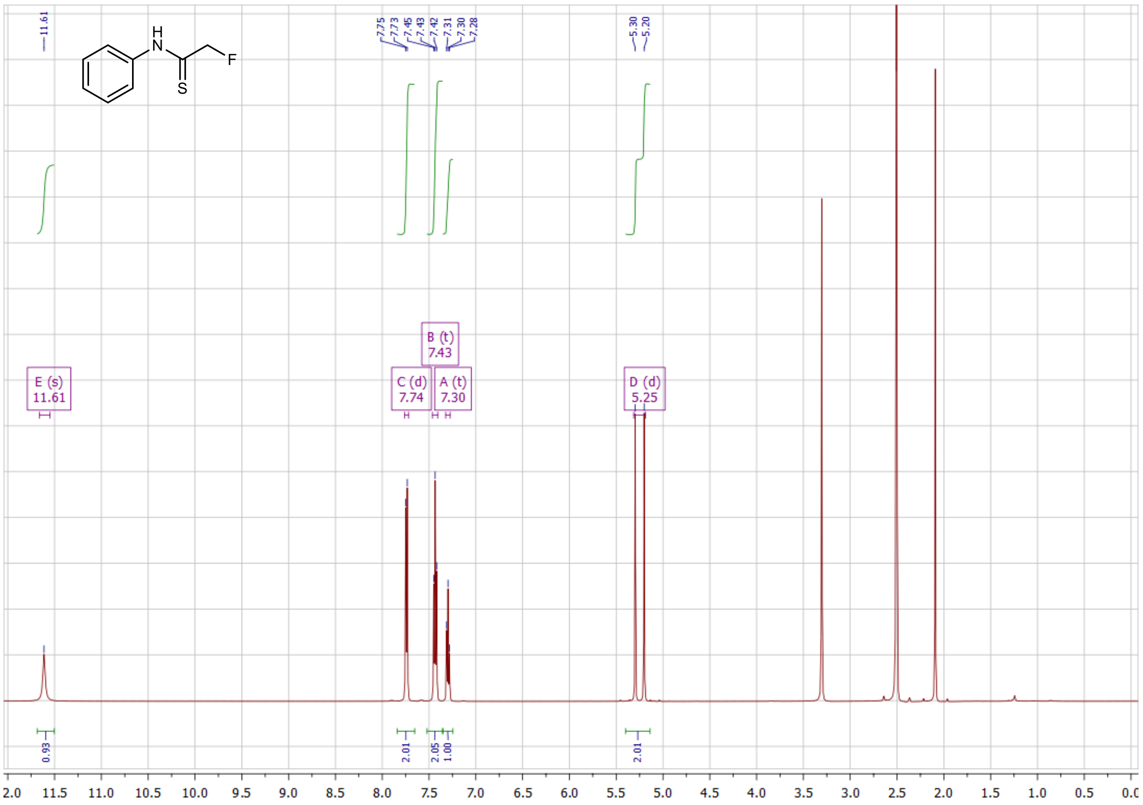

Figure S29. N-phenyl-2-fluorothioacetamide (**6a**, DMSO-d_6, 500MHz)

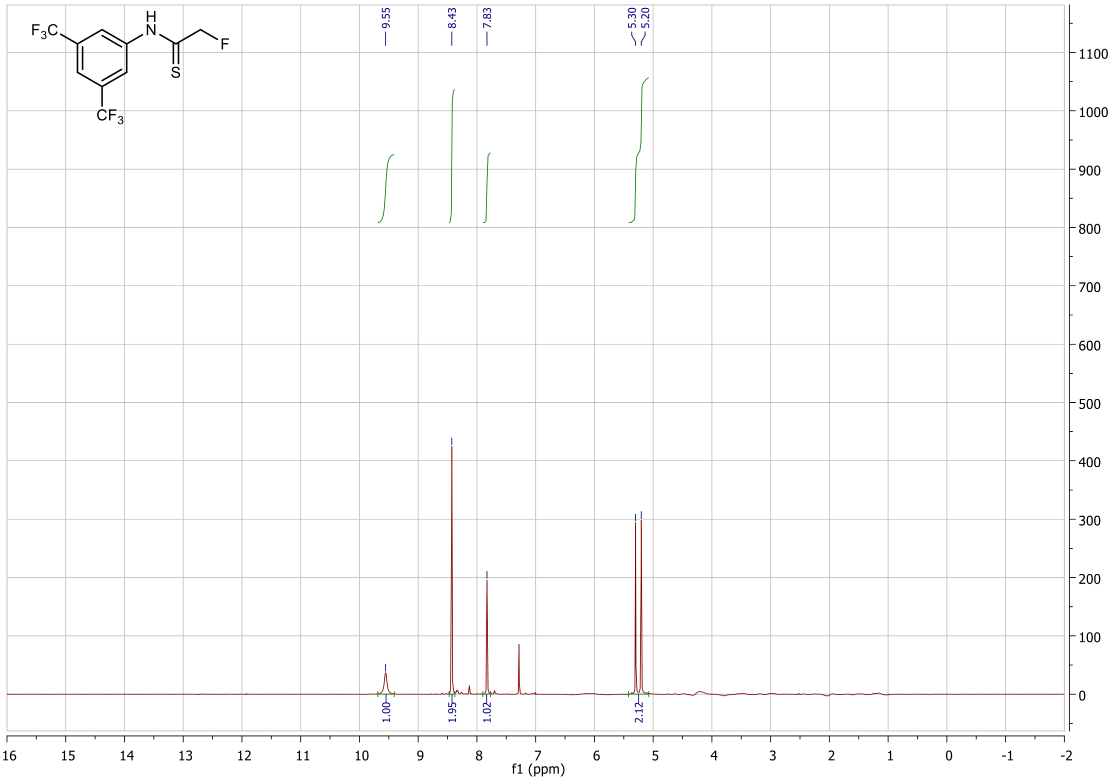

Figure S30. N-(3,5-bis(trifluoromethyl)phenyl)-2-fluorothioacetamide (**6b**, $CDCl_3$, 500MHz)

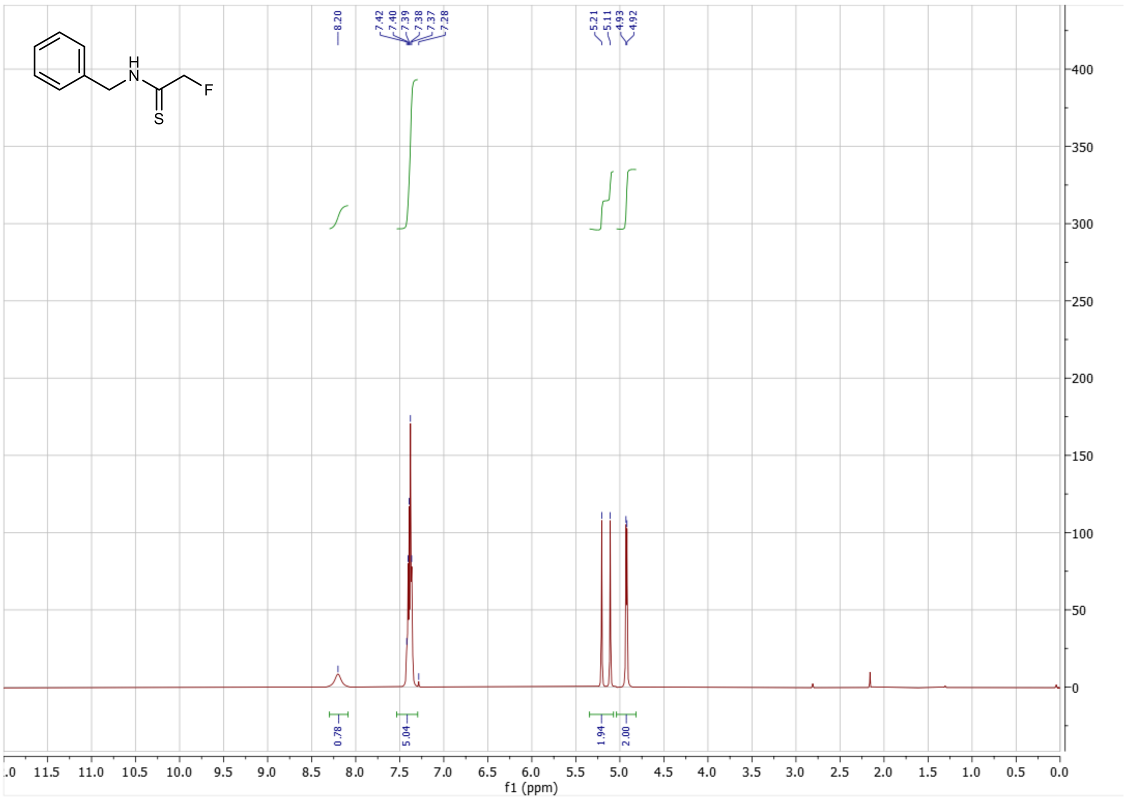

Figure S31. N-benzyl-2-fluoroethanethioamide (**6c**, $CDCl_3$, 500MHz)

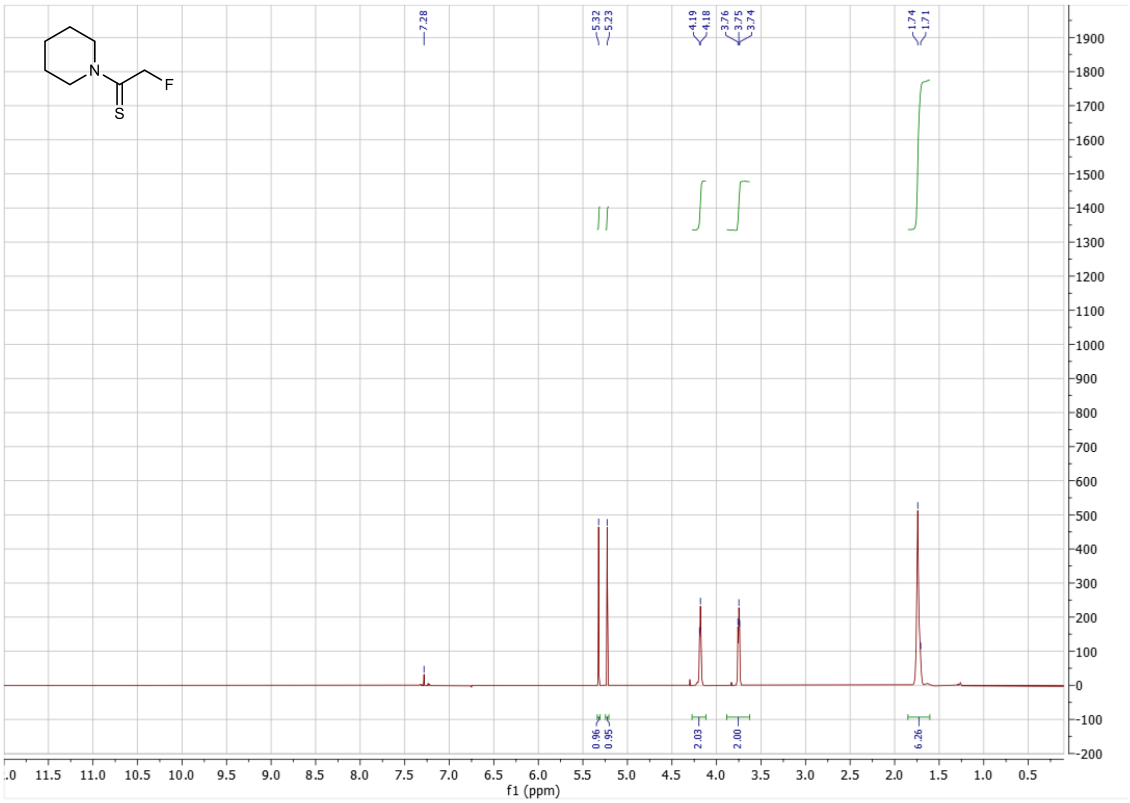

Figure S32. 2-fluoro-1-(piperidin-1-yl)ethanethione (**6d**, $CDCl_3$, 500MHz)

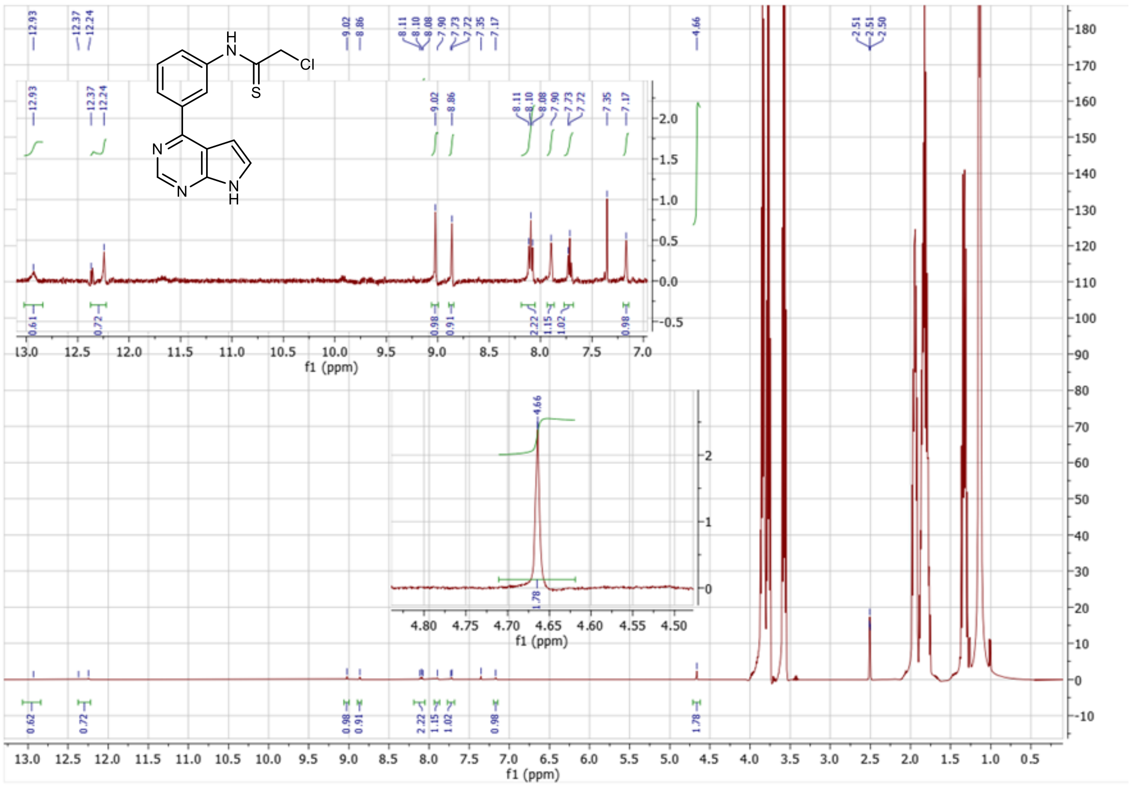

Figure S33. N-(3-(7H-pyrrolo[2,3-d]pyrimidin-4-yl)phenyl)-2-chloroethanethioamide *(***9**, DMSO-d_6, 500MHz)

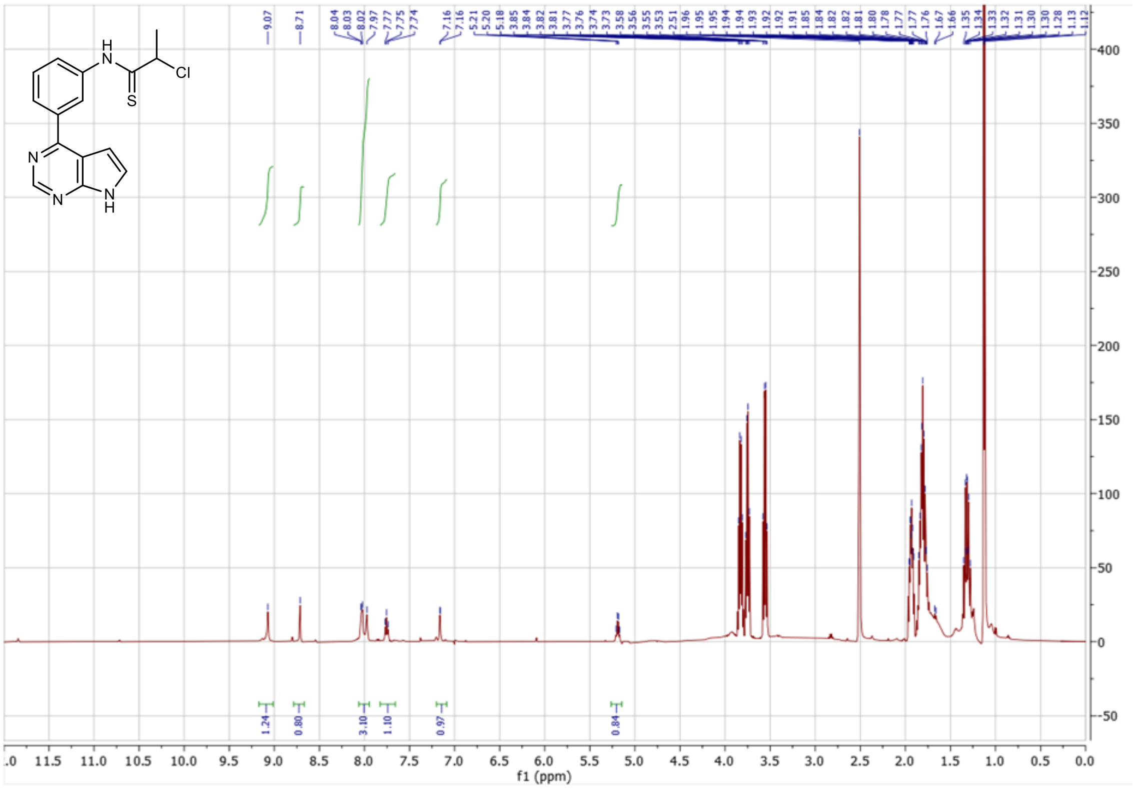

Figure S34. N-(3-(7H-pyrrolo[2,3-d]pyrimidin-4-yl)phenyl)-2-chloropropanethioamide *(***10**, DMSO-d_6, 500MHz)

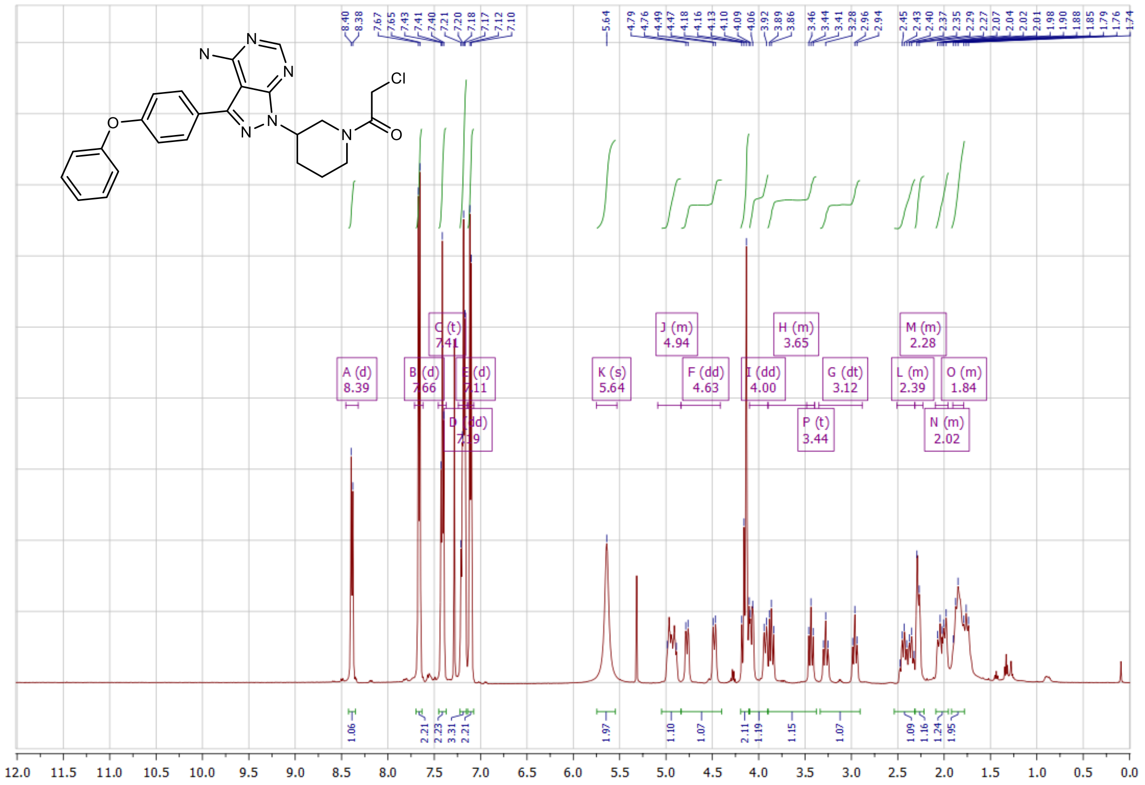

Figure S35. 1-(3-(4-Amino-3-(4-phenoxyphenyl)-1H-pyrazolo[3.4-d]pyrimidin-1-yl)piperidin-1-yl)-2-chloroethanone *(***12**, $CDCl_3$, 500MHz)

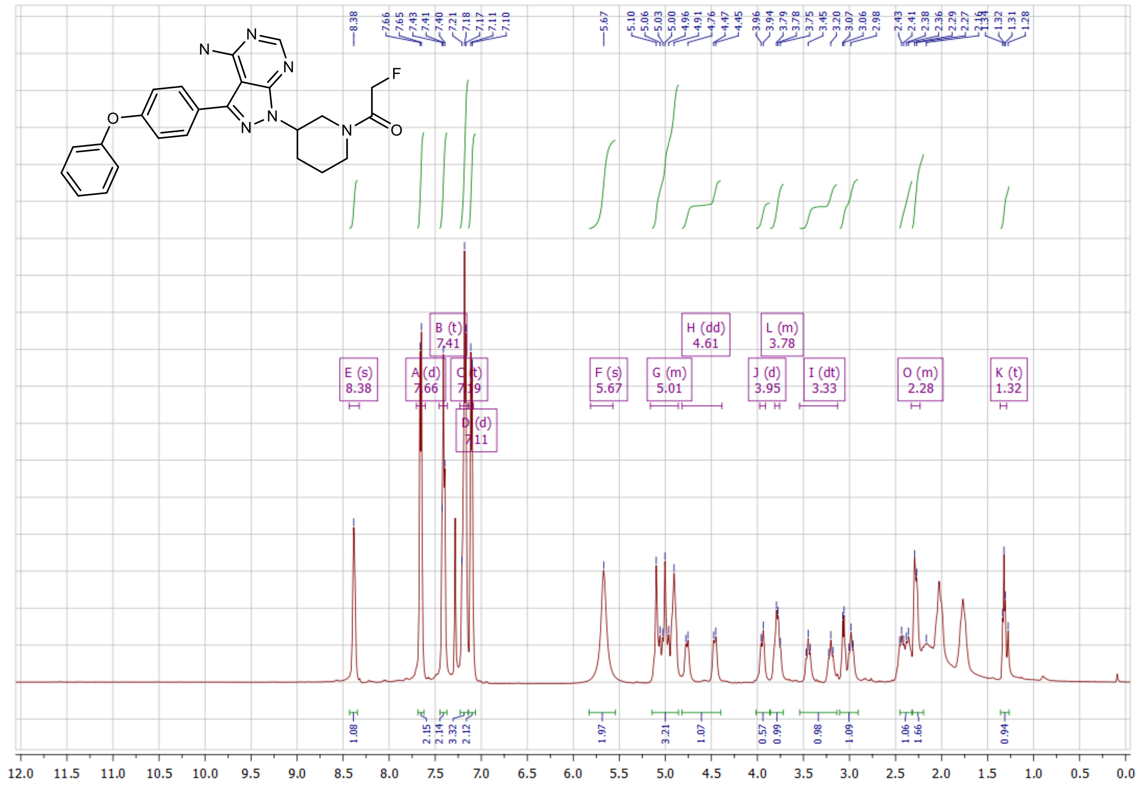

Figure S36. 1-(3-(4-amino-3-(4-phenoxyphenyl)-1H-pyrazolo[3.4-d]pyrimidin-1-yl)piperidin-1-yl)-2-fluoroethanone *(***13**, $CDCl_3$, 500MHz)

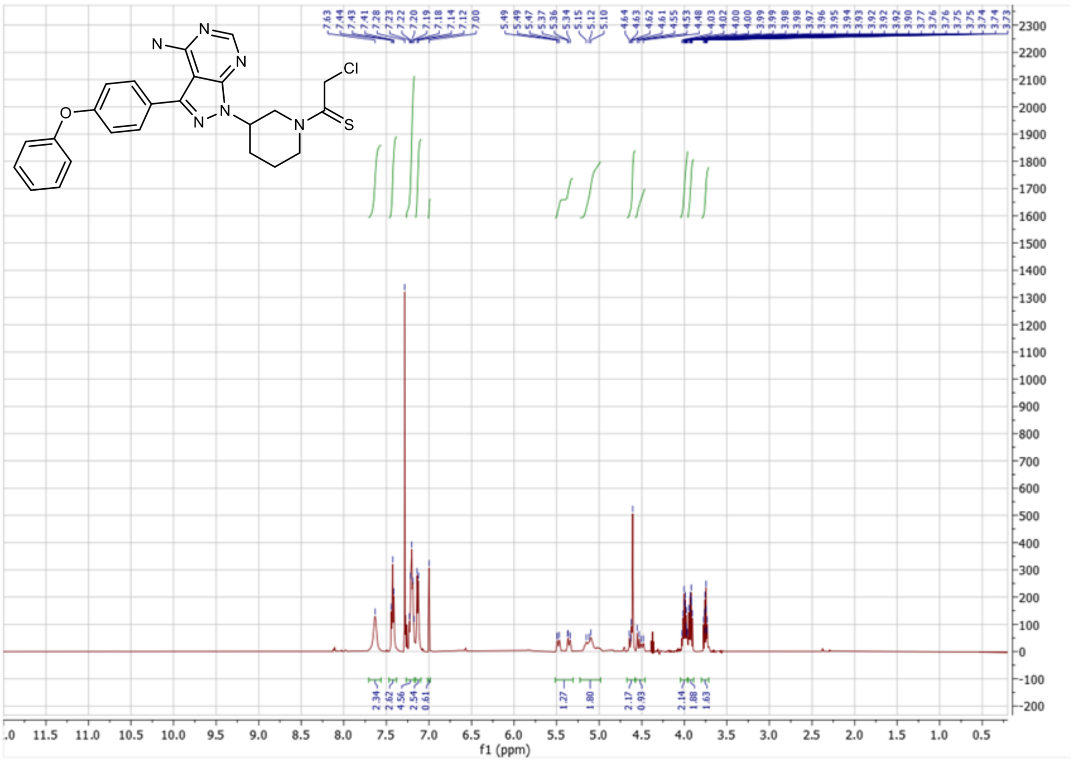

Figure S35. 1-(3-(4-Amino-3-(4-phenoxyphenyl)-1H-pyrazolo[3.4-d]pyrimidin-1-yl)piperidin-1-yl)-2-chloroethanethione *(***14**, $CDCl_3$, 500MHz)

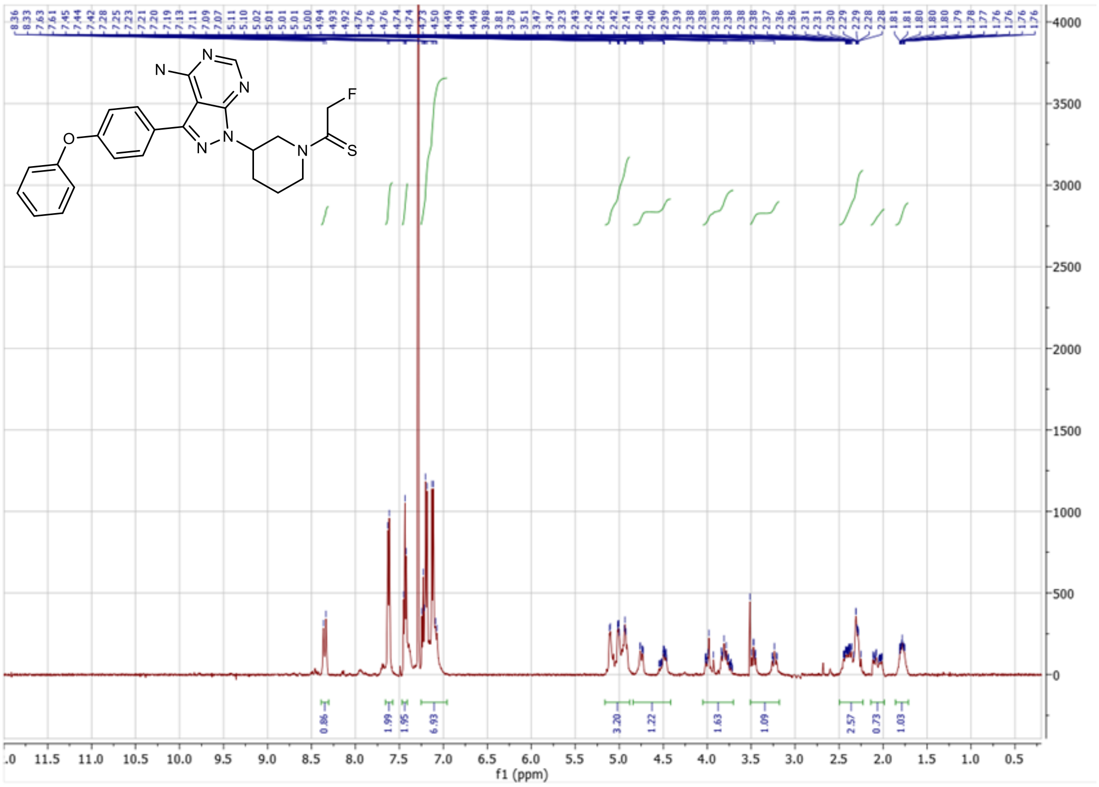

Figure S38. 1-(3-(4-Amino-3-(4-phenoxyphenyl)-1H-pyrazolo[3.4-d]pyrimidin-1-yl)piperidin-1-yl)-2-fluoroethanethione *(***15**, $CDCl_3$, 500MHz)

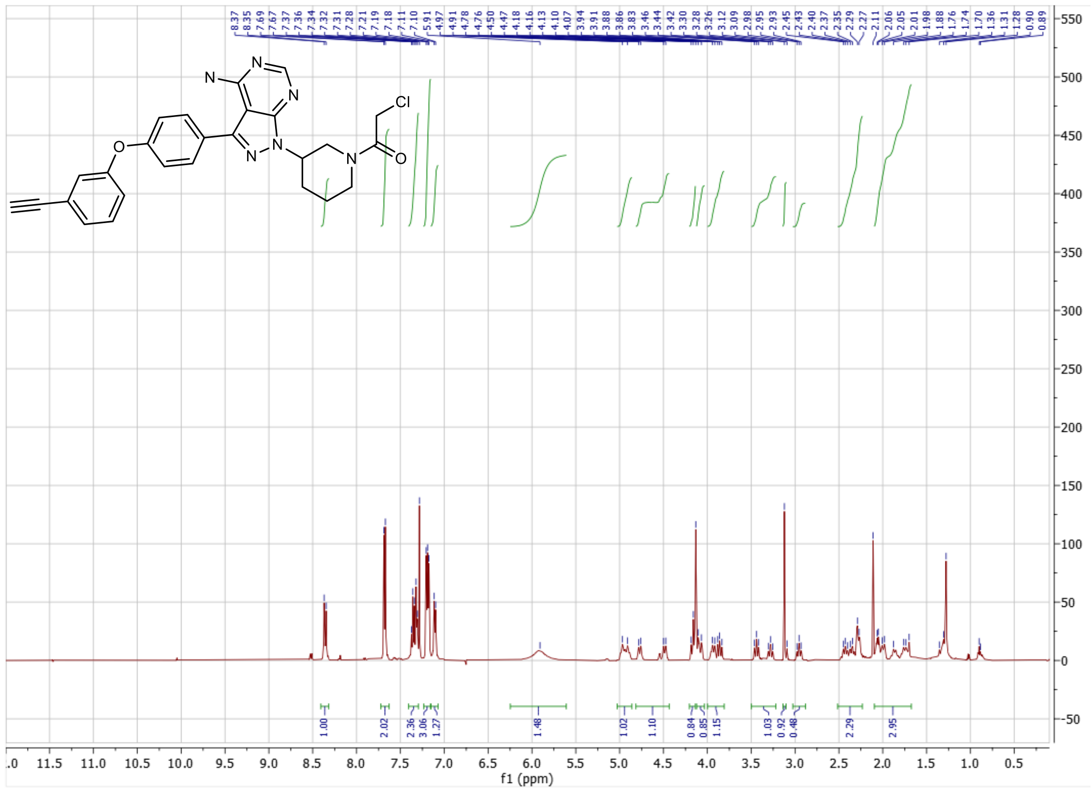

Figure S39. 1-(3-(4-Amino-3-(4-(3-ethynylphenoxy)phenyl)-1H-pyrazolo[3,4-d]pyrimidin-1-yl)piperidin-1-yl)-2-chloroethan-1-one *(***17**, $CDCl_3$, 500MHz)

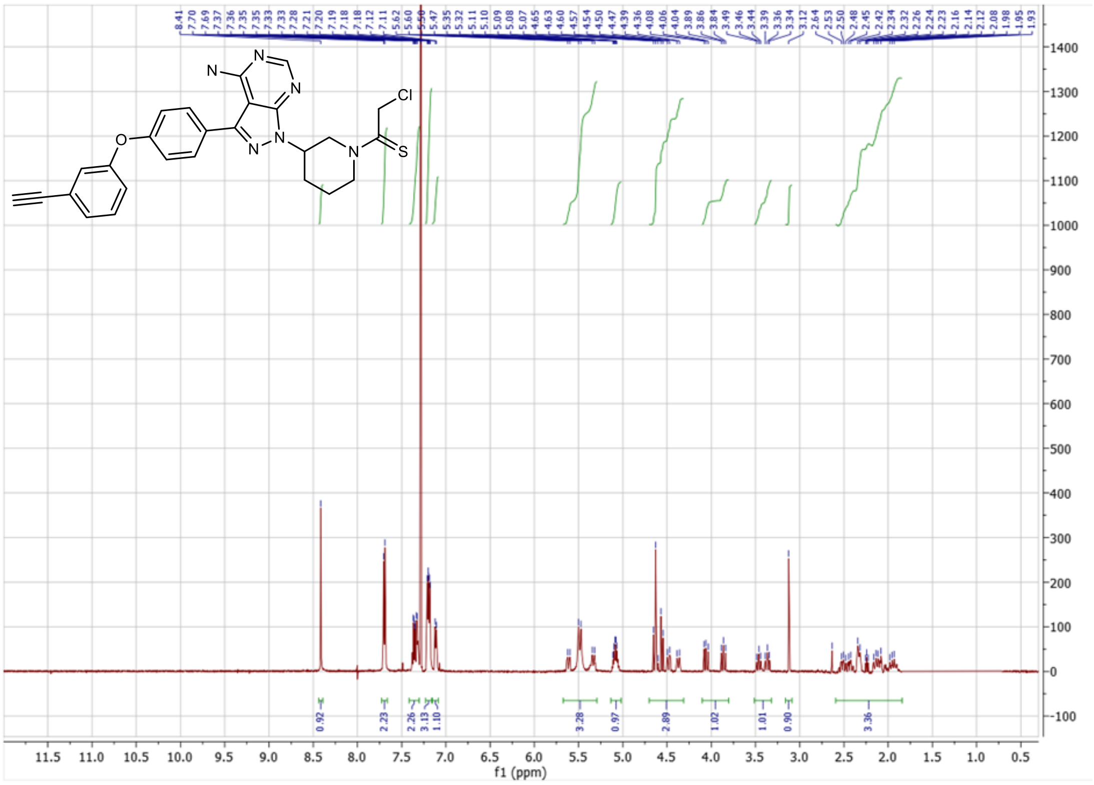

Figure S40. 1-(3-(4-Amino-3-(4-(3-ethynylphenoxy)phenyl)-1H-pyrazolo[3,4-d]pyrimidin-1-yl)piperidin-1-yl)-2-chloroethan-1-thione *(***18**, $CDCl_3$, 500MHz)

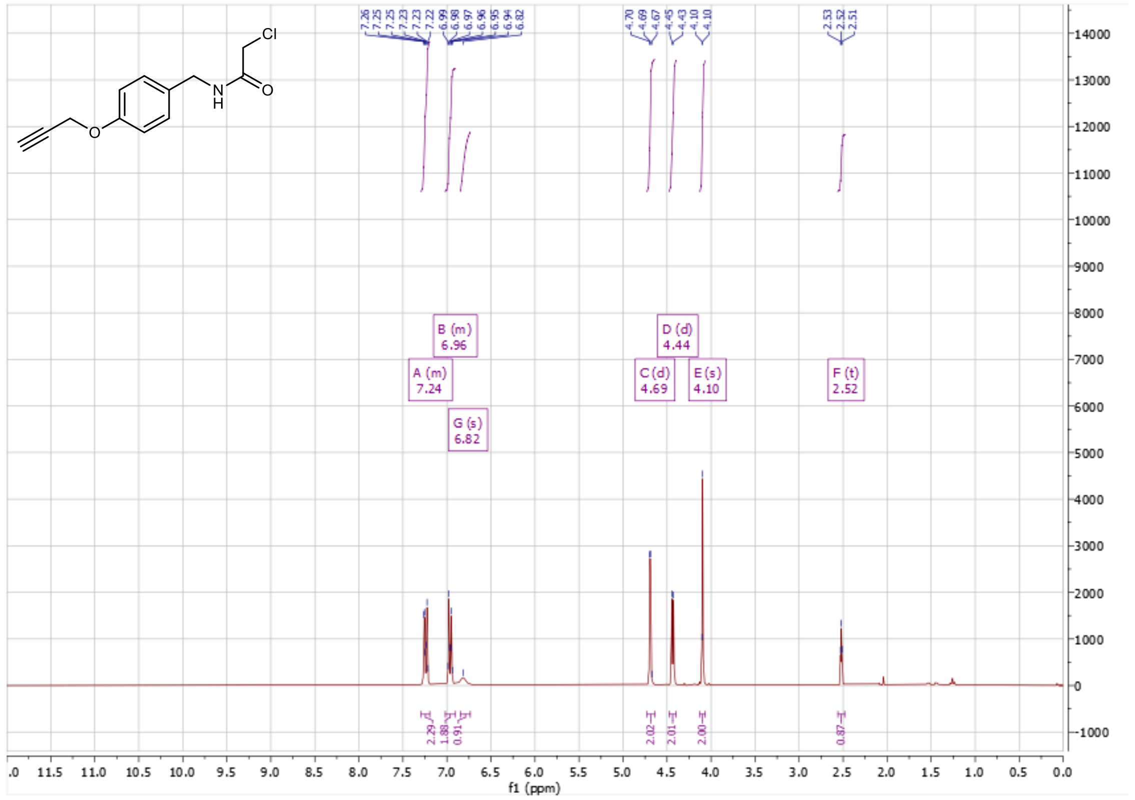

Figure S41. 2-Chloro-N-(4-(prop-2-yn-1-yloxy)phenyl)acetamide *(***19**, $CDCl_3$, 500MHz)

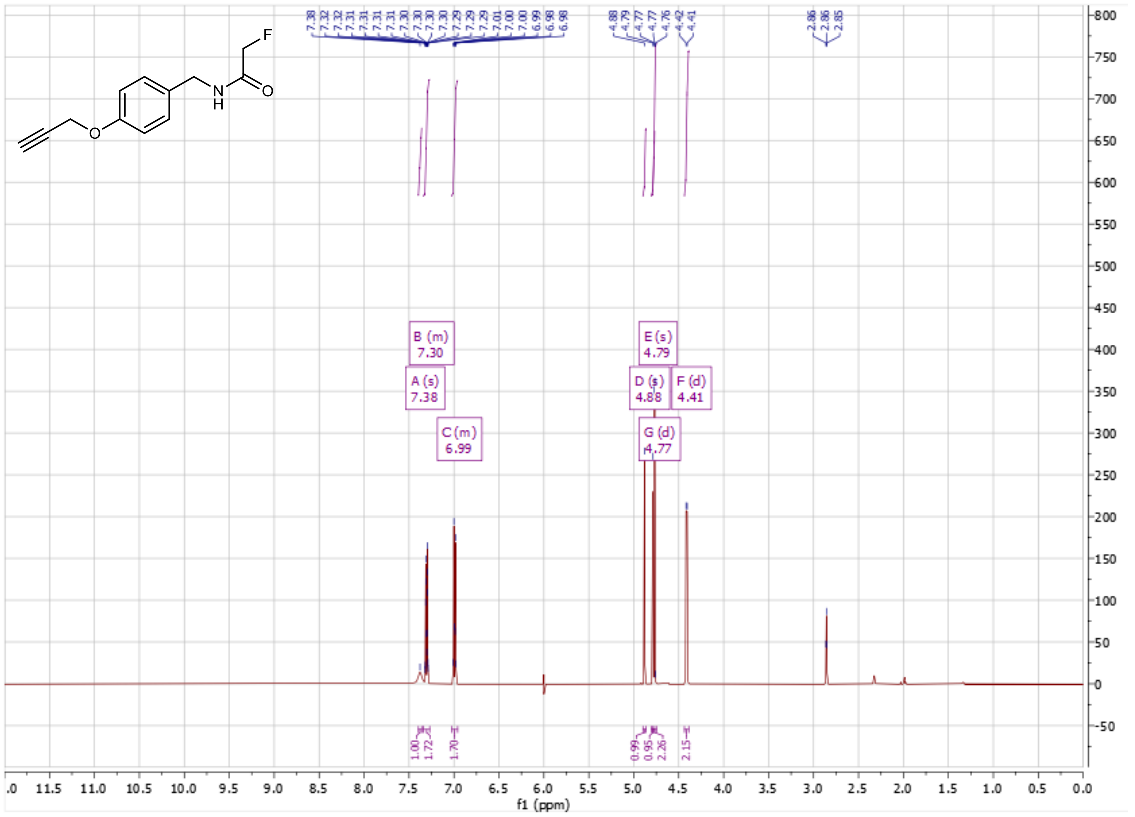

Figure S42. 2-Fluoro-N-(4-(prop-2-yn-1-yloxy)phenyl)acetamide *(***20**, CD_3CN, 500MHz)

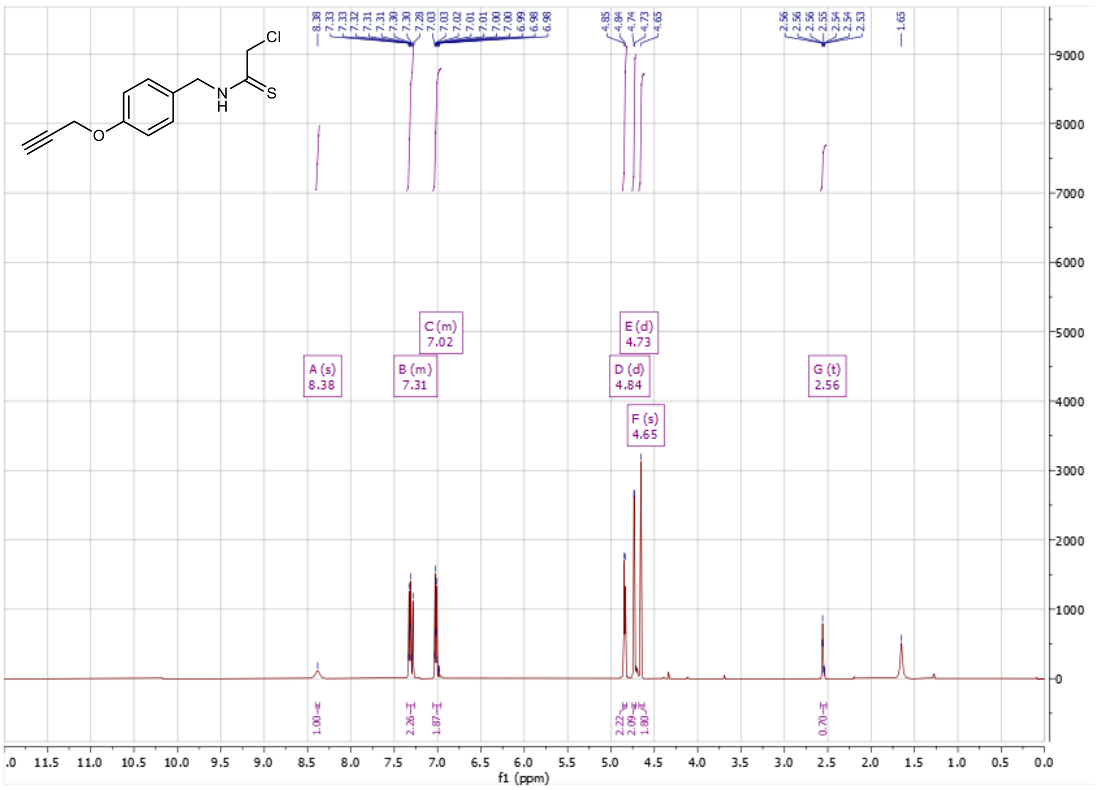

Figure S43. 2-Chloro-N-(4-(prop-2-yn-1-yloxy)phenyl)ethanethioamide *(***21**, $CDCl_3$, 500MHz)

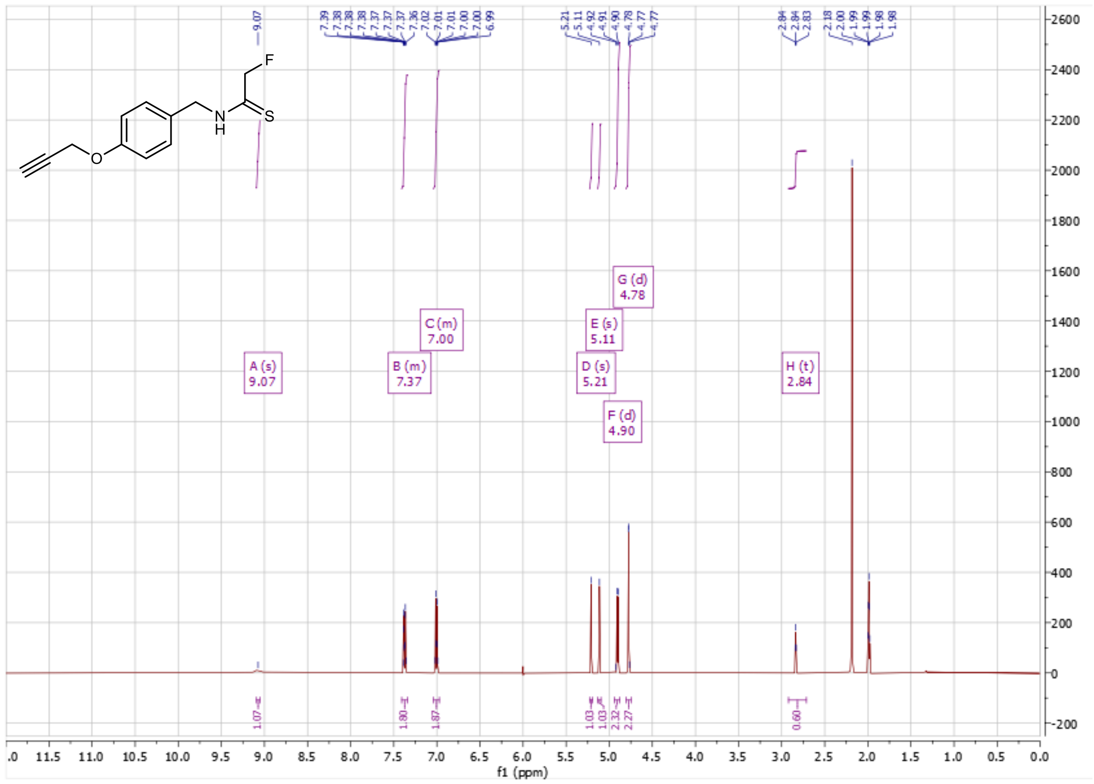

Figure S44. 2-Fluoro-N-(4-(prop-2-yn-1-yloxy)phenyl)ethanethioamide *(***22**, CD_3CN, 500MHz)

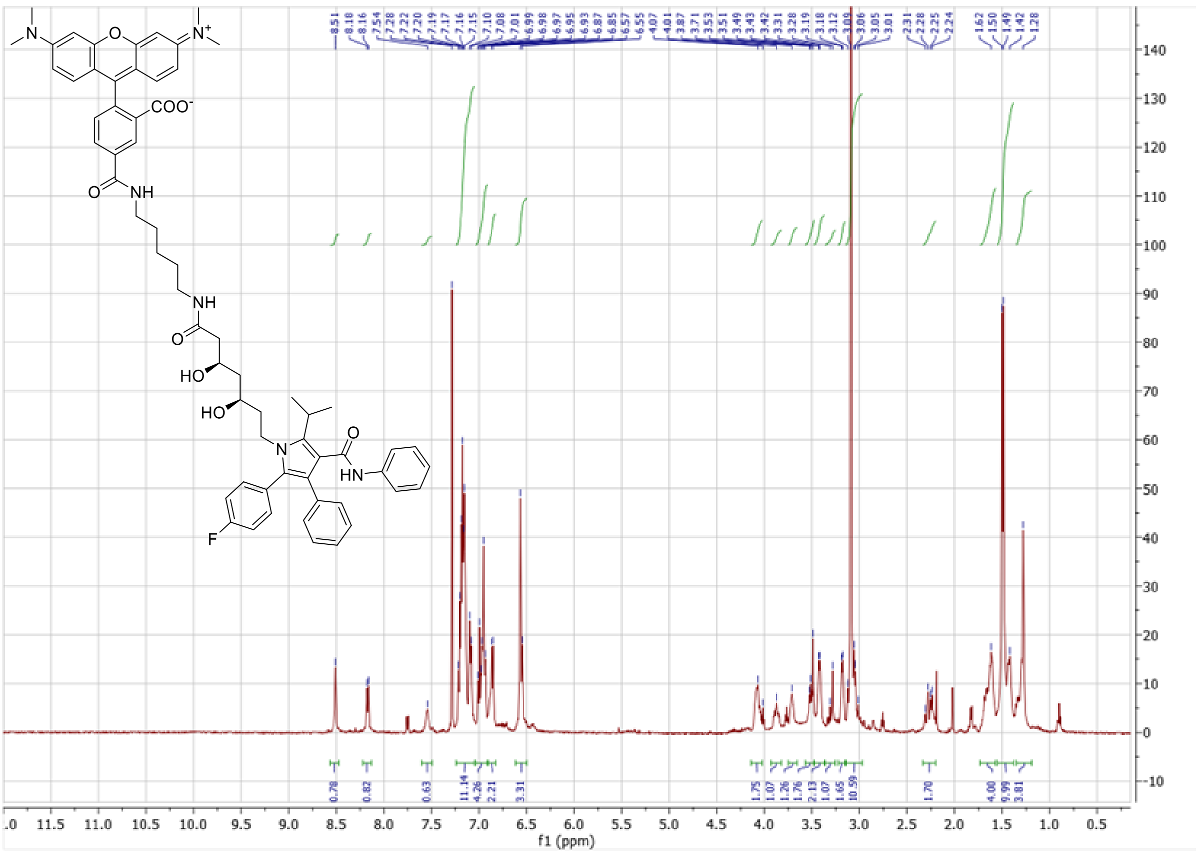

Figure S45. 2-(6-(dimethylamino)-3-(dimethyliminio)-3H-xanthen-9-yl)-5-(((5-((3R,5R)-7-(2-(4-fluorophenyl)-5-isopropyl-3-phenyl-4-(phenylcarbamoyl)-1H-pyrrol-1-yl)-3,5-dihydroxyheptanamido)pentyl)oxy) carbonyl)benzoate (**24**, $CDCl_3$, 500MHz)

C-NMR spectra of the covalent probes

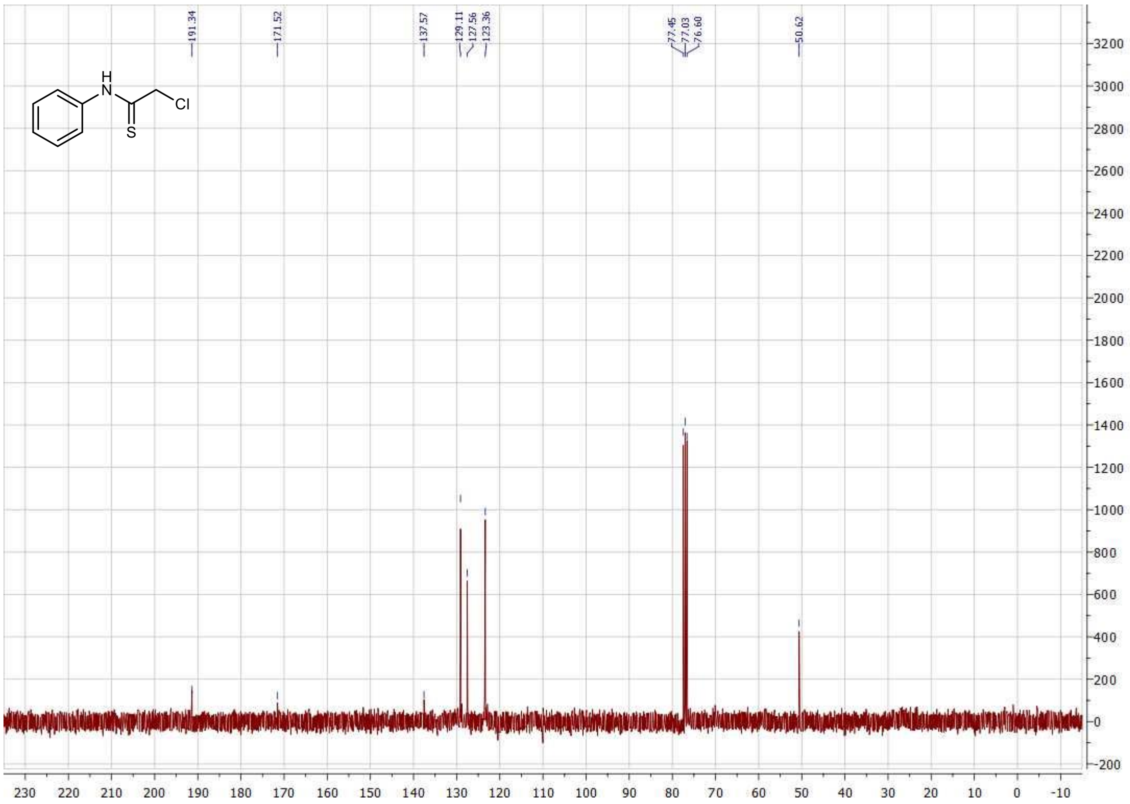

Figure S46. N-phenyl-2-chlorothioacetamide (**5a**, $CDCl_3$, 125MHz)

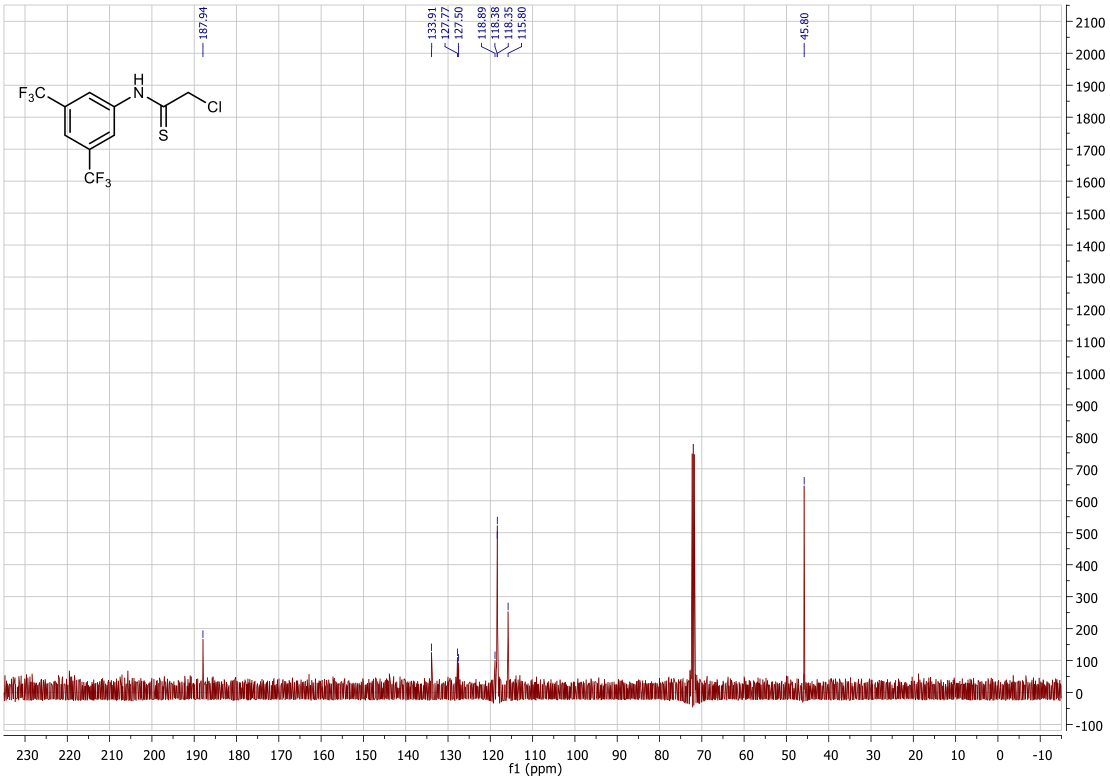

Figure S47. N-(3,5-bis(trifluoromethyl)phenyl)-2-chlorothioacetamide (**5b**, DMSO-d_6, 125MHz)

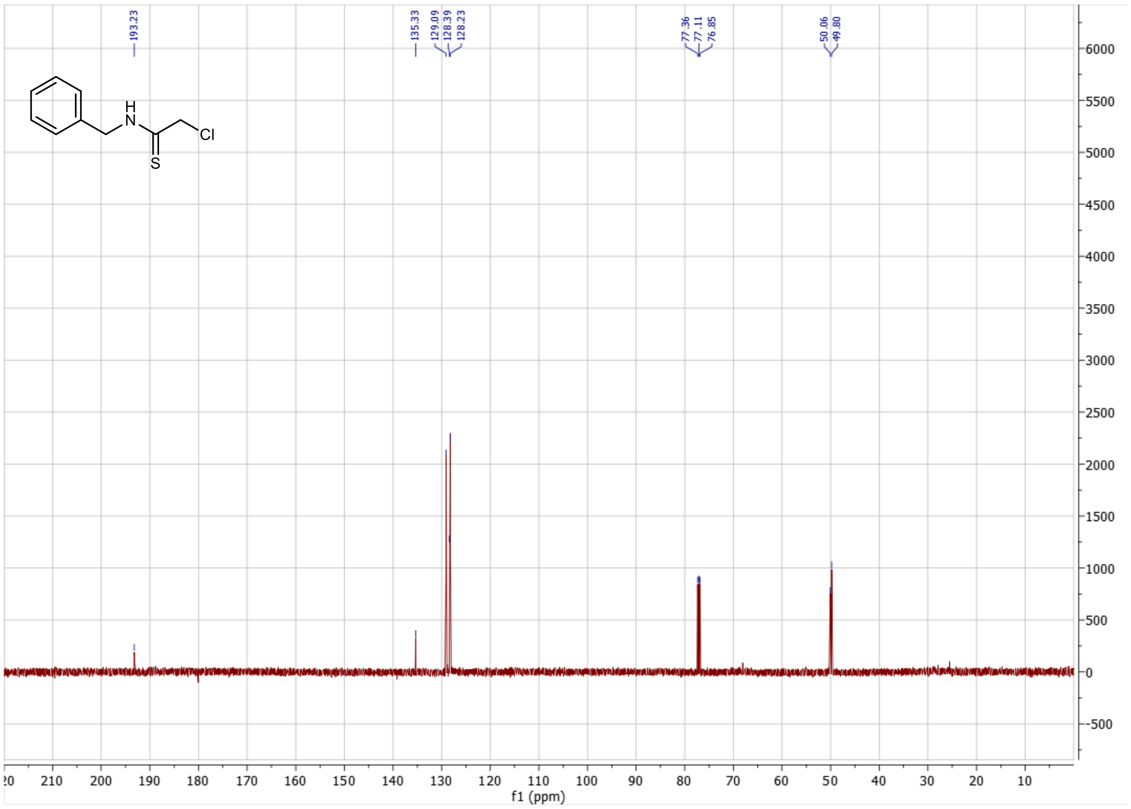

Figure S48. N-benzyl-2-chloroethanethioamide (**5c**, $CDCl_3$, 125MHz)

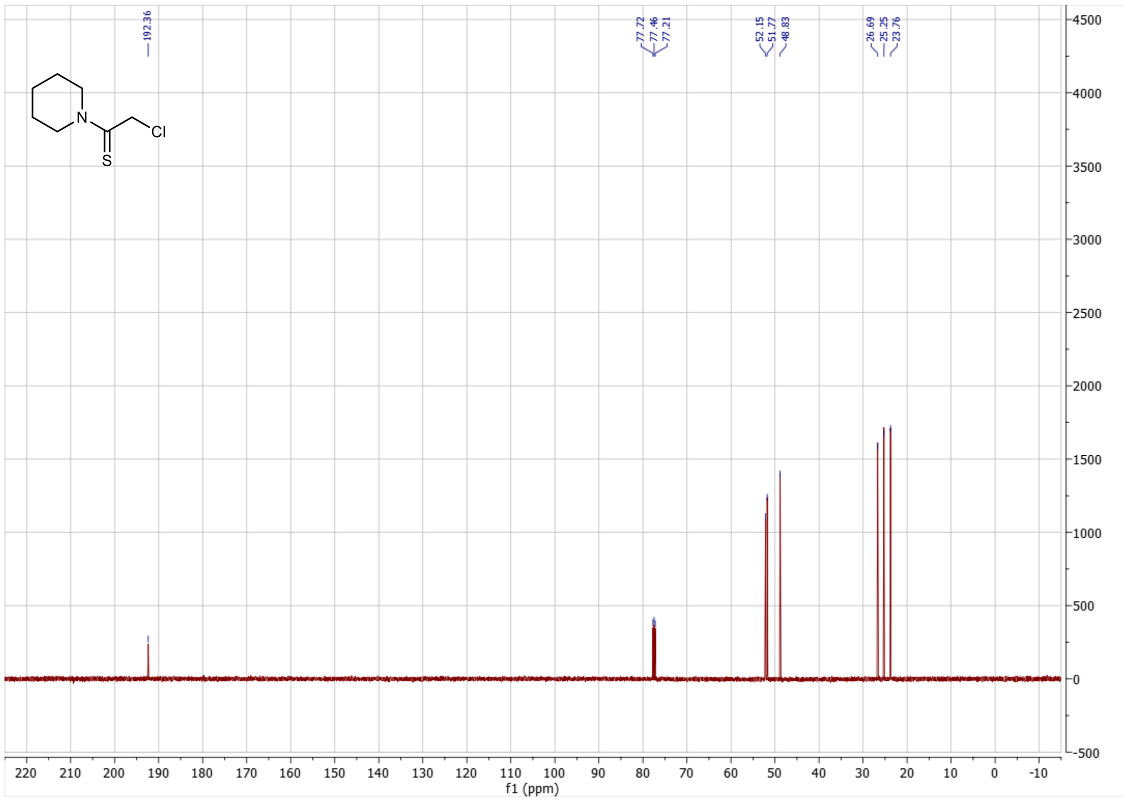

Figure S49. 2-chloro-1-(piperidin-1-yl)ethanethione (**5d**, $CDCl_3$, 125MHz)

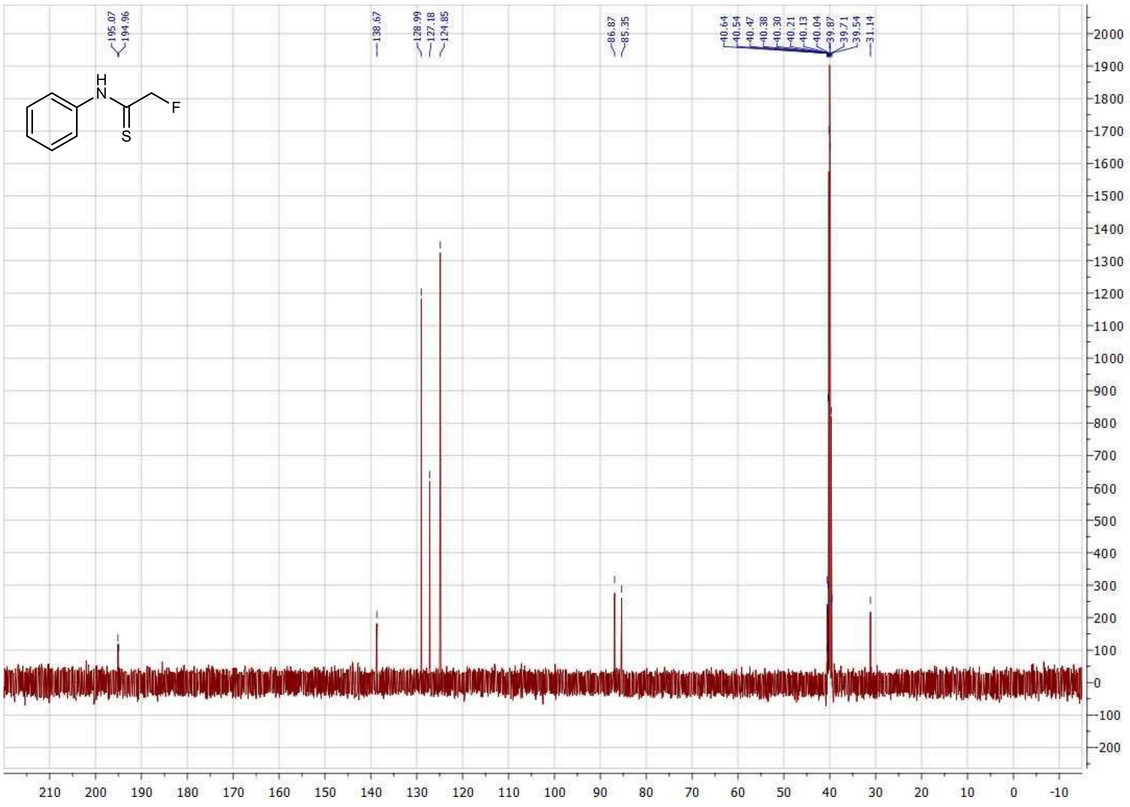

Figure S50. N-phenyl-2-fluorothioacetamide (**6a**, $CDCl_3$, 125MHz)

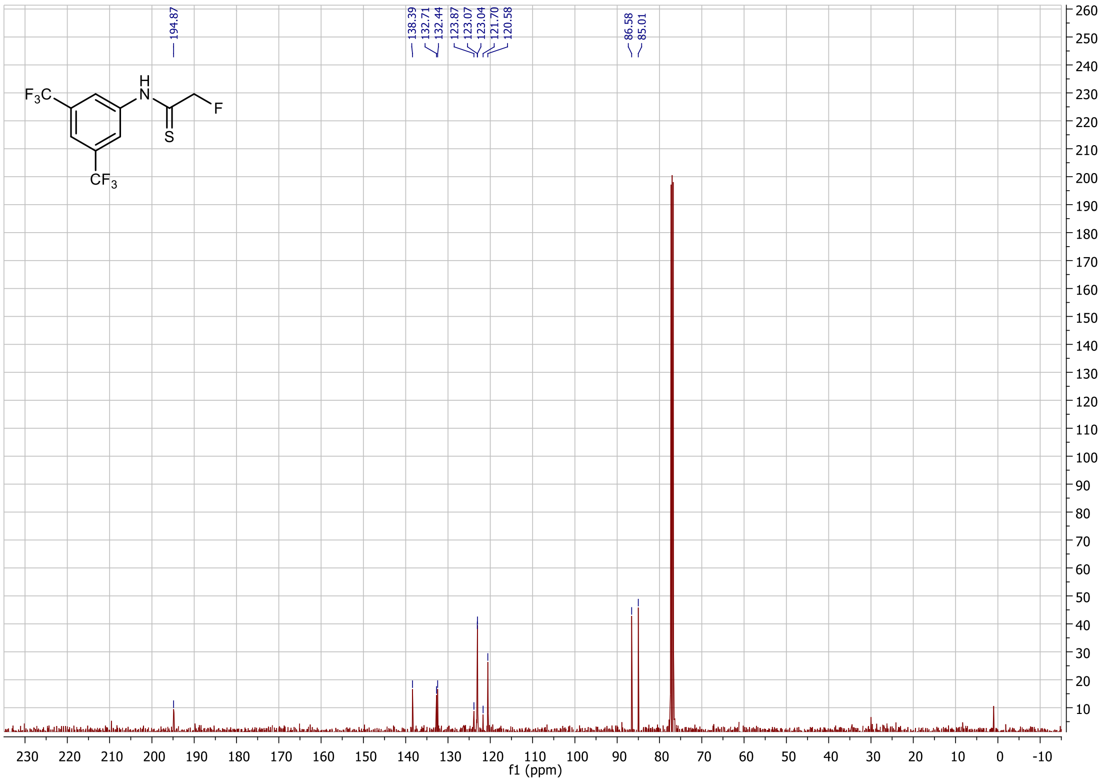

Figure S51. N-(3,5-bis(trifluoromethyl)phenyl)-2-fluorothioacetamide (**6b**, $CDCl_3$, 125MHz)

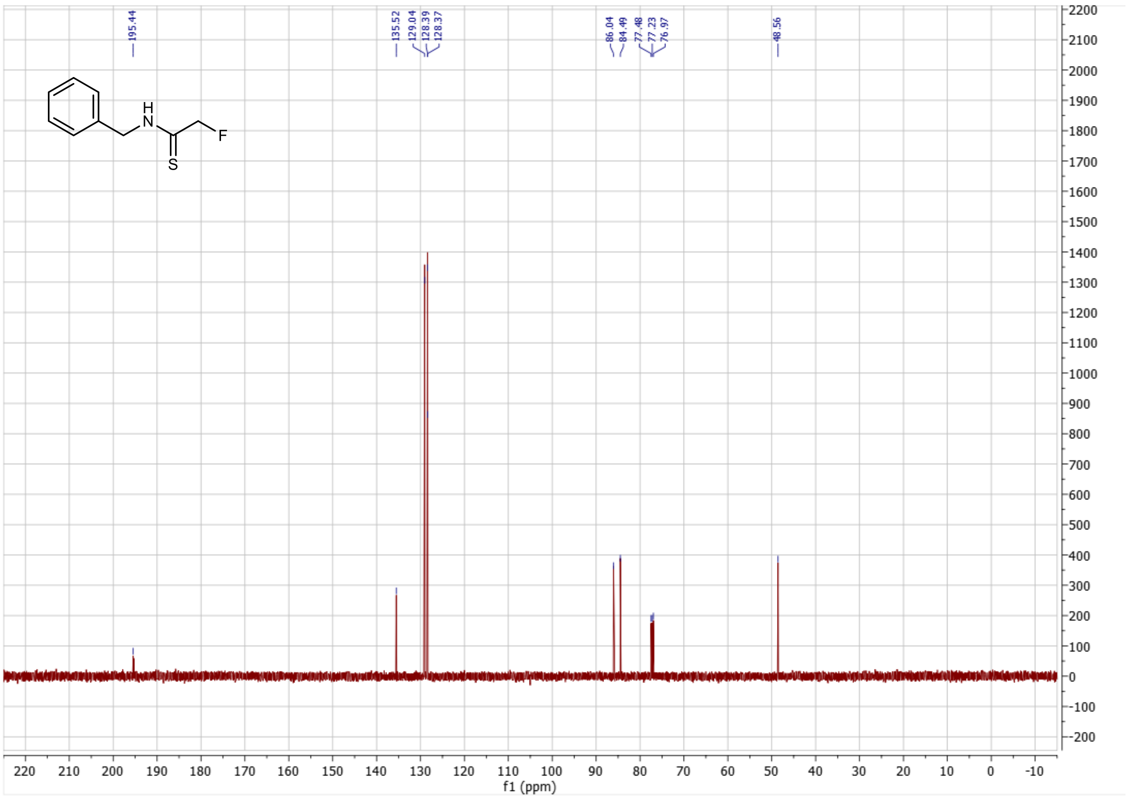

Figure S52. N-benzyl-2-fluoroethanethioamide (**6c**, $CDCl_3$, 125MHz)

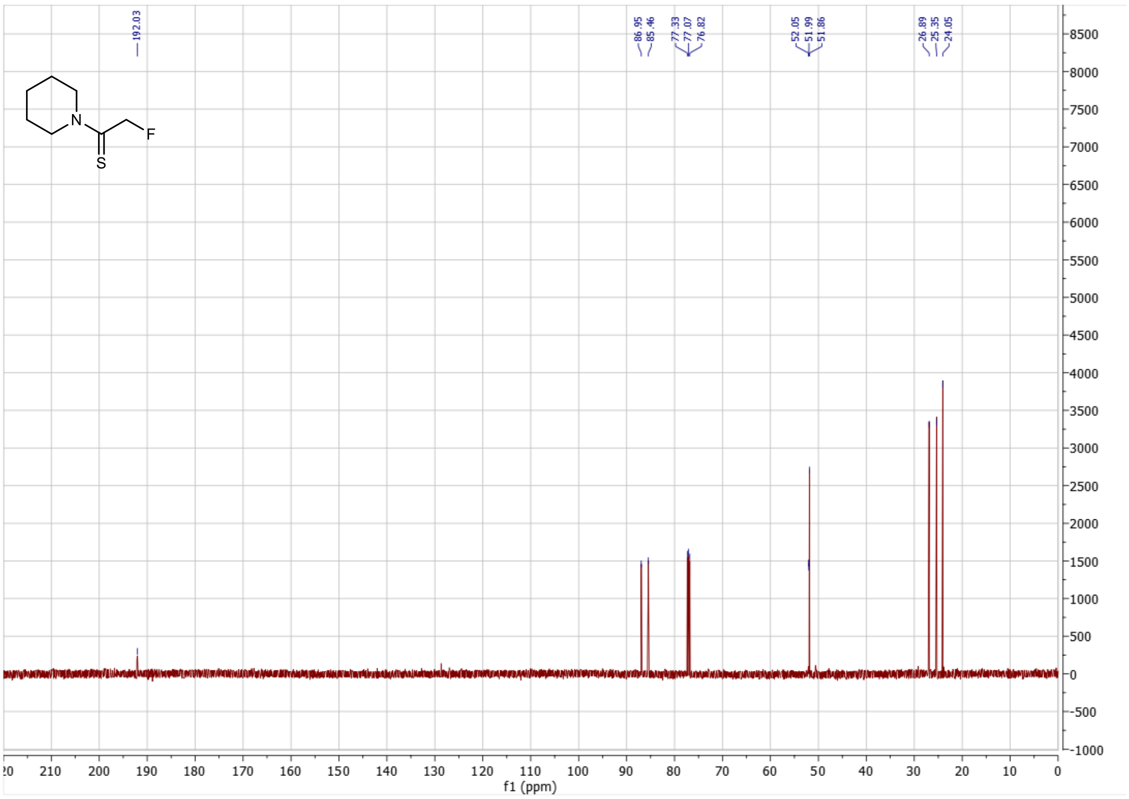

Figure S53. 2-fluoro-1-(piperidin-1-yl)ethanethione (**6d**, $CDCl_3$, 125MHz)

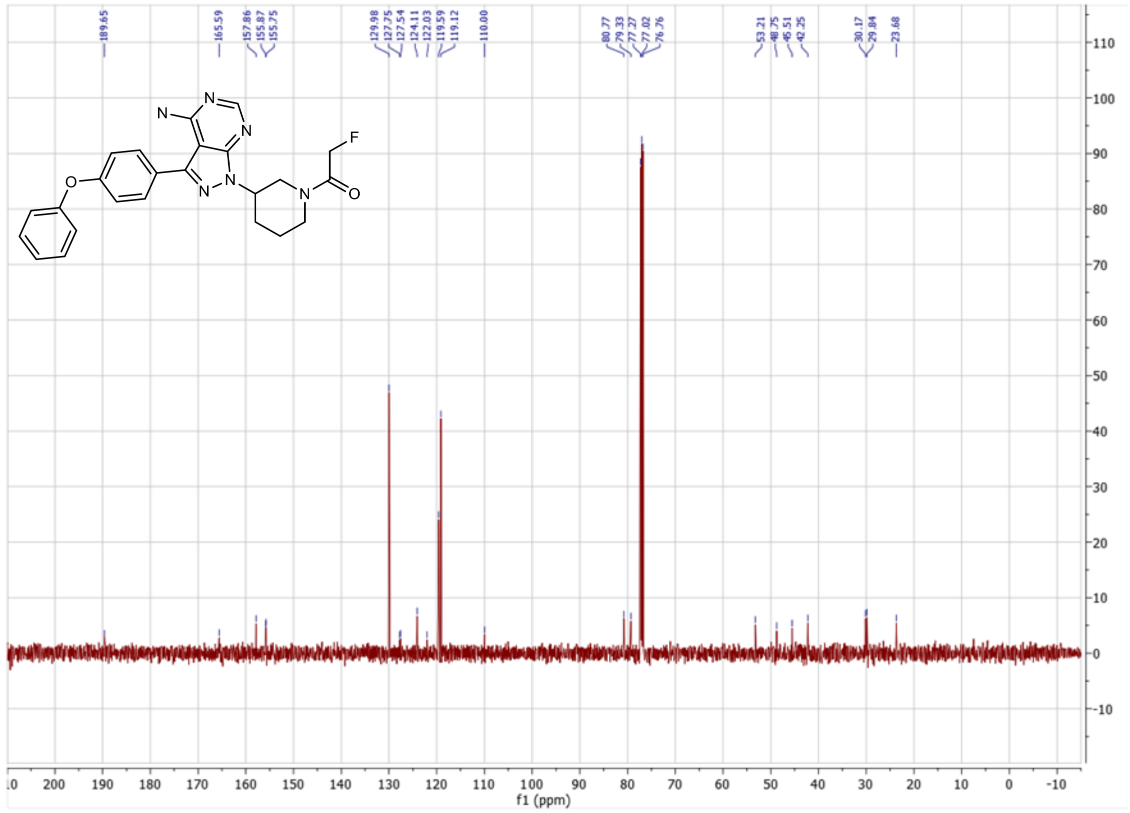

Figure S54. 1-(3-(4-amino-3-(4-phenoxyphenyl)-1H-pyrazolo[3.4-d]pyrimidin-1-yl)piperidin-1-yl)-2-fluoroethanone (**13**, $CDCl_3$, 125MHz)

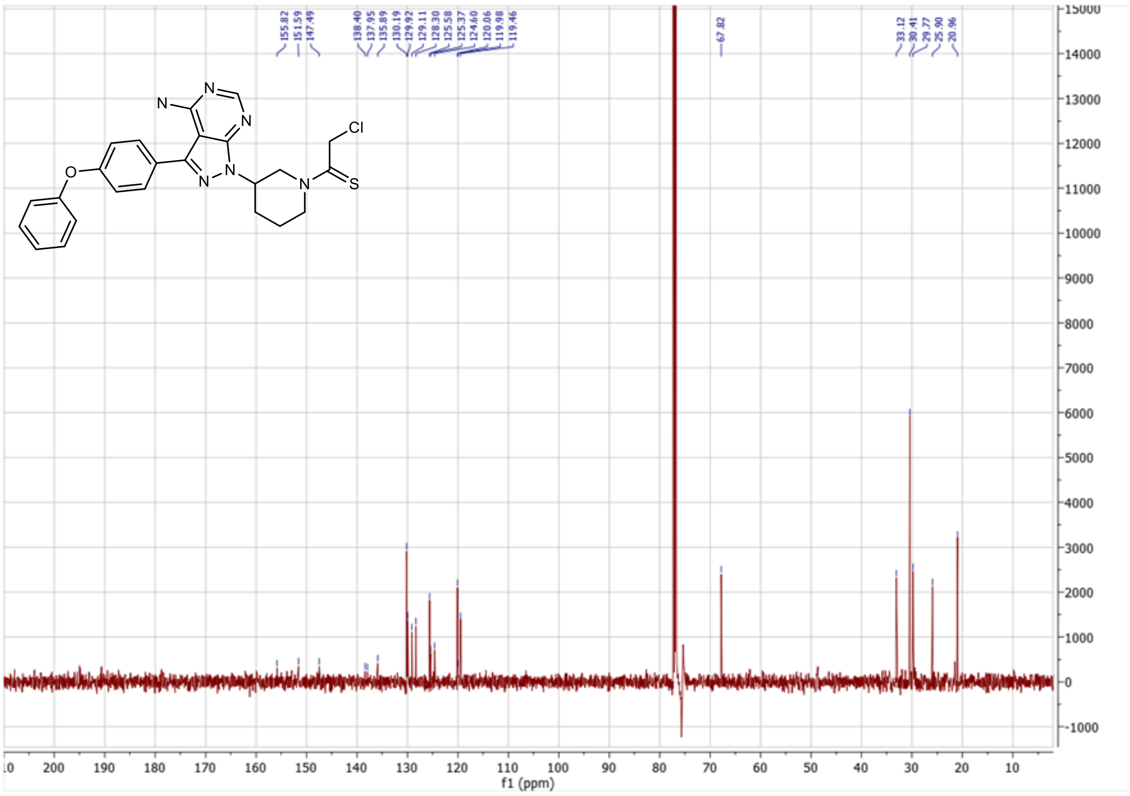

Figure S55. 1-(3-(4-Amino-3-(4-phenoxyphenyl)-1H-pyrazolo[3.4-d]pyrimidin-1-yl)piperidin-1-yl)-2-chloroethanethione (**14**, $CDCl_3$, 125MHz)

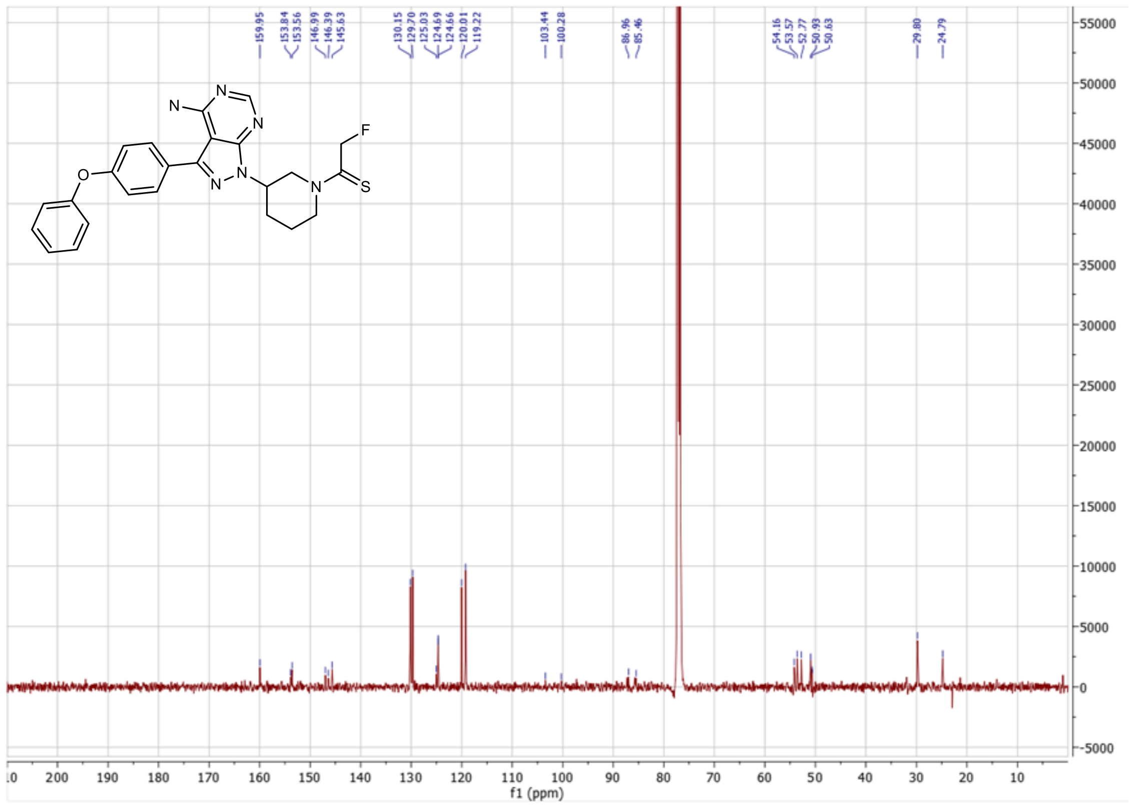

Figure S56. 1-(3-(4-Amino-3-(4-phenoxyphenyl)-1H-pyrazolo[3.4-d]pyrimidin-1-yl)piperidin-1-yl)-2-fluoroethanethione (**15**, $CDCl_3$, 125MHz)

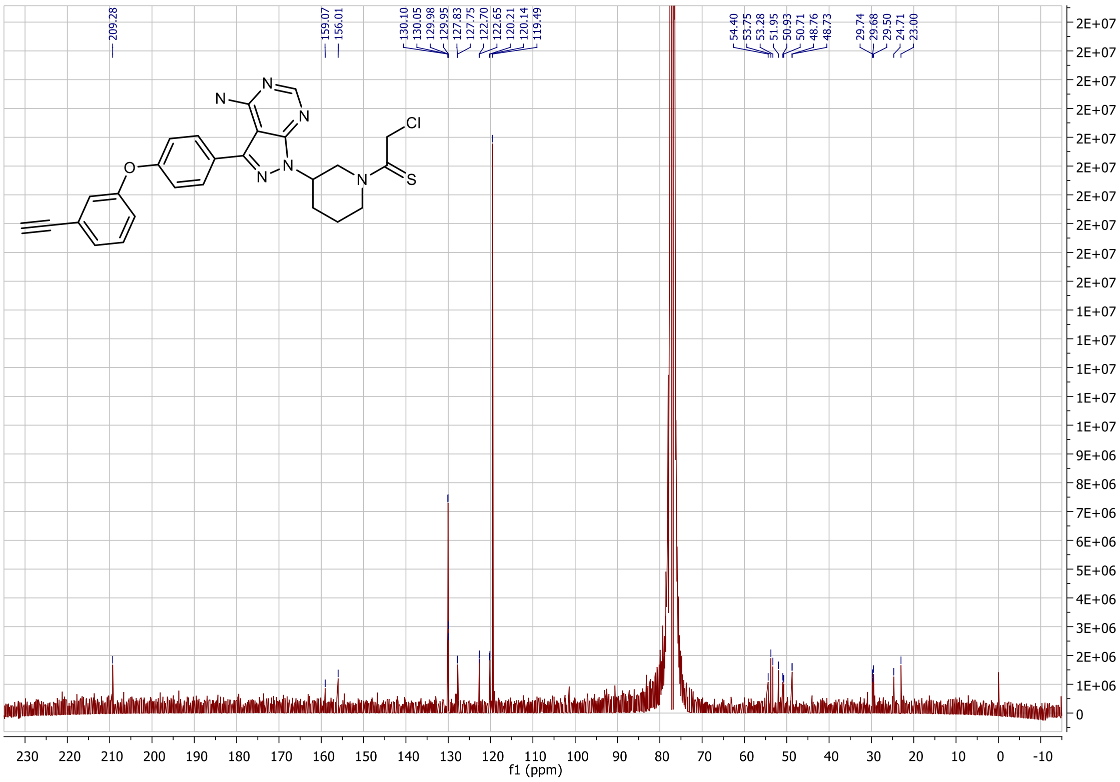

Figure S57. 1-(3-(4-Amino-3-(4-(3-ethynylphenoxy)phenyl)-1H-pyrazolo[3,4-d]pyrimidin-1-yl)piperidin-1-yl)-2-chloroethan-1-thione (**18**, $CDCl_3$, 125MHz)

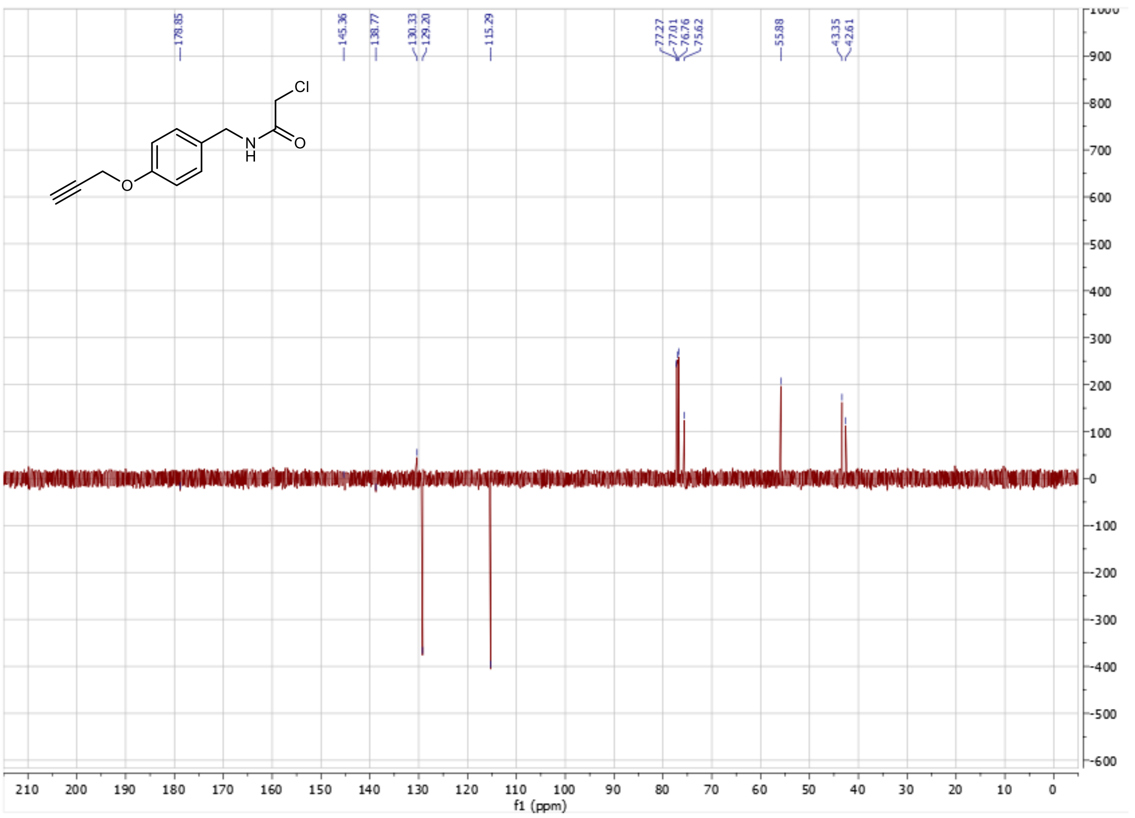

Figure S58. 2-Chloro-N-(4-(prop-2-yn-1-yloxy)phenyl)acetamide (**19**, $CDCl_3$, 125MHz)

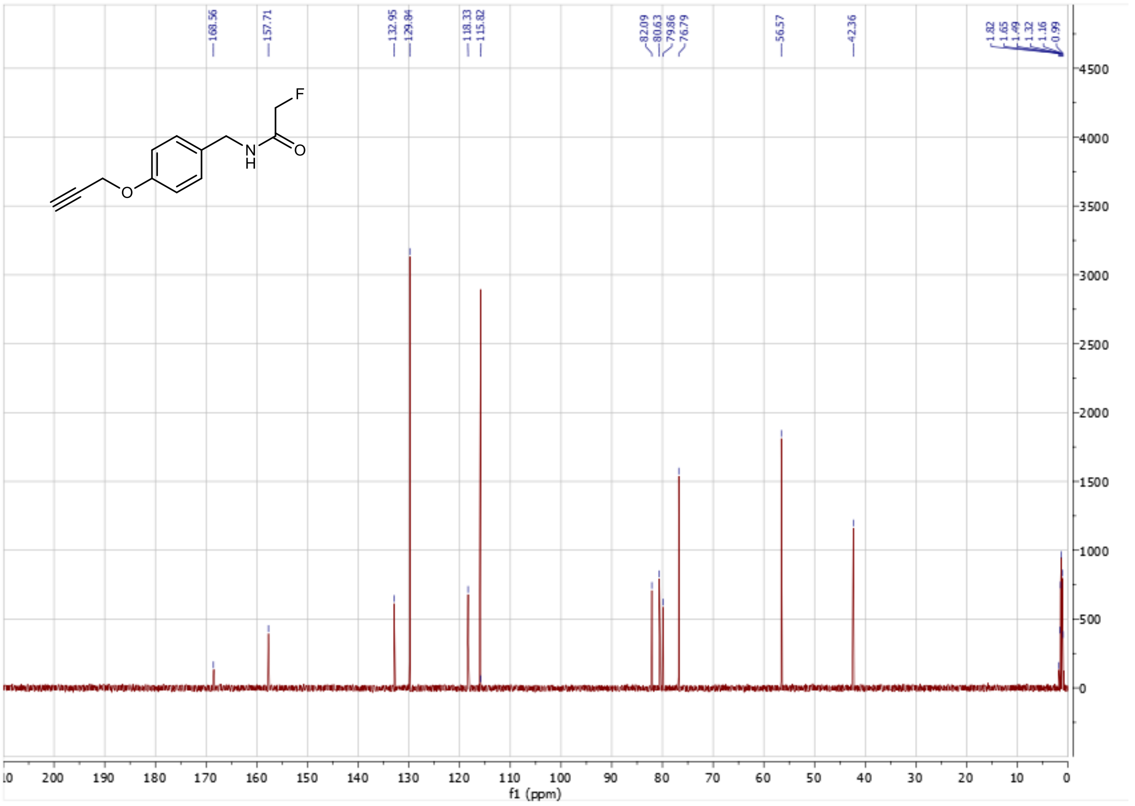

Figure S59. 2-Fluoro-N-(4-(prop-2-yn-1-yloxy)phenyl)acetamide (**20**, CD_3CN, 125MHz)

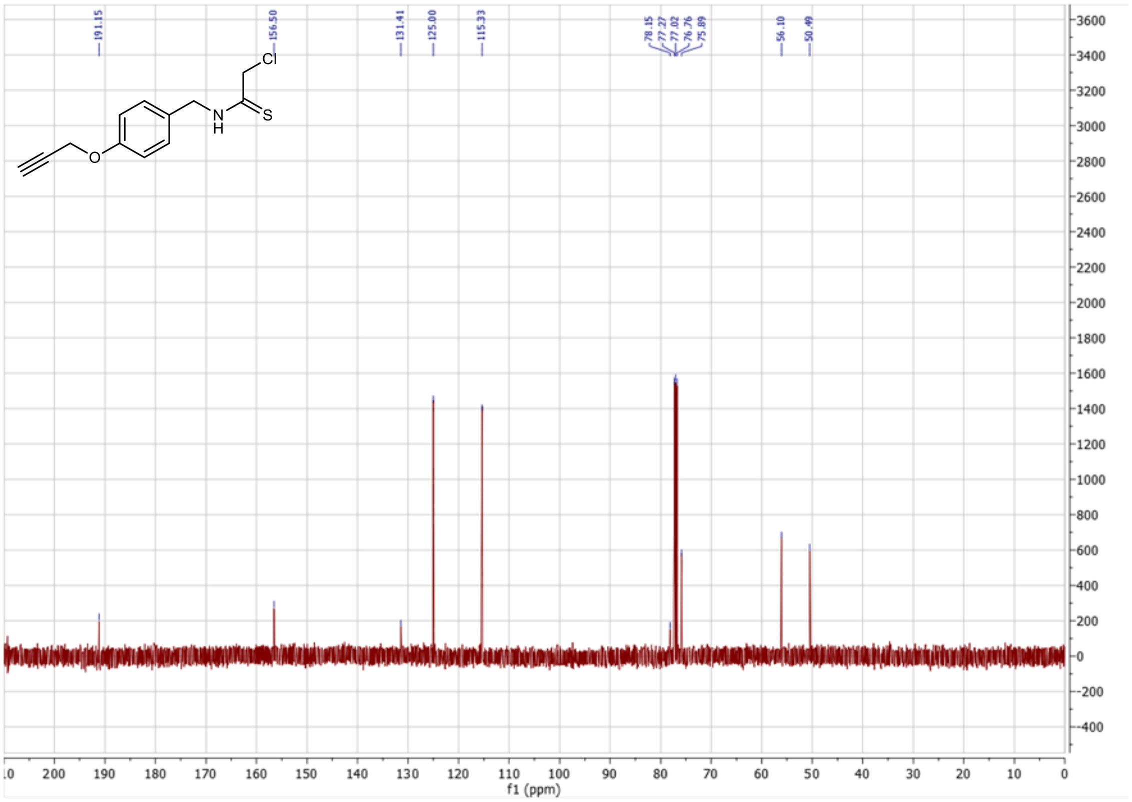

Figure S60. 2-Chloro-N-(4-(prop-2-yn-1-yloxy)phenyl)ethanethioamide (**21**, $CDCl_3$, 125MHz)

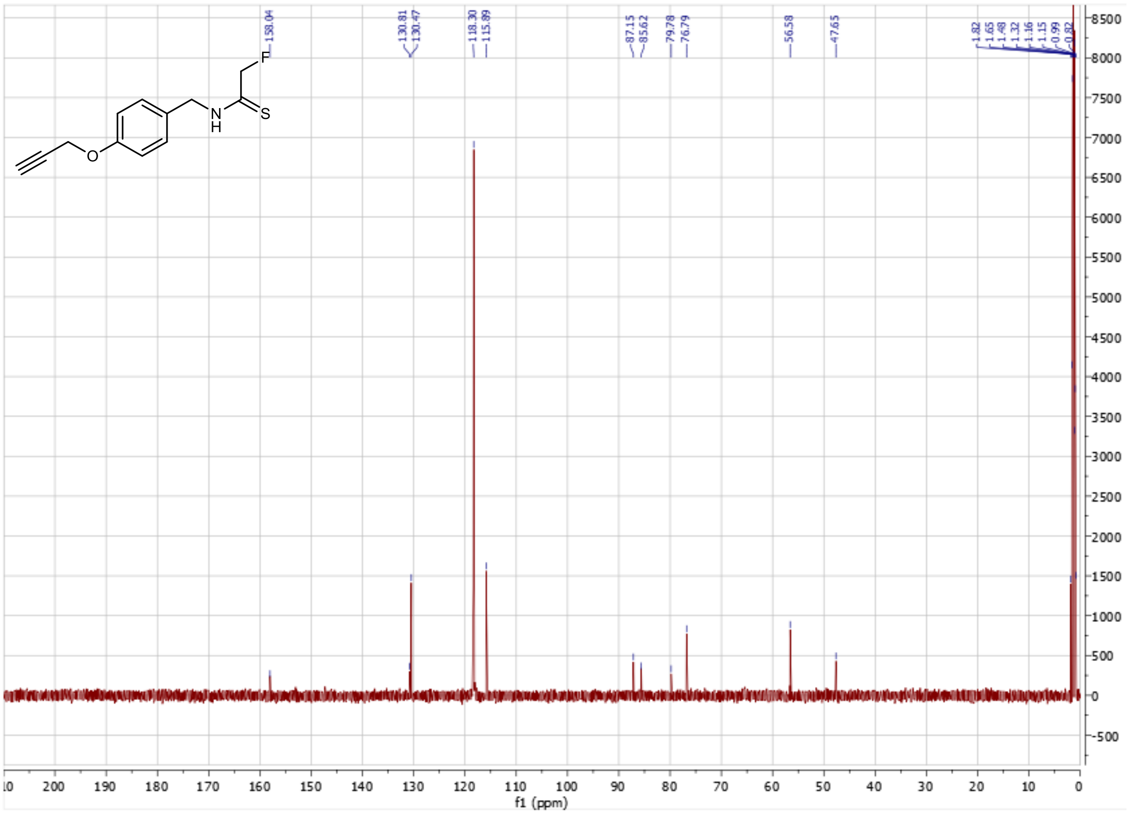

Figure S61. 2-Fluoro-N-(4-(prop-2-yn-1-yloxy)phenyl)ethanethioamide (**22**, CD_3CN, 125MHz)

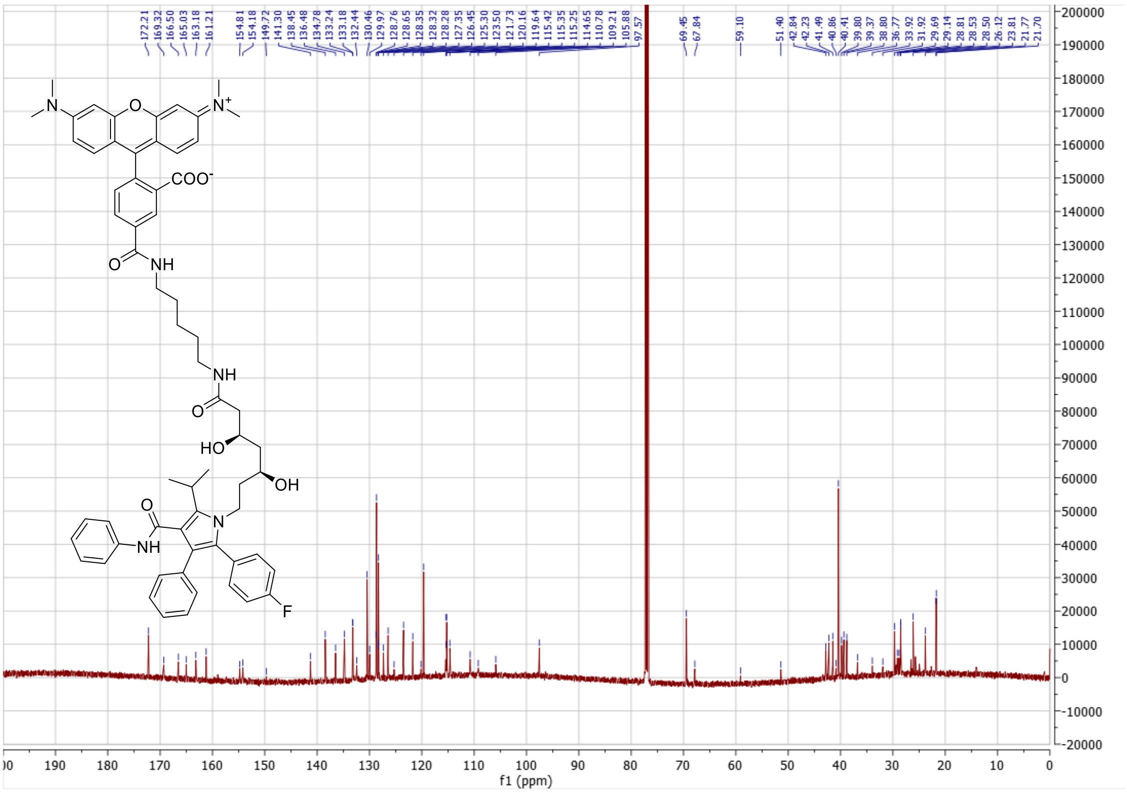

Figure S62. 2-(6-(dimethylamino)-3-(dimethyliminio)-3H-xanthen-9-yl)-5-(((5-((3R,5R)-7-(2-(4-fluorophenyl)-5-isopropyl-3-phenyl-4-(phenylcarbamoyl)-1H-pyrrol-1-yl)-3,5-dihydroxyheptanamido)pentyl)oxy) carbonyl)benzoate (**24**, $CDCl_3$, 125MHz)

HRMS spectra of the covalent probes

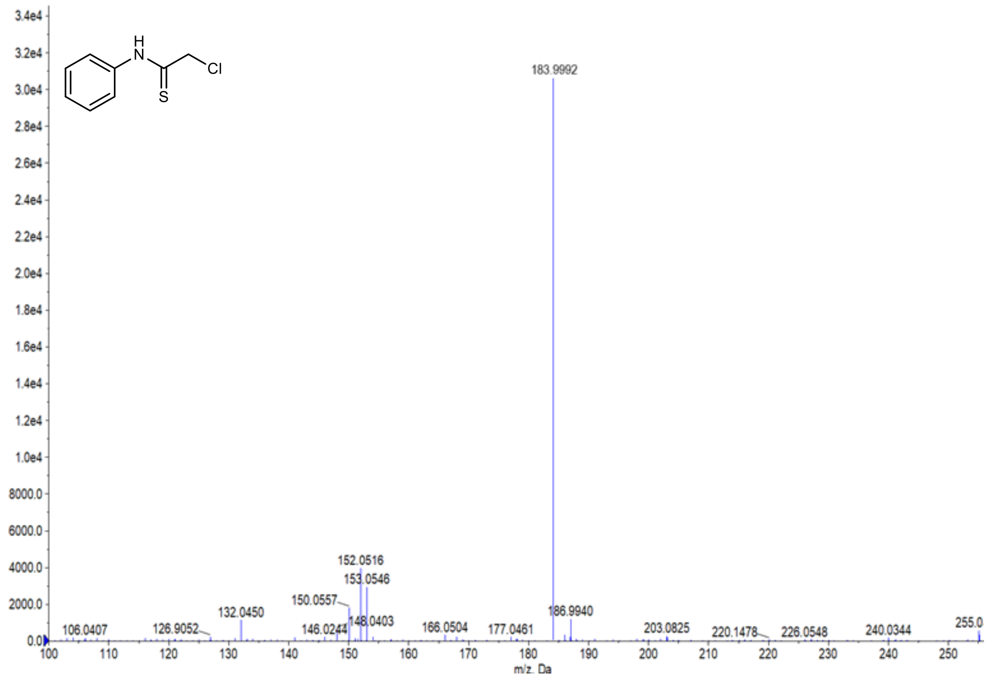

Figure S63. N-phenyl-2-chlorothioacetamide (**5a**, calc. 183.9988, found 183.9992)

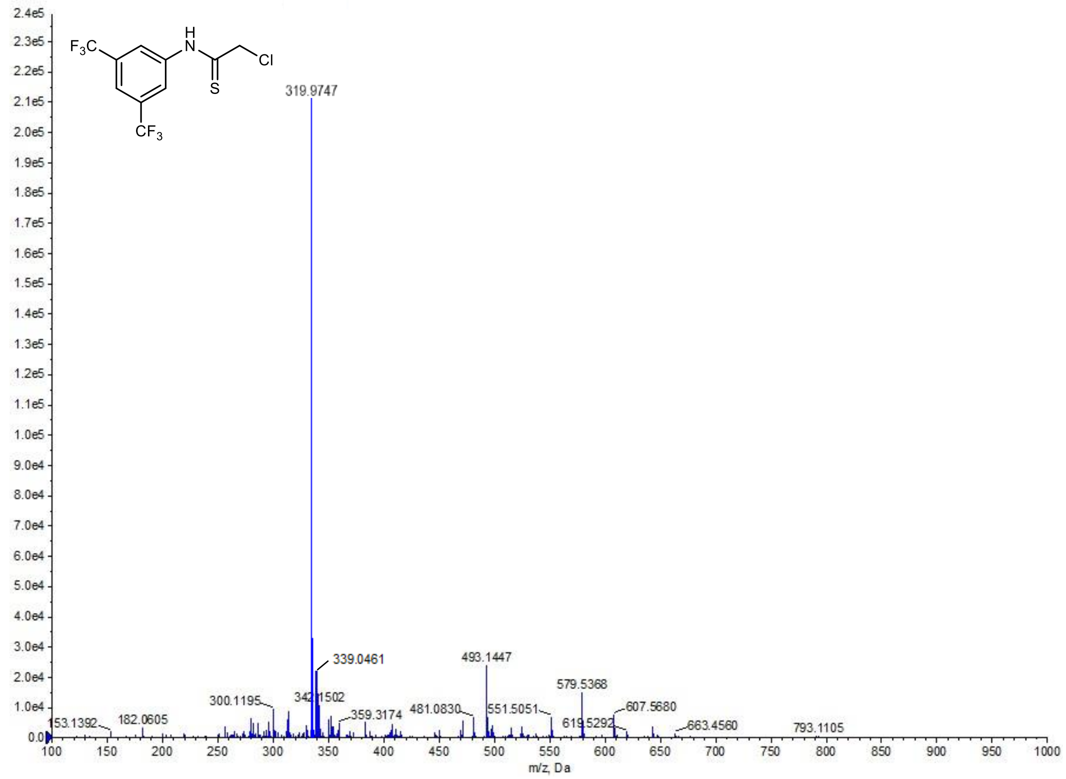

Figure S64. N-(3,5-bis(trifluoromethyl)phenyl)-2-chlorothioacetamide (**5b**, *calc.* 319.9735, found 319.9747)

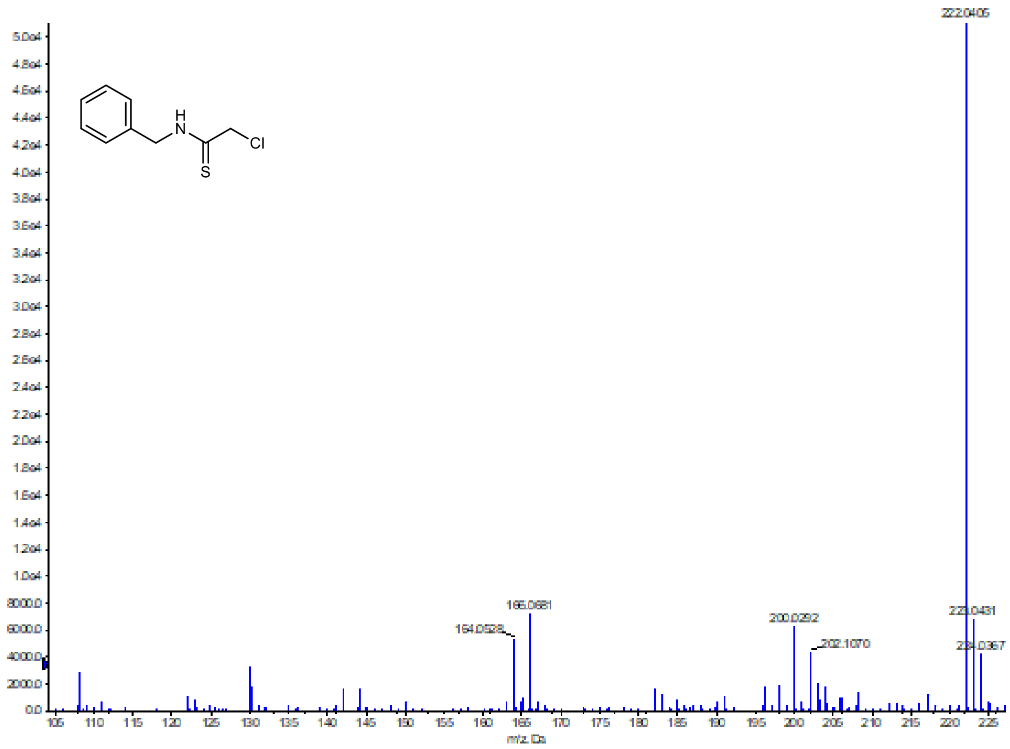

Figure S65. N-benzyl-2-chloroethanethioamide (**5c**, calc. 200.0295, found 200.0292)

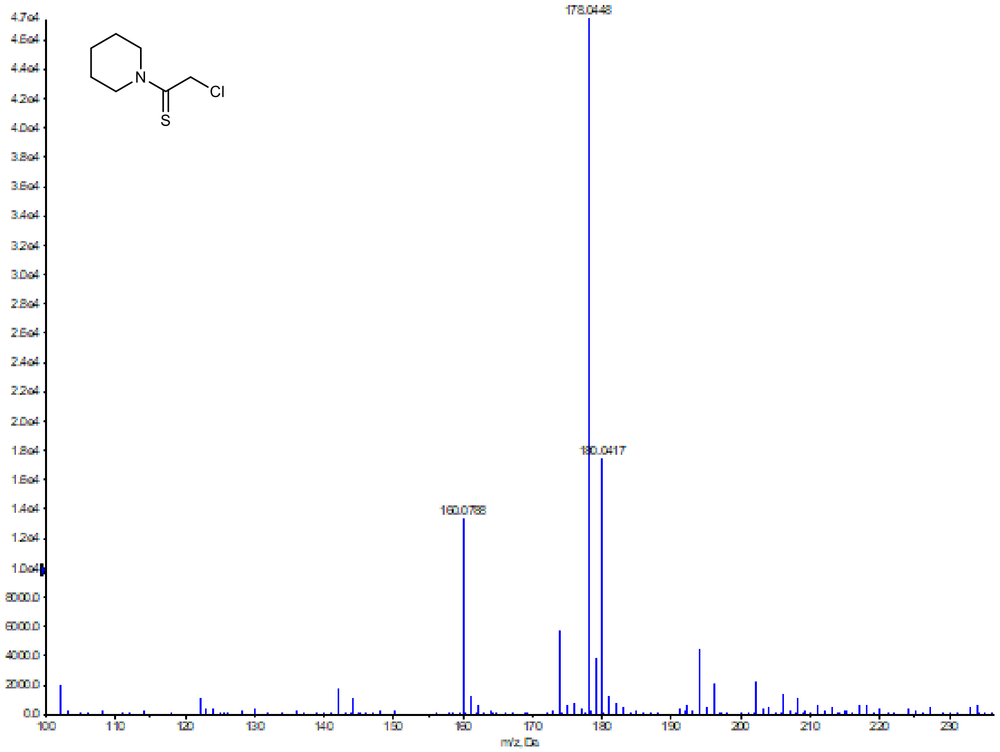

Figure S66. 2-chloro-1-(piperidin-1-yl)ethanethione (**5d**, calc. 178.0451, found 178.0448)

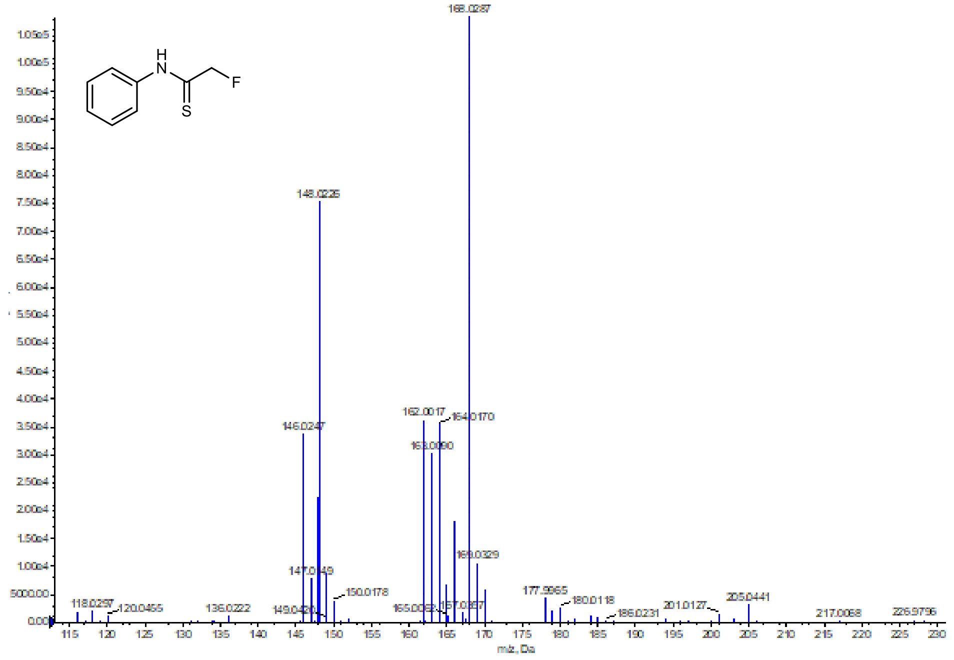

Figure S67. N-phenyl-2-fluorothioacetamide (**6a**, calc. 168.0288, found 168.0287)

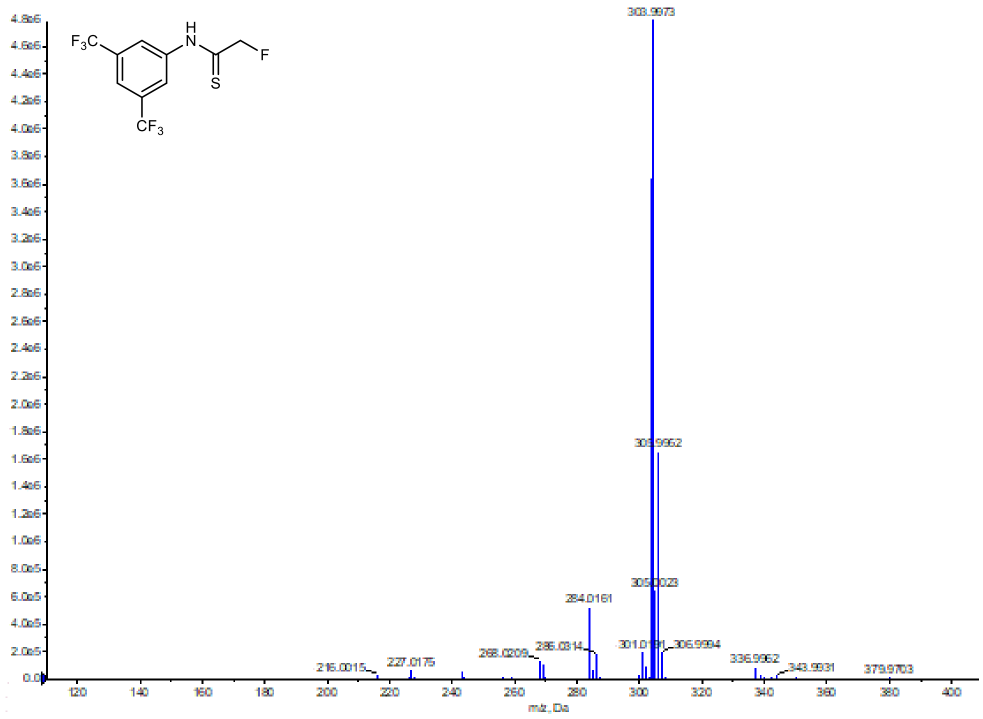

Figure S68. N-(3,5-bis(trifluoromethyl)phenyl)-2-fluorothioacetamide (**6b**, calc. 304.0031, found 303.9973)

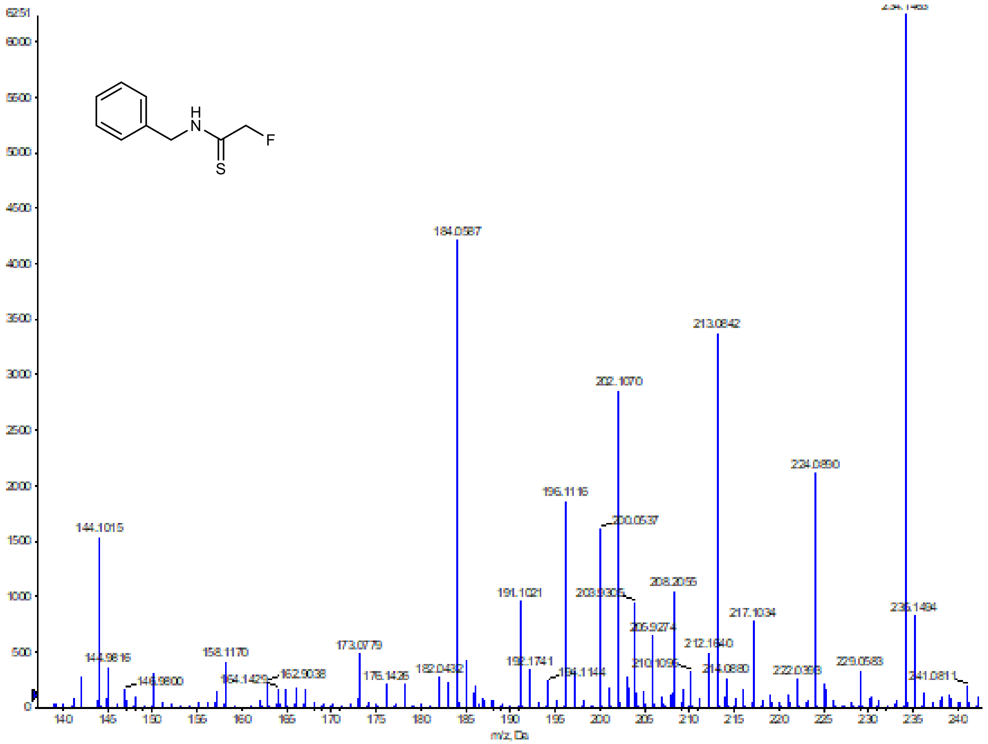

Figure S69. N-benzyl-2-fluoroethanethioamide (**6c**, calc. 184.0590, found 184.0587)

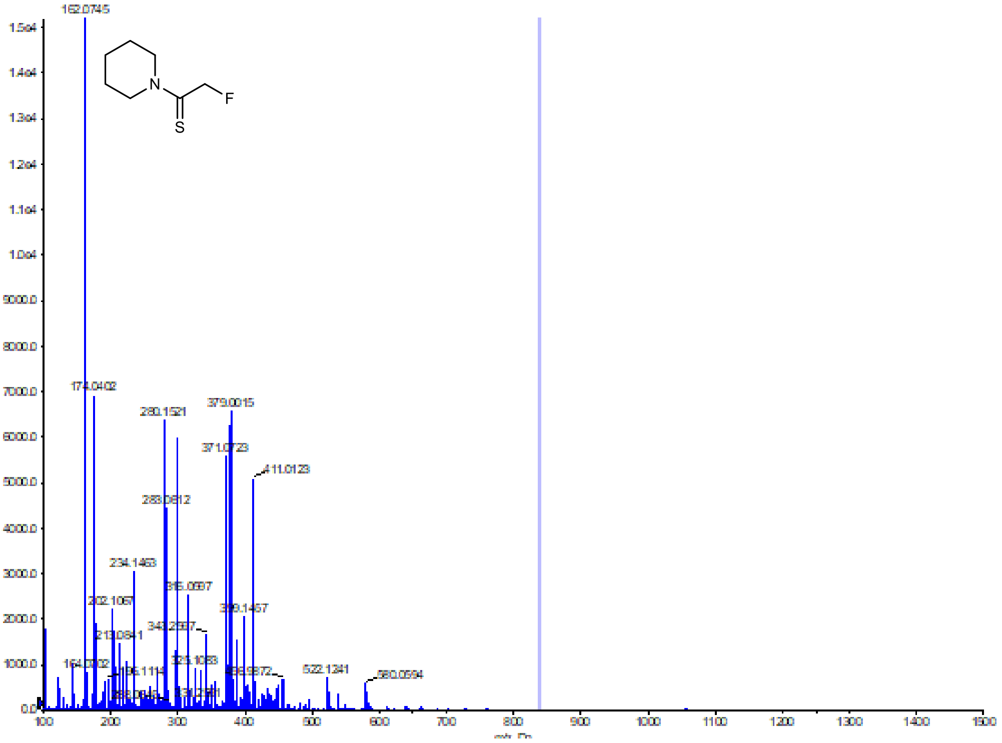

Figure S70. 2-fluoro-1-(piperidin-1-yl)ethanethione (**6d**, calc. 162.0747, found, 162.0745)

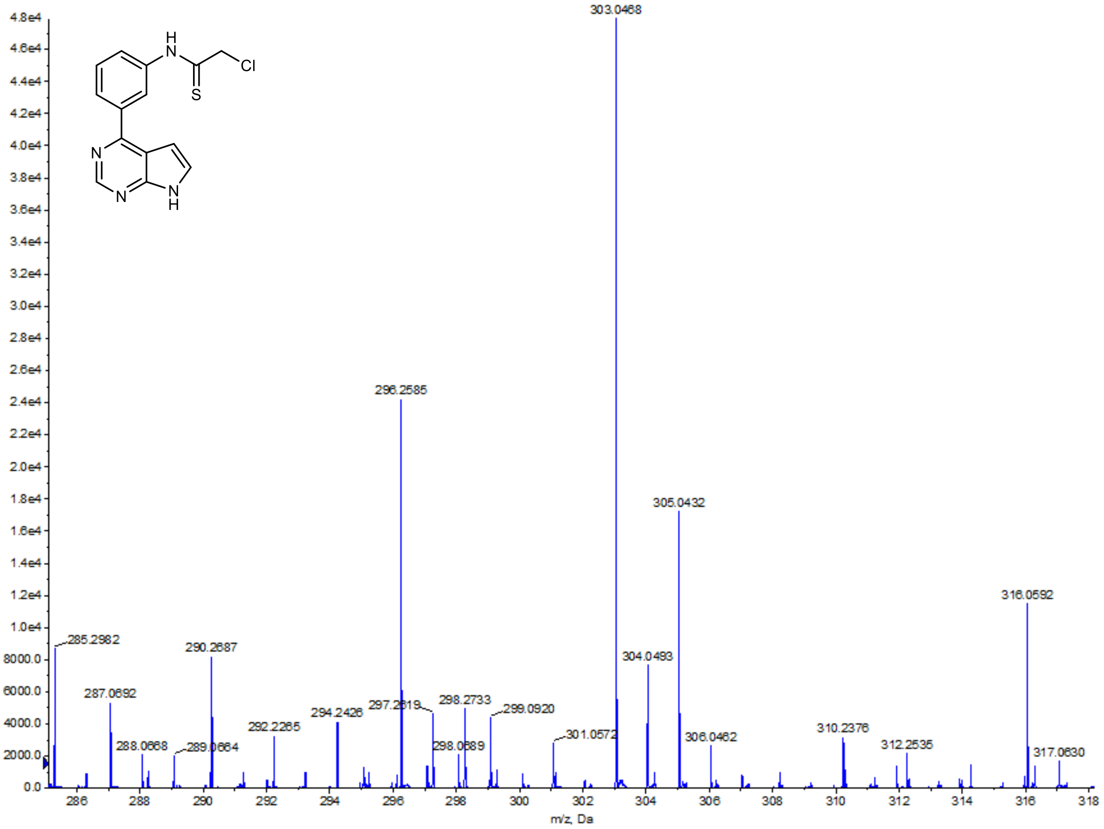

Figure S71. N-(3-(7H-pyrrolo[2,3-d]pyrimidin-4-yl)phenyl)-2-chloroethanethioamide (**9**, calc. 303.0471, found 303.0468)

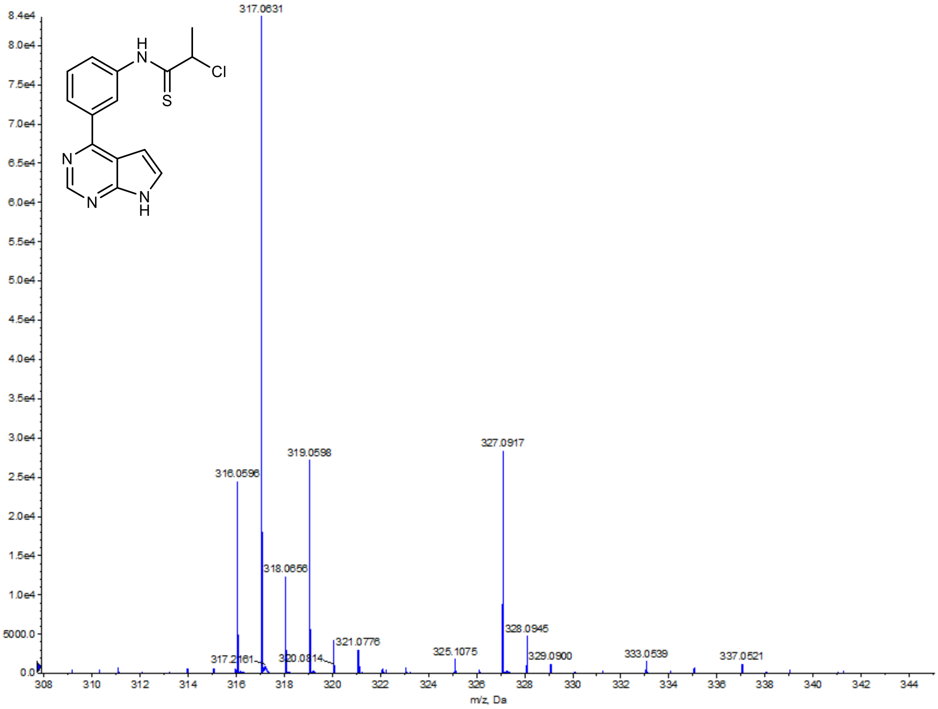

Figure S72. N-(3-(7H-pyrrolo[2,3-d]pyrimidin-4-yl)phenyl)-2-chloropropanethioamide (**10**, calc. 317.0622, found 317.0631)

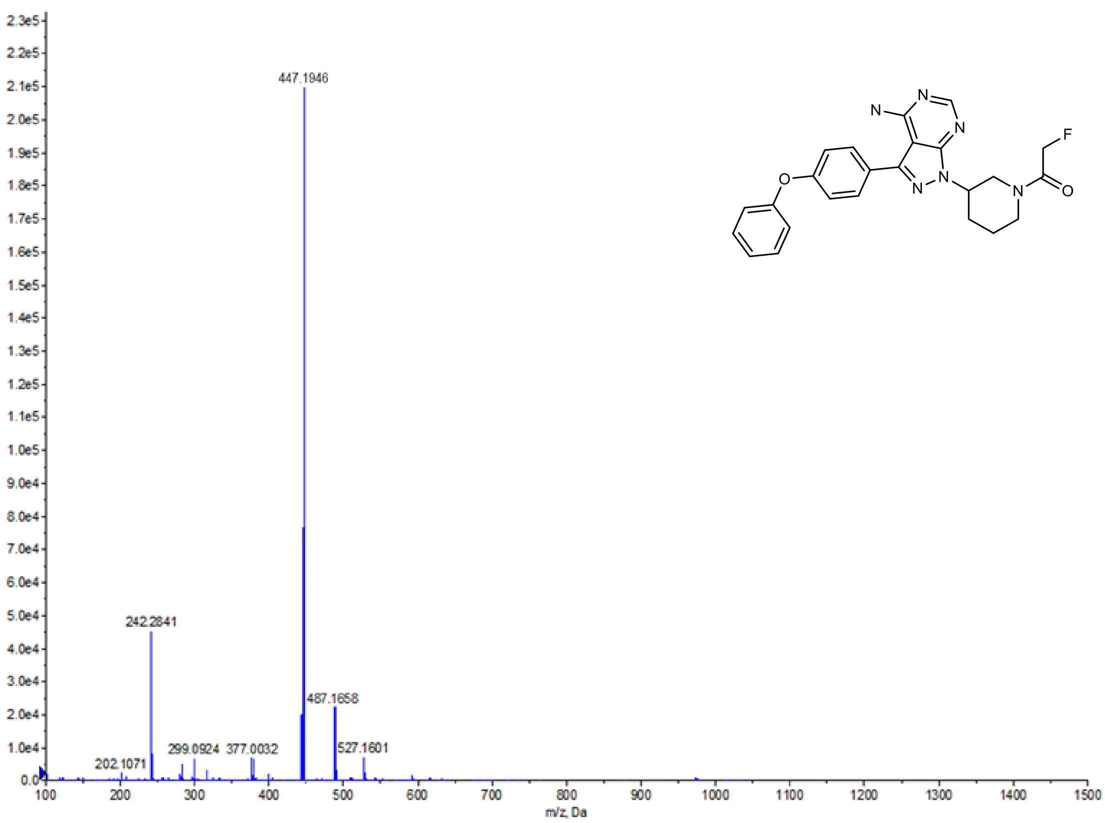

Figure S73. 1-(3-(4-amino-3-(4-phenoxyphenyl)-1H-pyrazolo[3.4-d]pyrimidin-1-yl)piperidin-1-yl)-2-fluoroethanone (**13**, calc. 447.1943, found 447.1946)

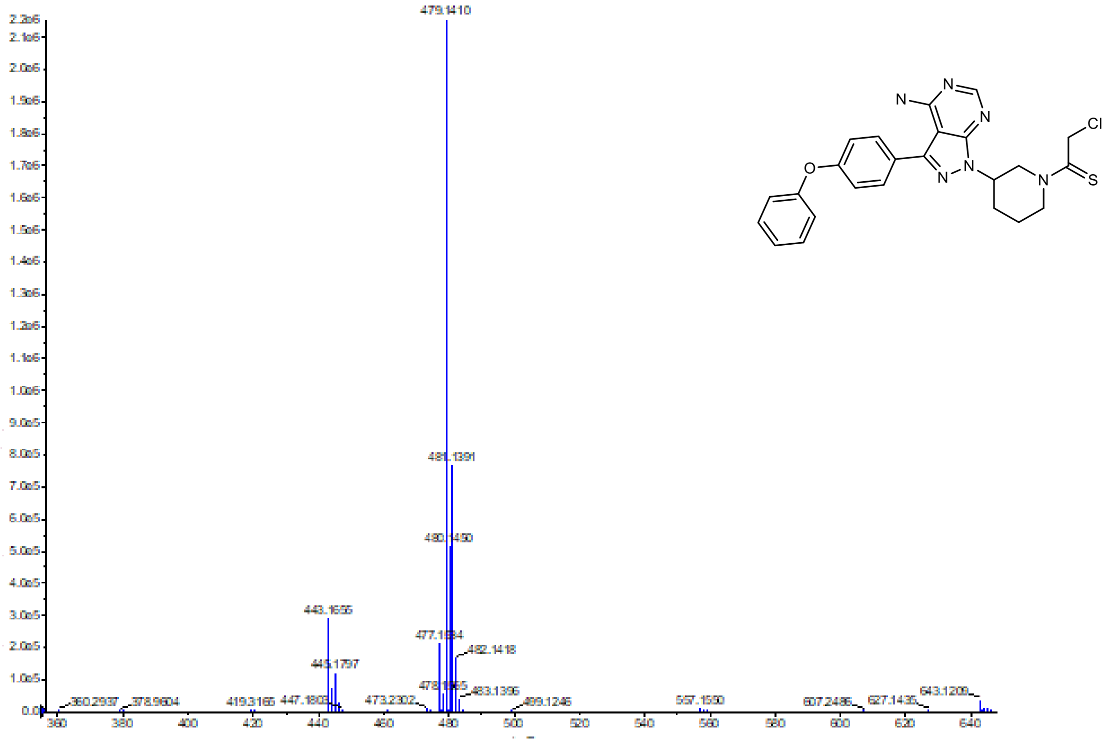

Figure S74. 1-(3-(4-Amino-3-(4-phenoxyphenyl)-1H-pyrazolo[3.4-d]pyrimidin-1-yl)piperidin-1-yl)-2-chloroethanethione (**14**, calc. 479.1420, found 479.1410)

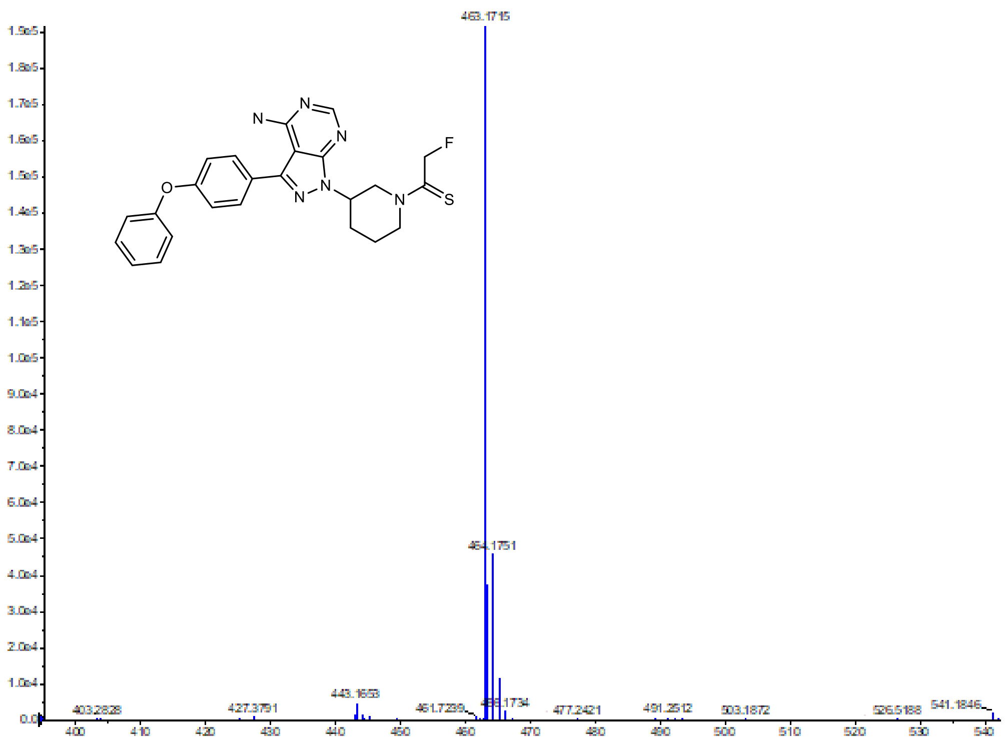

Figure S75. 1-(3-(4-Amino-3-(4-phenoxyphenyl)-1H-pyrazolo[3.4-d]pyrimidin-1-yl)piperidin-1-yl)-2-fluoroethanethione (**15**, calc. 463.1716, found 463.1715)

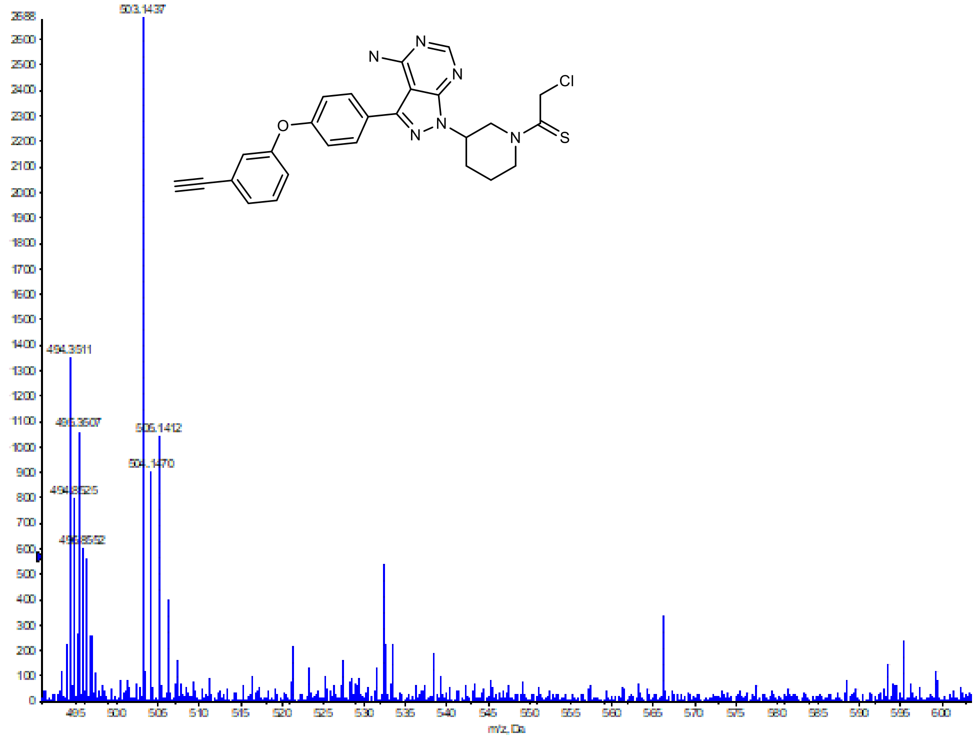

Figure S76. 1-(3-(4-Amino-3-(4-(3-ethynylphenoxy)phenyl)-1H-pyrazolo[3,4-d]pyrimidin-1-yl)piperidin-1-yl)-2-chloroethan-1-thione (**18**, calc. 503.1421, found 503.1437)

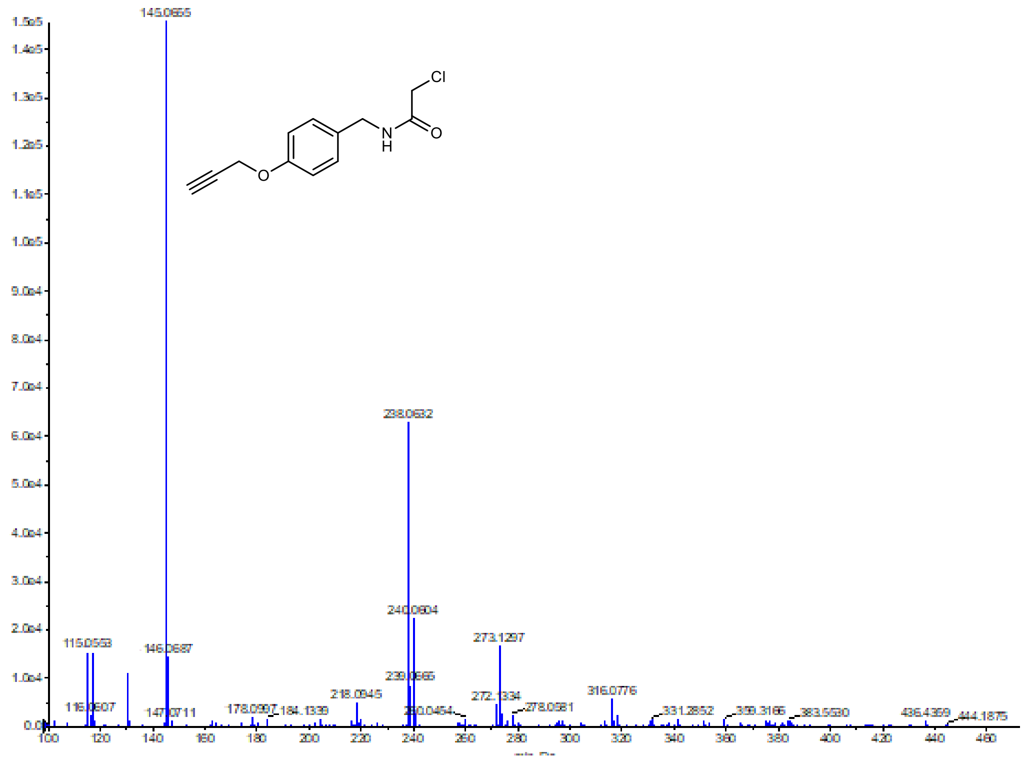

Figure S77. 2-Chloro-N-(4-(prop-2-yn-1-yloxy)phenyl)acetamide (**19**, calc. 238.0634, found 238.0632)

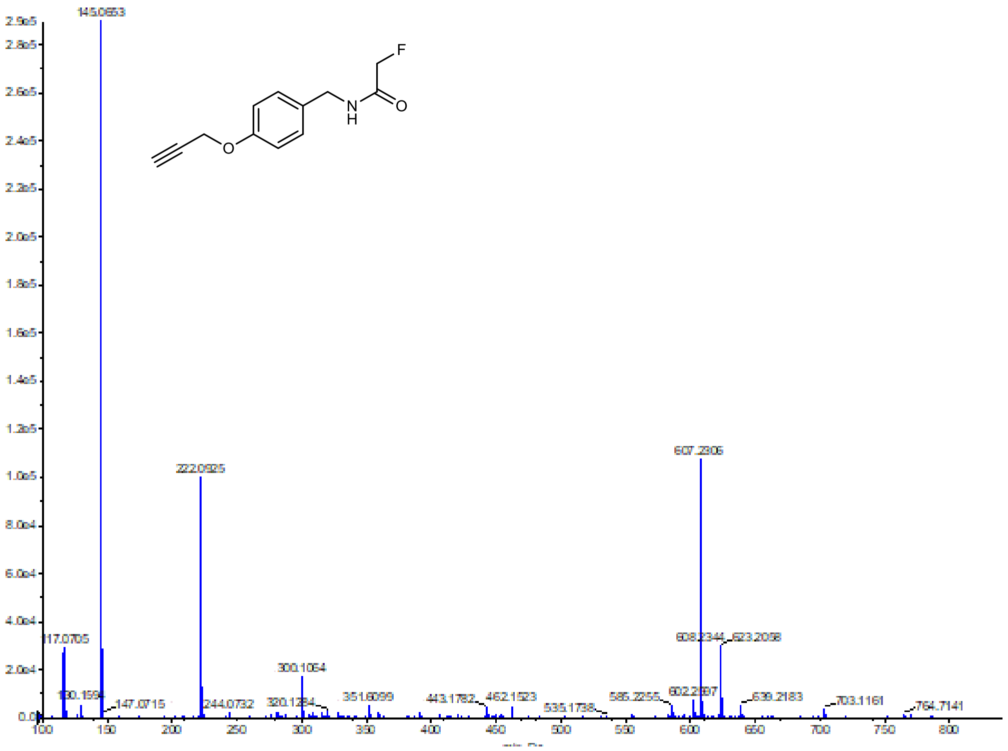

Figure S78. 2-Fluoro-N-(4-(prop-2-yn-1-yloxy)phenyl)acetamide (**20**, calc. 222.0930, found 222.0925)

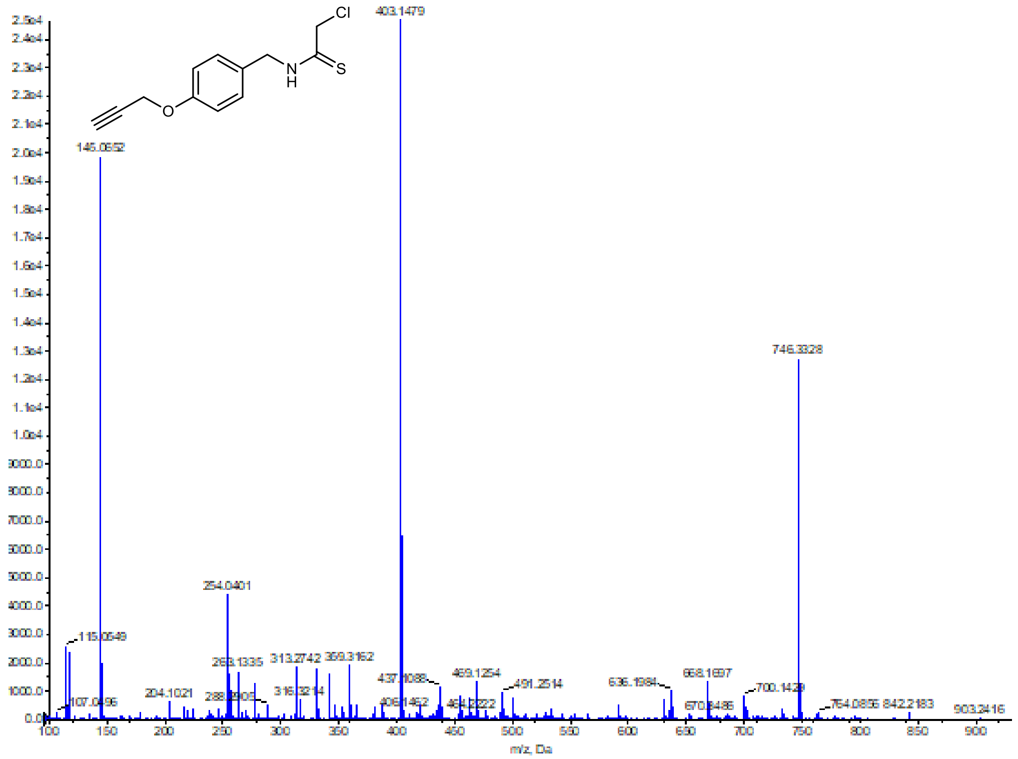

Figure S79. 2-Chloro-N-(4-(prop-2-yn-1-yloxy)phenyl)ethanethioamide (**21**, calc. 254.0406, found 254.0401)

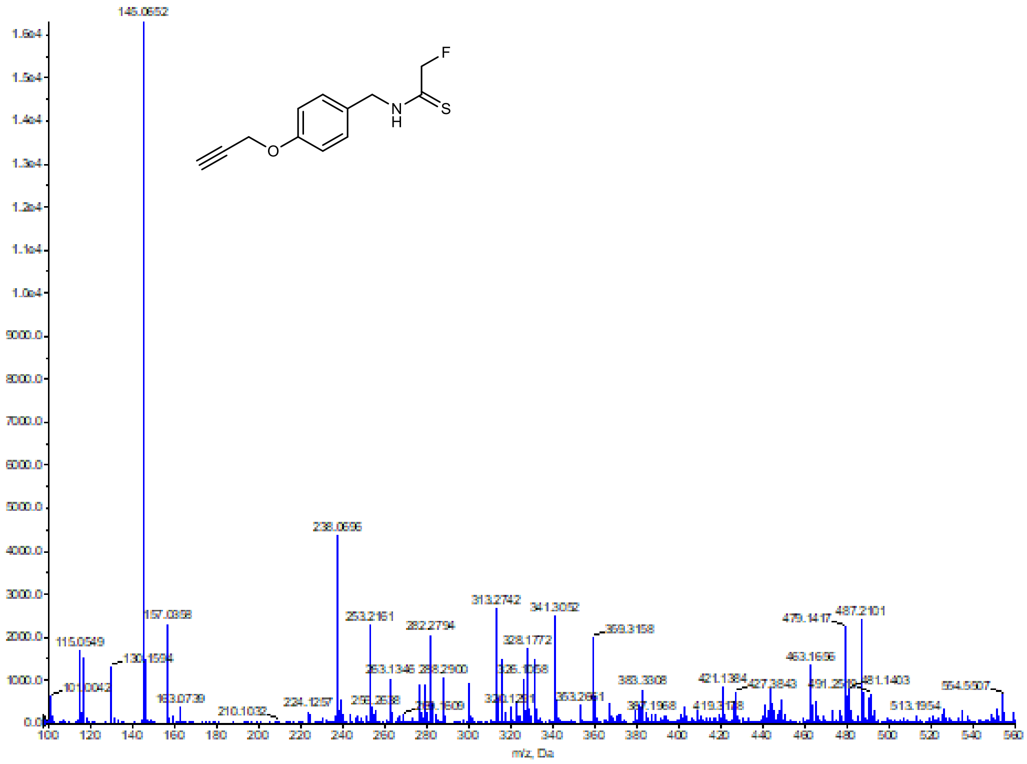

Figure S80. 2-Fluoro-N-(4-(prop-2-yn-1-yloxy)phenyl)ethanethioamide (**22**, calc. 238.0701, found 238.0696)

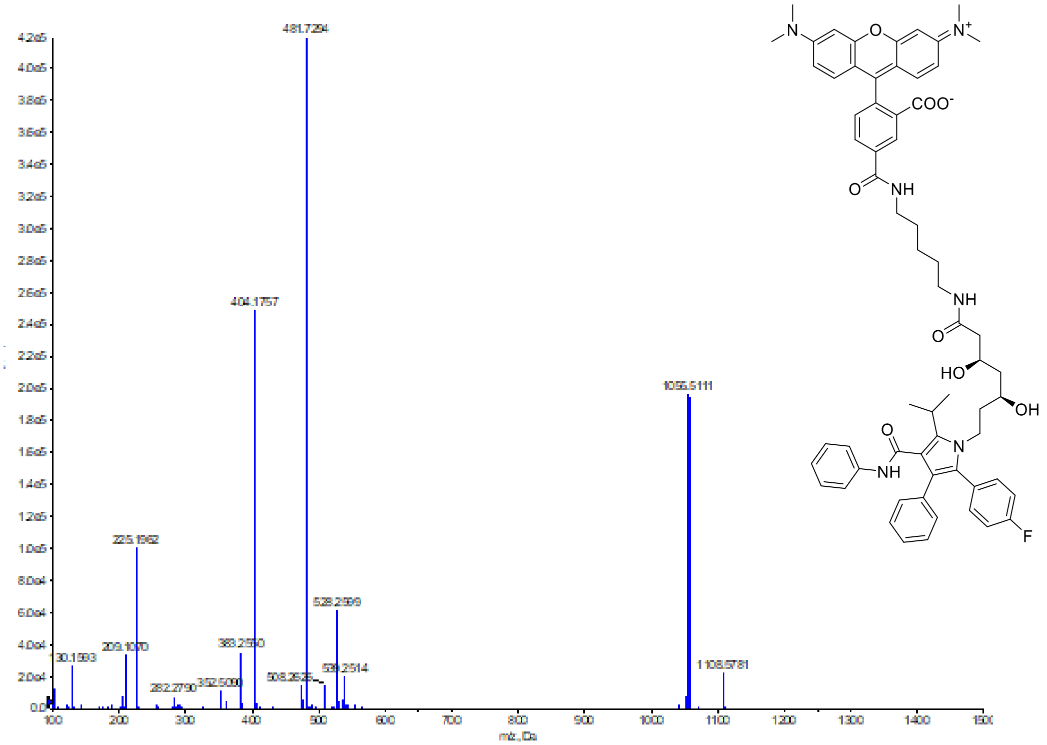

Figure S81. 2-(6-(dimethylamino)-3-(dimethyliminio)-3H-xanthen-9-yl)-5-(((5-((3R,5R)-7-(2-(4-fluorophenyl)-5-isopropyl-3-phenyl-4-(phenylcarbamoyl)-1H-pyrrol-1-yl)-3,5-dihydroxyheptanamido)pentyl)oxy) carbonyl)benzoate (**24**, calc. 1055.5077, found 1055.5111)

Uncropped SDS-PAGE gels

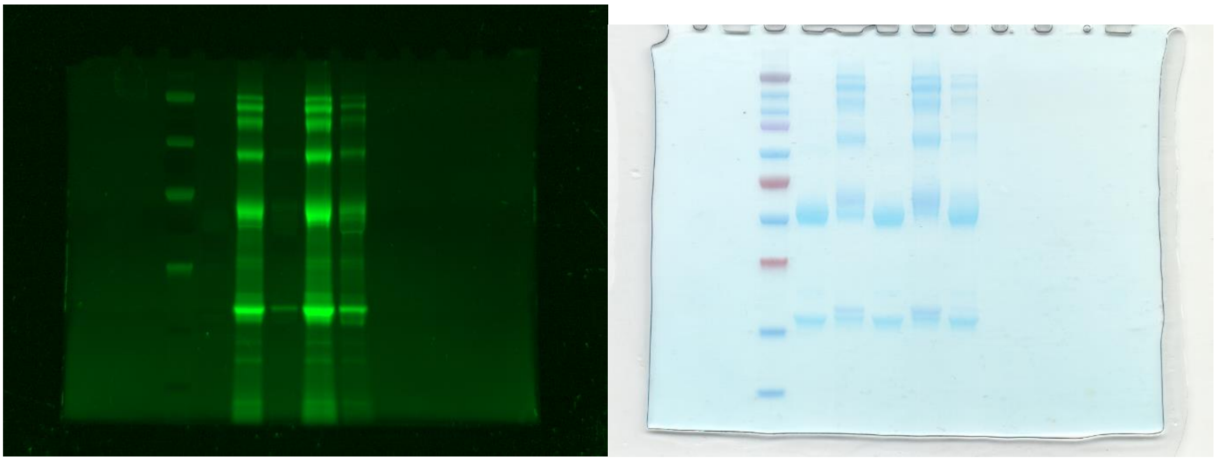

Figure S82. Uncropped gel images related to Figure 8d and Suppmenetary Figure S11.

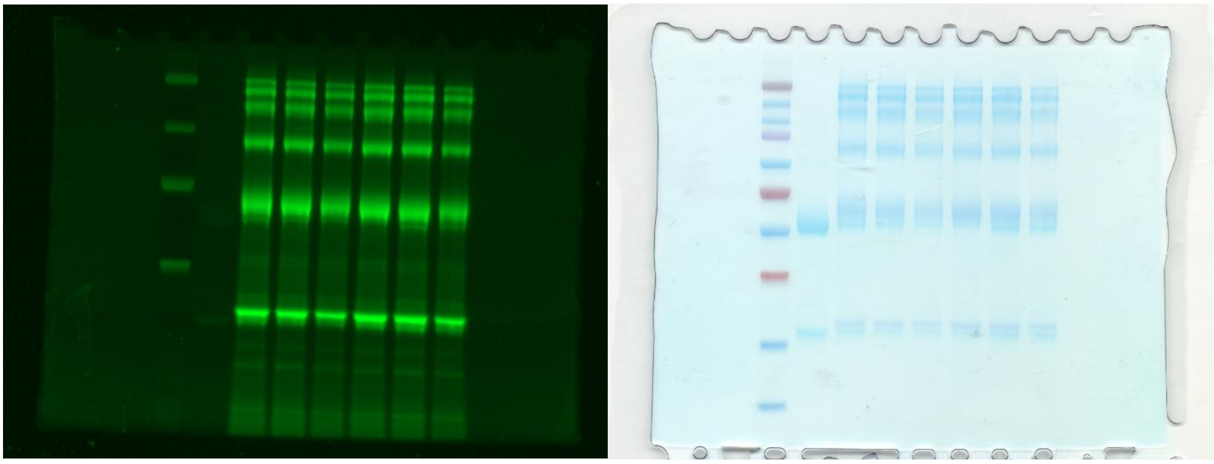

Figure S83. Uncropped gel images related to Figure 8f and Suppmenetary Figure S13.

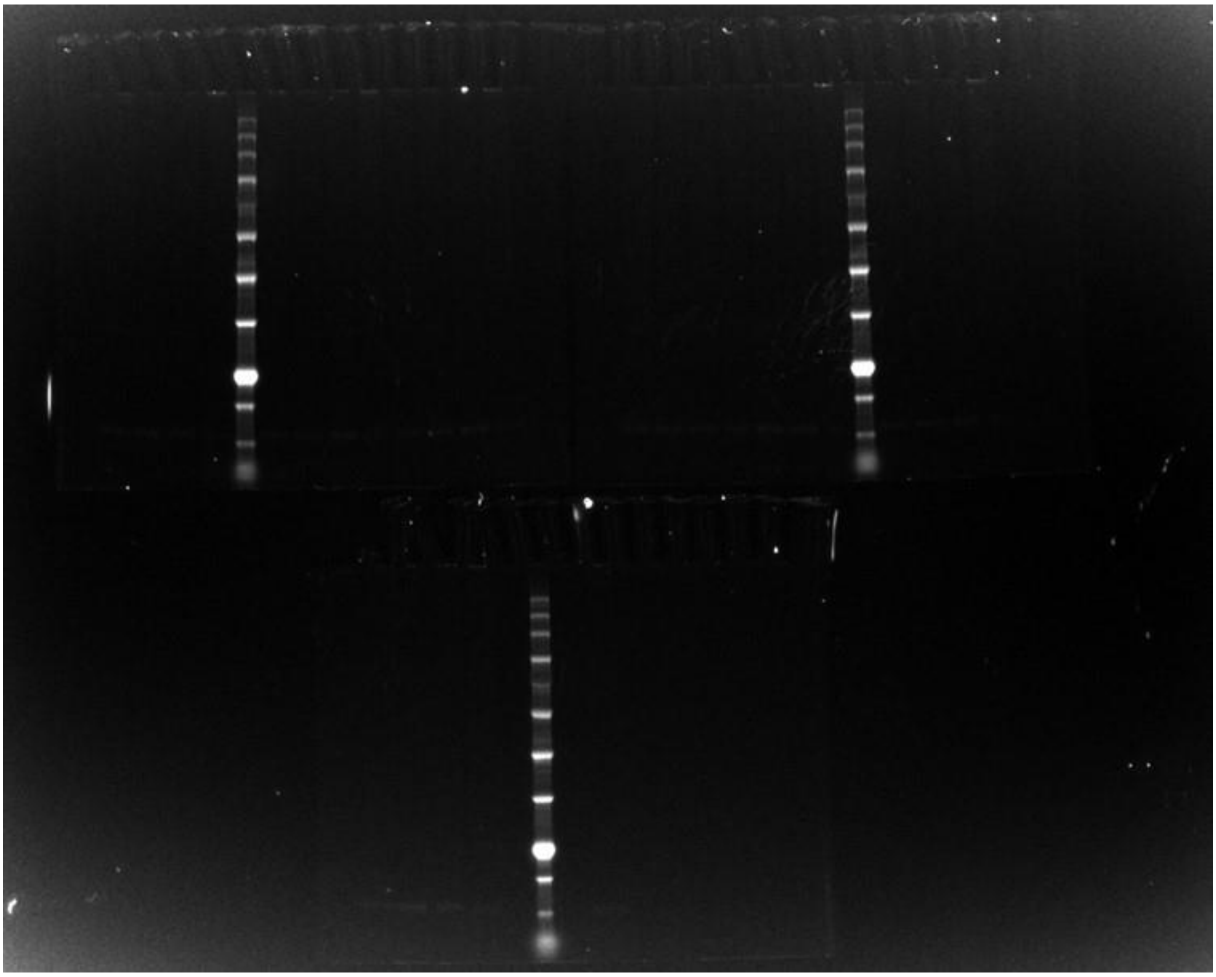

Figure S84. Uncropped gel images related to Suppmenetary Figure S14 showing Coomassie Brilliant Blue (CBB) stained gels with visible gel borders.

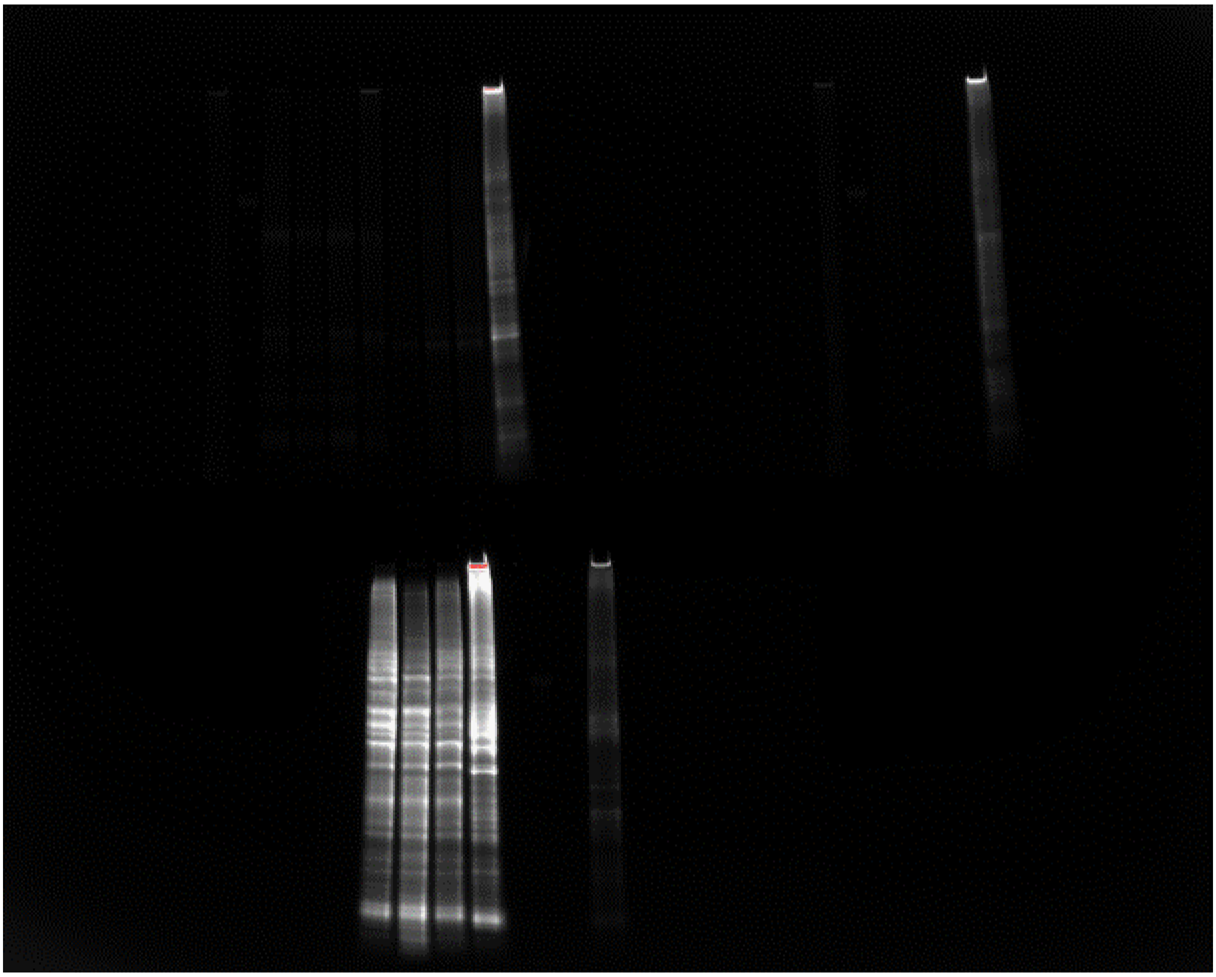

Figure S85. Uncropped gel images related to Suppmenetary Figure S14 showing fluorescent readout gels with with 10 s exposure.

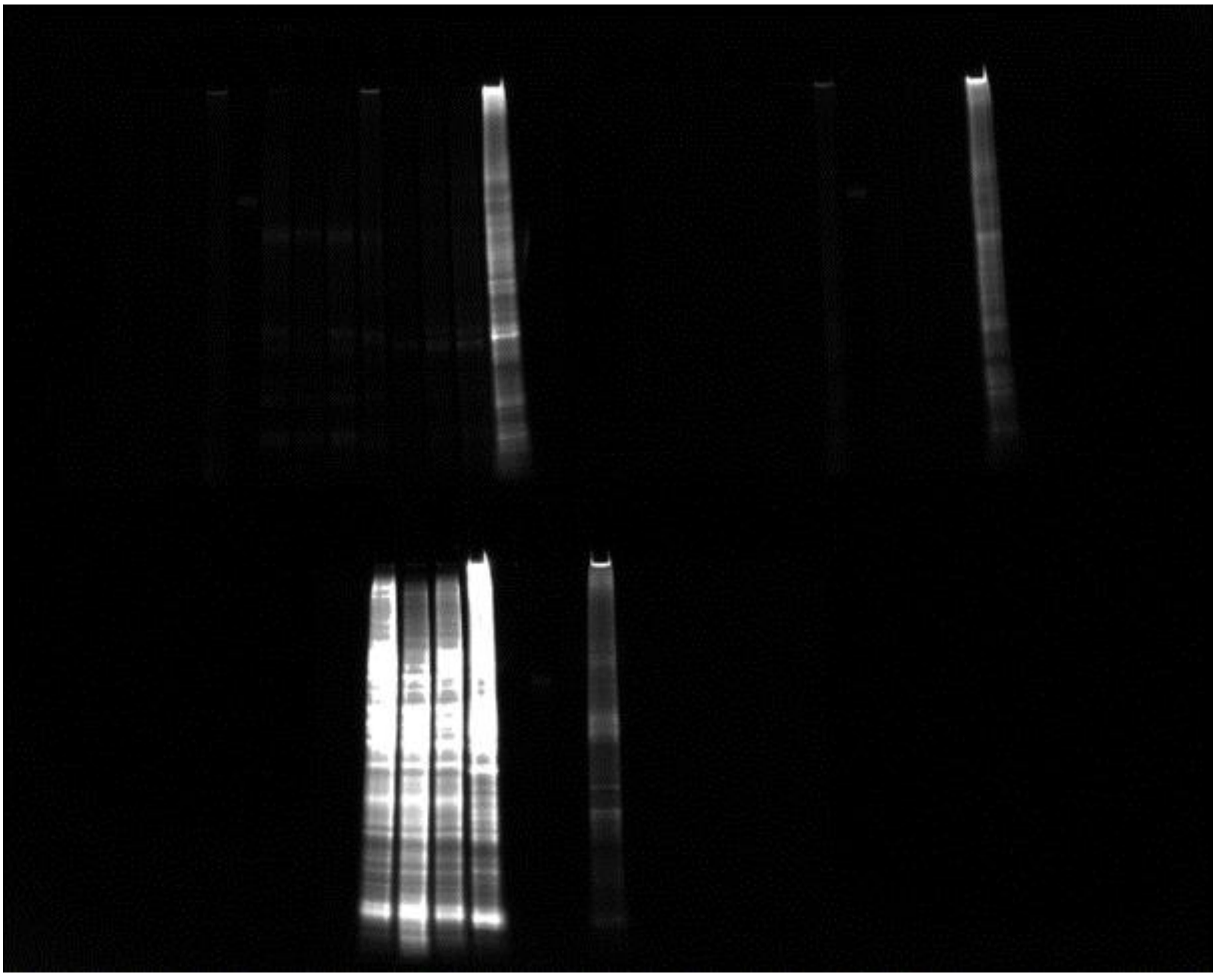

Figure S86. Uncropped gel images related to Suppmenetary Figure S14 showing fluorescent readout gels with with 60 s exposure.
