# Supplementary material for: α-Halothioamide warheads with enhanced cysteine reactivity and specificity for covalent protein labelling
Source: Nat Commun. 2026 May 14;17:6824. doi: 10.1038/s41467-026-72993-6 (PMC13388716; doi:10.1038/s41467-026-72993-6)
Supplement: Supplementary file 2 — Description of Additional Supplementary Files [file 41467_2026_72993_MOESM2_ESM.pdf]

Description of Additional Supplementary Files

Supplementary Data 1: Coordinates of the modelled molecular structures in DFT and MD. calculations.

Supplementary Data 2: The MSMS spectrum of the modified nonapeptide (NP) in selectivity assay.
