# Supplementary figures and images for: α-Halothioamide warheads with enhanced cysteine reactivity and specificity for covalent protein labelling
Source: Nat Commun. 2026 May 14;17:6824. doi: 10.1038/s41467-026-72993-6 (PMC13388716; doi:10.1038/s41467-026-72993-6)

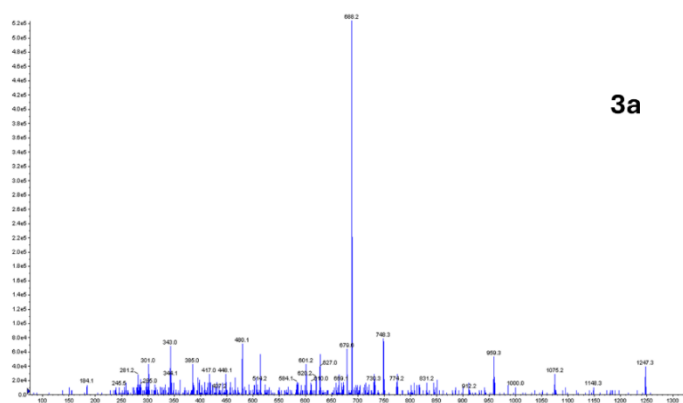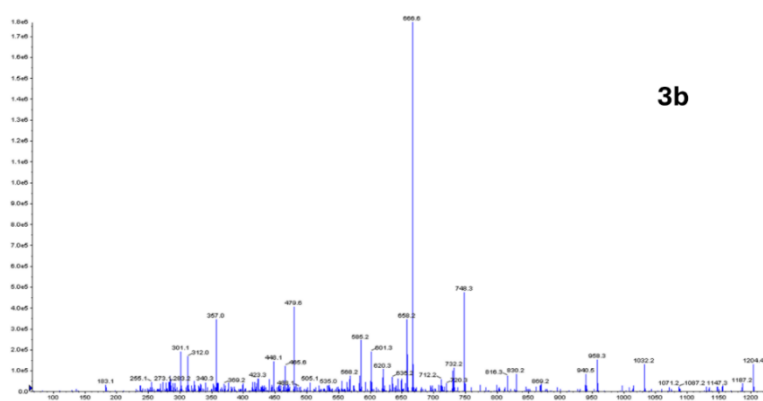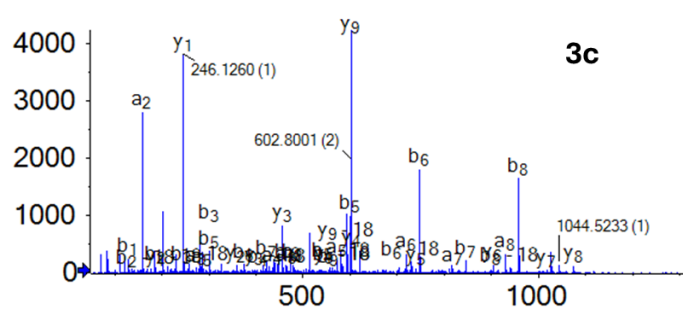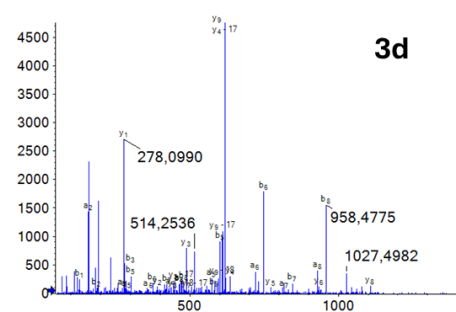

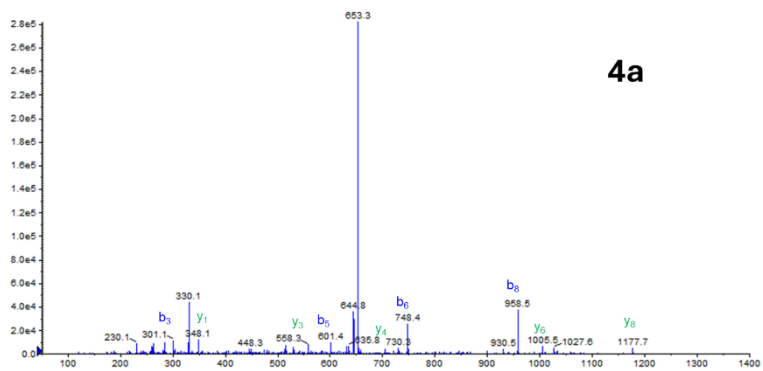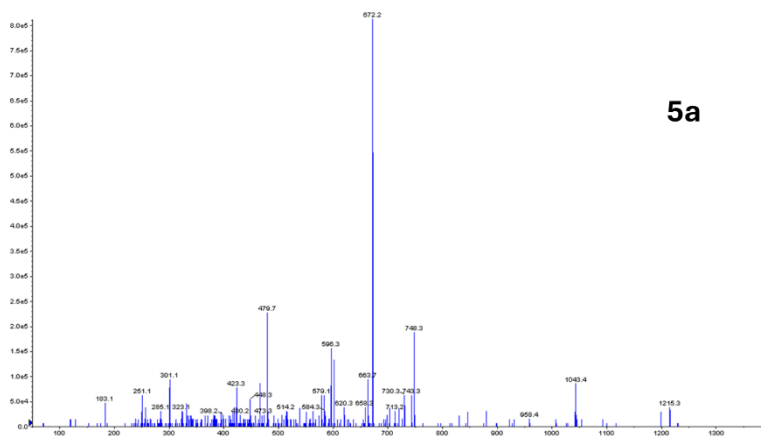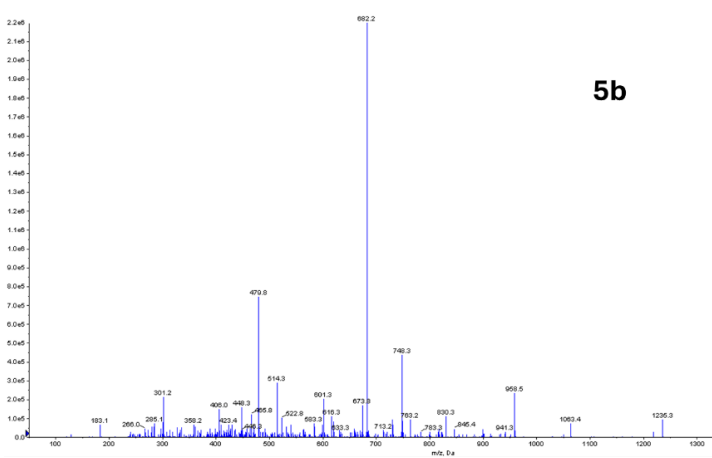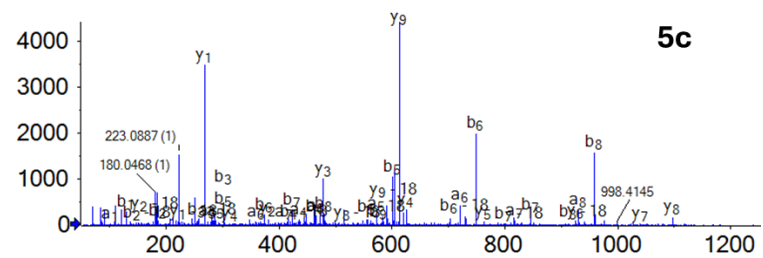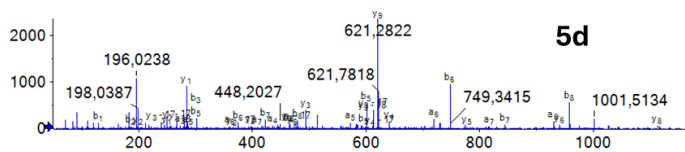

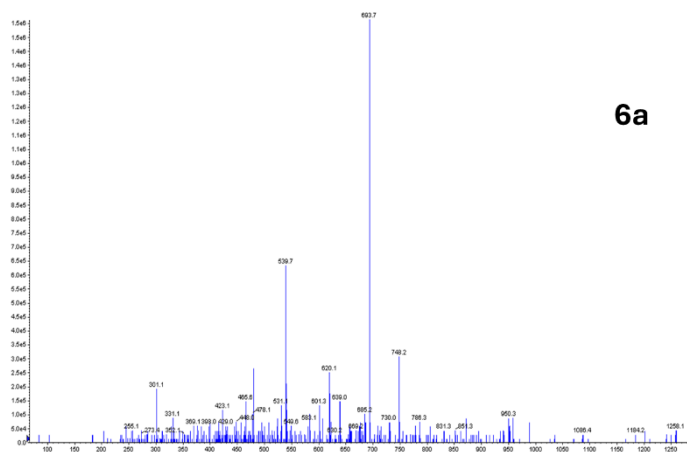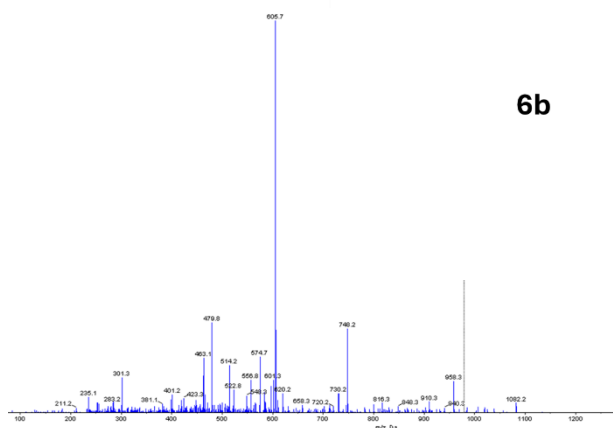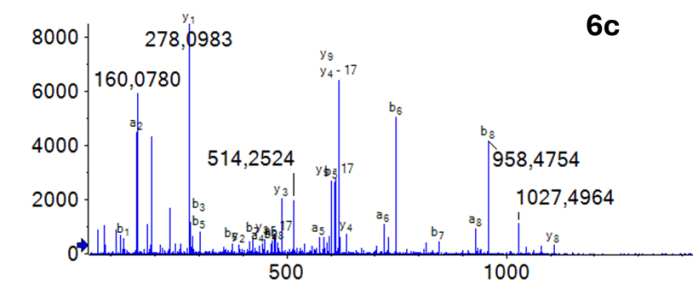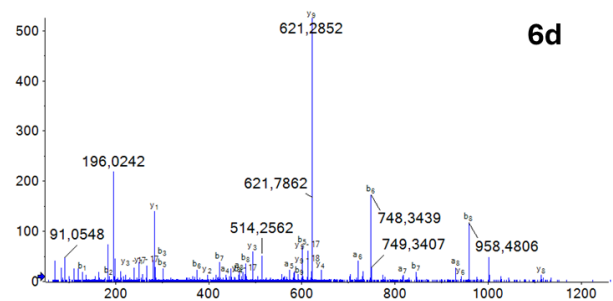

Supplement: Supplementary file 4 — Supplementary Data 2 [file 41467_2026_72993_MOESM4_ESM.pdf]
