# Supplementary material for: α-Halothioamide warheads with enhanced cysteine reactivity and specificity for covalent protein labelling
Source: Nat Commun. 2026 May 14;17:6824. doi: 10.1038/s41467-026-72993-6 (PMC13388716; doi:10.1038/s41467-026-72993-6)
Supplement: Supplementary file 5 — Reporting Summary [file 41467_2026_72993_MOESM5_ESM.pdf]

nature research

Corresponding author(s): György M. Keserű

Last updated by author(s): Feb 16, 2026

Reporting Summary

Nature Research wishes to improve the reproducibility of the work that we publish. This form provides structure for consistency and transparency in reporting. For further information on Nature Research policies, see our Editorial Policies and the Editorial Policy Checklist.

Statistics

For all statistical analyses, confirm that the following items are present in the figure legend, table legend, main text, or Methods section.

n/a	Confirmed	
☐	☒	The exact sample size (n) for each experimental group/condition, given as a discrete number and unit of measurement
☐	☒	A statement on whether measurements were taken from distinct samples or whether the same sample was measured repeatedly
☐	☒	The statistical test(s) used AND whether they are one- or two-sided<br>*Only common tests should be described solely by name; describe more complex techniques in the Methods section.*
☒	☐	A description of all covariates tested
☒	☐	A description of any assumptions or corrections, such as tests of normality and adjustment for multiple comparisons
☐	☒	A full description of the statistical parameters including central tendency (e.g. means) or other basic estimates (e.g. regression coefficient) AND variation (e.g. standard deviation) or associated estimates of uncertainty (e.g. confidence intervals)
☐	☒	For null hypothesis testing, the test statistic (e.g. F, t, r) with confidence intervals, effect sizes, degrees of freedom and P value noted<br>*Give P values as exact values whenever suitable.*
☒	☐	For Bayesian analysis, information on the choice of priors and Markov chain Monte Carlo settings
☒	☐	For hierarchical and complex designs, identification of the appropriate level for tests and full reporting of outcomes
☒	☐	Estimates of effect sizes (e.g. Cohen's d, Pearson's r), indicating how they were calculated

Our web collection on statistics for biologists contains articles on many of the points above.

Software and code

Policy information about availability of computer code

Data collection	No software was used for data collection from open-access databases.
Data analysis	DFT calculation was done using Gaussian09 software package, while TI was carried out by the Amber sofrware package. Statistical analysis and depiction of biological results was perfomed with GraphPad Prism software 8.0. MS results were processed for NP-assay by GPMAW 4.2, for protein mass spectrometry experiments Analyst TF 1.7.1, PeakView V2.2 and open-source applications by MSConvert (version 3.0.25071, ProteoWizard), SearchGUI (version 4.3.15, CompOmics) and PeptideShaker (version 3.0.11, CompOmics)., for chemoproteomics experiments Fragpipe (version 19.1) using Msfragger search engine (version 3.8), IonQuant (1.8.10121) and Philosopher (4.8.1122).

For manuscripts utilizing custom algorithms or software that are central to the research but not yet described in published literature, software must be made available to editors and reviewers. We strongly encourage code deposition in a community repository (e.g. GitHub). See the Nature Research guidelines for submitting code & software for further information.

Data

Policy information about availability of data

All manuscripts must include a data availability statement. This statement should provide the following information, where applicable:

- Accession codes, unique identifiers, or web links for publicly available datasets
- A list of figures that have associated raw data
- A description of any restrictions on data availability

Data supporting the findings of this study are available from the corresponding author(s) upon request. The data generated in this study are provided in the Supplementary Information, Supplementary Data files and Source Data file. Other proteomics data generated in this study have been deposited in the ProteomeXchange Consortium via the PRIDE partner repository under accession code PXD054470, PXD069527, PXD069336.

Field-specific reporting

Please select the one below that is the best fit for your research. If you are not sure, read the appropriate sections before making your selection.

☒ Life sciences ☐ Behavioural & social sciences ☐ Ecological, evolutionary & environmental sciences

For a reference copy of the document with all sections, see nature.com/documents/nr-reporting-summary-flat.pdf

Life sciences study design

All studies must disclose on these points even when the disclosure is negative.

Sample size

Sample sizes were selected based on the exploratory or validation-focused nature of the experiments, established practice for the respective assay types, prior experience in the field, and practical feasibility considerations, taking into account whether expected conclusions were intended to be qualitative or quantitative. Overall, replication levels were considered appropriate for the purpose of each experiment.
The GSH thiol activity assay, JAK3 and BTK kinase assays (HitHunter™), and UV–FAR analysis were conducted in biological duplicates. In the Ramos MTT assay, the reference compound was tested in five biological replicates, while new compounds were measured in biological duplicates. Immunoassays were performed without replication as they were used for qualitative assessment only. Protein mass spectrometry experiments (n = 1–2) were conducted to confirm covalent binding and no statistical analysis was demanded for data interpretation.
Proteomics experiments were performed in biological triplicates (n=3) to ensure statistical robustness.

Data exclusions

No data were excluded from this study.

Replication

The GSH thiol activity assay and kinase activity assays for JAK3 and BTK (based on the HitHunter™) assay method and the UV-FAR analyis of the antibody-dye conjugates were carried out in biological duplicates.
In the Ramos cell viability MTT assay, the reference compound was measured in five biological parallels, while the new compounds were tested in biological duplicates.
Immunoassay experiments were carried out without replication, as the assay was not intended to be quantitative and the likelihood of false-positive results was considered to be low.
Protein mass spectrometry analysis was carried out in n=1 or n=2 experiments to confirm binding of the covalent probes.
Proteomics were conducted in biological triplicates in all cases.

Randomization

No randomization was performed because all experiments were performed under controlled and standardized conditions without the use of living animals or individuals.

Blinding

No blinding was performed because all experiments were performed under controlled and standardized conditions without the use of living animals or individuals.

Reporting for specific materials, systems and methods

We require information from authors about some types of materials, experimental systems and methods used in many studies. Here, indicate whether each material, system or method listed is relevant to your study. If you are not sure if a list item applies to your research, read the appropriate section before selecting a response.

Materials & experimental systems

n/a	Involved in the study	
☐	☒	Antibodies
☐	☒	Eukaryotic cell lines
☒	☐	Palaeontology and archaeology
☒	☐	Animals and other organisms
☒	☐	Human research participants
☒	☐	Clinical data
☒	☐	Dual use research of concern

Methods

n/a	Involved in the study	
☒	☐	ChIP-seq
☒	☐	Flow cytometry
☒	☐	MRI-based neuroimaging

Antibodies

Antibodies used

Trastuzumab (MedChemExpress, Cat. No. HY-P9907) was used as a reagent for antibody-labelling experiments (not for immunodetection). The antibody was used at a concentration of 10 µM in the labelling reactions.
Fluorescein (FITC) AffiniPure Goat Anti-Human IgG (H+L) (Jackson ImmunoResearch Laboratories Inc., Cat. No. AB_2337649) was used for IHC imaging in confocal microscopy experiments. The antibody was applied at a 1:200 dilution.

Validation

Validation was provided by the supplier (https://www.medchemexpress.com/Trastuzumab.html), and HER2-targeted ELISA assays were routinely performed to confirm maintained target affinity.

Eukaryotic cell lines

Policy information about cell lines

Cell line source(s)	MDA-MB-231 (Cat. No. CRM-HTB-26), SKOV-3 (Cat. No. HTB-77), HEK293 (Cat. No. CRL-1573), Mino (Cat. No. CRL-3000) and Ramos (Cat. No. CRL-1596) cell lines were purchased from ATCC.
Authentication	The cell lines were authenticated by the supplier (ATCC) in STR profiling (results shown on manufacturer's webpages: https://www.atcc.org/products/crl-1596, https://www.atcc.org/products/crl-1573, https://www.atcc.org/products/crl-3000, https://www.atcc.org/products/htb-77, https://www.atcc.org/products/crm-htb-26) but were not independently re-authenticated in our laboratory after purchase.
Mycoplasma contamination	All cells were routinely tested for Mycoplasma contamination by Cambrex MycoAlert Mycoplasma Detection Assay Kit (Promega GmbH). Mycoplasma testing was performed every 3 weeks using MycoSPY PCR Mycoplasma Test Kit, Biontex, and all cell lines were tested negative for mycoplasma contamination. Only mycoplasma free cells were used in the experiments.
Commonly misidentified lines (See ICLAC register)	No commonly misidentified cell lines were used.
