# Supplementary material for: α-Halothioamide warheads with enhanced cysteine reactivity and specificity for covalent protein labelling
Source: Nat Commun. 2026 May 14;17:6824. doi: 10.1038/s41467-026-72993-6 (PMC13388716; doi:10.1038/s41467-026-72993-6)
Supplement: Supplementary file 6 — Transparent Peer Review file [file 41467_2026_72993_MOESM6_ESM.pdf]

nature portfolio

Peer Review File

α-Halothioamide warheads with enhanced cysteine reactivity and specificity for covalent protein labelling

Corresponding Author: Professor Gyorgy Keseru

This file contains all reviewer reports in order by version, followed by all author rebuttals in order by version.

Attachments originally included by the reviewers as part of their assessment can be found at the end of this file.

Version 0:

Reviewer comments:

Reviewer #1

(Remarks to the Author)
In this manuscript, the authors have synthesized warheads and explored their application in labeling Bruton's tyrosine kinase (BTK). My general perception is that this manuscript is better suited for a journal specialized in Medicinal Chemistry, as it involves synthesis, computational methods, and biochemical evaluation of the compounds. For this reason, I cannot recommend its publication in Nature Communications.
Major Points
a) The manuscript does not fit within the scope of the journal.
b) There is no urgency to publish it as a communication.
c) The computational section describes the reaction in solution; however, kinetics should take into consideration the enzymatic environment.
d) The design of the compounds is not based on computational methods. The authors wrote: "Since the steric and electronic effects of the substituents might change the non-covalent recognition of the scaffold, we wondered whether the isosteric O-S exchange of carboxamides is useful for tailoring the reactivity of the warhead." The computational section should be improved to better describe the enzymatic environment and the design of warhead.

Reviewer #2

(Remarks to the Author)
Covalent strategies have become increasingly important in drug discovery and chemical biology. There is a need for expanding the toolbox of covalent warheads. This study seeks to expand the scope of haloacetamide and acrylamide warheads by transitioning to analogous thioamide compounds, which modulates reactivity while having only a minor impact on reaction geometries. The approach has been validated using fragments in reactivity assays, DFT calculations, a kinase fragment screen, and by applying the electrophiles to a targeted covalent inhibitor (e.g., Ibrutinib), antibody-drug conjugation and in a chemoproteomic profiling. Overall, this is a simple yet very good idea, yielding interesting results. However, the manuscript feels somewhat incomplete. The discussion often appears superficial and not very nuanced, and significant additional experiments are needed to meet the level of rigor expected in a high-impact journal such as Nature Communications.

Therefore, I strongly recommend publication of the manuscript in Nature Communications on the condition that the requested experimental data are supplemented in a major revision or resubmission of the manuscript.

The following points should be considered:

Major:

- How did the authors unambiguously distinguish between the respective labeled amino acids in the peptide assay? Please specify in the body of the manuscript and provide data. The same applies to protein labeling.

- The kinase inhibition assays on page 6 require additional data for validation of the findings. Importantly, kinase inhibition tends to show a weak correlation with covalent modification determined by mass spectrometry as suggested for example by kinome screening data recently published (doi 10.1002/anie.202419736). Therefore, the kinase inhibition data must be complemented with intact protein MS data for all probes/proteins as a more rigorous measure of protein reactivity.

- The degree of intact protein labeling and stoichiometries need to be quantified and discussed for all tested probes and proteins (see comment above).

- The initial reactivity study does not include any alkyl amine-derived thioamide electrophiles, so it is unclear how the aniline-derived electrophiles from the first part of the study compare to the piperidine-based ones used in the second part. These two compound classes should be ranked against each other. I strongly suggest synthesizing benzylamine and piperidine-derived sets of halothioamide/thioacrylamide fragments and testing these in the GSH assay for comparison. It would also be important to show if the decreased reactivity commonly observed for N-alkyl acrylamide versus N-aryl acrylamide transfers to thioacrylamide.

- If the piperidine-derived thioacrylamide proofs to be significantly less reactive than the above aniline derivatives, this electrophile should be included in the TCI part of the study.

- TCI part, kinase inhibition profiling: For rigorous characterization of covalent inhibitors, especially if different warheads/electrophiles are to be compared, the contributions of non-covalent and covalent binding need to be deconvoluted (i.e., KI and kinact). Kinetic assays are now readily available for BTK, and such data must be complemented accordingly. The fact that probe 14 does not seem to modify the enzyme at all but still has a relatively potent IC50 of 45 nM should also be discussed. It seems that the covalent mode of action does not contribute much to the observed activity, in line with the slow labeling kinetics shown in the supplementary information. Interestingly, probe 13 shows very slow labeling kinetics in MS but higher potency than 12 and Ibrutinib, which have much faster labeling kinetics. This suggests that the observed boost in activity is due to non-covalent interactions. This further supports the critical need for kinact and KI determination for these derivatives and a more elaborate discussion of the results.

- The ADC part of the study is interesting, but the limited stability of the linkage and loss of cargo is a common problem encountered in ADCs. It is suggested to conduct experiments to prove that the bond is indeed stable under physiologically relevant conditions.

- Considering the potential application of these thioamide-derived warheads in TCIs, and the known susceptibility of sulfur to oxidation, some investigation of metabolism, at least in vitro, would be a beneficial addition to the data presented.

Minor Points:

- Title/manuscript: "Thioamide warhead" should be rephrased to "thioamide-derived warhead" or similar as thioamide itself is not the warhead functional group.

- Page 2: "were mostly avoided from drug discovery pipelines due to potential idiosyncratic toxicity issues" could also mention that lack of selectivity was often a factor associated with covalent drug modalities.

- Page 2: "the different reactivities of warheads" could mention that warhead topology, sterics, and polar features also play a role.

- Page 2: Instead of "electrophile without changing its geometry," use "electrophile without significantly changing its geometry."

- In the introduction, the authors may discuss the reaction types more clearly to provide context. Notably, the C=S bond is not directly involved in the displacement of alpha leaving groups. Although the reasoning sounds logical, the impact of the C=S bond on the softness of the beta-position in thioacrylamide electrophiles should be discussed with suitable citations or backed by own data.

- Page 3: "Isothiocyanates (4a-b) were reacted with vinylmagnesium bromide (5) Grignard-reagent (Scheme 1b), while 6a was obtained in sufficient purity," needs rephrasing. Please mention the warhead chemotype.

- Table 1 and discussion: Conditions (pH, solvent, temperature, concentrations) should be mentioned. Notably, it seems phenylacrylamide, a common standard, behaves relatively unreactively here (GSH t1/2 is often cited in the range of ~1-3h). It should also be mentioned if the depicted values are corrected for degradation or not.

- Page 3: "Our results confirmed that O-S exchange increased the thiol-reactivity," is an overgeneralization. While the GSH data support this statement for AA/TAA, differences are fairly minor for the CA/CTA and FA/FTA matched pairs; CTA with

R=H has essentially the same stability as its CA analog. Please refine this statement.

- Page 3: "seen for the quasi non-reactive fluoroacetamide 10b" is not accurate. In the GSH assay, compound 10b is surprisingly more reactive than phenylacrylamide (3a), which is known to possess significant reactivity (see e.g., doi 10.1021/jm501412a).

- Page 5, KGDYHFPIC peptide: Notably, Cys and Lys are terminal in this peptide which may impact their reactivity. Could the authors explain why this peptide was chosen and mention the possible impact of the terminal positioning?

- "data suggest O-S exchange as an attractive electrophile option that increases the reactivity," should have more nuanced phrasing given the limited and heterogeneous data.

- Page 5: "To understand and explain the observed reactivity differences of haloacetamides," the authors may mention that the nucleophilic attack presumably proceeds via SN2 and does not involve an attack on the (thio)carbonyl carbon.

- Figure 1: Given the low number of data points, assignment of individual data points to compounds should be made to increase comprehensibility.

- Figure 2 caption: please correct "Inhibiton by fargments"

- Page 6: "To evaluate their kinase targeting potential we have chosen six, covalently druggable, kinases representing each major kinase branch," the authors may comment on the positioning of the cysteines in the kinases and how this impacts their accessibility.

- Page 6: "suggesting that the O-S exchange can be further supported by the electron-withdrawing character of the substituents," should be rephrased as this is not surprising and is part of the rationale for including the CF3 compound.

- Page 7: Cys 909 labeling in JAK3: Were other labeling sites also observed?

- Page 8: "covalent labeling of BTK with efficacy proportional to the reactivity of the warheads," should note that potency is not correlated with MS labeling efficiency.

- Page 9: The statement "18 labeled a complementary subset of proteins as shown in the Venn diagram" would benefit from some discussion to rationalize this finding.

- Figure 3 has a distorted scaling.

- Page 13: For the kinase assays, could the authors please specify whether these assays were conducted by a CRO or by the authors themselves? The same should be done for the BTK radioligand assay.

- Color-coding in figure S3 is unclear (e.g., left column 8% inhibition red; 13% inhibition green).

- Error bars are needed for kinase assay curves in the supplementary information.

Reviewer #3

(Remarks to the Author)
This manuscript written by Petri et al. describes the O-S exchange strategy as late-stage modifications to offer halothioacetamide electrophiles that can be applied to the targeted covalent inhibitor (TCI) and antibody-drug conjugate (ADC) design. Sulfur exchange of oxygen generally increases the reactivity of warheads and can broaden the scope of target proteins (and cysteines). Four different aspects were explored for this new type of electrophiles: 1) kinase mapping with fragments, 2) covalent BTK inhibition, 3) antibody conjugation, and 4) chemoproteomics. Overall, the experiments were well-designed and executed. To provide clearer insight into thioamide warheads in comparison to amide warheads and strengthen the study, some additional experiments (see below specific comments) can be performed.

Specific comments:
1) In Figure 1, labeling individual probes for each data point would be more helpful than the group analysis of amide probes vs. thioamide probes.
2) What do authors think about why 9b shows decreased inhibition of JAK3 compared to 9a while 8b shows increased inhibition than 8a? Similarly, why is 11b showing increased inhibition of JAK3 and RSK2 compared to more reactive 9b?
3) Kinase mapping data indicate that the O-S exchange modulates not only the overall reactivity but also the target profile which needs to be mentioned and discussed more clearly.
4) The late-stage modification of TCIs using O-S exchange is exciting. But, as the intrinsic reactivity of halothioacetamides is higher than corresponding haloacetamides or acrylamides, it’s also possible that off-target reactivity is increased. Thus, it is important to assess and compare the global protein reactivity of TCIs containing thioamide. Authors can perform chemoproteomics analysis with alkyne-functionalized compounds or competitive chemoproteomics using a broad-spectrum cysteine reactive probe.
5) For antibody conjugation, FARs are similar between haloacetamides and halothioacetamides. Although this study

provides additional electrophiles that can be applied to ADCs, the added value of using thio-probe is not clearly demonstrated. Kinetic measurement can provide some more insight into the applicability of these warheads.
6) Does the IAA alkyne probe used in chemoproteomics contain the same scaffold as other probes?
7) It's surprising that fluorothioacetamide probe 20 labels more proteins (2708) than chloroacetamide probe 17 (1453). How do authors interpret this data?
8) Comparative analysis (Venn diagram) of proteins labeled by 17, 18, and 20 (in addition to IAA and 18) would help understand the target scope. Similarly, in addition to the number of proteins identified, the number of cysteines modified will be important to note.
9) Further validation of selected proteins that were uniquely modified by probe 18 (confirmation of covalent conjugation and identification of the binding site) would strengthen the authors' claim that thioprobes label a distinc subset of the proteome compared to the conventional iodoacetamide probe.

Reviewer #4

(Remarks to the Author)
Keseru and London et al. developed a relatively new cysteine-targeting warheads by transforming traditional carboxamide based warheads to soft electrophilic thioamides. The transformed electrophiles (including chlorothioacetamides and fluorothioacetamides) demonstrated enhanced reactivity and specificity for cysteine labeling while preserving the original warhead geometry. The authors further demonstrated these warheads can be used to develop BTK inhibitors and covalent modify antibody. In addition, the chlorothioacetamides compound (18) was able to covalently bind with more than 4000 proteins. This work can enrich the toolbox of cysteine-targeting warheads, offering new electrophiles for development of TCIs and chemical biology tools. However, the characterization of this kind of warhead is not thorough, and the overall advantages of this kind of warhead are not so significant. So it is recommended to include more results, current version was not good enough to be published in Nature Communications, but it can be given an opportunity to revise it. I will make the final decision based on the revised version.

Major Concerns:
1, The authors claimed the O-S exchange strategy enables precise late-stage modulation of covalent reactivity, which was proved only in Ibrutinib. The authors should include one more case with different structures, to demonstrate its feasibility on more structurally diversity compounds
2，The authors need to test covalent reaction kinetics of 12/13/15 with BTK, which can demonstrate and compare the covalent binding efficiency of inhibitors containing different warheads with proteins.
3, The authors should explain properly why they chose "N-benzyl-2-chloroethanethioamide"-based compounds in Fig.3/4 instead of "2-chloro-N-phenylacetamide" what they used in the beginning, these two versions may demonstrate different reactivities.
4, The results in Figure 3c are inconsistent with those described in S6. Please explain or describe them more clearly.
5, The fluorothioacetamides-containing compound 15 demonstrated significantly faster covalent binding rate with BTK comparing with chlorothioacetamides-containing compound 13, however, chlorothioacetamides-containing compound 18 displayed stronger reactivity with cysteine in comparing with fluorothioacetamides-containing compound 20
6, The authors claimed O-S exchange to this warheads improved its specificity for cysteine, so it's strongly suggested that the authors should determine proteomewide residue selectivity of the warheads they developed using residue-specific proteomics.
7, As warheads used for development of TCIs, the cellular potencies of chlorothioacetamides and fluorothioacetamides were also need to be evaluated, should include a piece of data to demonstrate the cellular potency for either chlorothioacetamides or fluorothioacetamides, such as evaluating the cellular potency of compound 13 or 15. Especially compound 18 or 20 show poor labeling efficiency in S6
Minor Issues:
4, , Line 481 in maintext "followed by 45 incubation at 25℃"
1, Didn't find Supplementary Table S7 which was mentioned line 224 in the maintext.
2, The authors claimed that "The labelling efficiency of the fluoro (20) and chloro (18) thioacetamide probes was comparable to that of the chloro (17) and iodo acetamide (IAA) probes, respectively" in the line 221-222 according to the results in S6. This statement was not true according to the results in S6. And the results quality in S6 was not good enough, especially for compound 19.
3, The starting molecular weight values in Figure S5 should be same, for easy reading.
4, , Line 481 in maintext "followed by 45 incubation at 25 ℃"

Version 1:

Reviewer comments:

Reviewer #1

(Remarks to the Author)
The authors have improved the presentation of their manuscript. However, I still cannot recommend the manuscript for publication in its current form.
Major Comments

a) I continue to believe that the manuscript does not fit well within the scope of the journal. My general perception is that this

manuscript is better suited for a journal specialized in Medicinal Chemistry.
b) The computational section still contains several methodological and technical deficiencies, as well as missing information in both the Methods and Discussion sections. For example, In the Methods section, the authors state: “QM/MM calculations Structure preparations for thermodynamic integration The structure for BTK–Ibrutinib complex (PDB: 5P9J85) was used as a starting structure to build the complexes of compound 12 (O) and 14 (S). The preparation was performed with Schrödinger’s Maestro module [Schrödinger Release 2024-3: Maestro, Schrödinger, LLC, New York, NY, 2024.] and included the deletion of the covalent bond between Cys481 and the ligand, the modification of the ligand warhead, and the protonation of the sulfur of Cys481. Restrained minimization was performed to alleviate steric clashes.” However, there is no information on the system preparation parameters, such as the treatment of titratable residues, protonation states, or pKa calculations of amino acids. Why was Cys481 protonated? Is there any experimental or theoretical evidence supporting this choice?
The authors must clarify the rationale for this decision and provide sufficient details for reproducibility.
c) The authors describe their TI setup as follows: “Thermodynamic integration was carried out by the AMBERtools24 software package (Amber 2024, University of California, San Francisco, CA, USA)... [etc.]” However, the methodology is inadequately described. The authors should include a thermodynamic cycle to illustrate the perturbations performed. Moreover, a simulation time of only 20 ps per window is insufficient to ensure proper sampling. The absence of replicate simulations further undermines the statistical reliability of the reported free energies.
d) In methods section, the manuscript states: “The QM region consisted of the active site cysteine residue cut between C and C and also the warheads of the ligands... The difference between the S–C and C–Cl bond distances was used as a reaction coordinate.” The reaction coordinate should be clearly illustrated in a figure. Without this, the description is difficult to follow. The authors must also provide details regarding the number of windows, sampling time per window, and QM/MM partitioning scheme to ensure reproducibility.
e)The computational results are poorly described and insufficiently presented. The authors should: Provide a table summarizing the relative free energies obtained from the TI calculations. Include the PMF profiles and tabulated activation free energies. Supply the starting structures used in the simulations as Supporting Information (SI) to allow reproducibility.

Reviewer #2

(Remarks to the Author)
The authors have revised their manuscript and most of this reviewer’s points have been addressed. However, some issues still require further refinement or clarification, and a few new concerns have arisen following the revisions. In addition, one important point regarding the analytical data was overlooked in the initial assessment, necessitating another round of major revision. The authors should address the following aspects:

Major:

New: Several compounds exhibit significant contamination in the NMR data and/or poorly resolved spectra. The following NMR spectra need improvement:

- ^{1}H NMR: Compounds 5a, 6b, 9, 14, 15, 24
- ^{13}C NMR: Compounds 5b, 6b, 13, 14, 15, 18, 21

Furthermore, the compounds should be depicted on the respective spectra to facilitate verification of chemical shifts.

Initial request: The initial reactivity study does not include any alkyl amine-derived thioamide electrophiles, so it is unclear how the aniline-derived electrophiles from the first part of the study compare to the piperidine-based ones used in the second part. These two compound classes should be ranked against each other. I strongly suggest synthesizing benzylamine and piperidine-derived sets of halothioamide/thioacrylamide fragments and testing these in the GSH assay for comparison. It would also be important to show if the decreased reactivity commonly observed for N-alkyl acrylamide versus N-aryl acrylamide transfers to thioacrylamide.

Author response: Accepting the comment of the referee we synthesized the following compounds: N-benzyl-chloroacteamide, N-benzyl-chlorothioacetamide, N-benzyl-fluorothioacetamide, N-piperidinyl chloroacetamide, N-piperidinyl chlorothioacetamide, N-piperidinyl fluorothioacetamide. These compounds were evaluated in the kinetic GSH assay as well as in the oligopeptide-based selectivity assay, and the results are now discussed in the main text. In brief, the new data do not alter the overall conclusions but provide significant confirmation of the O/S exchange effect originally reported in this study. As our attempts to prepare thioacrylamides were unsuccessful in most cases, we finally decided to remove all thioacrylic and acrylic probes from the study and instead focus exclusively on the haloacetamide and halothioacetamide series. The revised library now comprises 16 probes across four scaffolds (phenyl, bistrifluorophenyl, benzyl, and piperidyl), representing a robust and well-designed covalent fragment set.

Reviewer Response: I appreciate the synthesis and characterization of the new. However, the discussion should mention thioacrylamides, their synthesis attempts and the observed high reactivity and limited accessibility that ultimately led to their exclusion. Including this information would be highly valuable for other researchers, as it could prevent unnecessary repetition in future studies.

Initial request: The degree of intact protein labeling and stoichiometries need to be quantified and discussed for all tested probes and proteins (see comment above).

Author response: The extent of protein labeling has been quantified, and the results are now provided in the supplementary material. Since the MS measurement conditions were not standardized, we do not discuss these data in detail in the main text, as no robust conclusions can be drawn from the heterogeneous results obtained (see Supplementary Information)

Reviewer Response: Thank you for addressing this point. In addition, please include the labeling stoichiometries. Some discussion regarding compounds' degree of labeling would also be warranted.

Initial request: Considering the potential application of these thioamide-derived warheads in TCIs, and the known susceptibility of sulfur to oxidation, some investigation of metabolism, at least in vitro, would be a beneficial addition to the data presented.

Author response: We thank the reviewer for this valuable suggestion. To address it, we performed in vitro human liver microsome metabolism experiments and assessed the remaining compounds by HPLC measurements. In brief, both the chloroacetamide and chlorothioacetamide derivatives displayed a similar range of metabolic stability, with 1–4% of the compound remaining after 30 minutes. In contrast, the moderately reactive but proven covalent fluorothioacetamide derivative showed increased stability, with 10% remaining after 30 minutes.

Reviewer response: It seems this data has not been added. The authors should include the in vitro metabolism data and add a brief discussion of the observed properties.

Minor:

Initial request: How did the authors unambiguously distinguish between the respective labeled amino acids in the peptide assay? Please specify in the body of the manuscript and provide data. The same applies to protein labeling.

Author Response: The identification of labeled amino acids in both peptide and protein levels was performed using standard LC–MS/MS analysis. In the case of protein labeling, proteins were digested with trypsin (and, where appropriate, chymotrypsin) to generate a series of peptides with defined cleavage sites, including a/b/c and x/y/z ion series. Labeled peptides were detected as precursor (mother) ions, and their fragmentation patterns were analyzed to determine the exact position of the modification. Differences in the m/z ratios of fragment ions between labeled and unlabeled peptides allowed unambiguous identification of the labeling site.
For nonapeptide labeling, the approach is technically equivalent, but no enzymatic digestion was required because the oligopeptide is already compatible with downstream MS analysis. The labeled peptide was directly detected, and the position of the modification was confirmed by the MS/MS fragmentation pattern.
To illustrate this, we prepared a representative figure (below), showing the labeled peptide with the fragmentation pattern annotated to highlight the a, b and y ions that differ from the unlabeled series at the modification site. Additionally, we have added the MS/MS spectra of all covalent probes reacted with the nonapeptide surrogate in the supplementary material. These spectra demonstrate the unambiguous assignment of the labeling sites. This approach is consistent with standard practices in peptide mapping and chemical proteomics studies.

Reviewer Response: Thank you for providing the new figure. It clearly addresses and clarifies this point. However, it appears that it has not been included in the manuscript. Please ensure that it is added, at least as part of the Supporting Information (SI).

Initial request: If the piperidine-derived thioacrylamide proofs to be significantly less reactive than the above aniline derivatives, this electrophile should be included in the TCI part of the study.

Author response: We agree with the reviewer that the piperidine-derived thioacrylamide would represent an interesting electrophile to investigate. Therefore, we synthesized and tested a phenyl-thioacrylamide fragment (6a). This compound was hyper-reactive and labelled GSH promptly. Any further attempts to prepare thioacrylamides were unsuccessful due to the extremely high reactivity and the limited stability of these compounds. For this reason, as stated above, we finally decided to remove all thioacrylic and acrylic probes from the study and instead focus exclusively on the haloacetamide and halothioacetamide series. The revised library now comprises 16 probes across four scaffolds (phenyl, bistrifluorophenyl, benzyl, and piperidyl), representing a robust and well-designed covalent fragment set. In the revised manuscript, we discuss the presented thioamide-based warheads as a novel option for bioconjugation strategies, including TCI development, ADC design, and target identification. We believe that the chloro- and fluorothioacetamides introduced herein represent valuable new tools for the medicinal chemistry and chemical biology communities, opening avenues for next-generation covalent drug discovery.

Reviewer response: see major comment on discussion of thioacrylamides above

Initial request: TCI part, kinase inhibition profiling: For rigorous characterization of covalent inhibitors, especially if different warheads/electrophiles are to be compared, the contributions of non-covalent and covalent binding need to be deconvoluted (i.e., KI and kinact). Kinetic assays are now readily available for BTK, and such data must be complemented accordingly. The fact that probe 14 does not seem to modify the enzyme at all but still has a relatively potent IC50 of 45 nM should also be discussed. It seems that the covalent mode of action does not contribute much to the observed activity, in line with the slow labeling kinetics shown in the supplementary information. Interestingly, probe 13 shows very slow labeling kinetics in MS but higher potency than 12 and Ibrutinib, which have much faster labeling kinetics. This suggests that the observed boost in activity is due to non-covalent interactions. This further supports the critical need for kinact and KI determination for these derivatives and a more elaborate discussion of the results.

Author response: We thank the reviewer for this insightful comment. To address it, we deconvoluted the contributions of non-covalent and covalent binding to BTK by determining KI and kinact values in kinetic assays for the ibrutinib derivatives (compounds 11–15). The results confirmed the significant role of non-covalent interactions, in line with the trends anticipated from our preliminary reactivity experiments. These findings are now discussed in detail in the main text, and the corresponding data are reported in the Supplementary Material.

Reviewer response: The addition of this data is appreciated. However, it is recommended providing kinact values in s-1 (including for the kinact/KI ratio), as this format is more commonly used in the literature and facilitates comparison across studies. Additionally, please follow the style guidelines outlined in https://doi.org/10.1021/acs.jmedchem.4c01721 for kinact and KI in both the text and figures.

Initial request: title/manuscript: "Thioamide warhead" should be rephrased to "thioamide-derived warhead" or similar as thioamide itself is not the warhead functional group.

Author response: The title has been changed to “Thioamide-derived warheads with enhanced cysteine reactivity and specificity for covalent protein labelling”

Reviewer response: The changes are ok, but they should be implemented consistently throughout the manuscript. Since acrylamides have been removed from the scope, a more restricted and precise title is recommended, such as “α-Halo thioamide…” to accurately reflect the content.
Initial request: Page 2: "were mostly avoided from drug discovery pipelines due to potential idiosyncratic toxicity issues" could also mention that lack of selectivity was often a factor associated with covalent drug modalities.

Author response: The suggested text was added.

Reviewer response: Idiosyncratic reactions are not necessarily a consequence of unselectivity, and conversely, unselectivity can lead to issues other than idiosyncrasy. I suggest using more nuanced language here, especially since this is the opening sentence of the paper.

Initial request: In the introduction, the authors may discuss the reaction types more clearly to provide context. Notably, the C=S bond is not directly involved in the displacement of alpha leaving groups. Although the reasoning sounds logical, the impact of the C=S bond on the softness of the beta-position in thioacrylamide electrophiles should be discussed with suitable citations or backed by own data.

Author response: We have clarified in the text that the C=S bond is not directly involved in the displacement of α-leaving groups, but it does alter the electronic environment of the adjacent β-position. To support this, we performed DFT calculations (M062X/6-311++G(d,p)) and analyzed the NBO- charge distribution localized to the adjacent carbon atom in β-position. These results consistently showed a positive shift of charge on the carbon adjacent to the thiocarbonyl group compared to the corresponding carbonyl analogue, indicating its increased electrophilic character. This supports our conclusion that thioamides act as more potent electrophiles due to these intrinsic electronic effects.

Reviewer response: Please make sure the data is mentioned in the manuscript

Initial request: Table 1 and discussion: Conditions (pH, solvent, temperature, concentrations) should be mentioned. Notably, it seems phenylacrylamide, a common standard, behaves relatively unreactively here (GSH t1/2 is often cited in the range of ~1-3h). It should also be mentioned if the depicted values are corrected for degradation or not.

Author response: We thank the reviewer for pointing this out. We have now added the experimental conditions (pH, solvent, temperature, and concentrations) to the caption of Table 1. Regarding the reactivity of phenylacrylamide, we note that its relatively less reactivity in our experiments likely reflects the specific buffer system, lower incubation temperature (25°C vs. 37°C) and concentration settings we used, which can differ from conditions in previously cited studies reporting shorter half-lives. Furthermore, we systematically applied stability correction for background degradation of the electrophiles, which can substantially affect the apparent reactivity. These corrections provide a more reliable assessment of intrinsic thiol reactivity. We have clarified this in the revised text.

Reviewer response: The reference to aqueous stability correction may be misleading in the context of phenylacrylamides, as Table S2 indicates that the compound shows no significant degradation in buffer (t1/2 > 72 h). Rephrasing suggested.

Initial request: Page 3: "Our results confirmed that O-S exchange increased the thiol-reactivity," is an overgeneralization. While the GSH data support this statement for AA/TAA, differences are fairly minor for the CA/CTA and FA/FTA matched pairs; CTA with R=H has essentially the same stability as its CA analog. Please refine this statement.

Author response: We refined the wording as follows: “Our results confirmed that O–S exchange positively contributed to the thiol reactivity of the covalent probes investigated herein.” By extending the covalent fragment-level investigations with eight additional probes, we obtained solid support for the conclusion that the increase in thiol reactivity upon O–S exchange generally holds true. However, we acknowledge that this is not an exclusive rule, but rather a useful option for structure optimization.

Reviewer response: It seems the wording has been changed again after writing the rebuttal. This is should be avoided.

Initial request: Page 5, KGDYHFPIC peptide: Notably, Cys and Lys are terminal in this peptide which may impact their reactivity. Could the authors explain why this peptide was chosen and mention the possible impact of the terminal positioning?

Author response: Although we agree that the terminal position of Cys and Lys might impact their reactivity, we used the same peptide in multiple papers (https://doi.org/10.1016/j.ejmech.2018.10.010; https://doi.org/10.1016/j.ejmech.2020.112836; https://doi.org/10.1002/cbic.202000700; https://doi.org/10.1016/j.ejmech.2022.114163; https://doi.org/10.3390/molecules28073042) and it was generally found to be useful for the first assessment of labelling specificity.

Reviewer response: The authors argumentation is ok but it is recommended to mention the potential impact of terminal localization briefly.

Initial request: Page 6: "To evaluate their kinase targeting potential we have chosen six, covalently druggable, kinases representing each major kinase branch," the authors may comment on the positioning of the cysteines in the kinases and how this impacts their accessibility.

Author response: The target kinases were selected as therapeutically significant and already covalently targeted members of the protein kinase family. Taking into account the availability of covalently targetable cysteine residues (https://doi.org/10.7717/peerj.126), and based on the availability of LCMS-measurements of those targets, finally 3 kinases were selected: JAK3 and BTK from the TK (tyrosine kinase) branch, and MAP2K6 from the STE (yeast sterile 7-,11- and 20-homologue kinases) branch. Positioning of the targeted cysteines and characterization of their reactivity and accessibility based on Cy-preds server predictions (https://doi.org/10.1002/prot.24978; http://bioinformatics.akdeniz.edu.tr/cypreds/start.py) are summerized below. Also, we added some further information to the main text to discuss the covalent tractability of the cysteines in those 3 included kinases. Interestingly, those investigated kinases characterized by similar accessibility, yet significantly different reactivity on their targeted cysteine residues.

Reviewer response: Cys pKa prediction is prone to significant error and this limitation should be briefly acknowledged in the manuscript. Additionally, it would be helpful to mention in the text that the cysteine residues for BTK and JAK3 are located in the same position within the front pocket, whereas MKK6 has its cysteine in the DFG region.

Additional minor points:

Page 2: “over a hundred functional groups available for covalent labeling.” I recommend avoiding explicit numbers here, as these can vary depending on what is considered a distinct functional group. Instead, using a more general phrasing such as “many” or “a significant number of” is recommended.

Page 2: “thioamides are expected to be softer electrophiles compared to their carbonyl counterparts, implying higher cysteine specificity.” I am not convinced that this statement remains valid now that the Michael acceptor compounds have been excluded (see discussion regarding the involvement of the C=S bond). Rephrasing is recommended.

Page 3: “its electronic effect strongly influences the adjacent β-position” Since Michael-type electrophiles are now excluded, this consideration should only apply to the α-position. Please check.

Page 3: “electrophilicity, providing a straightforward explanation for the higher reactivity observed with thioacrylamide electrophiles.” Thioacrylamides are no longer included in the manuscript. However, as previously mentioned, the authors should add a brief discussion noting that these compounds were synthesized but excluded from further studies due to their excessive reactivity.

Page 5: “The methyl group on the adjacent β-carbon of the chloroacetamide sterically hinders the” The methyl group is actually attached at the α-position

Page 6: “covalently weakly reactive fluoroacetamide 13” Please delete “covalently”

Page 8: “a viable and advantageous late-stage warhead optimization strategy, enhancing potency without introducing additional off-target liabilities.” Softer phrasing is suggested, as the results presented here remain rather preliminary regarding their applicability to TCIs in a drug discovery context.

Page 10 “The labelling efficiency of the 21 chloro- and the 22 fluorothioacetamide probes was comparable to that of the iodoacetamide (23) probe” This would imply that these warheads are overly reactive when it comes to TCI applications. On the other hand, the authors make the point that their kinase inhibitors show drug-like proteome labeling profiles, which seems contradictory. Please rephrase the relevant parts of the manuscript to properly reflect the data and the applied assay conditions. Given the rather broad reactivity of the chemoproteomic probes, I also suggest generally down-tuning the language regarding selectivity claims and potential TCI applications, while placing greater emphasis on the application potential as chemoproteomic probes.

Page 11: “displaced the tracer with an IC50 of 54.4μM” Please provide the incubation time as this process is time-dependent. Does the calculation of a Ki via Cheng-Prusoff (see page 17) make sense given the time-dependent nature of the process?

Page 15 “cut between C and C” seems to have an issue in the converted pdf. Please check.

Reviewer #3

(Remarks to the Author)
The authors have thoughtfully considered the review suggestions and provided substantial additional experimental data. They have addressed the previous concerns. The added data and revisions have greatly enhanced the scientific value and significance of the revised manuscript. Therefore, I would recommend that you accept the paper for publication in Nature Communications without further revision.

Reviewer #4

(Remarks to the Author)
The reviewer has included many additional experiments, and my concerns have been properly addressed in the revisions.

However, the current version of the manuscript contains compound numerous errors in descriptions and data presentation. Without correction of these errors, the paper cannot be published.

1. Compound numbers are highly frequently incorrect, e.g., in lines 117, 118, 223, 226, and 243.

2. In the response letter to reviewers, the IC_{50} values for compounds 13 and 14 in response to Q7 are incorrect.

3. The legend in Figure S2 is confusing. Figure S2a should include more detailed information, specifying which peak corresponds to the covalent binding between the protein and the compound.

Besides the errors mentioned above, I suggested they can also revise the issues in below
1,The statement in Line 145, "while its selectivity over BTK was maintained," is not accurate. The selectivity fold over BTK for compound 7 is 5.6, while for compound 9 it is 2.65, indicating a significant decrease in selectivity.

2,In Line 188, it is unclear why site mapping identification was not performed for compound 14, which contains the chlorothioacetamide moiety they developed.

3,It is recommended to conduct kinome screening for BTK inhibitors 12 and 14, allowing a comparison of inhibition selectivity among compounds 11, 12, and 14.

4, In Line 127, the claim that "thioamide does not increase cellular promiscuity" is only based on a comparison with the corresponding acetamide. However, when compared to the parent compound ibrutinib (which contains an acrylamide), thioamide significantly increases cellular promiscuity.

Version 2:

Reviewer comments:

Reviewer #1

(Remarks to the Author)

The authors have addressed all of my concerns. I recommend the manuscript for publication.

Reviewer #2

(Remarks to the Author)
The authors have revised their manuscript and have appropriately addressed the majority of this reviewer's comments. While the newly added NMR data are acceptable for publication, baseline correction and phasing could be improved, and signal intensities and purity are not ideal in some cases. A few minor points remain to be addressed:

- The authors have included a useful discussion of quantification based on intact MS data and protein-compound stoichiometries in their incubation experiments. However, it would be valuable to also comment on adduct stoichiometries, specifically the extent to which 1:1 adducts versus higher order adducts are observed.
- The removal of phenylacrylamide from both the experimental set and the discussion is not appropriate, even though thioacrylamides were excluded. Phenylacrylamide remains an important benchmark compound that enables comparability with other studies. The authors should re-include this compound in their evaluation, explicitly noting that it is used as a benchmark and highlighting that it appears significantly less reactive in this study compared with many previous reports. This information is important for readers to properly rank the reactivity range of the compounds examined here
- With regard to the added discussion on chemoproteomic reactivities and assay conditions, the current wording remains somewhat unconvincing. If the compounds are indeed less reactive than IAA, one would generally expect reduced levels of labeling. Additional discussion addressing potential reasons for the apparent differences in promiscuity between the chemoproteomic probes and other compounds in the set would be helpful.

Reviewer #4

(Remarks to the Author)
My comments and questions have been adequately addressed. I therefore recommend this article for acceptance.

Open Access This Peer Review File is licensed under a Creative Commons Attribution 4.0 International License, which permits use, sharing, adaptation, distribution and reproduction in any medium or format, as long as you give appropriate credit to the original author(s) and the source, provide a link to the Creative Commons license, and indicate if changes were made.
In cases where reviewers are anonymous, credit should be given to 'Anonymous Referee' and the source.
The images or other third party material in this Peer Review File are included in the article's Creative Commons license, unless indicated otherwise in a credit line to the material. If material is not included in the article's Creative Commons license and your intended use is not permitted by statutory regulation or exceeds the permitted use, you will need to obtain permission directly from the copyright holder.
To view a copy of this license, visit https://creativecommons.org/licenses/by/4.0/

Response to Reviewers

Please also see the Nature Communications formatting instructions, which you may find useful while preparing your revised manuscript.

We have fully formatted the revised manuscript according to the Nature Communications formatting guidelines.

POLICIES AND FORMS REQUIRED FOR RESUBMISSION

* Please complete or update the following checklist(s) to verify compliance with our research ethics and data reporting standards. Address all points on the checklist, revising your manuscript in response to the points if needed. The form(s) must be downloaded and completed in Adobe Reader rather than opened in a web browser. Each form must be uploaded as a Related Manuscript file at the time of resubmission.

Editorial policy checklist:
https://www.nature.com/documents/nr-editorial-policy-checklist.pdf

Reporting summary:
https://www.nature.com/documents/nr-reporting-summary.pdf

Your work characterises chemical or biomolecular materials. Please see the link below for reporting requirements. There is no form to upload but you may need to revise your manuscript to comply with this policy.
https://www.nature.com/ncomms/submit/chemical-characterisation

We have completed all the checklist (and upload if necessary) to verify compliance with the research ethics and data reporting standards of Nature Communications.

DATA AND CODE AVAILABILITY

* All Nature Communications manuscripts must include a “Data Availability” section after the Methods section but before the References. If any of the data can only be shared on request or are subject to restrictions, please specify the reasons and explain how, when, and by whom the data can be accessed. For more information on this policy and a list of examples, see:
https://www.nature.com/documents/nr-data-availability-statements-data-citations.pdf

We added a ‘Data Availability’ section to the manuscript and deposited all raw data, with relevant datasets provided in the supplementary material and others (proteomic datasets) deposited in PRIDE repository, as described.

* As Nature Portfolio policies strongly encourage you to share your research data in a public repository (e.g. spreadsheets, text, images), we are partnering with the figshare repository so that you can use the figshare integration via the ‘Research Data Deposition’ tab when submitting your revised manuscript.

* Proteomic datasets must be deposited in a publicly accessible database, and accession codes and associated hyperlinks must be provided in the “Data Availability” section.

* To maximise the reproducibility of research data, we ask that you provide a Source Data file containing the raw data underlying the following types of display items:
- Any reported means/averages in box plots, bar charts, and tables
- Dot plots/scatter plots, especially when there are overlapping points
- Line graphs
- Uncropped and unprocessed scans of all blots and gels including all quantified replicates. The edge of membranes, molecular weight ladders and loading controls should be presented on all blots. Where membranes have been cut, please ensure that at least one marker above and below is present. For an example of presentation of full scan blots, see the Source Data file of https://www.nature.com/articles/s41467-020-16984-1#Sec35 and for more information, please refer to https://www.nature.com/nature-research/editorial-policies/image-integrity.

The data should be provided in a single Excel file with data for each figure/table in a separate sheet, or in multiple labelled files within a zipped folder. Name this file or folder ‘Source Data’, and include a brief description in your cover letter. The “Data Availability” section should also include the statement “Source Data are provided with this paper.”

We prepared and uploaded the required ‘Source Data’ file as requested and updated the ‘Data Availability’ section accordingly to reflect these changes.

Please replace your bar graphs with plots that feature information about the distribution of the underlying data. All data points should be shown for plots with a sample size less than 10. For larger sample sizes, please consider box-and-whisker or violin plots as alternatives. Measures of centrality, dispersion and/or error bars should be plotted and described in the figure legend.

We carefully reviewed all figures and revised the graph types where necessary in accordance with the guidelines.

ORCID*
Nature Communications is committed to improving transparency in authorship. As part of our efforts in this direction, we are now requesting that all authors identified as ‘corresponding author’ create and link their Open Researcher and Contributor Identifier (ORCID) with their account on the Manuscript Tracking System prior to acceptance. For more information please visit http://www.springernature.com/orcid

All co-authors have been informed and reminded to add their ORCID identifiers to their accounts, noting that this must be completed prior to acceptance.

AUTHOR CHANGES ON REVISION
If there are any changes to the author list in the revised manuscript, please use this

approval form www.nature.com/documents/nr-author-list-change-form.pdf, arranging for all authors on your paper to sign the statement confirming that they agree to the author list being changed, and add this document to your resubmission.

There were no changes made to the author list.

Reviewer #1 (Remarks to the Author):

In this manuscript, the authors have synthesized warheads and explored their application in labeling Bruton's tyrosine kinase (BTK). My general perception is that this manuscript is better suited for a journal specialized in Medicinal Chemistry, as it involves synthesis, computational methods, and biochemical evaluation of the compounds. For this reason, I cannot recommend its publication in Nature Communications.

Major Points

a) The manuscript does not fit within the scope of the journal.
We believe that our chemical biology work falls within the scope of this multidisciplinary journal, which publishes research across the biological, health, physical, chemical, Earth, social, mathematical, applied, and engineering sciences.

b) There is no urgency to publish it as a communication.
Our manuscript was submitted as a regular research article.

c) The computational section describes the reaction in solution; however, kinetics should take into consideration the enzymatic environment.
We agree that the reactivity of electrophiles is influenced by the protein environment, as it affects the nucleophilicity of the targeted residue. However, the computational work presented in this manuscript was designed to predict warhead reactivity against amino acid surrogates, which represents an important parameter in the development of compounds with a covalent mechanism of action, as binding kinetics play a crucial role in the design of covalent inhibitors (https://doi.org/10.1021/acs.jmedchem.4c02863, https://doi.org/10.1101/2025.06.19.660637). Importantly, efficacy of TCIs is not governed solely by equilibrium affinity but by a two-step kinetic mechanism, noncovalent binding (characterized by K_I) is followed by the formation of the covalent bond (characterized by k_{inact}). Factors such as the polarity and geometry of the binding pocket, steric constraints, nucleophilicity and accessibility of the targeted residue, as well as its conformational flexibility, directly modulate k_{inact} and K_I, thereby determining both selectivity and efficacy. Thus, rational optimization of covalent drugs requires a kinetic perspective, ensuring that the interplay of noncovalent recognition and chemical reactivity enables productive, selective, and physiologically relevant covalent modulation of the target.

d) The design of the compounds is not based on computational methods. The authors wrote: "Since the steric and electronic effects of the substituents might change the non-covalent recognition of the scaffold, we wondered whether the isosteric O-S exchange of carboxamides is useful for tailoring the reactivity of the warhead." The computational section should be improved to better describe the enzymatic environment and the design of warhead.
Accepting the comment of the referee, we utilized our previously published protocol (https://pubs.acs.org/doi/full/10.1021/acs.jcim.0c00834) to evaluate the

thermodynamics of the labeling reaction, comparing the carboxamide **12** and thiocarboxamide **13** warheads in the context of BTK inhibitors. This methodology, based on thermodynamic integration coupled with umbrella sampling, calculates the free energy of activation for the labeling reaction It generally provides valuable insights for the design of covalent probes across a variety of protein targets (https://onlinelibrary.wiley.com/doi/10.1002/9783527840748.ch23; https://onlinelibrary.wiley.com/doi/10.1002/qua.26768; https://www.sciencedirect.com/science/article/pii/S2001037021003391).

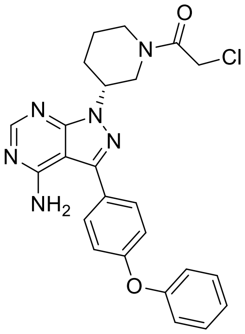

12

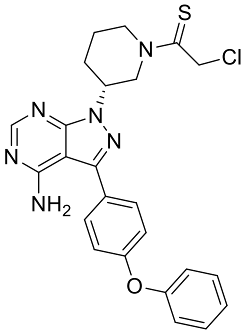

13

The effect of replacement of the chloroacetamide by chlorotioacetamid warhead was investigated by computing the binding free-energy difference of the noncovalent complexes on the one hand, and the reaction barrier free-energies, on the other hand. The binding free-energy difference ($\Delta\Delta G_b$) was obtained by thermodynamics integration using an O-to-S perturbation, while the reaction barriers ($G^{\#}$) were obtained from QM/MM free energy simulations. $\Delta\Delta G_b$ can be converted to the ratio of dissociation constants of the non-covalent complexes and $G^{\#}$ can be converted to k_{inact}. In this way, they can be compared with experimental observations.

	experimental	calculated
K_I[S-Cl]/ K_I[O-Cl]/	10.1	3.8
k_{inact}[S-Cl]/k_{inact}[O-Cl]	28	111
k_{inact} /K_I[S-Cl] / k_{inact} /K_I[O-Cl]	2.7	29

Experimental and calculated parameters show the same trend for the O-S replacement in the warhead. The non-covalent complex formation is slightly disfavored for the chlorothioacetamide warhead (**14**) compared to chloroacetamide (**12**) warhead as shown by the higher K_I value of the former. This is overcompensated by the higher reactivity of the chlorothioacetamide warhead exhibiting a higher k_{inact} value. The covalent inhibitory potential is conveniently characterized by the k_{inact} /K_I ration that is higher for compound with chlorothioacetamide warhead showing its higher inhibitory potential.

Reviewer #2 (Remarks to the Author):

Covalent strategies have become increasingly important in drug discovery and chemical biology. There is a need for expanding the toolbox of covalent warheads. This study seeks to expand the scope of haloacetamide and acrylamide warheads by transitioning to analogous thioamide compounds, which modulates reactivity while having only a minor impact on reaction geometries. The approach has been validated using fragments in reactivity assays, DFT calculations, a kinase fragment screen, and by applying the electrophiles to a targeted covalent inhibitor (e.g., Ibrutinib), antibody-drug conjugation and in a chemoproteomic profiling. Overall, this is a simple yet very good idea, yielding interesting results. However, the manuscript feels somewhat incomplete. The discussion often appears superficial and not very nuanced, and significant additional experiments are needed to meet the level of rigor expected in a high-impact journal such as Nature Communications.
Therefore, I strongly recommend publication of the manuscript in Nature Communications on the condition that the requested experimental data are supplemented in a major revision or resubmission of the manuscript.

We are grateful for the reviewer's supportive opinion. We carefully considered all comments and performed all the additional experiments requested. Thanks to these suggestions, we believe that the revised manuscript now includes all data necessary to evaluate the O/S exchange strategy in covalent ligand discovery programs.

The following points should be considered:

Major:

- How did the authors unambiguously distinguish between the respective labeled amino acids in the peptide assay? Please specify in the body of the manuscript and provide data. The same applies to protein labeling.
The identification of labeled amino acids in both peptide and protein levels was performed using standard LC–MS/MS analysis. In the case of protein labeling, proteins were digested with trypsin (and, where appropriate, chymotrypsin) to generate a series of peptides with defined cleavage sites, including a/b/c and x/y/z ion series. Labeled peptides were detected as precursor (mother) ions, and their fragmentation patterns were analyzed to determine the exact position of the modification. Differences in the m/z ratios of fragment ions between labeled and unlabeled peptides allowed unambiguous identification of the labeling site.
For nonapeptide labeling, the approach is technically equivalent, but no enzymatic digestion was required because the oligopeptide is already compatible with downstream MS analysis. The labeled peptide was directly detected, and the position of the modification was confirmed by the MS/MS fragmentation pattern.
To illustrate this, we prepared a representative figure (below), showing the labeled peptide with the fragmentation pattern annotated to highlight the a, b and y ions that differ from the unlabeled series at the modification site. Additionally, we have added the MS/MS spectra of all covalent probes reacted with the nonapeptide surrogate in the supplementary material. These spectra demonstrate the unambiguous

assignment of the labeling sites. This approach is consistent with standard practices in peptide mapping and chemical proteomics studies.

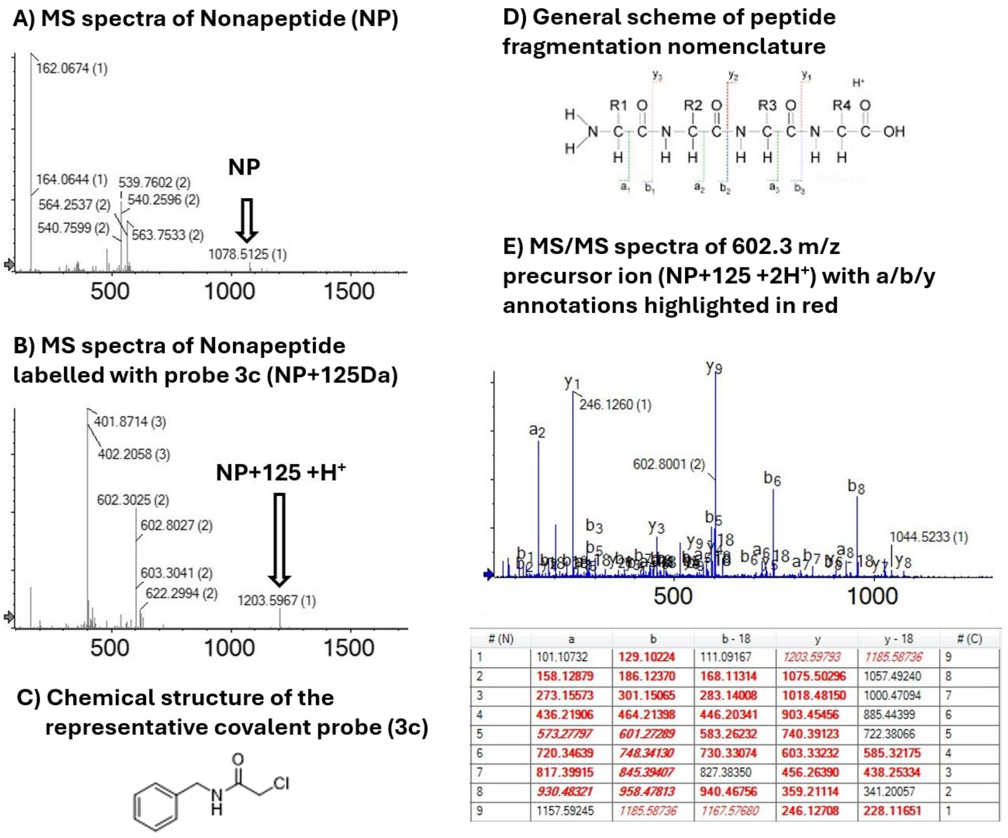

# (N)	a	b	b - 18	y	y - 18	# (C)
1	101.10732	129.10224	111.09167	1203.59793	1185.58736	9
2	158.12879	186.12370	168.11314	1075.50296	1057.49240	8
3	273.15573	301.15065	283.14008	1018.48150	1000.47094	7
4	436.21906	464.21398	446.20341	903.45456	885.44399	6
5	573.27797	601.27289	583.26232	740.39123	722.38066	5
6	720.34639	748.34130	730.33074	603.33232	585.32175	4
7	817.39915	845.39407	827.38350	456.26390	438.25334	3
8	930.48321	958.47813	940.46756	359.21114	341.20057	2
9	1157.59245	1185.58736	1167.57680	246.12708	228.11651	1

- The kinase inhibition assays on page 6 require additional data for validation of the findings. Importantly, kinase inhibition tends to show a weak correlation with covalent modification determined by mass spectrometry as suggested for example by kinome screening data recently published (doi 10.1002/anie.202419736). Therefore, the kinase inhibition data must be complemented with intact protein MS data for all probes/proteins as a more rigorous measure of protein reactivity.

To confirm the covalent mechanism of action, we originally applied orthogonal mass spectrometry methods: (i) trypsin digestion followed by MS/MS mapping studies on JAK3, which revealed covalent labeling by all biochemically active probes (**3a**, **3b**, **5a**, **5b**, **6b**) at Cys909 near the ATP-binding site, and (ii) intact LC–MS measurements on BTK, which confirmed covalent labeling by the same probe set. These data were already included in the original submission. In response to the reviewer's comment, we undertook extensive additional intact MS experiments across the broader kinase panel.

For MAP2K6, we obtained a formulation (SinoBiological 10422-H20B) that was MS-compatible and successfully confirmed covalent labeling with all active probes (**3a**, **3b**, **5a**, **5b**, **6b**), in well agreement with the expectations based on biochemical results and the known intrinsic thiol-reactivity of the probes. These new data are now included in the manuscript. For MELK, RSK2 and ERK2, we tested multiple commercial formulations (SinoBiological M50-18B and TargetMol TMPH-01642 for MELK, SinoBiological R17-10G for RSK2, SinoBiological 10030-H09B and M28-14B

for ERK2) and applied a variety of buffer-exchange approaches (Merck Microcon 10 kDa MWCO, Sartorius Vivaspin 10 kDa, Thermo ZebaSpin 7 kDa microSEC), but due to stabilizing additives and/or reducing agents present in the storage buffers, intact MS signals could not be recovered despite repeated efforts. Thus, unfortunately intact MS data for these three kinases could not be generated.
We note, however, that MS labeling could be confirmed for three kinases (BTK, JAK3, MAP2K6), out of the six proteins investigated. Together with the biochemical assays, this provides strong and convergent evidence for the covalent mode of action across the panel. Nevertheless, accepting the comment of the referee we discuss only the kinases we were able to confirm covalent labelling by MS.

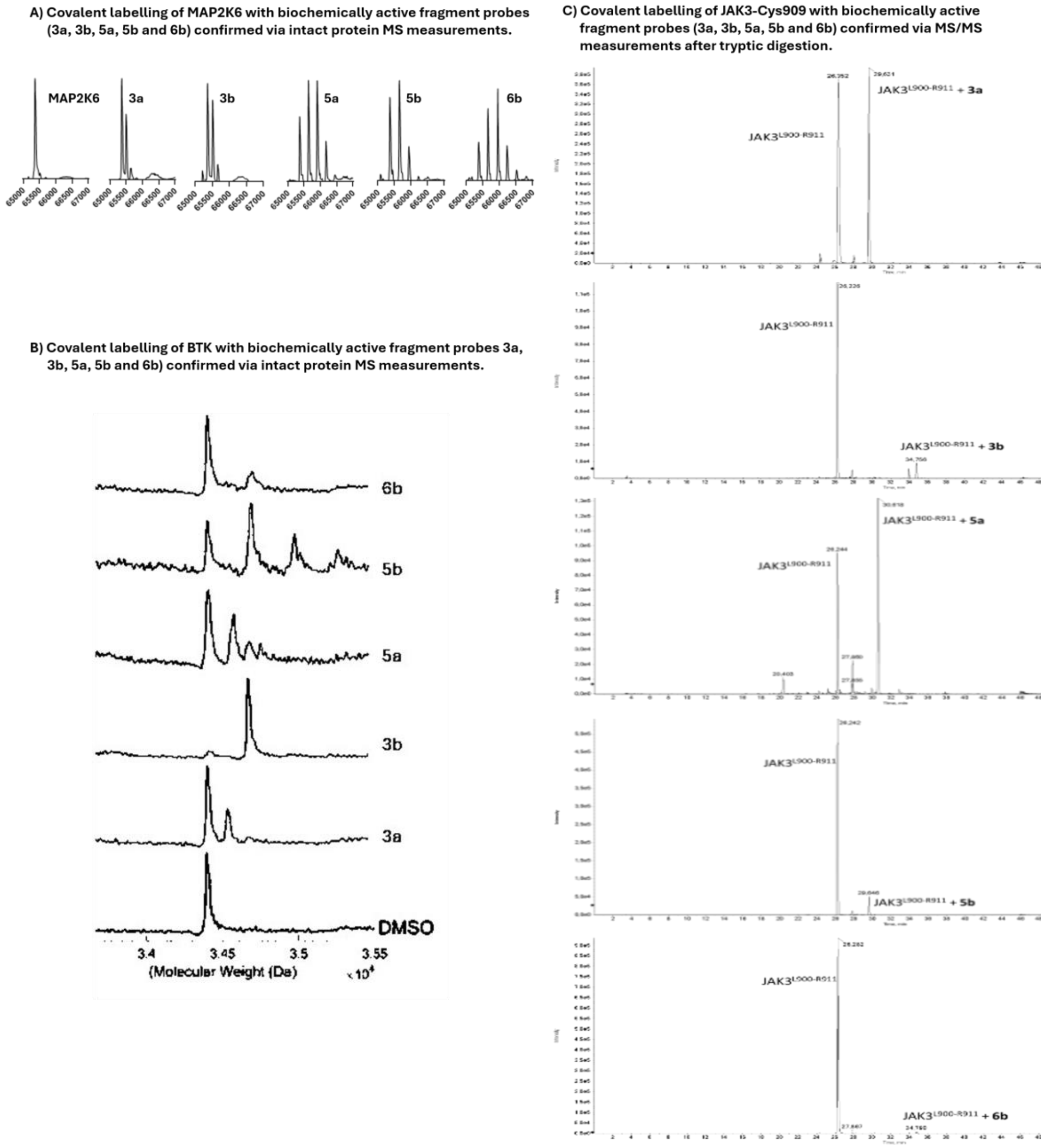

- The degree of intact protein labeling and stoichiometries need to be quantified and discussed for all tested probes and proteins (see comment above).
The extent of protein labeling has been quantified, and the results are now provided in the supplementary material. Since the MS measurement conditions were not

standardized, we do not discuss these data in detail in the main text, as no robust conclusions can be drawn from the heterogeneous results obtained (see Supplementary Information)

Entry	BTK	JAK3	MAP2K6
**3a**	31%	38%*	43%
**3b**	94%	9%*	51%
**5a**	32%	59%*	79%
**5b**	52%	8%*	57%
**6b**	11%	1%*	85%
**9**	-	41%	-
**10**	-	33%	-
**11**	86%	-	-
**12**	93%	-	-
**13**	1%	-	-
**14**	43%	-	-
**15**	13%	-	-

* Labelling ratio on JAK3 was calculated from the measured MS1 intensities of labelled versus nonlabelled peptides (L900-R911) resulted from tryptic digesteion of JAK3.

- The initial reactivity study does not include any alkyl amine-derived thioamide electrophiles, so it is unclear how the aniline-derived electrophiles from the first part of the study compare to the piperidine-based ones used in the second part. These two compound classes should be ranked against each other. I strongly suggest synthesizing benzylamine and piperidine-derived sets of halothioamide/thioacrylamide fragments and testing these in the GSH assay for comparison. It would also be important to show if the decreased reactivity commonly observed for N-alkyl acrylamide versus N-aryl acrylamide transfers to thioacrylamide. Accepting the comment of the referee we synthesized the following compounds: N-benzyl-chloroacteamide, N-benzyl-chlorothioacetamide, N-benzyl-fluorothioacetamide, N-piperidinyl chloroacetamide, N-piperidinyl chlorothioacetamide, N-piperidinyl fluorothioacetamide. These compounds were evaluated in the kinetic GSH assay as well as in the oligopeptide-based selectivity assay, and the results are now discussed in the main text. In brief, the new data do not alter the overall conclusions but provide significant confirmation of the O/S exchange effect originally reported in this study. As our attempts to prepare thioacrylamides were unsuccessful in most cases, we finally decided to remove all thioacrylic and acrylic probes from the study and instead focus exclusively on the haloacetamide and halothioacetamide series. The revised library now comprises 16 probes across four scaffolds (phenyl, bistrifluorophenyl, benzyl, and piperidyl), representing a robust and well-designed covalent fragment set.

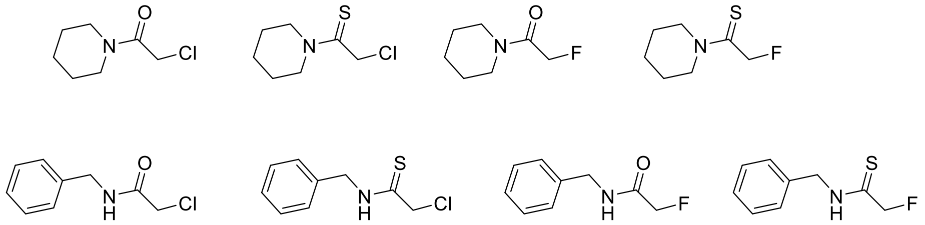

- If the piperidine-derived thioacrylamide proofs to be significantly less reactive than the above aniline derivatives, this electrophile should be included in the TCI part of the study.

We agree with the reviewer that the piperidine-derived thioacrylamide would represent an interesting electrophile to investigate. Therefore, we synthesized and tested a phenyl-thioacrylamide fragment (**6a**). This compound was hyper-reactive and labelled GSH promptly. Any further attempts to prepare thioacrylamides were unsuccessful due to the extremely high reactivity and the limited stability of these compounds. For this reason, as stated above, we finally decided to remove all thioacrylic and acrylic probes from the study and instead focus exclusively on the haloacetamide and halothioacetamide series. The revised library now comprises 16 probes across four scaffolds (phenyl, bistrifluorophenyl, benzyl, and piperidyl), representing a robust and well-designed covalent fragment set. In the revised manuscript, we discuss the presented thioamide-based warheads as a novel option for bioconjugation strategies, including TCI development, ADC design, and target identification. We believe that the chloro- and fluorothioacetamides introduced herein represent valuable new tools for the medicinal chemistry and chemical biology communities, opening avenues for next-generation covalent drug discovery.

- TCI part, kinase inhibition profiling: For rigorous characterization of covalent inhibitors, especially if different warheads/electrophiles are to be compared, the contributions of non-covalent and covalent binding need to be deconvoluted (i.e., KI and kinact). Kinetic assays are now readily available for BTK, and such data must be complemented accordingly. The fact that probe 14 does not seem to modify the enzyme at all but still has a relatively potent IC50 of 45 nM should also be discussed. It seems that the covalent mode of action does not contribute much to the observed activity, in line with the slow labeling kinetics shown in the supplementary information. Interestingly, probe 13 shows very slow labeling kinetics in MS but higher potency than 12 and Ibrutinib, which have much faster labeling kinetics. This suggests that the observed boost in activity is due to non-covalent interactions. This further supports the critical need for kinact and KI determination for these derivatives and a more elaborate discussion of the results.

We thank the reviewer for this insightful comment. To address it, we deconvoluted the contributions of non-covalent and covalent binding to BTK by determining K_I and k_{inact} values in kinetic assays for the ibrutinib derivatives (compounds **11–15**). The results confirmed the significant role of non-covalent interactions, in line with the trends anticipated from our preliminary reactivity experiments. These findings are now discussed in detail in the main text, and the corresponding data are reported in the Supplementary Material.

For these measurements, we established a BTK activity assay based on ADP detection. Briefly, BTK was pre-incubated with the covalent probes, followed by addition of substrate and ATP. After further incubation to allow phosphorylation, the produced ADP was quantified using the ADP Hunter method (Eurofins DiscoverX). This experimental setup enabled us to measure IC□□ values in parallel and to determine k_{inact}/K_I parameters, providing a more rigorous characterization of the mode of action of the tested probes.

IC_{50} [nM]	11 (Ibrutinib)	12 (chloro-acetamide)	13 (fluoro-acetamide)	14 (chloro-thioacetamide)	15 (fluoro-thioacetamide)
0p	16.6±2.2	21.9±3.5	31.5±8.1	13.5±2.9	26.8±4.2
1p	9.5±1.7	12.9±2.2	26.9±6.0	5.3±1.4	15.3±3.3
2p	N/D	6.5±1.4	24.6±6.6	2.0±0.5	8.6±1.5
5p	2.8±0.7	2.2±0.4	20.0±6.1	1.0±0.2	4.5±1.1
15p	1.5±0.2	1.8±0.2	15.1±3.8	0.9±0.2	3.1±0.7
k_{inact} [min^{-1}]	0.44	1.80	0.016	49.5	3.99
K_I [uM]	0.0035	0.033	0.0006	0.332	0.097
k_{inact} / K_I	125	55	27	149	41

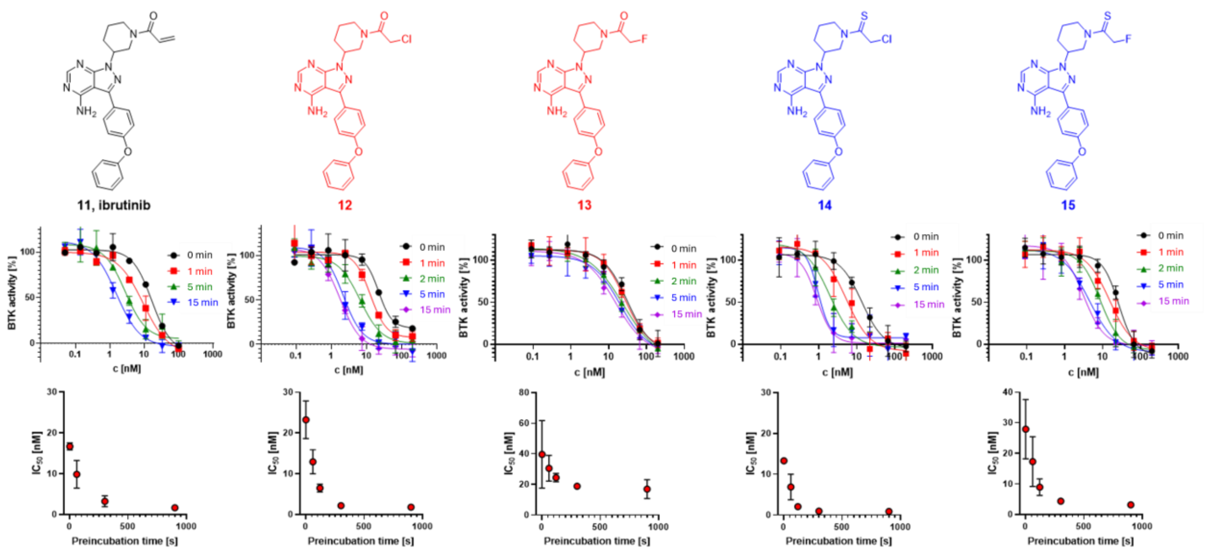

- The ADC part of the study is interesting, but the limited stability of the linkage and loss of cargo is a common problem encountered in ADCs. It is suggested to conduct experiments to prove that the bond is indeed stable under physiologically relevant conditions.

The stability of the chloroacetamide- and thiochloroacetamide-based conjugates was evaluated under physiologically relevant conditions, and the results have been incorporated into the main text. To this end, we reproduced the TAMRA-labelled

antibody conjugates and assessed their stability by SDS-PAGE after incubation at 37 °C in two media: PBS (pH 7.4) and a reductive PBS buffer (pH 7.4, supplemented with 5 mM GSH). In both cases, the conjugates remained stable, including those prepared with the novel thiochloroacetamide warhead.
It is worth noting that for the fluoroacetamide and fluorothioacetamide analogues, the conjugation mechanism involves displacement of the halogen adjacent to the carbonyl or thiocarbonyl group, resulting in the same final acetamide or thioacetamide linkage as observed with the chloro-compounds. Therefore, the stability data obtained for the chloroacetamide- and thiochloroacetamide-derived conjugates can be considered representative for their fluoro analogues as well, making additional measurements unnecessary.

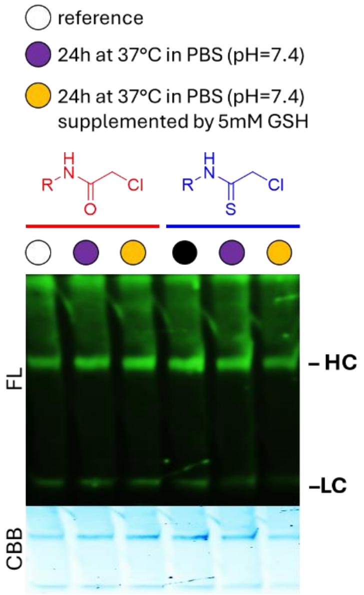

- Considering the potential application of these thioamide-derived warheads in TCIs, and the known susceptibility of sulfur to oxidation, some investigation of metabolism, at least in vitro, would be a beneficial addition to the data presented.
We thank the reviewer for this valuable suggestion. To address it, we performed in vitro human liver microsome metabolism experiments and assessed the remaining compounds by HPLC measurements. In brief, both the chloroacetamide and chlorothioacetamide derivatives displayed a similar range of metabolic stability, with 1–4% of the compound remaining after 30 minutes. In contrast, the moderately reactive but proven covalent fluorothioacetamide derivative showed increased stability, with 10% remaining after 30 minutes.

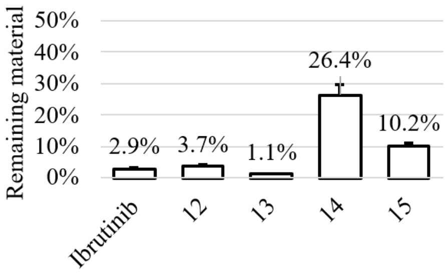

Minor Points:

- Title/manuscript: "Thioamide warhead" should be rephrased to "thioamide-derived warhead" or similar as thioamide itself is not the warhead functional group.
The title has been changed to “Thioamide-derived warheads with enhanced cysteine reactivity and specificity for covalent protein labelling”

- Page 2: "were mostly avoided from drug discovery pipelines due to potential idiosyncratic toxicity issues" could also mention that lack of selectivity was often a factor associated with covalent drug modalities.
The suggested text was added.

- Page 2: "the different reactivities of warheads" could mention that warhead topology, sterics, and polar features also play a role.
Warhead topology, sterics, and polar features are now mentioned providing a more sophisticated discussion of the different reactivities of warheads.

- Page 2: Instead of "electrophile without changing its geometry," use "electrophile without significantly changing its geometry."
Changed as suggested.

- In the introduction, the authors may discuss the reaction types more clearly to provide context. Notably, the C=S bond is not directly involved in the displacement of alpha leaving groups. Although the reasoning sounds logical, the impact of the C=S bond on the softness of the beta-position in thioacrylamide electrophiles should be discussed with suitable citations or backed by own data.
We have clarified in the text that the C=S bond is not directly involved in the displacement of α-leaving groups, but it does alter the electronic environment of the adjacent β-position. To support this, we performed DFT calculations (M062X/6-311++G(d,p)) and analyzed the NBO- charge distribution localized to the adjacent carbon atom in β-position. These results consistently showed a positive shift of charge on the carbon adjacent to the thiocarbonyl group compared to the corresponding carbonyl analogue, indicating its increased electrophilic character. This supports our conclusion that thioamides act as more potent electrophiles due to these intrinsic electronic effects.

**NBO charge distribution**	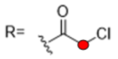	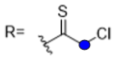	**ΔNBO$_{O \to S}$**	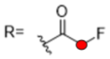	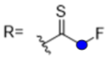	**ΔNBO$_{O \to S}$**
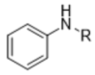	-0.454	-0.400	**+0.054**	0.003	0.029	**+0.026**
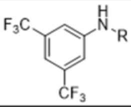	-0.459	-0.402	**+0.057**	0.002	0.031	**+0.029**
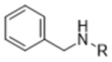	-0.437	-0.398	**+0.039**	0.007	0.034	**+0.027**
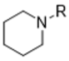	-0.461	-0.443	**+0.018**	-0.004	0.017	**+0.021**

- Page 3: "Isothiocyanates (4a-b) were reacted with vinylmagnesium bromide (5) Grignard-reagent (Scheme 1b), while 6a was obtained in sufficient purity," needs rephrasing. Please mention the warhead chemotype.
As we finally excluded acrylic/thioacrylic probes from the covalent fragment set under investigation, we also removed the discussion of their synthesis, therefore the addition of further details is considered unnecessary.

- Table 1 and discussion: Conditions (pH, solvent, temperature, concentrations) should be mentioned. Notably, it seems phenylacrylamide, a common standard, behaves relatively unreactively here (GSH t1/2 is often cited in the range of ~1-3h). It should also be mentioned if the depicted values are corrected for degradation or not.
We thank the reviewer for pointing this out. We have now added the experimental conditions (pH, solvent, temperature, and concentrations) to the caption of Table 1. Regarding the reactivity of phenylacrylamide, we note that its relatively less reactivity in our experiments likely reflects the specific buffer system, lower incubation temperature (25°C vs. 37°C) and concentration settings we used, which can differ from conditions in previously cited studies reporting shorter half-lives. Furthermore, we systematically applied stability correction for background degradation of the electrophiles, which can substantially affect the apparent reactivity. These corrections provide a more reliable assessment of intrinsic thiol reactivity. We have clarified this in the revised text.

- Page 3: "Our results confirmed that O-S exchange increased the thiol-reactivity," is an overgeneralization. While the GSH data support this statement for AA/TAA, differences are fairly minor for the CA/CTA and FA/FTA matched pairs; CTA with R=H has essentially the same stability as its CA analog. Please refine this statement.
We refined the wording as follows: *"Our results confirmed that O–S exchange positively contributed to the thiol reactivity of the covalent probes investigated herein."* By extending the covalent fragment-level investigations with eight additional probes, we obtained solid support for the conclusion that the increase in thiol reactivity upon O–S exchange generally holds true. However, we acknowledge that this is not an exclusive rule, but rather a useful option for structure optimization.

- Page 3: "seen for the quasi non-reactive fluoroacetamide 10b" is not accurate. In the GSH assay, compound 10b is surprisingly more reactive than phenylacrylamide (3a), which is known to possess significant reactivity (see e.g., doi 10.1021/jm501412a).
The wording has been corrected to avoid the misleading term "quasi non-reactive." The revised text now reflects our observations more accurately.

- Page 5, KGDYHFPIC peptide: Notably, Cys and Lys are terminal in this peptide which may impact their reactivity. Could the authors explain why this peptide was chosen and mention the possible impact of the terminal positioning?
Although we agree that the terminal position of Cys and Lys might impact their reactivity, we used the same peptide in multiple papers (https://doi.org/10.1016/j.ejmech.2018.10.010;

https://doi.org/10.1016/j.ejmech.2020.112836;
https://doi.org/10.1002/cbic.202000700;
https://doi.org/10.1016/j.ejmech.2022.114163;
https://doi.org/10.3390/molecules28073042) and it was generally found to be useful for the first assessment of labelling specificity.

- "data suggest O-S exchange as an attractive electrophile option that increases the reactivity," should have more nuanced phrasing given the limited and heterogeneous data.
The wording has been adjusted to more accurately reflect the perspective of our findings. The revised sentence now reads: *"Our data suggest that O–S exchange could be an attractive electrophile modification strategy, which in most cases increases thiol reactivity, although its impact depends on the electronic properties of the noncovalent scaffold as well as the nucleophilicity of the targeted residue."*

- Page 5: "To understand and explain the observed reactivity differences of haloacetamides," the authors may mention that the nucleophilic attack presumably proceeds via SN2 and does not involve an attack on the (thio)carbonyl carbon.
The suggested details were added to the discussion.

- Figure 1: Given the low number of data points, assignment of individual data points to compounds should be made to increase comprehensibility.
We updated Figure 1 to assign individual data points to the corresponding compounds, thereby improving clarity. In addition, we extended the dataset with 10 further covalent fragments as clarified above, which are now also depicted with their calculated versus experimental reactivity.

- Figure 2 caption: please correct "Inhibiton by fargments"
Corrected.

- Page 6: "To evaluate their kinase targeting potential we have chosen six, covalently druggable, kinases representing each major kinase branch," the authors may comment on the positioning of the cysteines in the kinases and how this impacts their accessibility.
The target kinases were selected as therapeutically significant and already covalently targeted members of the protein kinase family. Taking into account the availability of covalently targetable cysteine residues (https://doi.org/10.7717/peerj.126), and based on the availability of LCMS-measurements of those targets, finally 3 kinases were selected: JAK3 and BTK from the TK (tyrosine kinase) branch, and MAP2K6 from the STE (yeast sterile 7-,11- and 20-homologue kinases) branch. Positioning of the targeted cysteines and characterization of their reactivity and accessibility based on *Cy-preds* server predictions (https://doi.org/10.1002/prot.24978; http://bioinformatics.akdeniz.edu.tr/cypreds/start.py) are summerized below. Also, we added some further information to the main text to discuss the covalent tractability of the cysteines in those 3 included kinases. Interestingly, those investigated kinases characterized by similar accessibility, yet significantly different reactivity on their targeted cysteine residues.

Protein	Kinome tree major branch	Targeted residue	Region of the targeted residue	pKa	SASA* [Å^2]
BTK	TK	C481	front pocket	6.1	25.4
MAP2K6	STE	C196	DFG	11.1	25.6
JAK3	TK	C909	front pocket	4.7	25.5

* cysteine sulfur atom solvent-accessible surface area

- Page 6: "suggesting that the O-S exchange can be further supported by the electron-withdrawing character of the substituents," should be rephrased as this is not surprising and is part of the rationale for including the CF3 compound.
The sentence was rephrased to avoid overstating the observation. The sentence now reads: *"(the results)… consistent with the expected electron-withdrawing effect of the substituents"*

- Page 7: Cys 909 labeling in JAK3: Were other labeling sites also observed?
No other labelling sites were detected.

- Page 8: "covalent labeling of BTK with efficacy proportional to the reactivity of the warheads," should note that potency is not correlated with MS labeling efficiency.
The wording has been revised to clarify that while covalent labeling of BTK was observed, the potency of the probes did not correlate directly with MS labeling efficiency.

- Page 9: The statement "18 labeled a complementary subset of proteins as shown in the Venn diagram" would benefit from some discussion to rationalize this finding.
We expanded the discussion to rationalize the finding that compound **18** labeled a complementary subset of proteins. As described in the revised text, this pattern likely reflects differences in the distinct reactivity of the warhead, which result in a complementary selectivity profile relative to the other probes.

- Figure 3 has a distorted scaling.
Corrected.

- Page 13: For the kinase assays, could the authors please specify whether these assays were conducted by a CRO or by the authors themselves? The same should be done for the BTK radioligand assay.
Kinase assays were done by Life Technologies via the Z'-LYTE™ Kinase Assay Platform. For BTK we applied the HitHunter® Kinase Enzyme Activity Assay Technology (an ADP-measurement-based kinase activity assay) by purchasing ready-to-use BTK-assay kit (https://www.discoverx.com/catalog/btk-kinase-enzyme-activity-assay-kit) from Eurofins DiscoverX. The assay was performed in-house by the authors.

- Color-coding in figure S3 is unclear (e.g., left column 8% inhibition red; 13% inhibition green).

We corrected Figure S3, and the color coding has now been standardized uniformly across the entire panel.

- Error bars are needed for kinase assay curves in the supplementary information.

Error bars have been added to the kinase assay curves. We also note that all BTK activity was re-measured using the in-house BTK activity assay, and these updated results are now reported within the manuscript.

Reviewer #3 (Remarks to the Author):

This manuscript written by Petri et al. describes the O-S exchange strategy as late-stage modifications to offer halothioacetamide electrophiles that can be applied to the targeted covalent inhibitor (TCI) and antibody-drug conjugate (ADC) design. Sulfur exchange of oxygen generally increases the reactivity of warheads and can broaden the scope of target proteins (and cysteines). Four different aspects were explored for this new type of electrophiles: 1) kinase mapping with fragments, 2) covalent BTK inhibition, 3) antibody conjugation, and 4) chemoproteomics. Overall, the experiments were well-designed and executed. To provide clearer insight into thioamide warheads in comparison to amide warheads and strengthen the study, some additional experiments (see below specific comments) can be performed.
We are grateful for the positive evaluation of our work and for the comments contributed to completing the study. We believe that the expertise of the referee helped us to show the full potential of the suggested methodology.

Specific comments:
1) In Figure 1, labeling individual probes for each data point would be more helpful than the group analysis of amide probes vs. thioamide probes.
We updated Figure 1 to assign individual data points to the corresponding compounds, thereby improving clarity. In addition, we extended the dataset with 10 further covalent fragments including benzyl and piperidyl amides, which are now also depicted with their calculated versus experimental reactivity.

2) What do authors think about why 9b shows decreased inhibition of JAK3 compared to 9a while 8b shows increased inhibition than 8a? Similarly, why is 11b showing increased inhibition of JAK3 and RSK2 compared to more reactive 9b?
The observed inhibition depends on multiple factors, including the electrophilicity of the labeling fragment and the accessibility and nucleophilicity of the targeted residues. To rationalize the observed inhibition patterns, we assessed these properties for each kinase target, as summarized below. For JAK3, the targeted cysteine is highly acidic but exhibits only moderate accessibility, suggesting that steric effects may override intrinsic reactivity. This may occur, for example, when the noncovalent scaffold is switched from phenyl to the bulkier bistrifluoromethyl-phenyl, even though the electrophilic reactivity of the warhead is enhanced by strong electron-withdrawing groups. While a deeper analysis of binding poses could provide further insights, this was beyond the scope of the current study. The key message remains that O–S exchange represents an attractive electrophile modification strategy, which in most cases increases thiol reactivity, although its effect depends on the electronic properties of the noncovalent scaffold as well as the nucleophilicity and accessibility of the targeted residue.
However, we note that finally taking into account the availability of covalently targetable cysteine residues (https://doi.org/10.7717/peerj.126), and based on the availability of LCMS-measurements of those targets, finally 3 kinases were selected: JAK3 and BTK from the TK (tyrosine kinase) branch, and MAP2K6 from the STE (yeast sterile 7-,11- and 20-homologue kinases) branch. Positioning of the targeted cysteines and characterization of their reactivity and accessibility based on *Cy-preds*

server predictions (https://doi.org/10.1002/prot.24978; http://bioinformatics.akdeniz.edu.tr/cypreds/start.py) are summarized below.

Protein	Kinome tree major branch	Targeted residue	Region of the targeted residue	pKa	SASA* [Å^2]
BTK	TK	C481	front pocket	6.1	25.4
MAP2K6	STE	C196	DFG	11.1	25.6
JAK3	TK	C909	front pocket	4.7	25.5
RSK2	AGC	C229	DFG	6.8	81.9

* cysteine sulfur atom solvent-accessible surface area

3) Kinase mapping data indicate that the O-S exchange modulates not only the overall reactivity but also the target profile which needs to be mentioned and discussed more clearly.
We agree that differences in the accessibility and nucleophilicity of the labeled residues can influence reactivity, resulting in distinct target profiles. In response to the reviewer's comment, we predicted the accessibility and nucleophilicity of the targeted residues for each kinase, and this analysis is now discussed in the main text.

4) The late-stage modification of TCIs using O-S exchange is exciting. But, as the intrinsic reactivity of halothioacetamides is higher than corresponding haloacetamides or acrylamides, it's also possible that off-target reactivity is increased. Thus, it is important to assess and compare the global protein reactivity of TCIs containing thioamide. Authors can perform chemoproteomics analysis with alkyne-functionalized compounds or competitive chemoproteomics using a broad-spectrum cysteine reactive probe.
Accepting the comment of the referee, we assessed the off-target potential in chemoproteomics experiments treating Mino cells with alkyne-functionalized **16-18** probes. The results are now discussed in the main text, while raw data are provided in the Supplementary Information and proteomic datasets have been deposited in the PRIDE repository, as described.
Specifically, alkyne analogues of compounds **16** (ibrutinib analogue), **17** (chloroacetamide), and **18** (chlorothioacetamide) were synthesized and profiled at 10 μM concentration. In all cases, BTK was retrieved among the top-enriched targets, confirming efficient on-target engagement. At the proteome-wide level, compound **16** labeled 4222 proteins, while **17** showed broader engagement with 5457 proteins. Importantly, **18** displayed a slightly narrower scope (5312 proteins), falling closer to the marketed ibrutinib analogue **16** than to **17**. Intersections revealed 3888 proteins commonly captured by all three probes, with 909 shared exclusively between the two chloroacetamides (**17** and **18**). Notably, when the probe concentration was lowered to 1 μM, the number of off-targets for **16** was drastically reduced, whereas the profiles for **17** and **18** remained largely unchanged.
Together, these experiments confirm that O-to-S exchange does not exacerbate proteome-wide promiscuity. Despite the experimentally and computationally demonstrated increase in intrinsic reactivity and potency, probe **18** showed a slightly better off-target profile than probe **17**. These findings strongly support that thioamide

substitution is a valuable late-stage warhead optimization strategy, enhancing covalent efficiency without increasing global off-target liabilities.

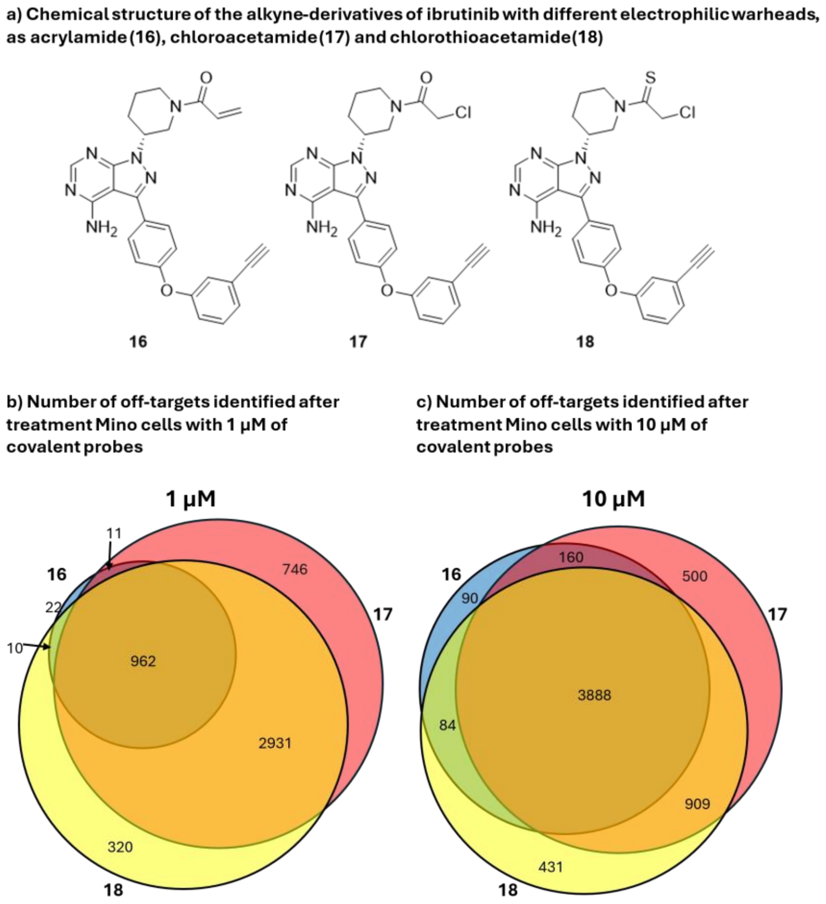

5) For antibody conjugation, FARs are similar between haloacetamides and halothioacetamides. Although this study provides additional electrophiles that can be applied to ADCs, the added value of using thio-probe is not clearly demonstrated. Kinetic measurement can provide some more insight into the applicability of these warheads.
We thank the reviewer for this valuable comment. Following the suggestion, we performed time-dependent UV-FAR analyses to compare the conjugation kinetics of haloacetamide and halothioacetamide probes. Antibody labeling was monitored over 90 minutes at five time points (5, 10, 30, 60, and 90 min). These experiments revealed distinct kinetic behaviors between the warheads. While the final UV-FAR values obtained with chloroacetamide and chlorothioacetamide were comparable, the thio-analogue reached its maximum labeling much faster (within 30 min), consistent with its higher intrinsic thiol reactivity. In contrast, fluorothioacetamide showed a more extended labeling process compared to fluoroacetamide, which plateaued early at lower FAR values.
Although a deeper kinetic and mechanistic analysis would require additional dedicated studies beyond the scope of the present work, these results provide new insight into the reactivity profiles of thioacetamide electrophiles in an ADC-relevant context. These findings demonstrate that thioacetamide-based probes are fully

compatible with antibody conjugation and can offer kinetic advantages, thereby expanding the electrophile toolkit for both TCI and ADC development.

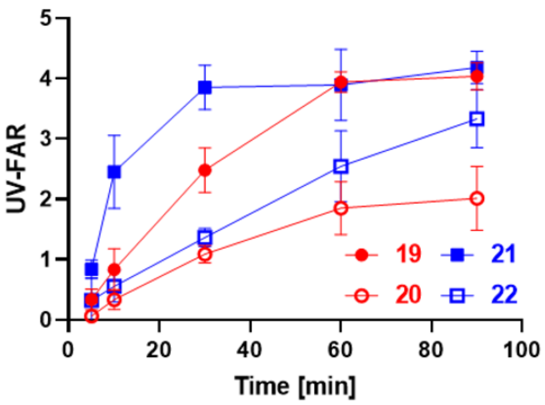

6) Does the IAA alkyne probe used in chemoproteomics contain the same scaffold as other probes?

No, the IAA alkyne probe used in the chemoproteomics experiments is a linear, aliphatic probe commonly used in the literature. The structures of all chemoproteomic probes are now clearly disclosed in the manuscript.

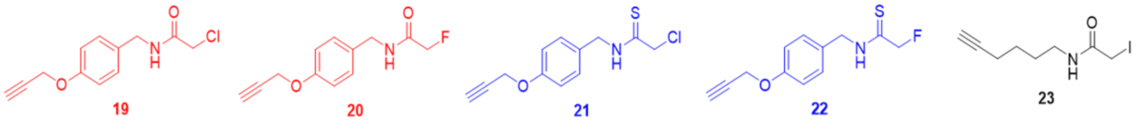

7) It's surprising that fluorothioacetamide probe 20 labels more proteins (2708) than chloroacetamide probe 17 (1453). How do authors interpret this data?

We agree that these results might be surprising; however, translating surrogate- and protein-level reactivity trends to proteome-wide labeling is never straightforward, and differences observed at the fragment or isolated protein level do not always correlate directly with cellular target engagement. Furthermore, the pull-down chemoproteomics results were also consinstent with the gel-based ABPP experiments. Nevertheless, the observation that the fluorothioacetamide probe **22** labels more proteins (2708) than the chloroacetamide probe **19** (1453) can be rationalized by multiple factors. Replacement of oxygen with sulfur may increase the lipophilicity of the warhead, favoring interactions in hydrophobic pockets and effectively increasing residence time, which facilitates labeling of otherwise challenging sites even for warheads with intrinsically lower thiol reactivity. A comprehensive proteome-wide kinetic study combined with proper identification and modeling of the binding sites would be required to fully dissect these contributions, which is beyond the scope of the present work.

8) Comparative analysis (Venn diagram) of proteins labeled by 17, 18, and 20 (in addition to IAA and 18) would help understand the target scope. Similarly, in addition to the number of proteins identified, the number of cysteines modified will be important to note.

The requested data are now included in Supplementary Table S8, and Figure 6d has been updated to provide a comparative Venn diagram of proteins labeled by probes

19, **21**, and **22** in addition to comparing IAA (**23**) and **21**, thereby illustrating the relative target scope more clearly.

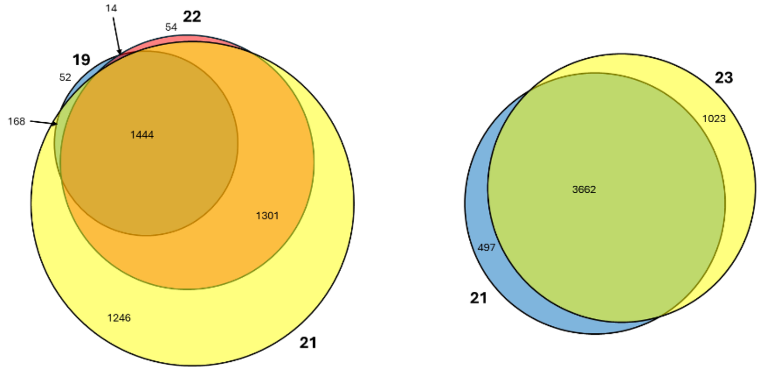

To address the number of modified cysteines, we performed additional chemoproteomics experiments with compounds **19–22**. Interestingly, we observed a surprisingly low abundance of cysteine identifications for the thioacetamide probes, which contrasts with the robust labeling we detected by gel-ABPP and pull-down experiments. Importantly, no evidence of labeling non-cysteine amino acids was detected beyond very minor background signals. Specifically, the chloroacetamide alkyne probe (**19**) yielded 377 labeled amino acids, including 352 Cys (93.3%), 5 Lys (1.3%), 9 His (2.4%) and 11 Tyr (3.0%), while the chlorothioacetamide alkyne probe (**21**) resulted in 864 identifications, including 764 Cys (88.4%), 19 Lys (2.2%), 44 His (5.1%) and 37 Tyr (4.3%).

a) Venn diagram showing the distribution of identified peptides that were labelled by 19 and 21 probes.

b) Proteome-wide residue specificity of chloroacetamide (19) and chlorothioacetamide (21) probes. Shown are the percentages of cysteine, lysine, histidine, and tyrosine residues identified as probe-modified sites.

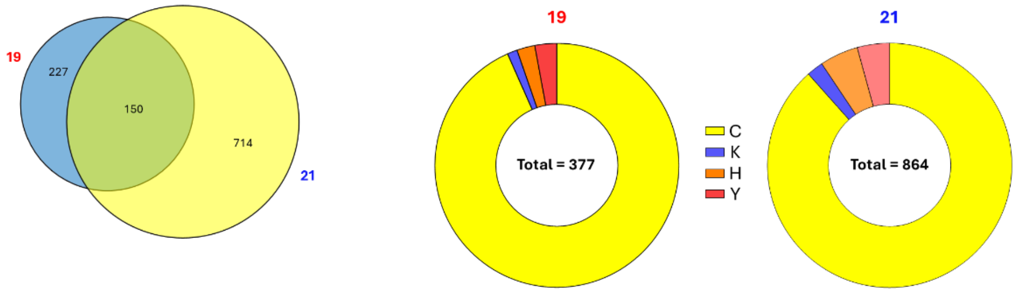

We believe the apparent discrepancy between protein-level pull-down data and residue-level site mapping most likely reflects methodological limitations. In particular, the cysteine adducts formed by thioacetamide warheads may display lower stability in and recovery during peptide-level sample preparation, which could lead to underestimation in site-level MS analysis. By contrast, pull-down and gel-ABPP assays capture intact probe–protein conjugates and therefore more faithfully reflect the true labeling efficiency. Altogether, these results indicate that thioacetamide probes achieve robust protein-level labeling, while peptide-level MS/MS may underestimate their coverage due to technical constraints.

9) Further validation of selected proteins that were uniquely modified by probe 18 (confirmation of covalent conjugation and identification of the binding site) would strengthen the authors' claim that thioprobes label a distinc subset of the proteome compared to the conventional iodoacetamide probe.

We thank the reviewer for this valuable suggestion. Following the comment, we carefully evaluated the proteins uniquely identified with probe **21** and finally we focused on PDE6δ as a particularly alluring target. PDE6δ plays a key role in KRAS biology by stabilizing and shuttling C-farnesylated KRAS through the cytosol until its integration into the plasma membrane (https://doi.org/10.1021/acs.jmedchem.1c01265). Although PDE6d contains two cysteine residues facing into the farnesyl-binding pocket (based on 7PAC PDB structure), to our knowledge no covalent binders of PDE6δ have previously been reported in chemoproteomic target identification campaigns. This rendered PDE6δ an especially attractive candidate for validation.
To this end, we purchased recombinant PDE6δ (Novus, NBP1-44480) and confirmed covalent labeling by probe **18** by intact protein MS analysis. Furthermore, subsequent enzymatic digestion with Glu-C and MS/MS analysis identified Cys56 as the covalent binding site of the chlorothioacetamide (**21**) probe. This represents, to our knowledge, the first demonstration of covalent modification of PDE6δ, a therapeutically relevant target implicated in KRAS-driven tumor development.
While full biological follow-up is beyond the scope of this study, we also sought to assess whether covalent labeling had a functional impact on PDE6δ. For this, we applied a fluorescence polarization assay widely used to evaluate PDE6δ inhibitors, based on displacement of a TAMRA-atorvastatin tracer (https://doi.org/10.1021/acs.jmedchem.4c02636; https://doi.org/10.1038/nature12205; https://doi.org/10.1016/j.apsb.2021.07.009). We confirmed tracer binding with a K_d of 154 nM and determined that probe **21** competitively displaced the tracer with an IC_{50} of 54.4μM, corresponding to a K_i of 45.1 μM (based on Cheng–Prusoff correction, generally used for competitive displacement assays).
Altogether, these experiments provide strong orthogonal validation that probe **21** covalently modifies PDE6 δ at Cys56 and functionally interferes with ligand binding. Furthermore, these results can be further rationalized by the protein structure, as Cys56 cysteine residue is close to the farnesyl-binding site (based on 5TB5 PDB structure, https://doi.org/10.1021/cb5002125).
These findings clearly highlight the ability of thioacetamide-derived probes to uncover novel and biologically relevant covalent targets and demonstrate their potential value for expanding target discovery efforts.

A) MS spectra of 21-labelled PDE6δ

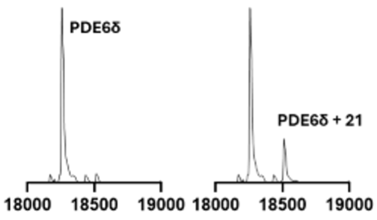

D) K_d-determination of TAMRA-atorvastatin on PDE6δ

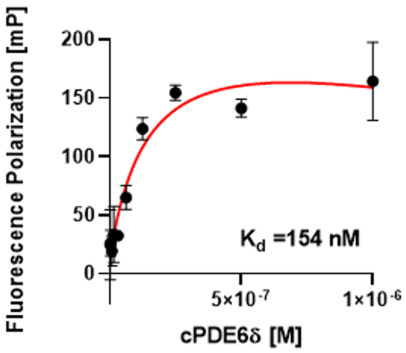

B) TAMRA-atorvastatin fluorescent tracer

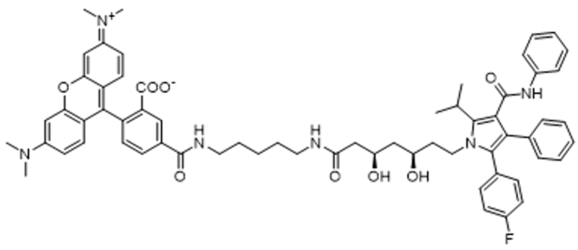

E) Fluorescence plarization measurment of probe 21

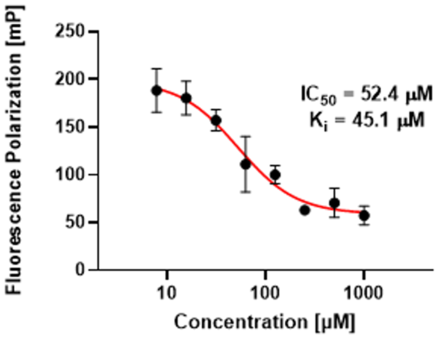

C) MS/MS spectra of 21-labelled PDE6δ after Glu-C digestion identifying Cys56 as the site of labelling

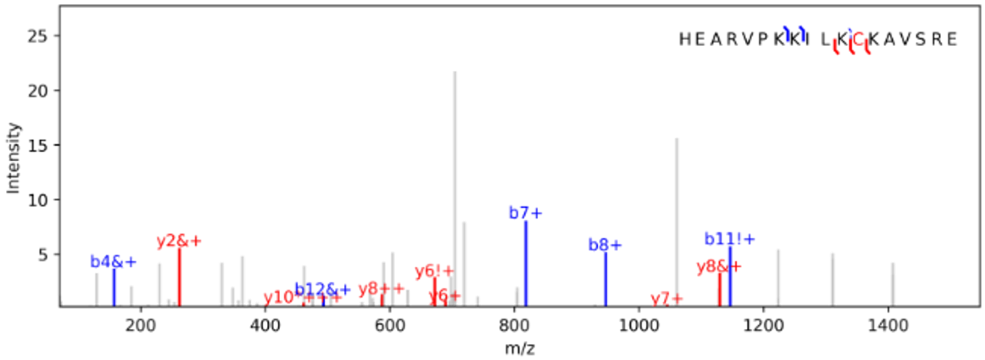

Reviewer #4 (Remarks to the Author):

Keseru and London et al. developed a relatively new cysteine-targeting warheads by transforming traditional carboxamide based warheads to soft electrophilic thioamides. The transformed electrophiles (including chlorothioacetamides and fluorothioacetamides) demonstrated enhanced reactivity and specificity for cysteine labeling while preserving the original warhead geometry. The authors further demonstrated these warheads can be used to develop BTK inhibitors and covalent modify antibody. In addition, the chlorothioacetamides compound (18) was able to covalently bind with more than 4000 proteins. This work can enrich the toolbox of cysteine-targeting warheads, offering new electrophiles for development of TCIs and chemical biology tools. However, the characterization of this kind of warhead is not thorough, and the overall advantages of this kind of warhead are not so significant. So it is recommended to include more results, current version was not good enough to be published in Nature Communications, but it can be given an opportunity to revise it. I will make the final decision based on the revised version.

Major Concerns:
1, The authors claimed the O-S exchange strategy enables precise late-stage modulation of covalent reactivity, which was proved only in Ibrutinib. The authors should include one more case with different structures, to demonstrate its feasibility on more structurally diversity compounds
We thank the reviewer for this important comment. To demonstrate the general applicability of the O-to-S exchange strategy beyond the ibrutinib scaffold, we applied it to a previously published JAK3 inhibitor series developed using our covalent fragment-guided methodology (https://doi.org/10.1016/j.ejmech.2020.112836). This series included two structurally distinct covalent inhibitors: a highly active chloroacetamide (**7**) and a moderately active methyl-substituted chloroacetamide (**8**), which differ in both steric and electronic properties at the electrophilic center. Both compounds displayed good selectivity for JAK3 over related kinases such as BTK. Both chloroacetamide derivatives were converted into the corresponding chlorothioacetamides (**9,10**) using a late-stage O-to-S exchange approach. The resulting thioamide analogues exhibited enhanced potency on JAK3 while maintaining selectivity, with the methyl-chloroacetamide derivative showing a remarkable improvement from 388.3 ± 149.1 nM to 71.2 ± 14.6 nM IC□□ on JAK3 and remaining inactive on BTK at 1 µM. Covalent engagement of the investigated probes (**7-10**) and JAK3 was confirmed *via* intact protein mass spectrometry, verifying successful covalent bond formation. These results can further demonstrate that the O-to-S exchange strategy is feasible across structurally diverse covalent inhibitors and can be used as a precise, late-stage modification to tune reactivity without compromising target selectivity. This provides strong evidence that the approach is generalizable and not limited to the ibrutinib scaffold.

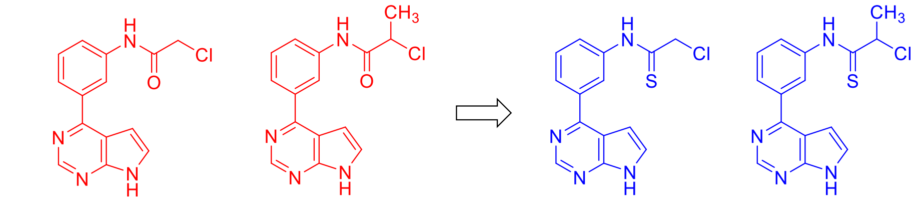

7 JAK3: 30.3 ± 11.2 nM BTK: 170.3 ± 40.6 nM
8 JAK3: 388.3 ± 149.1nM BTK: N/D
9 JAK3: 18.9 ± 4.7 nM BTK: 50.1 ± 12.6 nM
10 JAK3: 71.2 ± 14.6 nM BTK: N/D

2, The authors need to test covalent reaction kinetics of 12/13/15 with BTK, which can demonstrate and compare the covalent binding efficiency of inhibitors containing different warheads with proteins.

Accepting the comment, we tested the kinetics of compounds **12-15**. To address it, we deconvoluted the contributions of non-covalent and covalent binding to BTK by determining K_I and k_{inact} values in kinetic assays for the ibrutinib derivatives (compounds **11–15**). The results confirmed the significant role of non-covalent interactions, in line with the trends anticipated from our preliminary reactivity experiments. These findings are now discussed in detail in the main text, and the corresponding data are reported in the Supplementary Material.

For these measurements, we established a BTK activity assay based on ADP detection. Briefly, BTK was pre-incubated with the covalent probes, followed by addition of substrate and ATP. After further incubation to allow phosphorylation, the produced ADP was quantified using the ADP Hunter method (Eurofins DiscoverX). This experimental setup enabled us to measure IC□□ values in parallel and to determine k_{inact}/K_I parameters, providing a more rigorous characterization of the mode of action of the tested probes.

IC_{50} [nM]	11 (Ibrutinib)	12 (chloro-acetamide)	13 (fluoro-acetamide)	14 (chloro-thioacetamide)	15 (fluoro-thioacetamide)
0p	16.6±2.2	21.9±3.5	31.5±8.1	13.5±2.9	26.8±4.2
1p	9.5±1.7	12.9±2.2	26.9±6.0	5.3±1.4	15.3±3.3
2p	N/D	6.5±1.4	24.6±6.6	2.0±0.5	8.6±1.5
5p	2.8±0.7	2.2±0.4	20.0±6.1	1.0±0.2	4.5±1.1
15p	1.5±0.2	1.8±0.2	15.1±3.8	0.9±0.2	3.1±0.7
k_{inact} [min^{-1}]	0.44	1.80	0.016	49.5	3.99
K_I [uμM]	0.0035	0.033	0.0006	0.332	0.097
k_{inact} / K_I	125	55	27	149	41

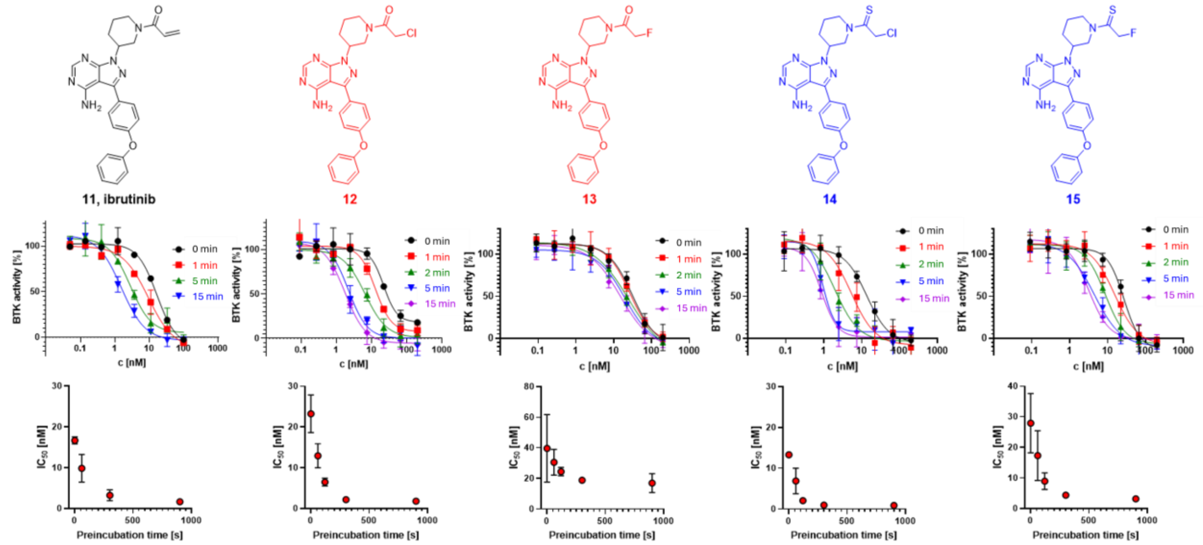

3, The authors should explain properly why they chose “N-benzyl-2-chloroethanethioamide”-based compounds in Fig.3/4 instead of “2-chloro-N-phenylacetamide” what they used in the beginning, these two versions may demonstrate different reactivities.

Accepting the comment of the referee we synthesized the following compounds:

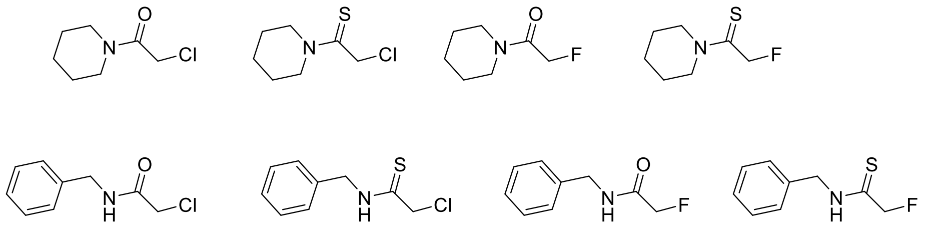

These compounds were investigated in the kinetic GSH assay, NP specificity assay, as well as characterized by theoretically calculated activation energy barriers (ΔG‡) and these additional results are now discussed in the main text. Based on the surrogate-level results, we confirmed that both probes exhibit significant thiol reactivity. However, the benzylic moiety provides conformational flexibility, which we assume contributes to the broad coverage of proteome-wide cysteine targets by reducing sensitivity to steric factors in the vicinity of the targeted residues.

4, The results in Figure 3c are inconsistent with those described in S6. Please explain or describe them more clearly.

We thank the reviewer for pointing out this inconsistency. In Supplementary Table S6 the results of probes **20** and **21** were mistakenly exchanged. This has now been corrected, and the table is fully consistent with the data shown in Figure 3c.

5, The fluorothioacetamides-containing compound 15 demonstrated significantly faster covalent binding rate with BTK comparing with chlorothioacetamides-containing compound 13, however, chlorothioacetamides-containing compound 18 displayed stronger reactivity with cysteine in comparing with fluorothioacetamides-containing compound 20

To address this comment, we performed kinetic measurements to provide deeper insight into the respective contributions of covalent and non-covalent interactions in

the binding process. Specifically, we determined K_I and k_{inact} values in kinetic assays for the ibrutinib derivatives (compounds **11–15**). It is important to note, however, that proteome-wide coverage and binding kinetics on an individual target are not directly comparable measures of covalent potency. The steric and electronic properties of the targeted cysteine within a given protein can strongly influence binding characteristics, and these effects are therefore distinct from the intrinsic proteome-wide reactivity of different electrophilic warheads.

6, The authors claimed O-S exchange to this warheads improved its specificity for cysteine, so it's strongly suggested that the authors should determine proteomewide residue selectivity of the warheads they developed using residue-specific proteomics.
We performed additional chemoproteomics experiments with compounds **19–22** to directly assess proteome-wide residue selectivity. Interestingly, we observed a surprisingly low abundance of cysteine identifications for the thioacetamide probes, which contrasts with the robust labeling detected by gel-ABPP and pull-down experiments. Importantly, we did not detect significant labeling of non-cysteine residues beyond minor background signals. Specifically, the chloroacetamide alkyne probe (**19**) yielded 377 labeled amino acids, including 352 Cys (93.3%), 5 Lys (1.3%), 9 His (2.4%) and 11 Tyr (3.0%), while the chlorothioacetamide alkyne probe (**21**) resulted in 864 identifications, including 764 Cys (88.4%), 19 Lys (2.2%), 44 His (5.1%) and 37 Tyr (4.3%). We believe the apparent discrepancy between protein-level pull-down data and residue-level site mapping most likely reflects methodological limitations, particularly reduced stability or recovery of thioacetamide adducts during peptide-level workflows. Nevertheless, these results clearly confirm that the labeling remains strongly cysteine-selective, supporting our conclusion that thioacetamide probes maintain robust cysteine specificity at the proteome level.

a) Venn diagram showing the distribution of identified peptides that were labelled by 19 and 21 probes.

b) Proteome-wide residue specificity of chloroacetamide (19) and chlorothioacetamide (21) probes. Shown are the percentages of cysteine, lysine, histidine, and tyrosine residues identified as probe-modified sites.

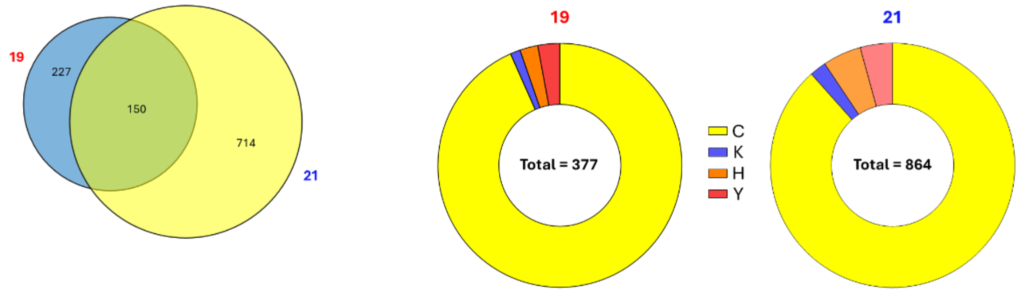

7, As warheads used for development of TCIs, the cellular potencies of chlorothioacetamides and fluorothioacetamides were also need to be evaluated, should include a piece of data to demonstrate the cellular potency for either chlorothioacetamides or fluorothioacetamides, such as evaluating the cellular potency of compound 13 or 15. Especially compound 18 or 20 show poor labeling efficiency in S6.
To address this point, we performed MTT cellular viability assays on Ramos cells to determine in vitro cellular IC_{50} values. The results indicated that the chlorothioacetamide derivative displayed comparable cellular potency to the original chloroacetamide compound (0.21 ± 0.09 µM vs. 0.14 ± 0.04 µM), and significantly

better potency compared to the parent drug (0.87 μM). In contrast, replacement of the chloroacetamide with a fluoroacetamide warhead resulted in a marked loss of activity (30.72 ± 9.86 μM). Importantly, introducing the fluorothioacetamide warhead restored potency by more than an order of magnitude (1.75 ± 0.81 μM), resulted similar range of potency as ibrutinib. These findings are now included and discussed in the manuscript to support that O–S exchange can contribute positively to the efficacy of targeted covalent inhibitors even in a cellular context, particularly when starting from a low-reactivity scaffold.

**Compound**	**Ramos cell viability IC_{50}**
**11** (ibrutinib)	0.94 ± 0.27 μM
**12** (chloroacetamide)	0.14 ± 0.04 μM
**13** (chlorothioacetamide)	0.21 ± 0.09 μM
**14** (fluoroacetamide)	30.72 ± 9.86 μM
**15** (fluorothioacetemide)	1.75 ± 0.81 μM

Minor Issues:

1, Didn't find Supplementary Table S7 which was mentioned line 224 in the maintext.
We thank the reviewer for catching this inconsistency. All supplementary references have been revised and corrected in the manuscript. Specifically, Supplementary Table S7 was mistakenly referenced in the main text; the correct reference is Supplementary Table S6.

2, The authors claimed that "The labelling efficiency of the fluoro (20) and chloro (18) thioacetamide probes was comparable to that of the chloro (17) and iodo acetamide (IAA) probes, respectively" in the line 221-222 according to the results in S6. This statement was not true according to the results in S6. And the results quality in S6 was not good enough, especially for compound 19.
We agree that our original phrasing was not accurate enough and have revised the sentence to state that, in HEK293 lysates, thioacetamide probes showed labeling efficiency comparable to that of the iodoacetamide probe. Importantly, as shown in Supplementary Figure S9, labeling in living cells was notably reduced, which may reflect permeability-related limitations. While pharmacokinetic optimization of thioprobes is beyond the scope of the current study, we recognize this as a valuable direction for future works. Regarding Supplementary Figure S9 (old Figure S6), we agree that the 60sec exposures can result in oversaturated bands for strongly labeling probes such as iodoacetamide, but we deliberately retained both the 10sec and 60sec exposured gel images. For weaker probes, labeling is only visible at longer exposure, whereas shorter exposure is better suited to illustrate differences among stronger probes. Together, the two exposures provide a more comprehensive overview of probe-dependent labeling intensities.
We emphasize, however, that these gel-based experiments were intended only as preliminary illustrations of probe reactivity. To rigorously assess proteome-wide labeling, we performed MS-based chemoproteomics with biological triplicates. These data provide the reliable and quantitative basis for our conclusions and are in good agreement with the gel and pull-down results in terms of labeling efficiency.

3, The starting molecular weight values in Figure S5 should be same, for easy reading.

Figure S5 has been corrected so that the starting molecular weight values are aligned and consistent across all panels.

4, , Line 481 in maintext “followed by 45 incubation at 25 ℃”

The text has been corrected to “45 min incubation at 25 °C” in the revised manuscript.

Reviewer #1

We are grateful to the referee for the detailed comments that helped us to add further details to the computational part of the study. In addition to provide the requested data for reproducibility we performed all the suggested computations requested by the referee. These results are now incorporated to the revised version.

a) I continue to believe that the manuscript does not fit well within the scope of the journal. My general perception is that this manuscript is better suited for a journal specialized in Medicinal Chemistry.

Based on the conversation with the Editor, we respectfully maintain that our manuscript fits within the journal's broad multidisciplinary scope. In addition to medicinal chemistry and chemical biology parts, the study addresses wider biological and methodological aspects that we believe make it interesting for the readership of this journal.

b) The computational section still contains several methodological and technical deficiencies, as well as missing information in both the Methods and Discussion sections. For example, In the Methods section, the authors state: "QM/MM calculations Structure preparations for thermodynamic integration The structure for BTK–Ibrutinib complex (PDB: 5P9J85) was used as a starting structure to build the complexes of compound 12 (O) and 14 (S). The preparation was performed with Schrödinger's Maestro module [Schrödinger Release 2024-3: Maestro, Schrödinger, LLC, New York, NY, 2024.] and included the deletion of the covalent bond between Cys481 and the ligand, the modification of the ligand warhead, and the protonation of the sulfur of Cys481. Restrained minimization was performed to alleviate steric clashes." However, there is no information on the system preparation parameters, such as the treatment of titratable residues, protonation states, or pKa calculations of amino acids. Why was Cys481 protonated? Is there any experimental or theoretical evidence supporting this choice?
The authors must clarify the rationale for this decision and provide sufficient details for reproducibility.

The description of the systems preparation and production runs is extended by giving all parameters to make the calculations reproducible. In particular, we specified that protein preparation was done with the Schrödinger's Protein Preparation Workflow using default parameters, that include the application of the PROPKA method for protonation states and H-bond network optimization. Concerning Cys481 protonation state, we cite its pKa=7.7 obtained by constant pH simulation. Since this pKa value suggests the presence of both protonated and unprotonated cysteine, we prepared both forms and used them in subsequent TI calculations. This paragraph now reads as follows.

"*Structure preparations for thermodynamic integration*
The structure for BTK-Ibrutinib complex (PDB: 5P9J[85]) was used as a starting structure to build the complexes of compound **12** (O) and **14** (S). The preparation was performed with Schrödinger's Protein Preparation Workflow [Schrödinger Release 2024-3:, Schrödinger, LLC, New York, NY, 2024.] using default settings that includes H-bond assignment optimization with PROPKA [https://doi.org/10.1021/ct100578z]. The covalent bond between Cys481 and the ligand was deleted, and the ligand warhead was modified. Cys481 was set either to thiol or to thiolate as both forms are present owing to its low pK_a of 7.7 [https://pubs.acs.org/doi/10.1021/acs.jmedchem.1c01186]. Restrained minimization was performed to alleviate steric clashes."

c) The authors describe their TI setup as follows: "Thermodynamic integration was carried out by the AMBERtools24 software package (Amber 2024, University of California, San Francisco, CA, USA)... [etc.]" However, the methodology is inadequately described. The authors should include a thermodynamic cycle to illustrate the perturbations performed. Moreover, a simulation time of only 20 ps per window is insufficient to ensure proper sampling. The absence of replicate simulations further undermines the statistical reliability of the reported free energies.

The thermodynamic cycle is presented in Figure A1. TI calculations are now reported for complexes with both protonated and unprotonated Cys481. Three parallel simulations for both forms were performed, each with 200 ps production run. The free energy change of each perturbation is reported in Table A1. The description of the extended TI calculations is as follows:

"*Thermodynamic integration*
Thermodynamic integration was carried out by the Amber software package (Amber 2024, University of California, San Francisco, CA, USA). Both the complexes and the ligands were immersed into an octahedral water box and neutralized by sodium ions. The systems were subjected to the relaxation protocol with 1000 steps of steepest descent minimization, 20 ps NVT heating to 300 K and 20 ps of NPT equilibration with the coupling parameter value set to 0.5. The alchemical transformations involved 3 steps: The removal of the softcore atom's partial charges, the transformation of the uncharged softcore atom into the perturbed structure (VdW step) and the reintroduction of the partial charge for the perturbed structure (Figure A1). Every simulation involved MD runs with lambda coupling parameter values set from 0.0 to 1.0 with 0.1 increment. Every lambda „window" contained a 20 ps long heating to 300 K in NVT ensemble and a 200 ps long thermodynamic integration, also in NVT ensemble with 1 fs timestep. SHAKE[86] was enabled for the non-TI region atoms, NOSHAKE was applied for the softcore hydrogens. TI was performed in triplicate both for the thiol and thiolate forms of Cys481. Results are shown in Table A1."

Figure A1. *Thermodynamic cycle of ligand mutations (LO, LS: ligands; E: enzyme; S: solvent ; ligands with charged softcore atoms are framed)*

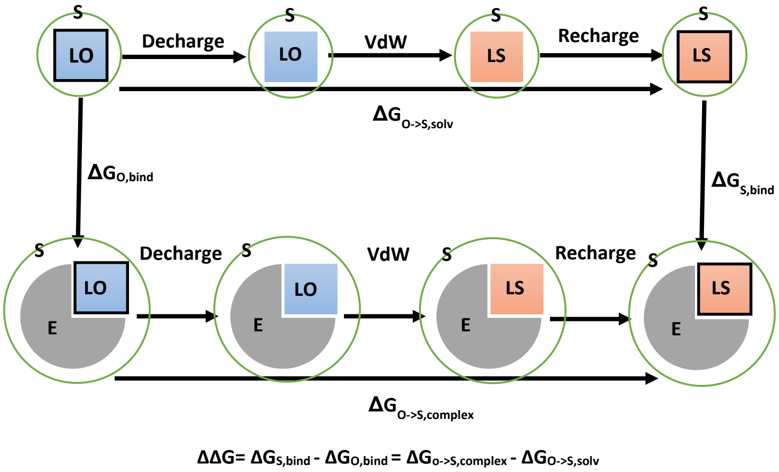

Table A1. *Results of thermodynamic integration mutating chloroacetamide to chlorothioacetamide ligand (**12** to **14**) in water and bound to BTK in water. All data are in kcal/mol.*

	**Cys481 thiol**				**Cys481 thiolate**			
	Calc 1	Calc 2	Calc 3	Mean	Calc 1	Calc 2	Calc 3	mean
ligand(O→S)	3.18	3.44	3.85		2.93	3.45	2.82	
complex(O→S)	3.97	4.63	3.65		3.83	3.56	4.53	
ΔΔG(O→S)	0.79	1.19	-0.19	0.60	0.90	0.11	1.71	0.75

d) In methods section, the manuscript states: “The QM region consisted of the active site cysteine residue cut between C and C and also the warheads of the ligands... The difference between the S–C and C–Cl bond distances was used as a reaction coordinate.” The reaction coordinate should be clearly illustrated in a figure. Without this, the description is difficult to follow. The authors must also provide details regarding the number of windows, sampling time per window, and QM/MM partitioning scheme to ensure reproducibility.

The separation of the system into QM and MM regions is shown in Figure A2. The reaction coordinate is presented in Figure A3. All details of the system preparation and PMF calculations are now given, including the number of windows and the sampling time per window.

“*Structure preparations for QM/MM calculations*

The covalent enzyme-inhibitor complexes were generated by mutating the molecule covalently bound to the active site cysteine in the PDB: 5P9J structure. The covalent bond between the sulfur and carbon atom was broken and the ligand warhead was modified. The resulted enzyme-ligand complexes were immersed in an octahedral water box of TIP3P waters[87] and neutralized by adding sodium ions. The QM region consisted of the active site cysteine residue cut between C_α and C_β and also the warheads of the ligands. All the other atoms were part of the MM region (Figure SX2). The dangling bonds of the QM region were filled with hydrogens (link atom approach). The systems were subjected to the relaxation protocol with 1000 steps of steepest descent minimization, 20 ps NVT heating to 300 K and 2 ns of NPT equilibration. The SHAKE algorithm was used to constrain hydrogens. The difference between the C-Cl and S-C bond distances was used as a reaction coordinate (Figure A3).

PMF calculations with QM/MM MD simulations

Simulation of the covalent reaction step was performed by molecular dynamics combined with umbrella sampling[88] (US) using the AMBERtools24 software package (Amber 2024, University of California, San Francisco, CA, USA). The QM part was treated with DFTB3 potential,[89] while FF14SB[90] and GAFF[91] force fields were applied for the MM region. Starting geometries for the simulation windows were generated by steered molecular dynamics simulations[92] performed in NVT ensemble at 300 K. The middle of the biasing potential was pulled along the reaction coordinate between -2.0 and 0.7 Å by the velocity of 1 Å/ps and a force constant of 250 kcal/(mol·$Å^2$). Frames with 0.1 ps intervals were extracted (see Supplementary Data 1) and used as starting structures for the US simulation windows. QM/MM MD US simulations were performed in NVT ensemble at 300 K with a 250 kcal/(mol·$Å^2$) force constant applied for the reaction coordinate. 30 ps simulation with 1fs time step was performed for each of the 28 windows and the last 20 ps was used in the generation of the PMF by the weighted histogram analysis method (WHAM).[93] The statistical uncertainties were calculated by WHAM’s bootstrapping algorithm using ten fake data sets coupled with a 140-step correlation time derived from the autocorrelation function of several data files of the Umbrella Sampling simulations. Approximate transition states were identified as maxima on the free energy curves. PMF profiles are shown in Figure A4.”

Figure A2. *Separation of the QM and MM systems. QM atoms are shown in red and MM atoms in black. All other atoms (protein and water) not shown are part of the MM system.*

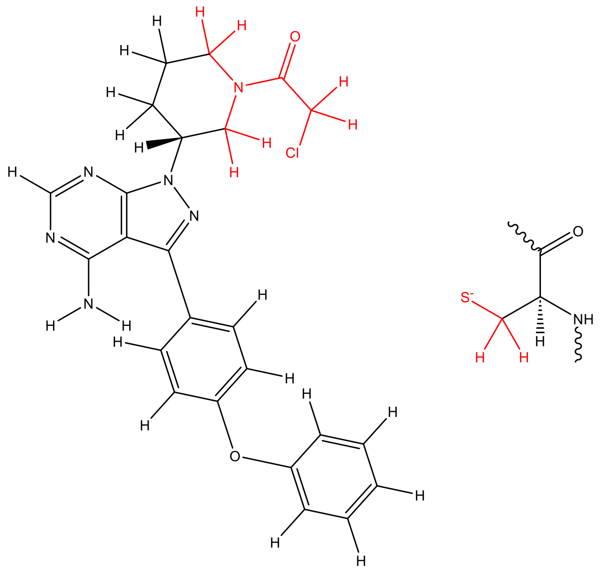

Figure A3. *Reaction coordinate is defined as the difference between the broken (C-Cl) and forming (S-C) bond lengths.*

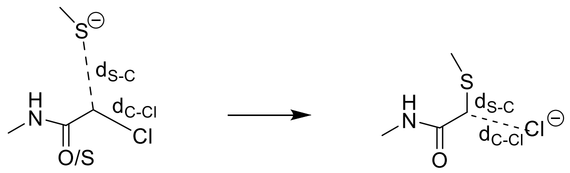

reaction coordinate: d_{C-Cl} - d_{S-C}

Figure A4. *Free energy profiles for the reactions of compounds* ***12*** *and* ***14*** *with BTK.*

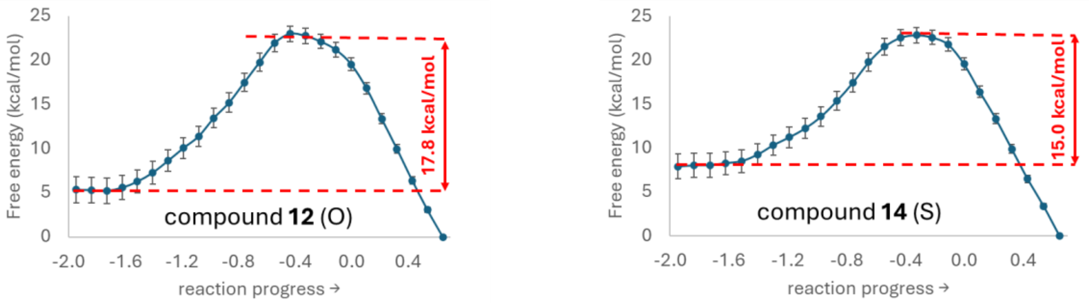

e) The computational results are poorly described and insufficiently presented. The authors should: Provide a table summarizing the relative free energies obtained from the TI calculations. Include the PMF profiles and tabulated activation free energies. Supply the starting structures used in the simulations as Supporting Information (SI) to allow reproducibility.

The relative free energies obtained in each perturbation is given in Table A1 (see our response to comment c). The PMF profiles are presented in Figure A4 (see our response to comment d) that also shows the numerical values of the free energy barriers (see our response to comment d). The starting structure of each window (28-28 structures for complexes with **12** (O) and **14** (S)) is presented as Source Data).

Reviewer #2

The authors have revised their manuscript and most of this reviewer's points have been addressed. However, some issues still require further refinement or clarification, and a few new concerns have arisen following the revisions. In addition, one important point regarding the analytical data was overlooked in the initial assessment, necessitating another round of major revision. The authors should address the following aspects:

We thank the referee carefully reading the manuscript and suggesting points for further improvements. We believe these comments helped us to complete this work.

Major:

New: Several compounds exhibit significant contamination in the NMR data and/or poorly resolved spectra. The following NMR spectra need improvement:

- ^1H NMR: Compounds 5a, 6b, 9, 14, 15, 24
- ^{13}C NMR: Compounds 5b, 6b, 13, 14, 15, 18, 21

Furthermore, the compounds should be depicted on the respective spectra to facilitate verification of chemical shifts.

We thank the reviewer for careful assessment. All NMR spectra listed have been thoroughly re-examined. We remeasured and/or reevaluated the spectra, refined all peak assignments, and ensured that each spectrum now includes a clear structural depiction of the corresponding compound for easier interpretation. The improved and fully curated data have been incorporated into the revised Supporting Information. We believe that the revised NMR characterization now fully meets the Nature Communications standards. The revised spectra and their assignments are shown in Figure A5

Figure A5. *Revised NMR spectrum and their assignments.*

^1H-NMR spectrum

***N-Phenyl-2-chlorothioacetamide* (5a)**

^1H NMR (500 MHz, $CDCl_3$) δ 9.71 (s, 1H), 7.78 (d, *J* = 7.9 Hz, 2H), 7.47 (t, *J* = 7.6 Hz, 2H), 7.35 (t, *J* = 7.3 Hz, 1H), 4.73 (s, 2H) ppm.

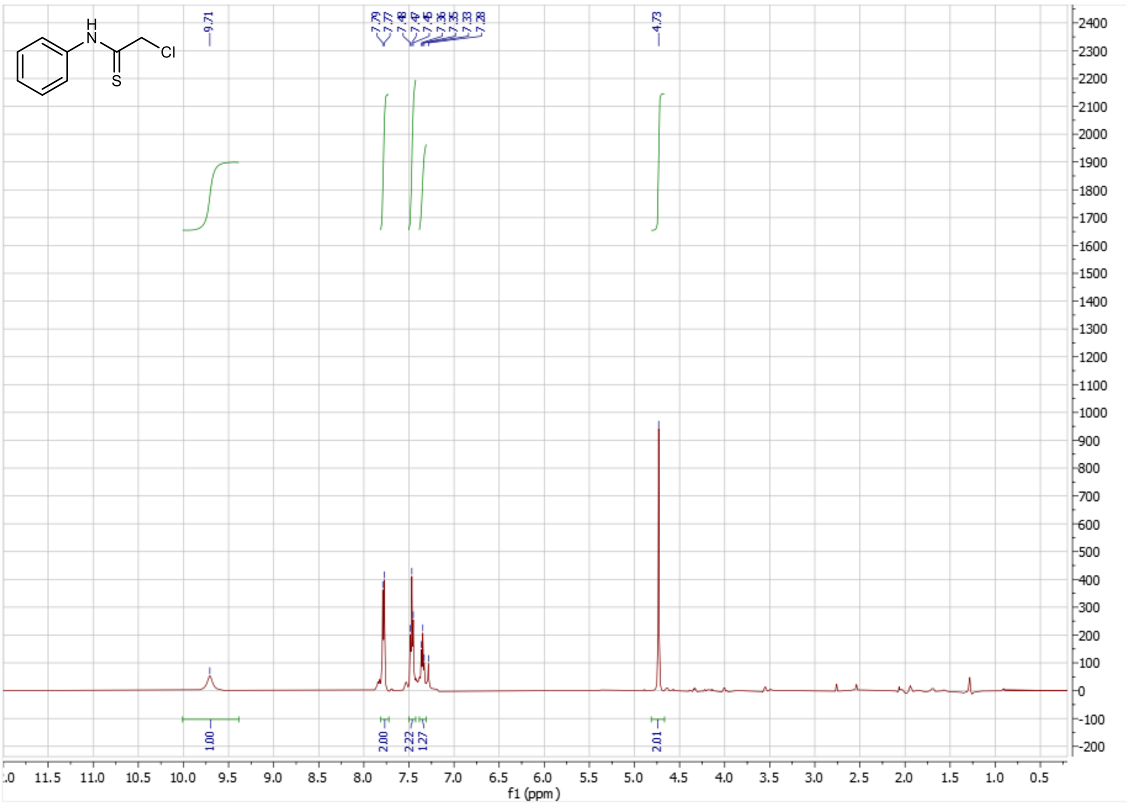
H
N
Cl
S
9.71
7.79
7.77
7.48
7.47
7.45
7.36
7.35
7.33
7.28
4.73
1.00
2.00
2.22
1.27
2.01
f1 (ppm)

N-(3,5-Bis(trifluoromethyl)phenyl)-2-fluorothioacetamide (6b)

^{1}H NMR (500 MHz, $CDCl_3$) δ 9.55 (s, 1H), 8.43 (s, 2H), 7.83 (s, 1H), 5.25 (d, *J* = 48.2 Hz, 2H) ppm.

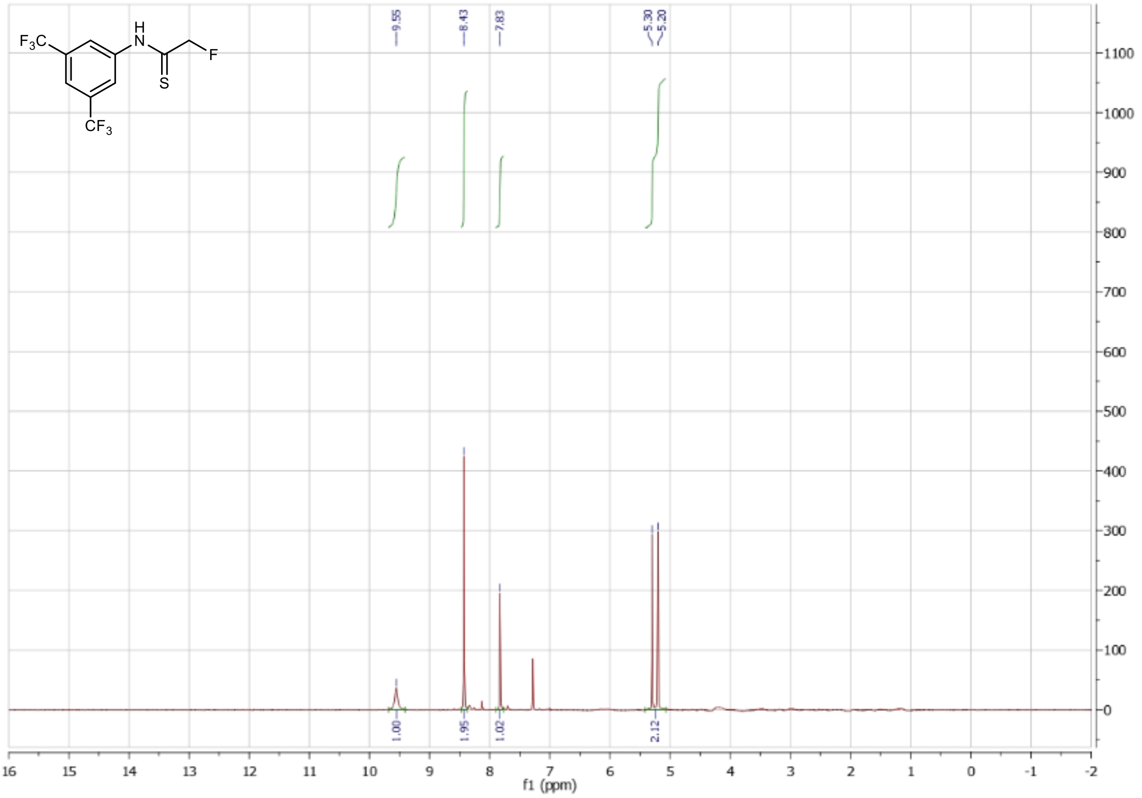

***N-(3-(7H-Pyrrolo[2,3-d]pyrimidin-4-yl)phenyl)-2-chloroethanethioamide* (9)**

^{1}H NMR (500 MHz, DMSO-d_6) δ 12.93 (s, 1H), 12.37 – 12.24 (m, 1H), 9.02 (s, 1H), 8.86 (s, 1H), 8.10 (t, *J* = 8.8 Hz, 2H), 7.90 (s, 1H), 7.72 (t, *J* = 7.9 Hz, 1H), 7.17 (s, 1H), 4.66 (s, 2H) ppm. In the ^{1}H NMR 2-methyltetrahydrofuran from the reaction is visible.

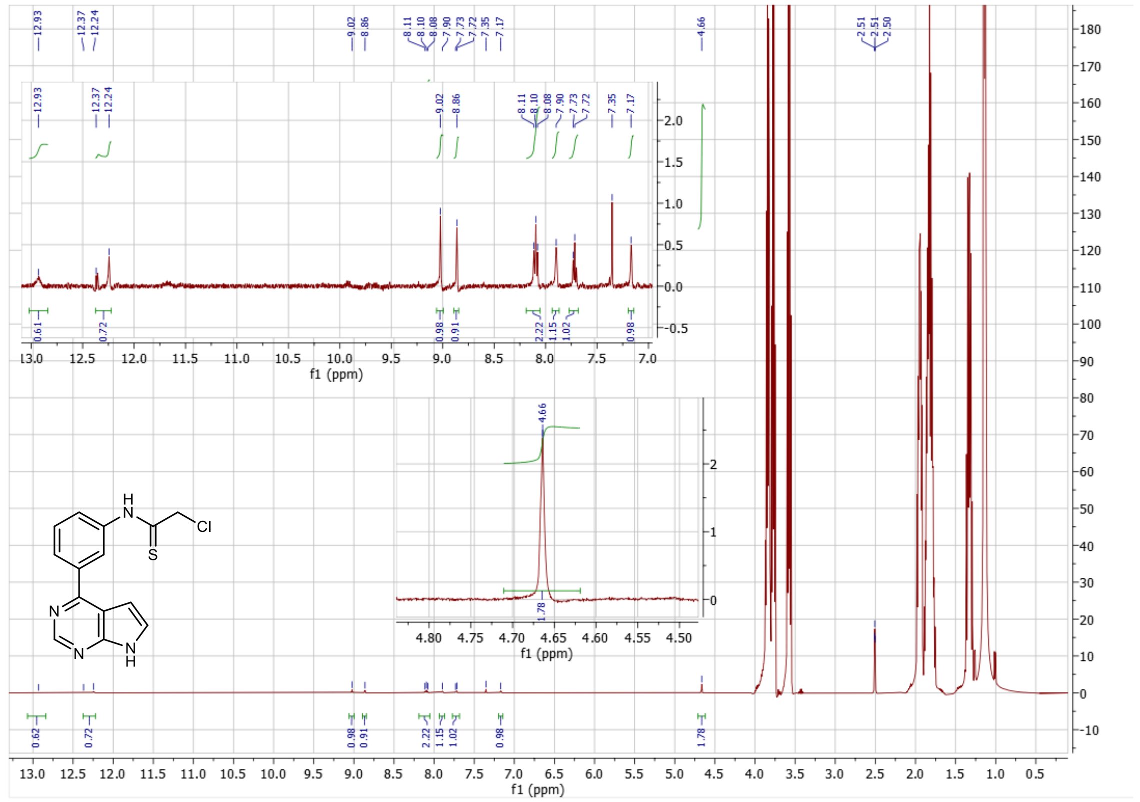

***1-(3-(4-Amino-3-(4-phenoxyphenyl)-1H-pyrazolo[3,4-d]pyrimidin-1-yl)piperidin-1-yl)-2-chloroethanethione* (14)**

^{1}H NMR (500 MHz, $CDCl_3$) δ 7.63 (bs, 2H), 7.43 (t, *J* = 7.8 Hz, 2H), 7.21 (q, *J* = 7.3 Hz, 5H), 7.13 (d, *J* = 8.2 Hz, 2H), 7.00 (s, 1H), 5.55 – 5.30 (m, 1H), 5.23 – 4.98 (m, 2H), 4.66 – 4.59 (m, 2H), 4.57 – 4.46 (m, 1H), 4.05 – 3.96 (m, 2H), 3.96 – 3.89 (m, 2H), 3.79 – 3.70 (m, 2H) ppm.

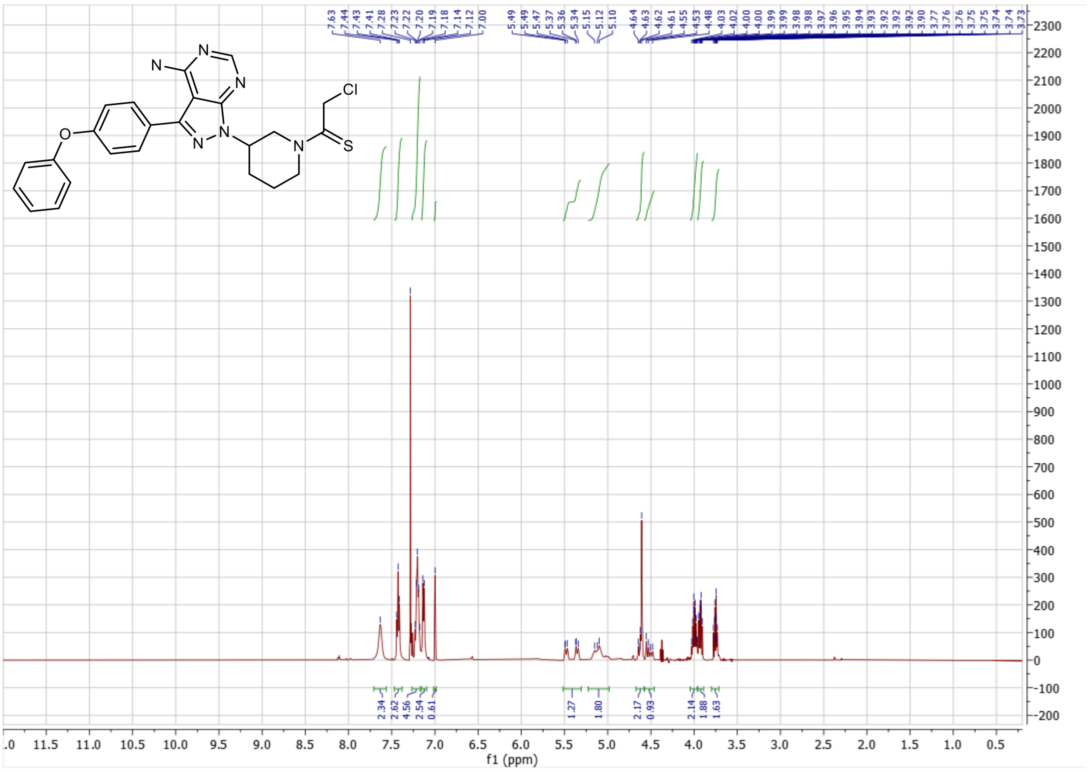

***1-(3-(4-Amino-3-(4-phenoxyphenyl)-1H-pyrazolo[3,4-d]pyrimidin-1-yl)piperidin-1-yl)-2-fluoroethanethione* (15)**

^{1}H NMR (500 MHz, $CDCl_3$) δ 8.35 (d, *J* = 14.8 Hz, 1H), 7.62 (d, *J* = 8.1 Hz, 2H), 7.44 (t, *J* = 7.7 Hz, 2H), 7.26 – 7.04 (m, 7H), 5.16 – 4.88 (m, 3H), 4.79 – 4.42 (m, 1H), 4.06 – 3.71 (m, 2H), 3.54 – 3.17 (m, 1H), 2.53 – 2.21 (m, 3H), 2.17 – 2.00 (m, 1H), 1.88 – 1.70 (m, 1H) ppm.

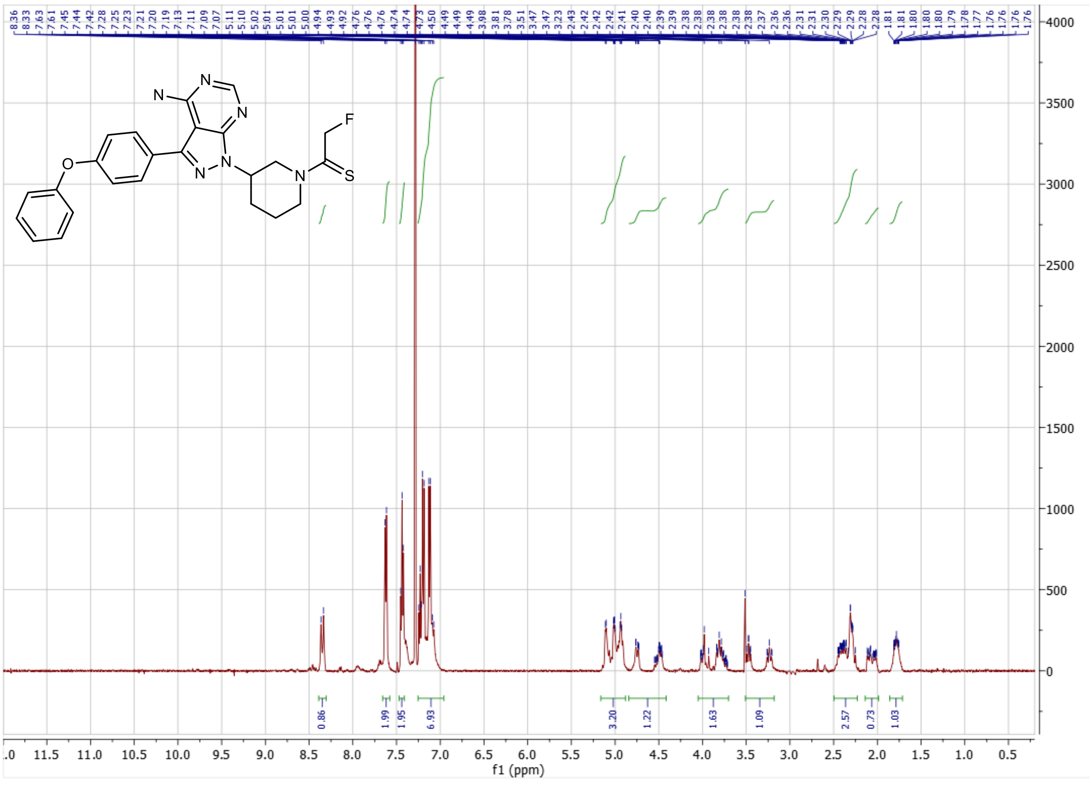

2-(6-(Dimethylamino)-3-(dimethyliminio)-3H-xanthen-9-yl)-5-(((5-((3R,5R)-7-(2-(4-fluorophenyl)-5-isopropyl-3-phenyl-4-(phenylcarbamoyl)-1H-pyrrol-1-yl)-3,5 (24)

^{1}H NMR (500 MHz, $CDCl_3$) δ 8.51 (s, 1H), 8.17 (d, *J* = 7.8 Hz, 1H), 7.54 (s, 1H), 7.24 – 7.06 (m, 11H), 7.03 – 6.91 (m, 4H), 6.86 (d, *J* = 8.9 Hz, 2H), 6.61 – 6.51 (m, 3H), 4.13 – 3.99 (m, 2H), 3.93 – 3.82 (m, 1H), 3.75 – 3.67 (m, 1H), 3.56 – 3.47 (m, 2H), 3.45 – 3.40 (m, 2H), 3.35 – 3.23 (m, 1H), 3.21 – 3.14 (m, 2H), 3.14 – 2.98 (m, 10H), 2.36 – 2.19 (m, 2H), 1.75 – 1.57 (m, 4H), 1.55 – 1.38 (m, 10H), 1.38 – 1.20 (m, 4H).

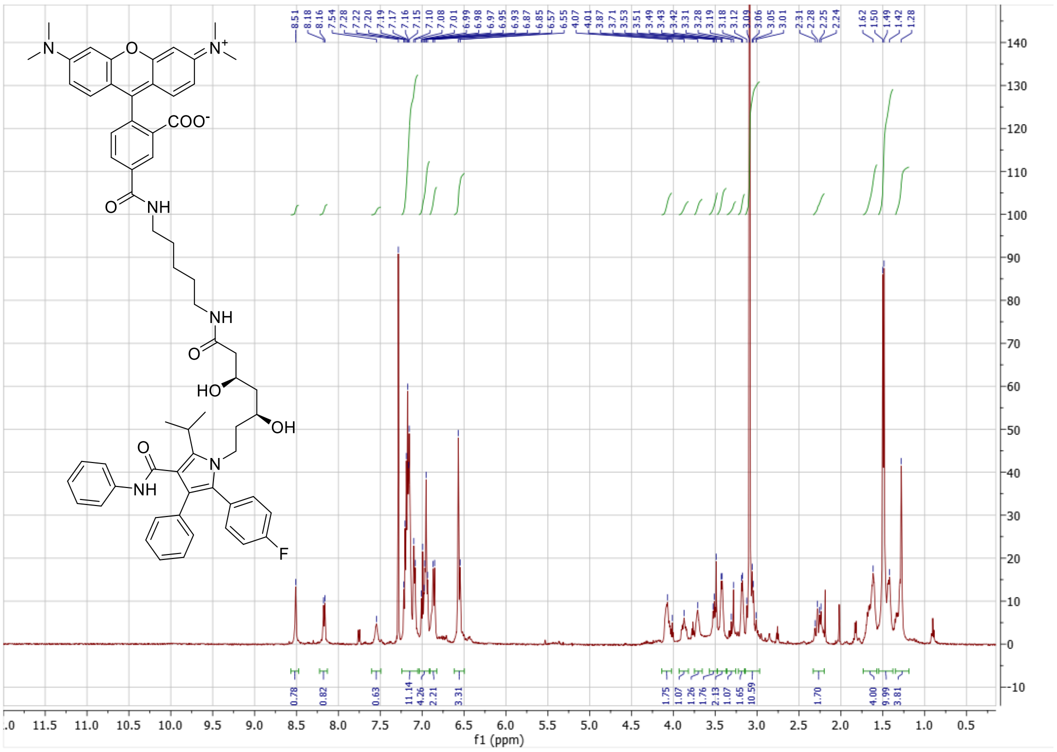

^{13}C-NMR spectrum

N-Phenyl-2-fluorothioacetamide (5b)

^{13}C NMR (125 MHz, $CDCl_3$) δ 187.9, 133.9, 127.8, 127.5, 118.9, 118.4, 118.4, 115.8, 45.8 ppm.

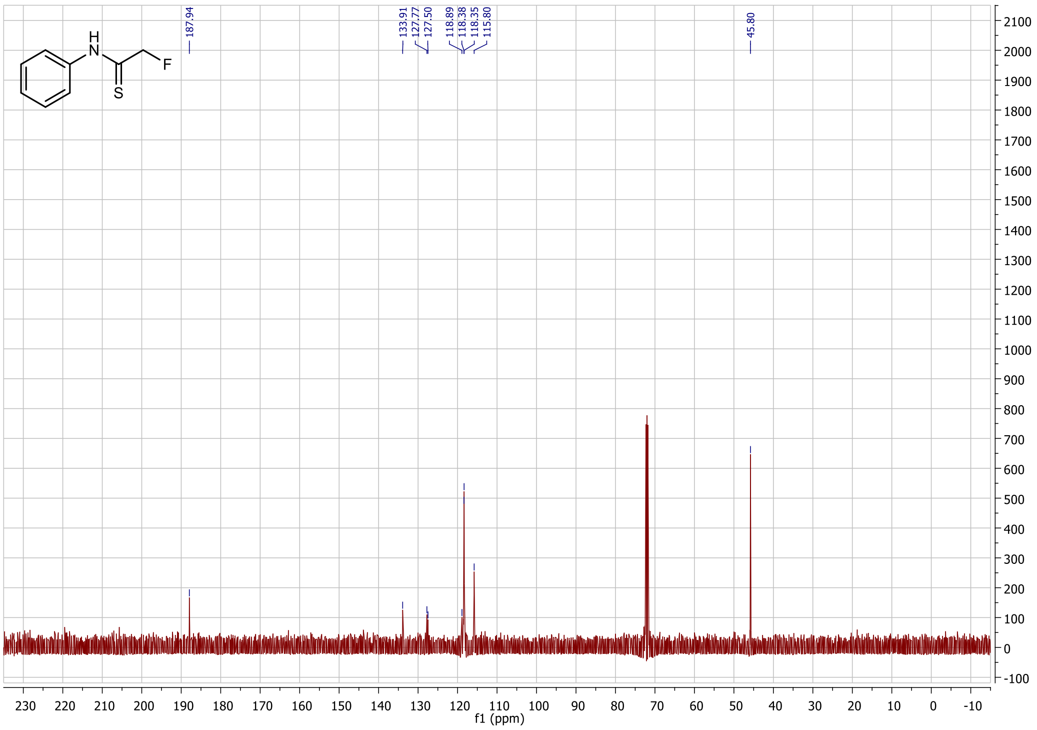

***N*-(3,5-Bis(trifluoromethyl)phenyl)-2-fluorothioacetamide (6b)**

^{13}C NMR (125 MHz, $CDCl_3$) δ 194.9, 138.4, 132.7, 132.4, 123.9, 123.1, 123.0, 121.7, 120.6, 85.8 (d, *J* = 197.4 Hz) ppm.

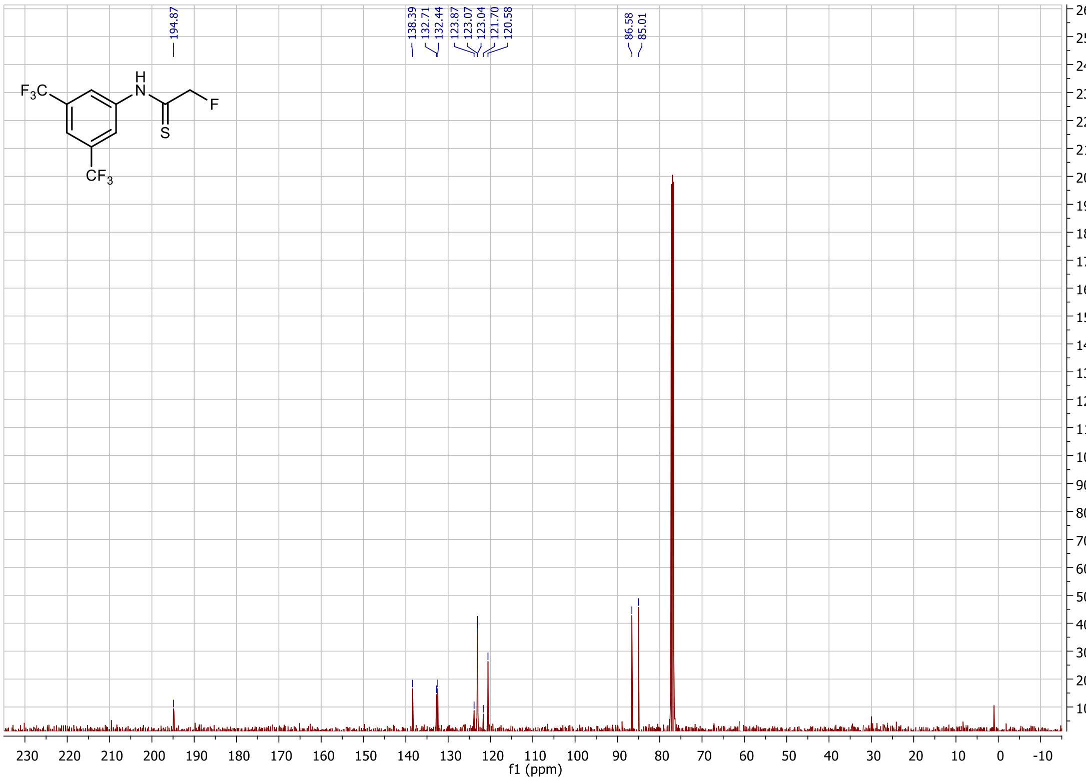

***1-(3-(4-Amino-3-(4-phenoxyphenyl)-1H-pyrazolo[3,4-d]pyrimidin-1-yl)piperidin-1-yl)-2-fluoroethanone* (13)**

^{13}C NMR (125 MHz, $CDCl_3$) δ 189.6, 165.6, 157.9, 155.9, 155.8, 130.0, 127.8, 127.5, 124.1, 122.0, 119.6, 119.1, 110.0, 80.8, 79.3, 53.2, 48.8, 45.5, 42.3, 30.2, 29.8, 23.7 ppm.

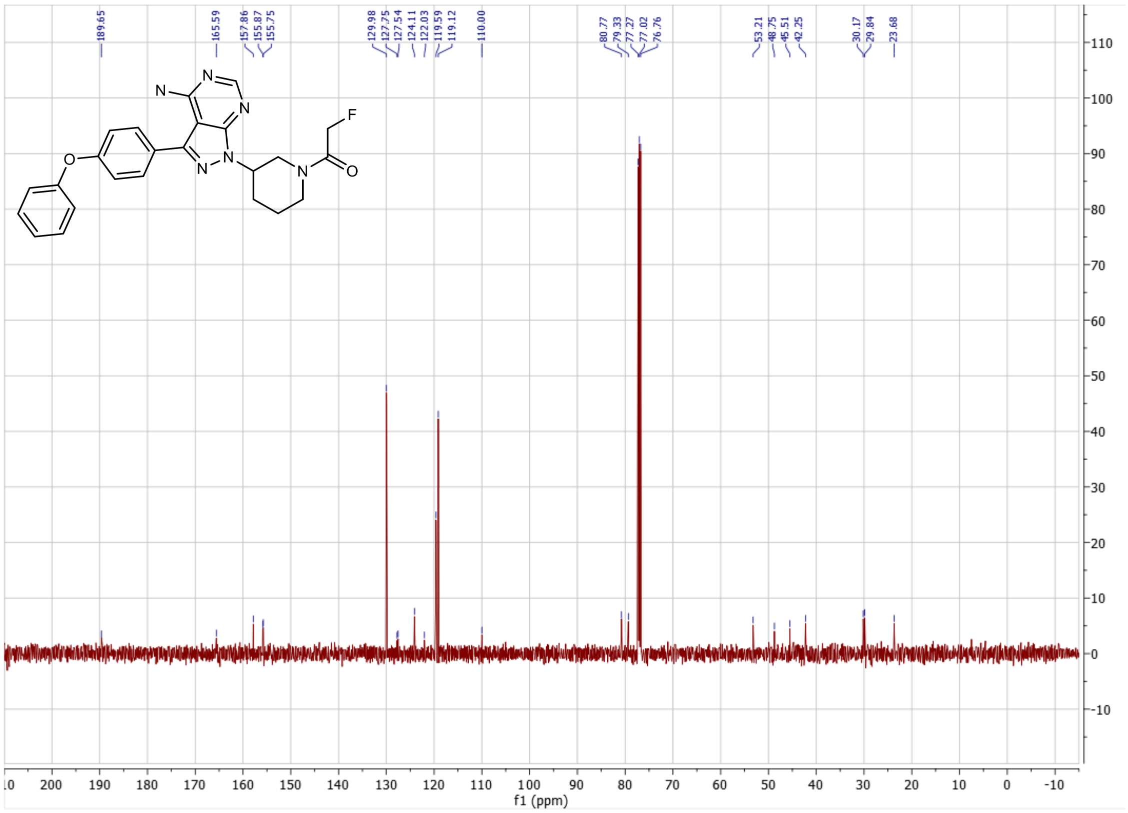

***1-(3-(4-Amino-3-(4-phenoxyphenyl)-1H-pyrazolo[3,4-d]pyrimidin-1-yl)piperidin-1-yl)-2-chloroethanethione* (14)**

^{13}C NMR (125 MHz, $CDCl_3$) δ 155.8, 151.6, 147.5, 138.4, 138.0, 135.9, 130.2, 129.9, 129.1, 128.3, 125.6, 125.4, 124.6, 120.1, 120.0, 119.5, 67.8, 33.1, 30.4, 29.8, 25.9, 21.0 ppm.

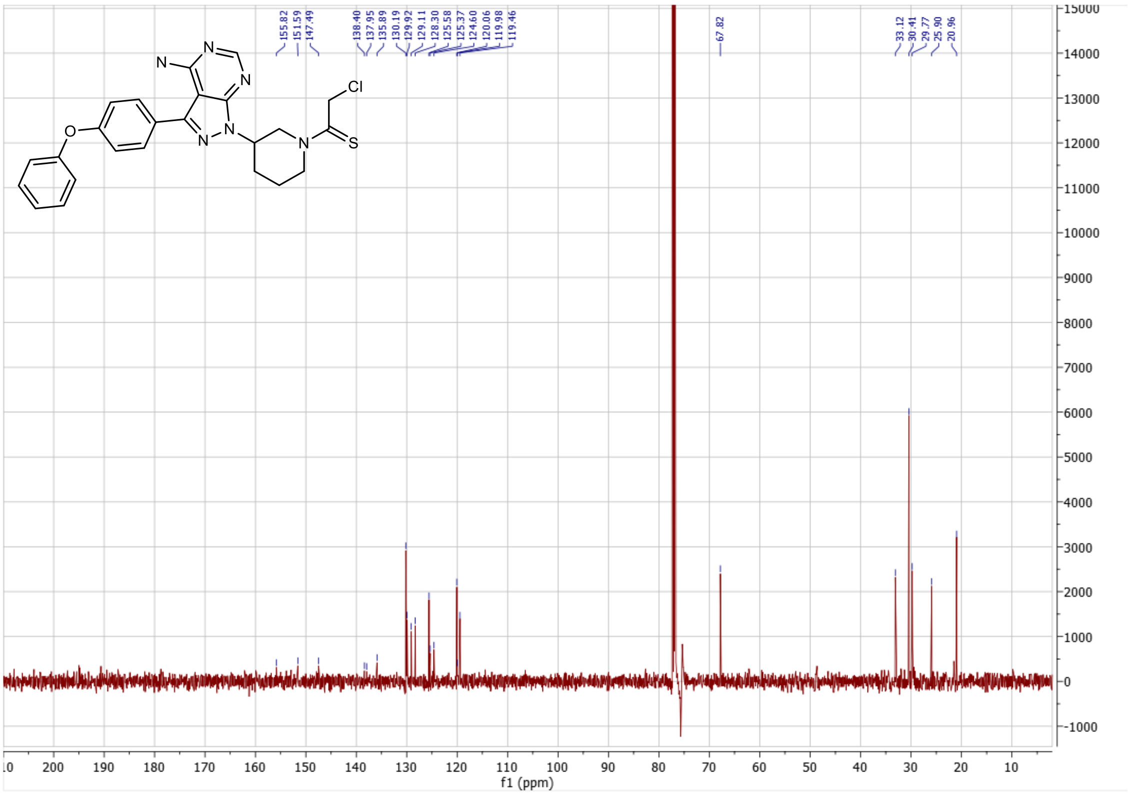

***1-(3-(4-Amino-3-(4-phenoxyphenyl)-1H-pyrazolo[3,4-d]pyrimidin-1-yl)piperidin-1-yl)-2-fluoroethanethione* (15)**

^{13}C NMR (125 MHz, $CDCl_3$) δ 160.0, 153.8, 153.6, 147.0, 146.4, 145.6, 130.2, 129.7, 125.0, 124.7, 124.7, 120.0, 119.2, 103.4, 100.3, 87.0, 85.5, 54.2, 53.6, 52.8, 50.9, 50.6, 29.8, 24.8 ppm.

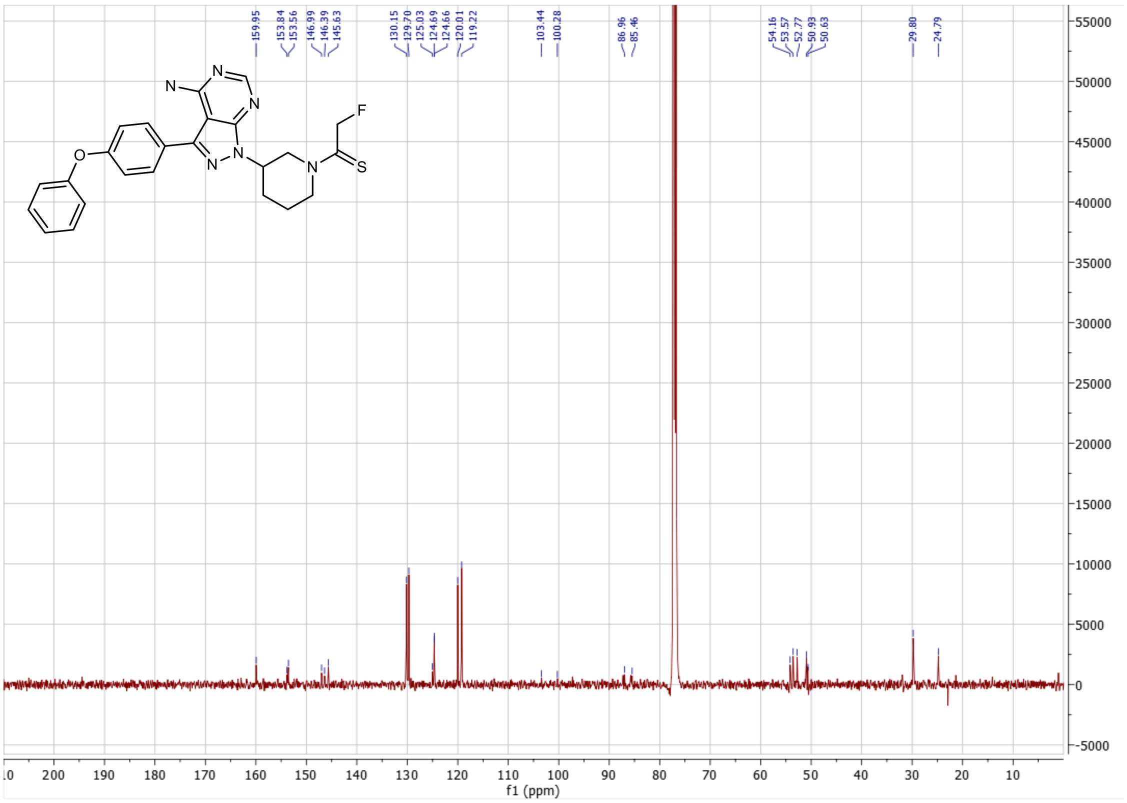

***1-(3-(4-Amino-3-(4-(3-ethynylphenoxy)phenyl)-1H-pyrazolo[3,4-d]pyrimidin-1-yl)piperidin-1-yl)-2-chloroethanethione* (18)**

^{13}C NMR (125 MHz, $CDCl_3$) δ 209.3, 159.1, 156.0, 130.1, 130.1, 130.0, 130.0, 127.8, 127.8, 122.7, 122.7, 120.2, 120.1, 119.5, 54.4, 53.8, 53.3, 52.0, 50.9, 50.7, 48.8, 48.7, 29.7, 29.7, 29.5, 24.7, 23.0 ppm.

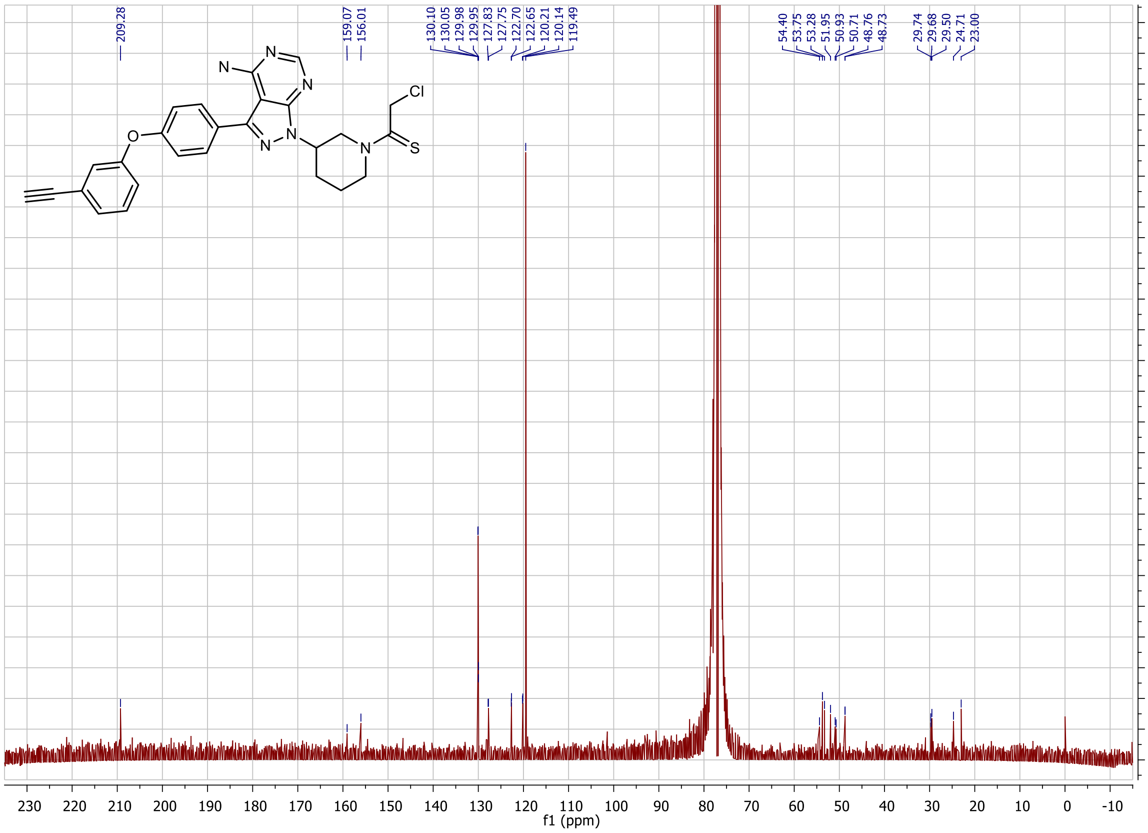

***2-Chloro-N-(4-(prop-2-yn-1-yloxy)phenyl)ethanethioamide* (21)**

^{13}C NMR (125 MHz, $CDCl_3$) δ 191.2, 156.5, 131.4, 125.0, 115.3, 78.2, 75.9, 56.1, 50.5 ppm.

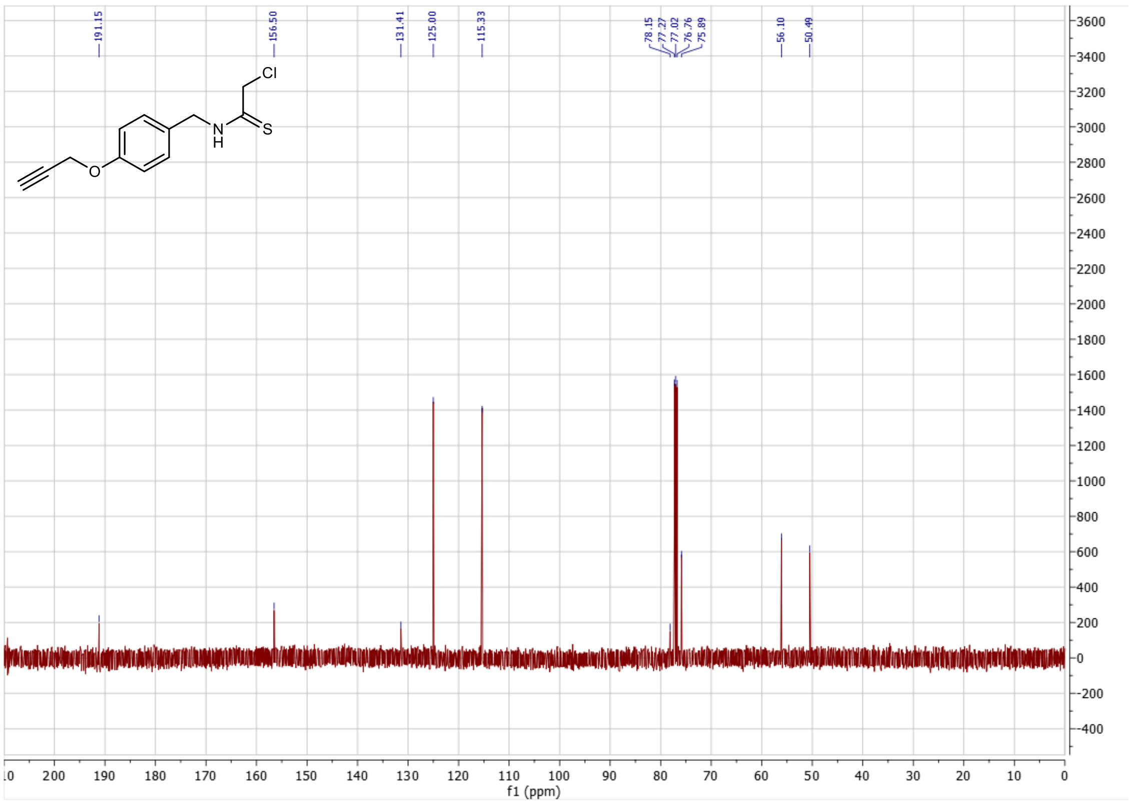

Initial request: The initial reactivity study does not include any alkyl amine-derived thioamide electrophiles, so it is unclear how the aniline-derived electrophiles from the first part of the study compare to the piperidine-based ones used in the second part. These two compound classes should be ranked against each other. I strongly suggest synthesizing benzylamine and piperidine-derived sets of halothioamide/thioacrylamide fragments and testing these in the GSH assay for comparison. It would also be important to show if the decreased reactivity commonly observed for N-alkyl acrylamide versus N-aryl acrylamide transfers to thioacrylamide. Author response: Accepting the comment of the referee we synthesized the following compounds: N-benzyl-chloroacteamide, N-benzyl-chlorothioacetamide, N-benzyl-fluorothioacetamide, N-piperidinyl chloroacetamide, N-piperidinyl chlorothioacetamide, N-piperidinyl fluorothioacetamide. These compounds were evaluated in the kinetic GSH assay as well as in the oligopeptide-based selectivity assay, and the results are now discussed in the main text. In brief, the new data do not alter the overall conclusions but provide significant confirmation of the O/S exchange effect originally reported in this study. As our attempts to prepare thioacrylamides were unsuccessful in most cases, we finally decided to remove all thioacrylic and acrylic probes from the study and instead focus exclusively on the haloacetamide and halothioacetamide series. The revised library now comprises 16 probes across four scaffolds (phenyl, bistrifluorophenyl, benzyl, and piperidyl), representing a robust and well-designed covalent fragment set.

Reviewer Response: I appreciate the synthesis and characterization of the new. However, the discussion should mention thioacrylamides, their synthesis attempts and the observed high reactivity and limited accessibility that ultimately led to their exclusion. Including this information would be highly valuable for other researchers, as it could prevent unnecessary repetition in future studies.

Accepting the comment of the referee, we have added a brief discussion to the main text addressing our attempts to synthesize thioacrylamides, the challenges and the reasons for their exclusion from further studies. Specifically, we now highlight that although these compounds could be obtained, their pronounced instability and hyperreactivity rendered them unsuitable for inclusion in our O-to-S exchange strategy. In addition, full experimental details related to the thioacrylamide attempts have been added to the Supplementary Information to ensure transparency and to support other researchers in avoiding unnecessary repetition. The related paragraph reads as follows:
"Noteworthy, in addition to haloacetamides, the method was also evaluated on acrylamide substrates. While thioacrylamides could in principle be formed, only phenylthioacrylamide proved sufficiently stable to be isolated in pure form. In contrast, three additional thioacrylamide derivatives could not be obtained due to rapid decomposition during workup and purification. This behavior is consistent with the generally high electrophilicity and hyperreactivity measured in GSH-assay of the only isolated phenylthioacrylamide. Owing to their limited stability and accessibility, acrylamide-derived thioamides were not pursued further in this study."

Initial request: The degree of intact protein labeling and stoichiometries need to be quantified and discussed for all tested probes and proteins (see comment above).

Author response: The extent of protein labeling has been quantified, and the results are now provided in the supplementary material. Since the MS measurement conditions were not standardized, we do not discuss these data in detail in the main text, as no robust conclusions can be drawn from the heterogeneous results obtained (see Supplementary Information)

Reviewer Response: Thank you for addressing this point. In addition, please include the labeling stoichiometries. Some discussion regarding compounds' degree of labeling would also be warranted.

Labeling stoichiometries have now been reported for all tested probes and proteins and added to the Supplementary Information alongside the previously provided quantification data. As requested, we have also expanded the main text with a brief discussion summarizing the observed labelling stoichiometries across the different probes.

Initial request: Considering the potential application of these thioamide-derived warheads in TCIs, and the known susceptibility of sulfur to oxidation, some investigation of metabolism, at least in vitro, would be a beneficial addition to the data presented.

Author response: We thank the reviewer for this valuable suggestion. To address it, we performed in vitro human liver microsome metabolism experiments and assessed the remaining compounds by HPLC measurements. In brief, both the chloroacetamide and chlorothioacetamide derivatives displayed a similar range of metabolic stability, with 1–4% of the compound remaining after 30 minutes. In contrast, the moderately reactive but proven covalent fluorothioacetamide derivative showed increased stability, with 10% remaining after 30 minutes.

Reviewer response: It seems this data has not been added. The authors should include the in vitro metabolism data and add a brief discussion of the observed properties.

The *in vitro* human liver microsome metabolism data have now been added to the Supplementary Information and shown in Figure A6. In addition, we have incorporated a short discussion into the main text summarizing the observed metabolic stability trends of the ibrutinib-derived covalent probes. In brief, both the chloroacetamide and chlorothioacetamide derivatives displayed a similar range of metabolic stability, with 1–4% of the compound remaining after 30 minutes. In contrast, the moderately reactive but proven covalent fluorothioacetamide derivative showed increased stability, with 10.2% remaining material after 30 minutes.

Figure A6. *In vitro metabolic stability of compounds 11-15 in human liver microsomes.*

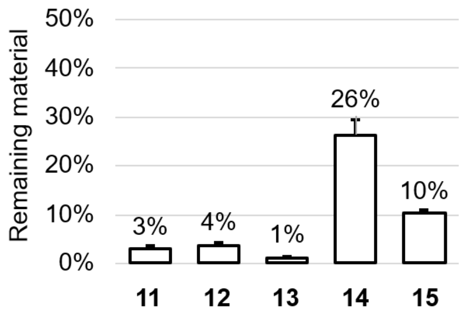

Minor:

Initial request: How did the authors unambiguously distinguish between the respective labeled amino acids in the peptide assay? Please specify in the body of the manuscript and provide data. The same applies to protein labeling.

Author Response: The identification of labeled amino acids in both peptide and protein levels was performed using standard LC–MS/MS analysis. In the case of protein labeling, proteins were digested with trypsin (and, where appropriate, chymotrypsin) to generate a series of peptides with defined cleavage sites, including a/b/c and x/y/z ion series. Labeled peptides were detected as precursor (mother) ions, and their fragmentation patterns were analyzed to determine the exact position of the modification. Differences in the m/z ratios of fragment ions between labeled and unlabeled peptides allowed unambiguous identification of the labeling site.
For nonapeptide labeling, the approach is technically equivalent, but no enzymatic digestion was required because the oligopeptide is already compatible with downstream MS analysis. The labeled peptide was directly detected, and the position of the modification was confirmed by the MS/MS fragmentation pattern.

To illustrate this, we prepared a representative figure (below), showing the labeled peptide with the fragmentation pattern annotated to highlight the a, b and y ions that differ from the unlabeled series at the modification site. Additionally, we have added the MS/MS spectra of all covalent probes reacted with the nonapeptide surrogate in the supplementary material. These spectra demonstrate the unambiguous assignment of the labeling sites. This approach is consistent with standard practices in peptide mapping and chemical proteomics studies.

Reviewer Response: Thank you for providing the new figure. It clearly addresses and clarifies this point. However, it appears that it has not been included in the manuscript. Please ensure that it is added, at least as part of the Supporting Information (SI).

The figure and the discussion provided in the first answer have been added to the Supplementary Information.

Initial request: If the piperidine-derived thioacrylamide proofs to be significantly less reactive than the above aniline derivatives, this electrophile should be included in the TCI part of the study.

Author response: We agree with the reviewer that the piperidine-derived thioacrylamide would represent an interesting electrophile to investigate. Therefore, we synthesized and tested a phenyl-thioacrylamide fragment (6a). This compound was hyper-reactive and labelled GSH promptly. Any further attempts to prepare thioacrylamides were unsuccessful due to the extremely high reactivity and the limited stability of these compounds. For this reason, as stated above, we finally decided to remove all thioacrylic and acrylic probes from the study and instead focus exclusively on the haloacetamide and halothioacetamide series. The revised library now comprises 16 probes across four scaffolds (phenyl, bistrifluorophenyl, benzyl, and piperidyl), representing a robust and well-designed covalent fragment set. In the revised manuscript, we discuss the presented thioamide-based warheads as a novel option for bioconjugation strategies, including TCI development, ADC design, and target identification. We believe that the chloro- and fluorothioacetamides introduced herein represent valuable new tools for the medicinal chemistry and chemical biology communities, opening avenues for next-generation covalent drug discovery.

Reviewer response: see major comment on discussion of thioacrylamides above

As noted in our response to the major comment above on thioacrylamides, we have added a brief discussion to the main text explaining that thioacrylamide derivatives were indeed synthesized and evaluated but ultimately excluded from further studies due to their extreme reactivity and limited stability.

Initial request: TCI part, kinase inhibition profiling: For rigorous characterization of covalent inhibitors, especially if different warheads/electrophiles are to be compared, the contributions of non-covalent and covalent binding need to be deconvoluted (i.e., KI and kinact). Kinetic assays are now readily available for BTK, and such data must be complemented accordingly. The fact that probe 14 does not seem to modify the enzyme at all but still has a relatively potent IC50 of 45 nM should also be discussed. It seems that the covalent mode of action does not contribute much to the observed activity, in line with the slow labeling kinetics shown in the supplementary information. Interestingly, probe 13 shows very slow labeling kinetics in MS but higher potency than 12 and Ibrutinib, which have much faster labeling kinetics. This suggests that the observed boost in activity is due to non-covalent interactions. This further supports the critical need for kinact and KI determination for these derivatives and a more elaborate discussion of the results.

Author response: We thank the reviewer for this insightful comment. To address it, we deconvoluted the contributions of non-covalent and covalent binding to BTK by determining KI and kinact values in kinetic assays for the ibrutinib derivatives (compounds 11–15). The results confirmed the significant role of non-covalent interactions, in line with the trends anticipated from our preliminary reactivity experiments. These findings are now discussed in detail in the main text, and the corresponding data are reported in the Supplementary Material.

Reviewer response: The addition of this data is appreciated. However, it is recommended providing kinact values in s-1 (including for the kinact/KI ratio), as this format is more commonly used in the literature and facilitates comparison across studies. Additionally, please follow the style guidelines outlined in https://doi.org/10.1021/acs.jmedchem.4c01721 for kinact and KI in both the text and figures.

Accepting the comment of the referee, the reporting format for k_{inact}, K_I and k_{inact}/K_I has now been updated throughout the manuscript to follow the suggested style guidelines.

Initial request: title/manuscript: "Thioamide warhead" should be rephrased to "thioamide-derived warhead" or similar as thioamide itself is not the warhead functional group.

Author response: The title has been changed to “Thioamide-derived warheads with enhanced cysteine reactivity and specificity for covalent protein labelling”

Reviewer response: The changes are ok, but they should be implemented consistently throughout the manuscript. Since acrylamides have been removed from the scope, a more restricted and precise title is recommended, such as “α-Halo thioamide…” to accurately reflect the content.

The manuscript title has been updated to “α-Halothioamide warheads with enhanced cysteine reactivity and specificity for covalent protein labeling” to more precisely reflect the scope of the study.

Initial request: Page 2: "were mostly avoided from drug discovery pipelines due to potential idiosyncratic toxicity issues" could also mention that lack of selectivity was often a factor associated with covalent drug modalities.

Author response: The suggested text was added.

Reviewer response: Idiosyncratic reactions are not necessarily a consequence of unselectivity, and conversely, unselectivity can lead to issues other than idiosyncrasy. I suggest using more nuanced language here, especially since this is the opening sentence of the paper.

The sentence has been reworded to use more nuanced language, clarifying that both potential idiosyncratic toxicity and lack of selectivity have historically limited the development of covalent drug modalities, without implying a direct causal relationship between these two. Now it reads as follows: "*Electrophilic compounds that form a covalent bond with their targets, were historically avoided from drug discovery pipelines due to concerns about potential idiosyncratic toxicity and other pharmacological challenges related to insufficient selectivity.*"

Initial request: In the introduction, the authors may discuss the reaction types more clearly to provide context. Notably, the C=S bond is not directly involved in the displacement of alpha leaving groups. Although the reasoning sounds logical, the impact of the C=S bond on the softness of the beta-position in thioacrylamide electrophiles should be discussed with suitable citations or backed by own data.

Author response: We have clarified in the text that the C=S bond is not directly involved in the displacement of α-leaving groups, but it does alter the electronic environment of the adjacent β-position. To support this, we performed DFT calculations (M062X/6-311++G(d,p)) and analyzed the NBO- charge distribution localized to the adjacent carbon atom in β-position. These results consistently showed a positive shift of charge on the carbon adjacent to the thiocarbonyl group compared to the corresponding carbonyl analogue, indicating its increased electrophilic character. This supports our conclusion that thioamides act as more potent electrophiles due to these intrinsic electronic effects.

Reviewer response: Please make sure the data is mentioned in the manuscript

These data are now briefly discussed in the main text, while the corresponding data are presented in the Supplementary Information.

Initial request: Table 1 and discussion: Conditions (pH, solvent, temperature, concentrations) should be mentioned. Notably, it seems phenylacrylamide, a common standard, behaves relatively unreactively here (GSH t1/2 is often cited in the range of ~1-3h). It should also be mentioned if the depicted values are corrected for degradation or not.

Author response: We thank the reviewer for pointing this out. We have now added the experimental conditions (pH, solvent, temperature, and concentrations) to the caption of Table 1. Regarding the reactivity of phenylacrylamide, we note that its relatively less reactivity in our experiments likely reflects the specific buffer system, lower incubation temperature (25°C vs. 37°C) and concentration settings we used, which can differ from conditions in previously cited studies reporting shorter half-lives. Furthermore, we systematically applied stability correction for background degradation of the electrophiles,

which can substantially affect the apparent reactivity. These corrections provide a more reliable assessment of intrinsic thiol reactivity. We have clarified this in the revised text.

Reviewer response: The reference to aqueous stability correction may be misleading in the context of phenylacrylamides, as Table S2 indicates that the compound shows no significant degradation in buffer (t1/2 > 72 h). Rephrasing suggested.

We would like to clarify that phenylacrylamides are no longer discussed in the revised manuscript and has been removed from both Table 1 and Table S2, as acrylic and thioacrylic probes were excluded in a previous round of revision. Accordingly, the statement referenced in the reviewer comment was originally intended to address Reviewer #2's earlier concern but is no longer applicable to the current version of the manuscript.

Nevertheless, we note that for compounds such as phenylacrylamide or the α-fluoroacetamides (**4a**, **4c**, and **4d**), which exhibit high aqueous stability ($t_{1/2}$ > 72 h), the aqueous stability correction has minimal impact on the intrinsic thiol reactivity compared to the raw (apparent) parameters obtained from GSH-containing samples. While the same correction methodology is generally applied across the dataset, it results in essentially no difference between the apparent and the intrinsic thiol-reactivity for these highly stable compounds.

Initial request: Page 3: "Our results confirmed that O-S exchange increased the thiol-reactivity," is an overgeneralization. While the GSH data support this statement for AA/TAA, differences are fairly minor for the CA/CTA and FA/FTA matched pairs; CTA with R=H has essentially the same stability as its CA analog. Please refine this statement.

Author response: We refined the wording as follows: "Our results confirmed that O–S exchange positively contributed to the thiol reactivity of the covalent probes investigated herein." By extending the covalent fragment-level investigations with eight additional probes, we obtained solid support for the conclusion that the increase in thiol reactivity upon O–S exchange generally holds true. However, we acknowledge that this is not an exclusive rule, but rather a useful option for structure optimization.

Reviewer response: It seems the wording has been changed again after writing the rebuttal. This is should be avoided.

We thank the reviewer for pointing this out. To ensure consistency, the text has now been revised in the manuscript and reads as follow: "… *O–S exchange positively contributed to the thiol reactivity*".

Initial request: Page 5, KGDYHFPIC peptide: Notably, Cys and Lys are terminal in this peptide which may impact their reactivity. Could the authors explain why this peptide was chosen and mention the possible impact of the terminal positioning?

Author response: Although we agree that the terminal position of Cys and Lys might impact their reactivity, we used the same peptide in multiple papers (https://doi.org/10.1016/j.ejmech.2018.10.010; https://doi.org/10.1016/j.ejmech.2020.112836; https://doi.org/10.1002/cbic.202000700; https://doi.org/10.1016/j.ejmech.2022.114163; https://doi.org/10.3390/molecules28073042) and it was generally found to be useful for the first assessment of labelling specificity.

Reviewer response: The authors argumentation is ok but it is recommended to mention the potential impact of terminal localization briefly.

The possible impact of terminal Cys and Lys localization has been added to the discussion, including also the argumentation why we consider this methodology still a reliable experiment to assess target specificity.

Initial request: Page 6: "To evaluate their kinase targeting potential we have chosen six, covalently druggable, kinases representing each major kinase branch," the authors may comment on the positioning of the cysteines in the kinases and how this impacts their accessibility.

Author response: The target kinases were selected as therapeutically significant and already covalently targeted members of the protein kinase family. Taking into account the availability of covalently targetable cysteine residues (https://doi.org/10.7717/peerj.126), and based on the availability of LCMS-measurements of those targets, finally 3 kinases were selected: JAK3 and BTK from the TK (tyrosine kinase) branch, and MAP2K6 from the STE (yeast sterile 7-,11- and 20-homologue kinases) branch. Positioning of the targeted cysteines and characterization of their reactivity and accessibility based on Cy-preds server predictions (https://doi.org/10.1002/prot.24978; http://bioinformatics.akdeniz.edu.tr/cypreds/start.py) are summerized below. Also, we added some further information to the main text to discuss the covalent tractability of the cysteines in those 3 included kinases. Interestingly, those investigated kinases characterized by similar accessibility, yet significantly different reactivity on their targeted cysteine residues.

Reviewer response: Cys pKa prediction is prone to significant error and this limitation should be briefly acknowledged in the manuscript. Additionally, it would be helpful to mention in the text that the cysteine residues for BTK and JAK3 are located in the same position within the front pocket, whereas MKK6 has its cysteine in the DFG region.

We thank the reviewer for this important suggestion. We have now added a brief statement to the manuscript acknowledging that cysteine pKa and reactivity predictions are subject to significant uncertainty and should be interpreted as qualitative rather than quantitative descriptors. Additionally, we have clarified that the reactive cysteine residues in BTK and JAK3 are located at the same position within the front pocket, whereas the cysteine in MAP2K6 is located in the DFG region.

Additional minor points:

Page 2: “over a hundred functional groups available for covalent labeling.” I recommend avoiding explicit numbers here, as these can vary depending on what is considered a distinct functional group. Instead, using a more general phrasing such as “many” or “a significant number of” is recommended.

Wording has been corrected.

Page 2: "thioamides are expected to be softer electrophiles compared to their carbonyl counterparts, implying higher cysteine specificity." I am not convinced that this statement remains valid now that the Michael acceptor compounds have been excluded (see discussion regarding the involvement of the C=S bond). Rephrasing is recommended.

Softening the electrophilic center upon replacing oxygen with sulfur is a known consequence of the HSAB theory. This O-to-S substitution can be reasonably anticipated to modify the reactivity of the α-carbon, potentially improving its behavior in cysteine-targeting chemical biology studies or other covalent drug discovery applications. Using "expected" emphasizes that this is a prediction based on the HSAB theory, and our work is designed to evaluate how this translates in practice. Nevertheless, to avoid overclaiming expectations about selectivity or specificity of the thiocarbonyl probes, we have partially rephrased the statement. The revised sentence now reads: *"The C_α atoms of halothioamides are expected to possess softer electrophilic character than the corresponding carbons in carbonyl-based probes, which could result in favorable electronic properties for cysteine-targeting applications."*

Page 3: "its electronic effect strongly influences the adjacent β-position" Since Michael-type electrophiles are now excluded, this consideration should only apply to the α-position. Please check.

Corrected according to the reviewer's comment.

Page 3: "electrophilicity, providing a straightforward explanation for the higher reactivity observed with thioacrylamide electrophiles." Thioacrylamides are no longer included in the manuscript. However, as previously mentioned, the authors should add a brief discussion noting that these compounds were synthesized but excluded from further studies due to their excessive reactivity.

The wording in the manuscript has been refined to replace "thioacrylamide electrophiles" with "α-halothioamide electrophiles". Additionally, as described above, a brief note has been added to the discussion mentioning that thioacrylamide derivatives were synthesized but excluded from further studies due to their excessive reactivity and limited stability.

Page 5: "The methyl group on the adjacent β-carbon of the chloroacetamide sterically hinders the" The methyl group is actually attached at the α-position

The text has been corrected to indicate that the methyl group is attached at the α-position rather than the β-position.

Page 6: "covalently weakly reactive fluoroacetamide 13" Please delete "covalently"

The word "covalently" has been deleted, and the text now reads "*weakly reactive fluoroacetamide **13***."

Page 8: “a viable and advantageous late-stage warhead optimization strategy, enhancing potency without introducing additional off-target liabilities.” Softer phrasing is suggested, as the results presented here remain rather preliminary regarding their applicability to TCIs in a drug discovery context.

We agree that the original phrasing may overstate the translational relevance of the approach at this stage. We have therefore softened the language to more accurately reflect the actual data and examples reported herein. However, we note that our thiowarhead strategy demonstrates promising features, and we believe it may still hold potential for future application in TCI development. The sentence now reads as: *“Taken together, these results suggest that O-to-S exchange may represent a promising late-stage warhead optimization strategy, with the potential to enhance potency while maintain a favorable off-target profile.”*

Page 10 “The labelling efficiency of the 21 chloro- and the 22 fluorothioacetamide probes was comparable to that of the iodoacetamide (23) probe” This would imply that these warheads are overly reactive when it comes to TCI applications. On the other hand, the authors make the point that their kinase inhibitors show drug-like proteome labeling profiles, which seems contradictory. Please rephrase the relevant parts of the manuscript to properly reflect the data and the applied assay conditions. Given the rather broad reactivity of the chemoproteomic probes, I also suggest generally down-tuning the language regarding selectivity claims and potential TCI applications, while placing greater emphasis on the application potential as chemoproteomic probes.

We appreciate the reviewer’s comment and have revised the manuscript to clarify that the comparable labeling of the chloroacetamide (**21**) and fluorothioacetamide (**22**) probes relative to iodoacetamide (**23**) reflects the specific chemoproteomic assay conditions (high probe concentration in lysate), which are designed to probe intrinsic cysteine reactivity rather than evaluate target-selective behavior in living cells. To avoid overstating selectivity or therapeutic relevance, we have down-tuned statements regarding TCI applications and now place greater emphasis on the chemical biology utility of these electrophiles, particularly as chemoproteomic probes.

Page 11: “displaced the tracer with an IC50 of 54.4μM” Please provide the incubation time as this process is time-dependent. Does the calculation of a Ki via Cheng-Prusoff (see page 17) make sense given the time-dependent nature of the process?

We thank the reviewer for this important point. The incubation times have now been added to the protocol: 15 min at 25 °C for preincubation with the covalent probe, followed by 60 min incubation at 25 °C with the fluorescent tracer. We also appreciate the reviewer’s comment regarding the Cheng-Prusoff calculation. Given the time-dependent nature of covalent engagement, deriving a Ki from the IC_{50} is indeed not appropriate. We have therefore removed this calculation and now report only the displacement IC_{50} values from the fluorescence polarization assay to describe the functional consequences of covalent binding to PDE6δ.

Page 15 “cut between C and C” seems to have an issue in the converted pdf. Please check.

The issue has been checked and resolved by adjusting the font style.

Reviewer #3

The authors have thoughtfully considered the review suggestions and provided substantial additional experimental data. They have addressed the previous concerns. The added data and revisions have greatly enhanced the scientific value and significance of the revised manuscript. Therefore, I would recommend that you accept the paper for publication in Nature Communications without further revision.

We greatly appreciate the positive feedback and support, and we also thank for earlier suggestions, which genuinely helped strengthen and complete the manuscript.

Reviewer #4

The reviewer has included many additional experiments, and my concerns have been properly addressed in the revisions.

However, the current version of the manuscript contains compound numerous errors in descriptions and data presentation. Without correction of these errors, the paper cannot be published.

We thank this reviewer for the comments that were all addressed to complete our manuscript.

1. Compound numbers are highly frequently incorrect, e.g., in lines 117, 118, 223, 226, and 243.

We have carefully double-checked all compound numbers throughout the manuscript and corrected any inconsistencies.

2. In the response letter to reviewers, the IC□□ values for compounds 13 and 14 in response to Q7 are incorrect.

The numbering was unfortunately mixed up in the response letter; however, the correct IC□□ results were reported in the manuscript. Nevertheless, the numbering of all compounds has now been carefully double-checked (see our response to comment #1).

**Compound**	**Ramos cell viability IC_{50}**
**11** (ibrutinib)	0.94 ± 0.27 µM
**12** (chloroacetamide)	0.14 ± 0.04 µM
**13** (fluoroacetamide)	30.72 ± 9.86 µM
**14** (chlorothioacetamide)	0.21 ± 0.09 µM
**15** (fluorothioacetemide)	1.75 ± 0.81 µM

3. The legend in Figure S2 is confusing. Figure S2a should include more detailed information, specifying which peak corresponds to the covalent binding between the protein and the compound.

The figure has been updated to clearly indicate the peaks corresponding to the covalent adduct species, providing the requested clarification. or clarity and consistency, the scales have also been unified across the panels in Figure S2a (as shown in Figure A7).

Figure A7. *Intact protein MS spectrum of MAP2K6 modified by covalent probes (**3**-**6**).*

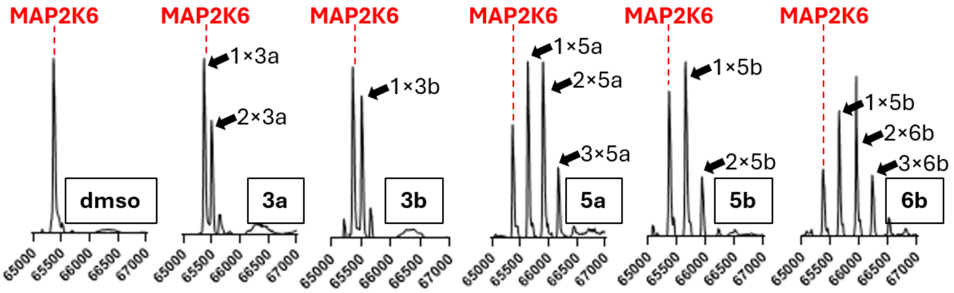

Besides the errors mentioned above, I suggested they can also revise the issues in below
1,The statement in Line 145, "while its selectivity over BTK was maintained," is not accurate. The selectivity fold over BTK for compound 7 is 5.6, while for compound 9 it is 2.65, indicating a significant decrease in selectivity.

We have revised the wording to accurately reflect the observed selectivity differences between compounds 7 and 9. While the selectivity decreases from 5.6× to 2.65×, we note that the ΔpIC□□$^{JAK3/BTK}$ values are 0.75 for compound 7 and 0.46 for compound 9. Therefore, we describe the effect of the oxygen–sulfur exchange as “the selectivity over BTK was not significantly altered”.

2, In Line 188, it is unclear why site mapping identification was not performed for compound 14, which contains the chlorothioacetamide moiety they developed.

Accepting the comment of the referee, we performed tryptic digestion on BTK labeled with compound **14** to confirm the site of modification. The MS data (Figure A8) revealed that, similar to compound **12** (re-measured under the same conditions for direct comparison), thioprobe **14** engages the same Cys481 residue, which has been also confirmed as reference for **11** Ibrutinib. As shown below in Figure A8, the quantification of labelled Cys481 clearly indicated that **12** and **14** probes (in hand with **11** Ibrutinib reference) significantly reduce the availability of Cys481 for iodoacetamide (CAM) labelling, while (on the right) we could also confirm the labelling of Cys481 with **12** and **14** probes after tryptic digestion of the labelled BTK (Figure A8).

Figure A8. *Tryptic digestion analysis of BTK protein labelled with **11**, **12** and **14** covalent probes.*

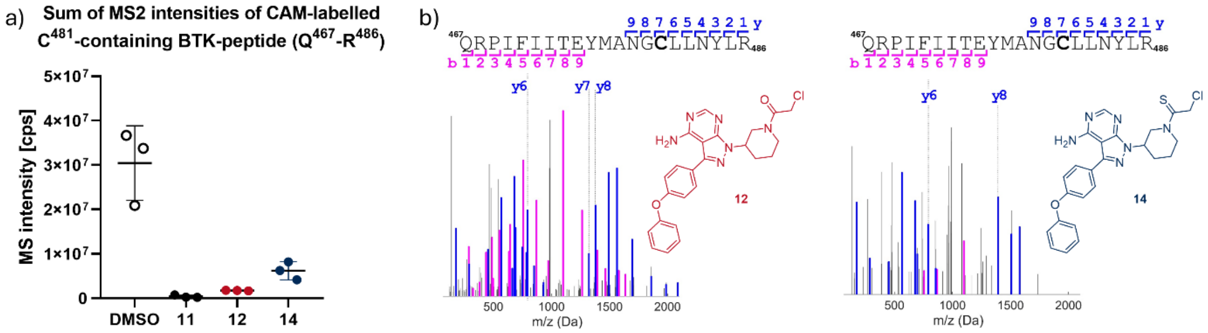

In addition, we conducted molecular dynamics simulations to evaluate the dynamic behaviour of these compounds within the binding pocket. We analysed three characteristic distances between the scaffold and the protein, as shown below, which confirmed that the probes adopt similar binding modes both in static and dynamic contexts. This is supported by the same key interactions between BTK and both the **12** and **14** probes. These interactions

are between Cys481 *N* and ligand *S/O* (d_1), between Glu475 backbone carbonyl and ligand NH_2 (d_2), and between the centroids of the aromatic rings of Phe570 and the ligand (d_3). The three distances (d_1, d_2, d_3) as functions of molecular dynamics simulation times are shown in Figure A9. Together, the MD trajectories clearly demonstrate that probes **12** and **14** share highly similar dynamic binding features, which, in hand with the tryptic digestion results, provides strong confidence that sulfurization does not alter the binding mode of the covalent inhibitors.

Figure A9. *α-Chloroacetamide (**12**) and α-chlorothioacetamide (**14**) binding modes to BTK.*

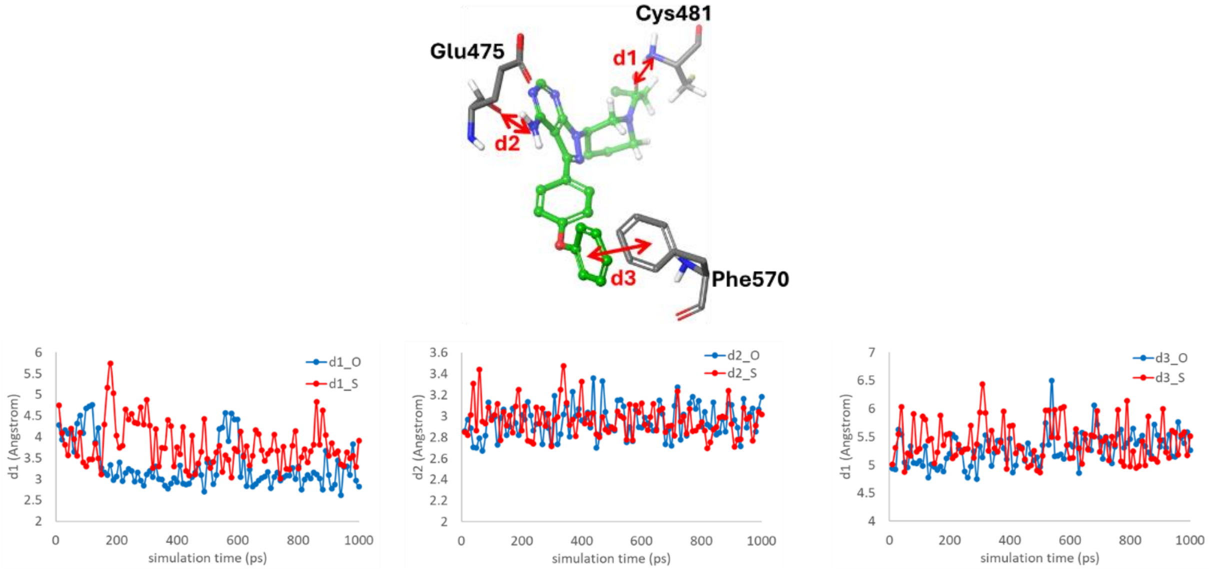

3,It is recommended to conduct kinome screening for BTK inhibitors 12 and 14, allowing a comparison of inhibition selectivity among compounds 11, 12, and 14.

We used kinase inhibitor scaffolds to demonstrate the applicability of thiohaloacetamides in TCI development, with JAK3 and BTK covalent inhibitors exemplifying this approach. Furthermore, the target scope of thiohaloacetamides was extensively investigated by chemoproteomic experiments, as discussed in the revised manuscript. Particularly, alkyne probes **16** (acrylamide), **17** (chloroacetamide), and **18** (chlorothioacetamide) were synthesized and profiled by pull-down chemoproteomic experiments (Figure A10). We believe the provided data and examples sufficiently support the concept and utility of the α-halothioacetamide warheads in covalent inhibitor design and covalent probe development. Therefore, we feel that full kinase profiling might be out of the scope of the present study.

Figure A10. *Proteome-wide target-scope analysis of ibrutinib-derivatives (**16-18**) showing the chemical structure of the applied probes (a) and the number of identified off-targets (b) in Mino cells treated with 1 or 10 μM of the probes.*

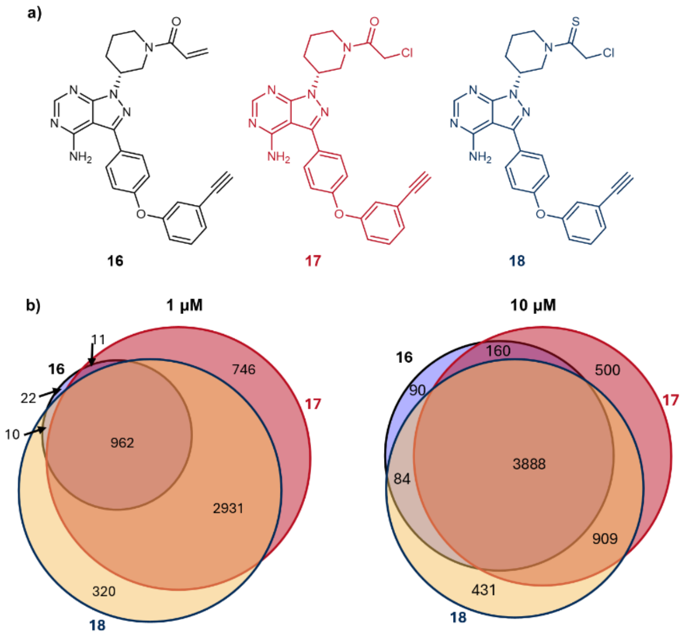

4, In Line 127, the claim that "thioamide does not increase cellular promiscuity" is only based on a comparison with the corresponding acetamide. However, when compared to the parent compound ibrutinib (which contains an acrylamide), thioamide significantly increases cellular promiscuity.

Our original statement referred specifically to the comparison between the **17** acetamide and the corresponding **18** thioacetamide analog. We have now clarified in the revised text that the O-to-S exchange within this matched pair does not appear to increase cellular promiscuity. We agree that relative to the parent compound ibrutinib containing an acrylamide warhead, the thioacetamide analogue shows higher promiscuity, and the wording has been adjusted accordingly to avoid ambiguity.

Reviewer #2 (Remarks to the Author):

The authors have revised their manuscript and have appropriately addressed the majority of this reviewer's comments. While the newly added NMR data are acceptable for publication, baseline correction and phasing could be improved, and signal intensities and purity are not ideal in some cases. A few minor points remain to be addressed:

- The authors have included a useful discussion of quantification based on intact MS data and protein-compound stoichiometries in their incubation experiments. However, it would be valuable to also comment on adduct stoichiometries, specifically the extent to which 1:1 adducts versus higher order adducts are observed.

Analysis of intact protein MS data showed that adduct formation was dominated by 1:1 protein–compound stoichiometries across all targets and compounds. We explicitly add this clarification to the manuscript to emphasize that the reported quantification reflects predominantly single-site modification events, consistent with selective cysteine engagement rather than nonspecific labelling. In fact, higher-order adducts (2× and 3× labeling) were only observed for MAP2K6 and exclusively with the most reactive covalent fragments (5a, 5b, 6b). This is consistent with the fragment-sized nature of the compounds, which lack extensive non-covalent secondary interactions and can therefore modify multiple accessible cysteine residues. We have now clarified this point in the manuscript. Further analysis of multiple MAP2K6 labelling revealed that the sequence includes multiple cysteines with most of them already captured in chemoproteomic target identification campaigns: Cys38[1–4], Cys196[4,5], Cys212[1] and Cys216.[3,5]

Text added to the manuscript: *"Across the majority of proteins and compounds, intact protein MS analysis revealed exclusively 1:1 protein-compound adducts. In fact, higher order adducts (double and triple labeling) were only observed for MAP2K6 after treated with the most reactive covalent fragments (5a, 5b, 6b). This behavior is consistent with the fragment-sized nature of the compounds, which at this early stage lack extensive non-covalent secondary interactions and may therefore modify multiple accessible cysteine residues, and is in line with MAP2K6 in chemoproteomic data that identified labelling multiple cysteine sites.[1–5]"*

1. Takahashi, M. *et al.* DrugMap: A quantitative pan-cancer analysis of cysteine ligandability. *Cell* **187**, 2536-2556.e30 (2024).
2. Heindel, A. J. *et al.* Chemoproteomic capture of RNA binding activity in living cells. *Nat. Commun.* **14**, 6282 (2023).
3. Abegg, D. *et al.* Chemoproteomic Profiling by Cysteine Fluoroalkylation Reveals Myrocin G as an Inhibitor of the Nonhomologous End Joining DNA Repair Pathway. *J. Am. Chem. Soc.* **143**, 20332–20342 (2021).
4. Koo, T.-Y., Lai, H., Nomura, D. K. & Chung, C. Y.-S. N-Acryloylindole-alkyne (NAIA) enables imaging and profiling new ligandable cysteines and oxidized thiols by chemoproteomics. *Nat. Commun.* **14**, 3564 (2023).
5. He, J.-X. *et al.* A modification-centric assessment tool for the performance of chemoproteomic probes. *Nat. Chem. Biol.* **18**, 904–912 (2022).

- The removal of phenylacrylamide from both the experimental set and the discussion is not appropriate, even though thioacrylamides were excluded. Phenylacrylamide remains an important benchmark compound that enables comparability with other studies. The authors should re-include this compound in their evaluation, explicitly noting that it is used as a benchmark and highlighting that it appears significantly less reactive in this study compared with many previous reports. This information is important for readers to properly rank the reactivity range of the compounds examined here

Although acrylamide- and thioacrylamide-derived compounds are not central to the main scope of this study due to their limitations in stability, accessibility, and broader relevance compared with haloacetamides, we agree that phenylacrylamide remains useful for contextual comparison with prior studies. Accordingly, phenylacrylamide is now added as a benchmark compound. In addition, we have added a clarifying note highlighting that sulfuration of phenylacrylamide to the corresponding thioacrylamide leads to a pronounced increase of intrinsic reactivity, with the resulting compound exhibiting hyperreactive behavior, showing all reactions and measured thiol-activities in Supplementary Scheme S1. These results also demonstrate the substantial impact of acrylamide-to-thioacrylamide transformation on electrophilic reactivity, while remaining consistent with the overall focus and conclusions of the study.

Text added to the manuscript: *"In addition to haloacetamides, the strategy was evaluated on acrylamide substrates (Supplementary Scheme S1), including phenylacrylamide (S3a), which can be considered as a benchmark electrophile to enable contextual comparison with other studies. While thioacrylamides could in principle be formed in a similar reaction, only phenylthioacrylamide proved sufficiently stable to be isolated in pure form. In contrast, five additional thioacrylamide derivatives could not be obtained due to rapid secondary reactivity and decomposition during workup and purification. Notably, conversion of phenylacrylamide (S3a) to the corresponding thioacrylamide (S6a) resulted in pronounced increase of electrophilic reactivity, displaying hyperreactive behavior in GSH-assay. However, owing to their limited stability and accessibility of acrylamide-derived thioamides, these compounds were not pursued further in this study."*

- With regard to the added discussion on chemoproteomic reactivities and assay conditions, the current wording remains somewhat unconvincing. If the

compounds are indeed less reactive than IAA, one would generally expect reduced levels of labeling. Additional discussion addressing potential reasons for the apparent differences in promiscuity between the chemoproteomic probes and other compounds in the set would be helpful.

Alhough the compounds investigated here are indeed intrinsically less reactive than the iodoacetamide probe (**23**), their reduced intrinsic electrophilicity does not necessarily translate into proportionally reduced proteome-wide labeling in chemoproteomic experiments. Several factors may contribute to the observed differences in apparent promiscuity. First, cysteine residues display a wide range of effective nucleophilicities that are strongly influenced by their local protein microenvironments (e.g., pKa perturbation or solvent exposure), which can selectively enhance reactivity toward less electrophilic warheads. Second, direct quantitative comparison across probes is intrinsically challenging due to experimental differences, including probe concentration and cellular context (e.g., 10 μM in Mino cells vs. 100 μM in HEK293 cells

Importantly, additional technical considerations further complicate direct interpretation of “promiscuity.” These include (1) scaffold-dependent differences in click chemistry efficiency, which can influence protein detection independently of cysteine engagement; (2) the fact that a single identified protein may harbor multiple labeled cysteines; and (3) possible differences in the stability of the covalent linkages formed, which may affect peptide recovery and identification. Collectively, these factors indicate that apparent promiscuity reflects a convolution of intrinsic reactivity, target microenvironment, and assay-specific parameters rather than electrophilicity alone. Nevertheless, we have partially revised the discussion to better articulate these mechanistic considerations and to avoid the oversimplified implication that lower intrinsic reactivity must directly correspond to reduced labeling levels.

Text added to the manuscript: *“Reduced electrophilicity, however, does not necessarily translate into proportionally reduced chemoproteomic labeling, as local cysteine microenvironments and experimental conditions can substantially modulate apparent proteome-wide engagement, thereby explaining the observed differences in labeling promiscuity across the chemoproteomic probes in the set.”*

Keseru and London et al. developed a relatively new cysteine-targeting warheads by transforming traditional carboxamide based warheads to soft electrophilic thioamides. The transformed electrophiles (including chlorothioacetamides and fluorothioacetamides) demonstrated enhanced reactivity and specificity for cysteine labeling while preserving the original warhead geometry. The authors further demonstrated these warheads can be used to develop BTK inhibitors and covalent modify antibody. In addition, the chlorothioacetamides compound (18) was able to covalently bind with more than 4000 proteins. This work can enrich the toolbox of cysteine-targeting warheads, offering new electrophiles for development of TCIs and chemical biology tools. However, the characterization of this kind of warhead is not thorough, and the overall advantages of this kind of warhead are not so significant. So it is recommended to include more results, current version was not good enough to be published in Nature Communications, but it can be given an opportunity to revise it. I will make the final decision based on the revised version.

Major Concerns:

1, The authors claimed the O-S exchange strategy enables precise late-stage modulation of covalent reactivity, which was proved only in Ibrutinib. The authors should include one more case with different structures, to demonstrate its feasibility on more structurally diversity compounds

2， The authors need to test covalent reaction kinetics of 12/13/15 with BTK, which can demonstrate and compare the covalent binding efficiency of inhibitors containing different warheads with proteins.

3, The authors should explain properly why they chose “*N*-benzyl-2-chloroethanethioamide”-based compounds in Fig.3/4 instead of “2-chloro-*N*-phenylacetamide” what they used in the beginning, these two versions may demonstrate different reactivities.

4, The results in Figure 3c are inconsistent with those described in S6. Please explain or describe them more clearly.

5, The fluorothioacetamides-containing compound 15 demonstrated significantly faster covalent binding rate with BTK comparing with chlorothioacetamides-containing compound 13, however, chlorothioacetamides-containing compound 18 displayed stronger reactivity with cysteine in comparing with fluorothioacetamides-containing compound 20

6, The authors claimed O-S exchange to this warheads improved its specificity for cysteine, so it’s strongly suggested that the authors should determine proteomewide residue selectivity of the warheads they developed using residue-specific proteomics.

7, As warheads used for development of TCIs, the cellular potencies of chlorothioacetamides and fluorothioacetamides were also need to be evaluated, should include a piece of data to demonstrate the cellular potency for either chlorothioacetamides or fluorothioacetamides, such as evaluating the cellular potency of compound 13 or 15. Especially compound 18 or 20 show poor labeling efficiency in S6

Minor Issues:

4, , Line 481 in maintext “followed by 45 incubation at 25°C”

1, Didn’t find Supplementary Table S7 which was mentioned line 224 in the maintext.

2, The authors claimed that “The labelling efficiency of the fluoro (20) and chloro (18) thioacetamide probes was comparable to that of the chloro (17) and iodo acetamide (IAA) probes, respectively” in the line 221-222 according to the results in S6. This statement was not true according to the results in S6. And the results quality in S6 was not good enough, especially for compound 19.

3, The starting molecular weight values in Figure S5 should be same, for easy reading.

4, , Line 481 in maintext “followed by 45 incubation at 25 °C”
